# Proceedings 35th Symposium ESVN‐ECVN

**DOI:** 10.1111/jvim.70017

**Published:** 2025-02-19

*The European College of Veterinary Neurology (ECVN) Symposium and the Journal of Veterinary Internal Medicine (JVIM) are not responsible for the content or dosage recommendations in the abstracts. The abstracts are not peer‐reviewed before publication. The opinions expressed in the abstracts are those of the author(s) and may not represent the views or position of the ECVN. The authors are solely responsible for the content of the abstracts*.

**Table:** 

**NEUROSURGERY**	
**Main Conference**	
	**DAY 1 Friday 22 September**
**7.30**	**Registration**
	**CP: Balducci F**.
**8.30**	**Opening Ceremony and Welcome**.
**8.45**	**Keynote Lecture: Neurosurgical Options for Glioma (Fioravanti Antonio)**
**9.30**	**Keynote Lecture: Surgical treatment of Chiari Malformation (Fioravanti Antonio)**
10.15	Coffee Break and Exhibition
	CP: Luisa De Risio
**11.00**	**Oral Presentations (6 × 8 m)**
	**O1**
	**O2**
	**O3**
	**O4**
	**O5**
	**O6**
**11.50**	**Surgical Treatment of Brain Tumors: Obstacles and Opportunities, Part I: (Rossmeisl)**
**12.35**	**Flash Presentations (7 × 4 m)**
	**FP1**
	**FP2**
	**FP3**
	**FP4**
	**FP5**
	**FP6**
	**FP7**
13.05	Lunch Break and Exhibition and Poster Discussion
	CP: D'Angelo A.
**14.30**	**Surgical Treatment of Brain Tumors: Obstacles and opportunities, Part II: (Rossmeisl)**
**15.15**	**3D Printing: Applications in Spinal Surgery (Sebastien Behr)**
15.35	Coffee break and Exhibition
**16.00**	**AGM**
**18.00**	**Fine Della Giornata**
	**Day 2 Saturday 23 September**
	**CP: Fadda A**.
**9.00**	**Oral Presentations (6 × 8 m)**
	**O7**
	**O8**
	**O9**
	**O10**
	**O11**
	**O12**
**9.50**	**Keynote Lecture: Degenerative Lumbosacral Stenosis: Surgical Treatment. Frank Steffen**
10.35	Coffee Break and Exhibition
	CP Falzone C.
**11.15**	**Oral Presentations (6 × 8 m)**
	**O13**
	**O14**
	**O15**
	**O16**
	**O17**
	**O18**
**12.05**	**Keynote Lecture: Surgical Treatment of Thoracic Vertebral Malformation: Massimo Baroni**
**12.50**	**Flash Presentations**
	**(7 × 4 m)**
	**Fp8**
	**FP9**
	**FP10**
	**FP11**
	**FP12**
	**FP13**
	**FP14**
**13.20**	**Presentation ESVN‐ECVN Annual Meeting 2024**
13.30	Lunch Break and Exhibition and Poster Discussion
	CP: Simona Redaelli
14.45	Flash Presentation
	**(7 × 4 m)**
	**FP15**
	**FP16**
	**FP17**
	**FP18**
	**FP19**
	**FP20**
	**FP21**
**15.15**	**Keynote Lecture: Neurosurgical Options for Disc Associated Cervical Spondylomyelopathy: Daniele Corlazzoli**
16.00	Coffee Break and Exhibition
	CP: Arianna Maiolini
**16.30**	**Oral Presentations (7 × 8 m)**
	**O19**
	**O20**
	**O21**
	**O22**
	**O23**
	**O24**
	**025**
**17.30**	**Awards and Closing Remarks**
**RESIDENT DAY**	
**Cristian Falzone/Frank Steffen**	
8.30	Registration
9.00–10.30	Surgical Approach to the Thoracolumbar Spine: Hemilaminectomy/Minihemilaminectomy/Disk Fenestration
	Dr. Cristian Falzone
10.30	Coffee Break
11.00–12.30	Surgical Approach to the Cervical Spine: Ventral Slot/Dorsal Laminectomy/Hemilaminectomy Prof. Frank Steffen
12.30	Lunch Break
13.30	Surgical Approach to the Lumbosacral Spine: Dorsal Laminectomy, Lateral Foraminotomy and Vertebral Stabilization Prof. Frank Steffen
14.45	Coffee Break
15.15	Surgical Treatment of Vertebral Fractures and Subluxation
	Dr. Cristian Falzone
16.30	End of the Day
	Recommended Lecture, With Particular Emphasis on the Related Topics of the Day: Small Animal Spinal Disorders, 2nd Edition, Nicholas JH Sharp and Simon J Wheeler, Elsevier 2005
	Chair Person: Federica Balducci
	Proposte Chair Person: Una Mezza Sessione a Testa
	OPZIONE 1: TOTALE 7 Sessioni
	Balducci F. (mezza mattina 1° gg)
	Falzone C. (Mezza Mattina 1° gg)
	De Risio L.
	Gutierrez Quintana R.
	Stalin C.
	Goncalves R.
	Stein V.
	OPZIONE 2
	Balducci F. (Mezza Mattina 1° gg)
	Falzone C. (Mezza Mattina 1° gg la Seconda)
	De Risio L.
	Veronika S.
	Cizinauskas S.
	Alejandro Luján Feliu‐Pascual
	De Decker S.
	X Cri sono un po' troppi. Suff. 2 chair per giornata. Propone tutti italiani.
	De Risio
	(Angela Fadda)
	Falzone C
	Balducci F
	Monica.borghisani@evsr.it

## 1 Oral Presentations

## 2 (OP 01)

### 2.1 Complex Partial Seizures With Orofacial Involvement in 35 Cats: Hippocampal Changes on MRI, CSF Analysis, Voltage‐Gated Potassium Channel Antibodies and Clinical Outcome

#### 2.1.1 T. Flegel, Dr. Med. Vet, DECVN, DACVIM,^1^ M. Füßer, DVM,^1^ L. F. Becker, DVM,^1^ Corresponding Address I. C. Böttcher, Dr. Med. Vet., DECVN, J. Dietzel, DVM, S. Gutmann, Dr. Med. Vet., T. Kalliwoda, DVM,^1^ S. Loderstedt, Dr. Med. Vet., DECVN,^1^ C. Tästensen, Dr. Med. Vet.,^1^ V. Weiß, DVM,^1^ L. P. Schulte, student,^1^ J. König, student^1^

##### 2.1.1.1 ^1^ Department for Small Animals, Leipzig University, Leipzig, Germany

The aim of this study in cats with complex partial seizures with orofacial involvement (CPSOFI) was to determine (1) the incidence of hippocampal MRI changes, antibodies against voltage‐gated potassium channels (VGKC Ab), (2) to investigate if one of the following factors could serve as a prognostic indicator: signalment, MRI changes, VGKC Ab, and CSF findings.

The following information was retrospectively extracted from the hospital data base: signalment, seizure semiology (CPSOFI alone or in combination with generalized tonic–clonic seizures), brain MRI findings, VGKC Ab, CSF findings, treatment, and follow up. Thirty‐five cats were included. In 20/35 cats (57.1%) CPSOFI was the only type of seizure seen, whereas 11/35 cats (31.4%) experienced CPSOFI as well as generalized tonic–clonic seizures. MRI hyperintensity of the hippocampus was seen on T2 and/or FLAIR images in 20/35 cats (57.1%). Serum VGKC Ab was found in 11/35 cats (31.4%). Ten of those were positive for Lgl1 antibodies, whereas one cat tested positive for CASPR2 antibodies. There was a weak correlation between hippocampal hyperintensity and being positive for antibodies against VGKC (PHI coefficient 0.12; *n* = 22). The mean survival time of cats being deceased was 485 days, whereas the follow‐up time of cats still alive was 868 days. None of the factors: age at first seizure, breed, sex, T2/FLAIR hyperintensity, being positive for VGKC antibodies, and CSF findings did have any influence on survival. Neither VGKC Ab nor T2/FLAIR hyperintensity influences prognosis. VGKC Ab and hyperintensity show only a weak correlation which may fuel the discussion about the underlying pathology again.

## 3 (OP 02)

### 3.1 Enteric Neuron Activation: The Potential Route of Brain Stimulation in Canine Idiopathic Epilepsy

#### 3.1.1 A. Watanangura, DVM,^1^ ^,4^ K. Elfers, PhD,^2^ P. Hoffmann, PhD,^2^ J. S. Suchodolski, MedVet, Dr. Med Vet, PhD, AGAF, DACVM,^5^ M. R. Khattab, BVSc, MVSc, PhD,^5^ R. Pilla, DVM, PhD,^5^ S. Meller, DVM, PhD,^1^ H. A. Volk, DECVN, PhD, PGCAP, FHEA, MRCVS,^1^ ^,3^ G. Mazzuoli‐Weber, Dr. Med Vet, PhD^2^ ^,3^

##### 3.1.1.1 ^1^ Department of Small Animal Medicine and Surgery, University of Veterinary Medicine Hannover, Hannover, Germany; ^2^ Institute for Physiology and Cell Biology, University of Veterinary Medicine Hannover, Hannover, Germany; ^3^ Center for Systems Neuroscience (ZSN), Hannover, Germany; ^4^ Veterinary Research and Academic Service, Faculty of Veterinary Medicine, Kasetsart University, Nakhon Pathom, Thailand; ^5^ Gastrointestinal Laboratory, Department of Small Animal Clinical Sciences, Texas A&M University, Texas, USA

There is growing evidence that gastrointestinal microbiota (GIM) are involved in epilepsy through the microbiota–gut–brain axis. However, how GIM and its metabolites potentially interact with the enteric nervous system is still unknown. The aim of the study was to investigate the effects of GIM and microbial products on cultured myenteric neurons. Fecal supernatants (FS) from healthy dogs and dogs with idiopathic epilepsy including drug‐naive, phenobarbital responsive‐ and non‐responsive dogs were examined on cultured myenteric neurons from guinea pigs using voltage‐sensitive dye neuroimaging to evaluate action potential induction ability. The concentration of short‐chain fatty acids (SCFA) contained in FS was measured. The results showed that FS was able to induce neuronal action potential discharge. FS from PB non‐responsive dogs activated a significantly higher number of myenteric neurons compared with healthy controls and the highest number of neurons among all groups. However, the induced burst frequency was highest after the application of FS from drug‐naïve with a significant difference compared with FS from PB non‐responsive dogs and a statistical trend compared with healthy controls. The results show the ability of FS to evoke neuronal activity dependent on the treatment status of the patients. The lower SCFA level of acetate and isobutyrate can partly explain the effect of FS from PB non‐responsive dogs. Consequently, this study provides further evidence that the GIM and the enteric nervous system are involved in epilepsy and could provide the basis for a novel therapeutic approach.

## 4 (OP 03)

### 4.1 Fecal Microbiota Transplantation as a Novel Tool for Behavioral Comorbidities Treatment in Canine Idiopathic Epilepsy – A Pilot Study

#### 4.1.1 A. Watanangura, DVM,^1^ ^,2^ S. Meller, DVM, PhD,^1^ N. Farhat, BSc,^3^ J. S. Suchodolski, MedVet, Dr. Med Vet, PhD, AGAF, DACVM,^4^ R. Pilla, DVM, PhD,^4^ M. R. Khattab, BVSc, MVSc, PhD,^4^ A. Bathen‐Nöthen, Dr. Med Vet, DECVN,^5^ A. Fischer, Dr. Med Vet, DECVN, DACVIM,^6^ K. Busch‐Hahn, Dr. Med Vet, DECVIM‐CA,^6^ C. Flieshardt, Dr. Med Vet, DECVN,^7^ M. Gramer, CTA,^8^ F. Richter, Dr. Med Vet, PhD,^8^ ^,9^ A. Zamansky, BA, MSc, PhD,^3^ H. A. Volk, DECVN, PhD, PGCAP, FHEA, MRCVS^1^ ^,9^

##### 4.1.1.1 ^1^ Department of Small Animal Medicine and Surgery, University of Veterinary Medicine Hannover, Hannover, Germany; ^2^ Veterinary Research and Academic Service, Faculty of Veterinary Medicine, Kasetsart University, Nakhon Pathom, Thailand; ^3^Information Systems Department, University of Haifa, Haifa, Israel; ^4^ Gastrointestinal Laboratory, Department of Small Animal Clinical Sciences, College of Veterinary Medicine and Biomedical Sciences, Texas A&M University, Texas, USA; ^5^ Tierarztpraxis, Dr. A. Bathen‐Nöthen, Cologne, Germany; ^6^ Small Animal Clinic, Centre for Clinical Veterinary Medicine, Ludwig‐Maximilians‐University, Munich, Germany; ^7^ Small Animal Clinic, Tierklinik Posthausen, Posthausen, Germany; ^8^ Department of Pharmacology, Toxicology, and Pharmacy, University of Veterinary Medicine Hannover, Hannover, Germany; ^9^ Center for Systems Neuroscience (ZSN), Hannover, Germany

Anxiety and attention deficit hyperactivity disorders (ADHD) are well‐known behavioral comorbidities in canine idiopathic epilepsy (IE). Many neurological and neuropsychiatric disorders in dogs and humans including epilepsy have been reported to have altered gastrointestinal microbiota (GIM). Fecal microbiota transplantation (FMT) is known to modify GIM. The primary aim of the study was to evaluate the effect of FMT on behavior in canine epilepsy. Nine dogs with IE tier II confidence levels with drug‐resistant epilepsy were included. The fecal donor was a long‐term seizure‐free IE‐patient under phenobarbital treatment. FMTs were performed three times 2 weeks apart with follow‐ups at 3 and 6 months thereafter. Behavior was analyzed using validated questionnaires and behavioral tests with computational analysis. Urine and fecal samples were collected and analyzed for urine neurotransmitter concentration and GIM by shallow DNA shotgun sequencing, respectively. The results revealed improvement in anxiety and ADHD‐like behavior. Only changes in individual microbiota taxa were observed after FMT, with a Firmicutes and a Blautia_A species decreasing, and a Ruminococcus species increasing. Urine neurotransmitter analysis revealed decreased excitatory neurotransmitters including aspartate and glutamate and increased inhibitory neurotransmitter GABA, as well as GABA/glutamate ratio after FMT. FMT decreased > 50% of seizure cluster day frequency in 4/9 dogs at the 6 months follow‐up appointment, as well as a decrease in seizure duration and intensity in 6/9 dogs. This pilot study suggests that FMT can modify behavior and potentially modulate seizure severity in some dogs with IE. Further studies are required to validate the initial positive results.

## 5 (OP 04)

### 5.1 A Novel Epileptic Encephalopathy Affecting Bengal Cats: Can Uridine Therapy Help?

#### 5.1.1 Kaczmarska, DVM MVM,^1^ M. Christen, DVM,^2^ F. del Caño‐Ochoa, PhD,^3^ S. Ramon‐Maiques, PhD,^3^ ^,4^ A. Cloquell Miro, DVM MSc,^1^ A. Rupp, DVM PhD,^1^ V. Jagannathan, PhD,^2^ T. Leed, PhD,^2^ ^,5^ R. Gutierrez‐Quintana, MVZ MVM^1^ ^,5^

##### 5.1.1.1 ^1^ School of Biodiversity, One Health and Veterinary Medicine, College of Medical, Veterinary and Life Sciences, University of Glasgow, Glasgow, UK; ^2^ Institute of Genetics, Vetsuisse Faculty, University of Bern, Bern, Switzerland; ^3^ Instituto de Biomedicina de Valencia (IBV), CSIC, Valencia, Spain; ^4^ Group 739, Centro de Investigación Biomédica en Red de Enfermedades Raras (CIBERER)–Instituto de Salud Carlos III, Valencia, Spain; ^5^ Shared Senior Authorship, Glasgow, UK

Epileptic encephalopathies are severe brain disorders showing intractable seizures at a young age. Progress in identifying their molecular basis has translated into effective treatment for some of these conditions. We examined a 4‐month‐old Bengal kitten with intractable seizures and abnormal behavior. Physical and neurological examinations were unremarkable, except for abnormal mentation. Investigations including hematology, biochemistry, infectious diseases titers, brain magnetic resonance imaging, and cerebrospinal fluid analysis were normal. The kitten received phenobarbital and levetiracetam, but the owners elected euthanasia due to frequent seizures. Necropsy showed neuronal vacuolation affecting the deep cerebral cortex and thalamus. Autosomal recessive inheritance was suspected as parents and litter mates were unaffected. The genome was sequenced, and the data was compared with 80 control cat genomes. A private homozygous variant in the CAD gene encoding an enzyme required for pyrimidine de novo synthesis was identified, XP_006930506.2:(p.Ser2032Asn). Genotypes of 110 unaffected Bengal cats identified 4 additional carriers, confirming its presence in the breed. CAD variants cause uridine‐responsive epileptic encephalopathy in humans. Using CRSPR/Cas9 the functional effect of the variant was assessed by testing its ability to rescue growth of a human CAD‐knockout cell line that requires uridine supplements for survival. The mutant enzyme did not rescue the growth phenotype, suggesting that this kitten could have benefited from uridine therapy. We report a novel CAD variant causing epileptic encephalopathy in young Bengal cats. These findings enable genetic testing to detect carriers and affected animals, as well as a potential treatment for affected kittens by supplementing uridine.

## 6 (OP 05)

### 6.1 Behavioral Changes as Feature of Temporal Lobe Epilepsy Confirmed With Electroencephalography Examination and Good Response to Antiepileptic Treatment in Dogs

#### 6.1.1 A. Czerwik, DVM, PhD, A. Olszewska, DVM, Dr., A. Knittel, DVM, M. J. Schmidt, DVM, Prof, Dr., Dipl. ECVN^1^

##### 6.1.1.1 ^1^ Neurology and Neurosurgery Department of Veterinary Clinical Sciences, Small Animal Clinic, Justus‐Liebig‐University, Giessen, Germany

In humans, temporal lobe seizures can progress to another type of seizure, such as focal impaired awareness seizures with symptoms like staring into the space, confusion, and repetitive behaviors (i.e., lip‐smacking, chewing). In veterinary medicine, epileptic motor activity can be focal or generalized as well as accompanied by sensory impairment, which can be mistaken for behavioral disorders. Differentiation between them can be challenging. Confirmation of the epileptic nature of those events can be only obtained with electroencephalography. The aim of this study was to present the neurological and electroencephalographic diagnostic workup of such behavioral incidents in 2 dogs. A 2‐year‐old Border Terrier and 1‐year‐old Poodle‐mix breed dog were presented with a history of recurrent episodes of fear, restlessness, hyperactivity, hiding, staring into space/at the sky, and lip licking with impaired consciousness. Each episode lasted a few minutes, multiple times per day. No other symptoms were observed before and after the episodes. Clinical and neurological examination was normal. Electroencephalography using Nihon Kohden and magnetic resonance imaging of the brain using Siemens 1.5 T was performed and it was normal. The 4‐h EEG showed interictal epileptic uni‐ and bilateral discharges from the parietal and temporal channeling with synchronous activity from the temporal lobe. The current case series describes the occurrence of behavioral symptoms resulting from temporal lobe epilepsy diagnosed by electroencephalography. Both dogs were treated with phenobarbital, and they showed significant improvement with the decrease in frequency of the episodes as well as decrease in intensity.

## 7 (OP 06)

### 7.1 The Idiopathic Epilepsy Mystery: Cerebrospinal Fluid Metabolomics

#### 7.1.1 F. Verdoodt, MVetMed,^1^ ^,3^ L. van Hemeryck, PhD,^2^ L. Vanhaecke, Prof.,^2^ L. van Ham, Prof.,^3^ M. Hesta, Prof.,^1^ S. Bhatti, PhD^3^

##### 7.1.1.1 ^1^ Equine and Companion Animal Nutrition, Department of Morphology, Imaging, Orthopedics, Rehabilitation and Nutrition, Faculty of Veterinary Medicine, Ghent University, Merelbeke, Belgium; ^2^ Laboratory of Integrative Metabolomics, Department of Translational Physiology, Infectiology and Public Health, Faculty of Veterinary Medicine, Ghent University, Merelbeke, Belgium; ^3^ Small Animal Department, Faculty of Veterinary Medicine, Ghent University, Merelbeke, Belgium

Cerebrospinal fluid (CSF) metabolomics is a promising approach for the elucidation of canine idiopathic epilepsy (IE). A liquid chromatography coupled to mass spectrometry (UHPLC‐HRMS) method for polar metabolomics in CSF was developed to this extent. As a proof‐of‐principle, diagnostic CSF samples were analyzed comparing the CSF metabolome of 8 dogs with IE Tier II (De Risio et al. 2015) (mean age: 4.7 ± 3 years; 6 male, 2 female) and 7 dogs with non‐brain related disease (mean age: 6.7 ± 4 years; 4 male, 3 female). Data were normalized, log‐transformed and Pareto scaled before untargeted multivariate modeling, and a selection of 42 target metabolites was monitored as well. The CSF of dogs with IE showed significantly lower levels of glutamic acid and eicosapentaenoic acid (EPA) compared with non‐brain related diseases, but significantly higher levels of cortisol and epinephrine, indicating a link with stress metabolism. Glutaric acid and glucose levels were also higher in dogs with IE and the latter may be explained by the energy shortage theory for seizure generation (Han et al. 2021). Untargeted metabolome fingerprints allowed to discriminate for disease (R2y = 0.99, Q2y = 0.83, CV‐ANOVA *p* < 0.001). The valid model showed 61 potential discriminating metabolic features (based on Jack‐Knifed confidence intervals, position in the S‐plot, and VIP‐score > 1.5), of which some could be putatively annotated, including norepinephrine as a marker for IE. In conclusion, these findings support the further elucidation of the involvement of EPA, known for its neuroprotective properties (Dyall 2015) and stress and energy metabolism in the pathophysiology of IE.

## 8 (OP 07)

### 8.1 Thoracolumbar Hydrated Nucleus Pulposus Extrusion and Intervertebral Disc Extrusion in Dogs: Comparison of Clinical Presentation and Magnetic Resonance Imaging Findings

#### 8.1.1 E. Alcoverro, DVM, Dip. ECVN, MRCVS, I. Schofield, BVSc, MSc (VetEpi), PhD, MRCVS, S. Spinillo, DVM, GPCert (Neuro), GPCert (SAS), MRCVS, A. Tauro, DVM, Dip. ECVN, GPCert (Neuro), MRCVS,^1^ ^,4^ M. Ruggeri, DVM, MRCVS,^1^ M. Lowrie, MA, VetMB, MVM, Dip. ECVN, MRCVS,^3^ ^,5^ S. Gomes, DVM, MRes, Dip. ECVN, MRCVS^3^

##### 8.1.1.1 ^1^ ChesterGates Veterinary Specialists, Chester, UK; ^2^ CVS Referrals, Norfolk, UK; ^3^ Dovecote Veterinary Hospital, Castle Donington, UK; ^4^ North Carolina State University College of Veterinary Medicine, Raleigh, USA; ^5^ Movement Referrals: Independent Veterinary Specialists, Runcorn, UK

Introduction/Purpose: In dogs, thoracolumbar (TL) myelopathies caused by hydrated nucleus pulposus extrusion (HNPE) and intervertebral disc extrusion (TL‐IVDE) may share some similarities. The aim of this study was to analyze the differences in clinical presentation between a group of dogs with TL‐HNPE, and a group with TL‐IVDE, and to study the MRI findings.

Methods: Retrospective case–control study. Medical records of two referral hospitals were searched for cases of TL‐HNPE and TL‐IVDE (controls).

Results: Fifty‐one dogs with TL‐HNPE and 105 with TL‐IVDE were included. Dogs with TL‐HNPE were significantly older and less likely to be of chondrodystrophic breed than those with TL‐IVDE (both *p* < 0.001). The onset of clinical signs in TL‐HNPE was more commonly acute/peracute (*p* < 0.001), associated with a traumatic event or exuberant exercise (*p* < 0.001) and with a history of vocalization at onset (*p* = 0.039); whereas hyperesthesia at presentation and progression of clinical signs were more common in TL‐IVDE (both *p* < 0.001). On MRI, there was a greater degree of cross‐sectional spinal cord compression in TL‐IVDE (51.0%) than in TL‐HNPE (34.3%; *p* < 0.001). The herniated disc material with TL‐HNPE was more commonly confined to the intervertebral disc space (IVDS), whereas in the TL‐IVDE it was beyond the IVDS.

Discussion/Conclusion: Our findings reveal some key differences in the signalment and clinical presentation between TL‐HNPE and TL‐IVDE that may aid in clinical reasoning when approaching dogs with signs of thoracolumbar myelopathy. These alongside the MRI findings may help understand the pathophysiology of TL‐HNPE.

## 9 (OP 08)

### 9.1 Effect of Epidural Application of Methylprednisolone Acetate on Time to Ambulation in Non‐Ambulatory Dogs With Thoracolumbar IVDE After Surgical Decompression. A Prospective Study

#### 9.1.1 P. Natsios, DVM, MSc., L. Golini, MSc, Dipl ECVN, MRCVS, B. Park, PhD, F. Steffen, Dipl. ECVN, Prof

##### 9.1.1.1 ^1^ Vetsuisse Faculty University of Zurich, Clinic for Small Animal Surgery, Zurich, Switzerland; ^2^ Vetsuisse Faculty University of Zurich, Section of Neurology, Zurich, Switzerland

Objectives: To analyze the benefit of epidural steroid application at the time of surgical decompression on motor recovery in non‐ambulatory paraparetic dogs with intervertebral disc extrusion.

Study Design: Randomized blinded control trial.

Animals: A total of 41 dogs presented with non‐ambulatory paraparesis due to thoracolumbar disc extrusion.

Methods: Dogs were randomly allocated to a control and treatment group. Dogs of both groups were treated with surgical decompression. After surgical removal of extruded disc material NaCl was locally applied in the control group (*n* = 23). In the treatment group (*n* = 18), methylprednisolone acetate was applied. Time to ambulation, defined as the number of days required to make 10 independent steps, was recorded for each dog.

Results: The median number of days to ambulation was 7 days (Range: 1–17 days) in the control group and 3 days (Range: 1–8 days) in the treatment group. In the methylprednisolone acetate group, the time to ambulation was significantly shorter compared with the control group (*p* < 0.001, Mann–Whiney *U* test). No side effects were noted during the study period.

Conclusion: Epidural application of methylprednisolone acetate in combination with surgical decompression shortened the time to ambulation in non‐ambulatory dogs caused by intervertebral disc extrusion.

Clinical Significance: Local application of Methylprednisolone acetate is a feasible and effective add‐on treatment to surgical decompression in dogs with acute spinal cord trauma due to intervertebral disc extrusion.

## 10 (OP 09)

### 10.1 TIRC7 Expression Might Serve as Activation Marker in Canine Lymphocytes in Non‐Inflammatory Canine Neurological Diseases

#### 10.1.1 S. Perret, Veterinarian,^1^ T. Löwe, Veterinarian,^1^ R. Carlson, Technician,^1^ J. Nessler, Dr. Med. Vet., Dipl. ECVN, EBVS,^1^ E. Packeiser, Dr. Med. Vet.,^1^ K. Hülskotter, PhD,^3^ W. Baumgärtner, Prof., Dr. Med. Vet., PhD, Dipl. ECVP/ACVP,^3^ N. Utku, PD, Dr. Med.,^2^ A. Tipold, Prof., Dr. Med. Vet., Dipl. ECVN^1^

##### 10.1.1.1 ^1^Department of Small Animal Medicine and Surgery, University of Veterinary Medicine, Hanover, Germany; ^2^Institute for Medical Immunology, Charité—Universitätsmedizin Berlin, Berlin, Germany; ^3^Department of Pathology, University of Veterinary Medicine, Hanover, Germany

This study was conducted to characterize T cell Immune response cDNA 7 (TIRC7) expression in healthy dogs and dogs suffering from intervertebral disc herniation (IVDH) or idiopathic epilepsy (IE). An inflammatory reaction in these diseases might contribute to disease progression. TIRC7 is a transmembrane receptor expressed in subsets of activated human and rodent lymphocytes and contributes to the regulation of lymphocyte activation through various pathways. TIRC7 expression gained great interest, since anti‐TIRC7 antibodies selectively modulate immune responses or monitor the clinical course of immune‐related diseases. To elucidate the expression of TIRC7 in dogs suffering from IVHD and IE, we analyzed peripheral blood mononuclear cells (PBMC) of 8 healthy beagles, 26 dogs with IVDH, and 20 dogs with IE using multicolor flow cytometry. Intracellular and extracellular staining with a specific anti‐TIRC7 antibody enabled the characterization of receptor expression. TIRC7 was expressed on lymphocytes and monocytes. Patients suffering from IVDH (range 135–1681, median 567) and IE (range 121–1458, median 385) showed elevated TIRC7 mean fluorescence intensity (MFI) levels compared with healthy beagles (range 127–534, median 330). Dogs with acute seizures (0–1 days: median 816) showed higher TIRC7 expression compared with patients with a longer duration between seizures and blood sampling (> 1 days: median 348). TIRC7 might serve as a novel activation marker in canine PBMC. In comparison to inflammatory disease, induction of TIRC7 expression might be used as an early biomarker in primarily non‐inflammatory neurological diseases such as IE or IVDH, particularly in acute cases and seizures.

## 11 (OP 10)

### 11.1 High Field MRI Findings in Epileptic Dogs With A Normal Inter‐Ictal Neurological Examination

#### 11.1.1 S. Phillipps, BA, BVetMed, DipECVN, AFHEA, MRCVS,^1^ R. Goncalves, DVM, MVM, DipECVN, FHEA, MRCVS^1^

##### 11.1.1.1 ^1^ Institute of Infection, Veterinary and Ecological Sciences, University of Liverpool, Liverpool, UK

Epilepsy is one of the most common neurological conditions affecting dogs. Previous research exploring the likelihood of a structural cause of epilepsy in dogs with a normal inter‐ictal examination is limited to a small population of dogs using low‐field MRI. The aims of this study were to establish high‐field MRI findings in dogs presenting with seizures and a normal inter‐ictal examination. Medical records were retrospectively searched for dogs presenting with at least two seizure events more than 24 h apart. To be included in the study, patients had to have a normal neurological examination, high‐field MRI of the brain and have had metabolic and toxic causes of seizures excluded. Four hundred and two dogs were eligible for inclusion. Crossbreeds were the most commonly affected breed (*n* = 62, 15.4%) followed by border collies (*n* = 38, 9.5%) and Labrador retrievers (*n* = 25, 6.2%). Seventy‐five dogs (18.75%) had abnormalities detected on MRI, 60 (80.0%) of which were considered to be incidental. Overall, 15 dogs (3.7%) had a structural cause of their seizures including neoplasia (*n* = 12), anomalous (*n* = 2), and meningoencephalitis of unknown origin (MUO) (n = 1). When split into age groups at diagnosis, structural lesions were documented in: 0/29 dogs aged < 1 year, 4/247 dogs aged 1 year = 6 years (1.6%, three neoplastic and one anomalous), 3/65 aged > 6 years = 8 years (4.6%, two neoplastic and one MUO), and 8/51 dogs aged > 8 years (15.7%, seven neoplastic and one anomalous). Structural lesions are an uncommon cause of epilepsy at any age in dogs with a normal inter‐ictal examination.

## 12 (OP 11)

### 12.1 Circulating MicroRNAs as Potential Diagnostic Biomarkers in Canine Idiopathic Epilepsy and Drug‐Resistant Epilepsy

#### 12.1.1 M. Pasierbinska, DVM MRCVS,^1^ M. Perez, PhD,^1^ C. Stalin, MA VetMB DipECVN MRCVS,^1^ A. Kaczmarska, DVM MRCVS,^1^ A. Cloquell‐Miro, DVM PGCert MRCVS,^1^ P. Paul Capewell, PhD,^1^ W. Weir, PhD,^1^ C. Britton, PhD,^1^ R. Gutierrez‐Quintana, MVZ MVM DipECVN MRCVS^1^

##### 12.1.1.1 ^1^ School of Biodiversity, One Health and Veterinary Medicine, College of Medical, Veterinary and Life Sciences, University of Glasgow, Glasgow, UK

MicroRNAs (miRNAs) are small non‐coding RNAs that regulate post‐transcriptional gene expression. Circulating miRNAs might represent a form of intercellular communication, as they can act as signaling molecules. MiRNAs are differentially expressed in the brain under pathological conditions and might therefore represent both therapeutic targets and diagnostic or prognostic biomarkers for neurological diseases, including epilepsy. Using miRNA expression profiling, we analyzed blood circulating miRNA in drug‐naive dogs diagnosed with idiopathic epilepsy, as well as dogs responding to treatment, refractory cases, and normal healthy individuals. Plasma samples from seven healthy dogs and 14 dogs with a tier II confidence level for idiopathic epilepsy were collected. Among epileptic dogs seven were drug‐naive and seven on antiseizure drugs (ASD) of which three were drug‐resistant (had < 50% reduction of seizures despite at least two ASD). Differentially expressed miRNAs were identified and their specificity, sensitivity, and expression levels were used to identify the most promising biomarkers. Analyzed data revealed differences between groups, including both down and upregulation of specific miRNA genes. Certain miRNAs were identified (e.g., miR‐381, miR‐214, and miR‐224 miRNA) that distinguish dogs with epilepsy versus control/healthy dogs, and miRNAs that distinguish resistant versus responders within the epileptic group (e.g., miR‐223, miR‐129, and miR‐210 miRNA). The results of this study indicate a potential role of miRNAs in the underlying molecular mechanisms of idiopathic and drug‐resistant epilepsy in dogs, highlighting their use as potential biomarkers and establishing the basis for further studies.

## 13 (OP 12)

### 13.1 Towards a Better Characterization of Spontaneous Canine Cerebrovascular Accidents: A Prospective Pilot Study

#### 13.1.1 C. Escriou, DVM, PhD,^1^ C. Pouzot, DVM, PhD, Dip ECVECC,^2^ E. Segard, DVM, Dip ECVDI,^3^ F. Dimeglio, Phys,^4^ S. Charaoui, PhD,^5^ H. Dorez, PhD,^4^ E. Canet‐Soulas, DVM, PhD^6^

##### 13.1.1.1 ^1^Unité de Neurologie, CHUVAC VetAgro Sup, Marcy l'Etoile, France; ^2^Siamu, CHUVAC VetAgro Sup, Marcy l'Etoile, France; ^3^Service d'imagerie, CHUVAC VetAgro Sup, Marcy l'Etoile, France; ^4^Hawkcell, Marcy l'Etoile, France; ^5^ CICV VetAgro Sup, Marcy l'Etoile, France; ^6^ U1060 INSERM CarMeN Université Lyon 1, Lyon, France

Although the occurrence of spontaneous cerebrovascular accidents (CVA) is well established in dogs, their characterization by the mean of advanced MRI remains fragmented. Most published case series are retrospective with no to poor clinical and imaging follow‐up leading to difficulties in giving “evidence‐based” recovery prognosis. The aim of our prospective clinical study was to characterize and follow‐up MRI aspect of CVA with all sequences currently used to characterize human stroke and to correlate with clinical and neurological data. Ten dogs were included in a one‐year study. Inclusion criteria were peracute neurological signs with no evolution after 24 h and MRI brain imaging consistent with CVA. Serial MRI imaging (T0, T0 + 7, and 30 days) was performed with an MRI vascular dedicated protocol including (T2, 3D T1, 3D Flair, 3D Swan, DWI, 3D Flair + contrast, bolus tracking perfusion imaging, and angio‐MRI [Time of Flight]). A complete check‐up (hematology, coagulation state with ROTEM, blood biochemistry, urine and CSF analysis, echocardiography, and abdominal echography) was performed on all dogs to detect stroke predisposing factors. Our vascular protocol allowed to identify, characterize, and follow CVA in a consistent manner with the delay of neurological signs occurrence (4 dogs < 24 h, 24 h < 3 dogs < 48 h, and 3 dogs > 72 h) whatever their localization (forebrain 3, cerebellum 4, and brain stem 3). Notably, perfusion imaging allowed to discriminate ischemic penumbra from a necrotic central core and to identify mismatch as described in humans. Exactly 8/10 dogs recover within 15–30 days in correlation with MR imaging evolution. The hypercoagulable state was identified for all dogs, the origin of which was identified for 9/10 dogs. Further studies are needed to better correlate the canine CVA recovery prognosis with this advanced MR imaging.

## 14 (OP 13)

### 14.1 Arthroscope‐Assisted Foraminotomy of the Lumbosacral Neuroforamen in Dogs: Establishment of Surgical Technique

#### 14.1.1 D. Bekiaridis, DVM,^1^ A. Pozzi, DVM, MSc, DECVS, DACVS, DACVSMR,^1^ J. Guevar, DVM, DECVN, MRCVS,^2^ L. Smolders, DVM, PhD, DECVS^1^

##### 14.1.1.1 ^1^Clinic for Small Animal Surgery, Vetsuisse‐Faculty, University of Zurich, Zurich, Switzerland; ^2^Clinic for Small Animal Neurology, Vetsuisse‐Faculty, University of Bern, Bern, Switzerland

Introduction/Purpose: The purpose of this pilot study was to establish a surgical technique for arthroscope‐assisted dorsolateral foraminotomy (AADF) of L7–S1 in dogs and to macroscopically assess the effectiveness and safety of AADF.

Methods: Four canine cadavers (BW: 30–40 kg) euthanized due to reasons unrelated to spinal disease were used (owner consent was obtained for all cadaveric specimens). Bilateral AADF of L7–S1 was performed in all specimens (8 procedures), evaluating different arthroscopes, arthroscopic instruments, and scope/instrument portals. The surgical technique was optimized based on working ergonomics (triangulation), visualization and preservation of the nerve root, and effectiveness of decompressing the neuroforamen.

Results: A 2 cm dorsal keyhole approach to the L7 transverse process was established to confirm the correct surgical location and to optimize arthroscopic visualization. AADF was feasible using both a 0° Nanoscope and a 30° 1.9 mm arthroscope. Optimal scope/instrument portal configuration was defined as: (1) dorsolateral to ventromedial scope port dorsally crossing the iliac wing, allowing continuous visualization of the L7 nerve root and neuroforamen, (2) a dorsal instrument portal through the keyhole approach, and (3) a caudal instrument portal dorsally crossing the sacroiliac joint in a caudocranial direction. Macroscopic evaluation showed effective enlargement of the lumbosacral neuroforamen without macroscopic damage to the L7 nerve root.

Discussion/Conclusion: AADF is a feasible technique that provides enhanced visualization and thereby safety for decompressing the L7 nerve root in dogs. At the time of writing this abstract, the accuracy, effectiveness, and safety of AADF is further evaluated and compared with conventional dorsolateral foraminotomy in a larger sample size using computer tomography and histopathology.

## 15 (OP 14)

### 15.1 Recurrence of Cervical Intervertebral Disc Extrusion in 55 Small Breed Dogs After Surgical Decompression With or Without Prophylactic Fenestration (2015–2021)

#### 15.1.1 C. Berthomé, DMV, IPSAV,^1^ A. Castel, DMV, MSc, DACVIM (Neurology),^2^ D. Gagnon, DVM, DVSc, DACVS‐SA,^3^ R. Malenfant, DMV, DACVS‐SA^4^

##### 15.1.1.1 ^1^Neurology Service, Centre Vétérinaire DMV, Montréal, Canada; ^2^Neurology Service, Department of Clinical Sciences, Faculté de médecine vétérinaire, Université de Montréal, Saint‐Hyacinthe, Canada; ^3^Surgery Service, Department of Clinical Sciences, Faculté de médecine vétérinaire, Université de Montréal, Saint‐Hyacinthe, Canada; ^4^Surgery Service, Centre vétérinaire Daubigny, Québec, Canada

Introduction: Prophylactic fenestration (PF) of adjacent intervertebral disc spaces during decompression surgery for thoracolumbar intervertebral disc extrusion in dogs is recommended to prevent recurrence. The objectives of this study were to determine whether concomitant PF of adjacent interverbal disc spaces decreases the recurrence rate (RR) of cervical intervertebral disc extrusion (C‐IVDE) in small breed dogs treated by ventral slot (VS) decompression, and whether this procedure is associated with an increased risk of surgical complications.

Method: Medical records of dogs that underwent VS decompression for C‐IVDE between 2015 and 2021 with a follow‐up of at least 1‐year were reviewed retrospectively in this multicenter study. The RR and complication rates were compared for dogs with and without PF using likelihood ratio tests. Surgical time and neurological grade before, after VS decompression, and at first recheck were also compared using Mann–Whitney tests.

Results: Fifty‐five small breed dogs were included (PF group: *n* = 18 vs. non‐PF group: *n* = 37). Neurological grades at all timepoints were similar between groups (*p* = 0.612 and *p* = 0.724, respectively). Recurrence of C‐IVDE occurred in 25% of dogs (14/55), all in the non‐PF group. Prophylactic fenestration was significantly associated with a lower RR (*p* < 0.001). Although surgery (mean surgical time ± standard deviation) was significantly longer in the PF group (158.0 ± 13.5 min) versus the non‐PF group (118.0 ± 6.8 min, *p* = 0.017), the complication rate was similar (*p* = 0.838).

Discussion/Conclusion: Prophylactic fenestration appears protective against the recurrence of C‐IVDE in small‐breed dogs and was not associated with a higher risk of complications in this study.

## 16 (OP 15)

### 16.1 Comparison of Three Surgical Techniques Used in the Management of Congenital Thoracic Vertebral Body Malformations in 22 Dogs Across Two Referral Centers and Their Complications and Outcome

#### 16.1.1 S. Riziq Picón, DVM MRCVS,^1^ G. Bruto Cherubini, DVM DipECVN FRCVS,^2,^ ^3^ L. Motta, DVM DipECVN MRCVS^1^

##### 16.1.1.1 ^1^Northwest Veterinary Specialist, Runcorn, UK; ^2^Dick White Referrals, Cambridgeshire, UK; ^3^University of Pisa, Pisa, Italy

To compare three surgical techniques to treat congenital thoracic vertebral body malformations (CTVBM) in 22 brachycephalic dogs, and retrospectively describe the perioperative complications and outcome. The most common clinical presentation was acute‐progressive or chronic ambulatory paraparesis with spinal ataxia. The median age at presentation was 21.6 months. The most commonly affected vertebrae were between T6 and T9. Transthoracic vertebral distraction and stabilization (TVDS), positively threaded‐profile‐pins and polymethylmethacrylate with laminectomy (PTPP‐PMMA), and dorsal laminectomy (DL), were performed in 5/22, 6/22, and 11/22 dogs, respectively. No life‐threatening intraoperative complications were reported. The mean follow‐up was 426 days (547 for TVDS group, 180 for PTPP‐PMMA group, and 402 for DL group). At presentation, all TVDS dogs were modified Frankel scale (MFS) Grade 4. The immediate postoperative neurological MFS did not change but at follow‐up all dogs became neurologically normal. Immediate postoperative deterioration was present in 5/6 dogs in the PTPP‐PMMA group. All dogs were Grade 4 at presentation postoperatively 2/6 were Grade 3a, 1/6 Grade 3b, and 2/6 Grade 2. Long‐term, 5/6 dogs remained Grade 4 and 1/6 Grade 6. In the DL group at presentation, the neurological grade was 4 (7/11), 3a (3/11), and 1 (1/11). Post‐operative neurological deterioration occurred in 9/11 dogs, 1/11 became neurologically normal, and 1/11 improved. Five dogs that initially deteriorated, improved at follow‐up, but neurological signs persisted (Grade 4 2/5, Grade 3a 3/5). Four dogs suffered deterioration and were euthanized 1–22 months post‐operatively. This study identified a positive influence on long‐term outcomes when vertebral stabilization techniques were used to treat CTVBM, especially TVDS.

## 17 (OP 16)

### 17.1 Is Prophylactic Fenestration in the Cervical Vertebral Column Necessary? Confirmed Recurrence of Cervical Intervertebral Disc Extrusions Adjacent to Previous Ventral Slots in Dogs

#### 17.1.1 E. Mahon, BVM, BVS, MRCVS,^1^ O. Marsh, BVM, BVS, MRCVS,^2^ F. Stabile, DVM (Hons), PhD, DipECVN, MRCVS^1^

##### 17.1.1.1 ^1^The Neurology and Neurosurgery Department, Southfields Veterinary Specialists, Basildon, UK; ^2^The Neurology and Neurosurgery Department, Dick White Referrals, Newmarket, UK

Intervertebral disc (IVD) prophylactic fenestration in chondrodystrophic dog breeds following a thoracolumbar intervertebral disc extrusion (IVDE) decreases the risk of IVDE recurrence. The benefit of cervical prophylactic IVD fenestration in dogs is unknown. Recurrence of cervical myelopathy has been reported in 33% of dogs who underwent a previous ventral slot (VS). The aim of this study was to assess the recurrence rate of cervical IVDEs requiring a second VS in dogs. The medical records of 59 dogs who had a VS due to IVDE in a single institution between January 2020 and March 2023 were retrospectively examined. No dogs had prophylactic IVD fenestration of IVDs adjacent to the VS. French bulldogs (25/59) and dachshunds (9/59) were overrepresented. The most frequent site of IVDE was C3–C4 (21/59), then C4–C5 (16/59), C2–C3 (15/59), C5–C6 (6/59), and C6–C7 (1/59). Three dogs (5%) experienced the recurrence of clinical signs suggestive of cervical myelopathy at 7, 10, and 18 months, respectively, after the first VS. In all three cases repeat MRI confirmed a second cervical IVDE requiring VS. The second IVDE was adjacent to the first VS site in two dogs. In one dog, the second IVDE was two IVD spaces caudal from the first VS site. All dogs recovered completely after both surgeries. In our cohort, confirmed recurrence of a cervical IVDE requiring a second VS was lower (5%, 3/59) compared with previously reported confirmed recurrence (19%, 16/83), supporting that routine prophylactic IVD fenestration in the cervical region might not be necessary.

## 18 (OP 17)

### 18.1 Application of Diffusion Tensor Tractography in Traumatic Spinal Cord Injuries in Small Animals

#### 18.1.1 T. Heredia, LV,^1^ J. González, LV,^2^ S. Ortiz, LV,^2^ S. Ródenas, DipECVN^3^

##### 18.1.1.1 ^1^ AniCura Ars Veterinaria, Barcelona, Spain; ^2^ AniCura Valencia Sur, Valencia, Spain; ^3^Hospital Bluecare Partners, Málaga, Spain

Traumatic spinal cord injuries (TSCI) are common in small animals and can irreversibly compromise patient's neurological status. Magnetic resonance imaging (MRI) is the test of choice for the assessment of parenchymal TSCI but it is limited for functional prediction in patients with acute TSCI. Application of diffusion tensor tractography (DTT) is required to evaluate the spinal fibers state. The aim of this article is to describe DTT role in seven dogs and one cat with TSCI. Patients were evaluated neurologically for TSCI due to different events. MRI (T2W, FLAIR, T2*, T1W pre‐ and post‐contrast sequences) and DTT were performed, three in cervical region and five in thoracolumbar region. DTT revealed fiber displacement in one case and a lack of continuity of spinal cord fibers in 7/8 cases. Hemorrhage was correlated with fiber discontinuity. The intramedullary hyperintensity detected in T2W and STIR was not associated in all cases with fiber discontinuity, which allowed to differentiate cases with suspected edema or gliosis from those with suspected necrosis. In one case with fiber disruption, follow‐up MRI was performed 6 months later showing changes in T1W intensity, while DTT maintained the same aspect, demonstrating its early veracity. DTT is a non‐invasive technique that allows to determine the prognosis for patients with early‐stage TSCI. To the author's knowledge, this is the first publication on the practical application of DTT in small animals with TSCI in early‐stage and chronic cases. Further studies are needed to support its application.

## 19 (OP 18)

### 19.1 Susceptibility‐Weighted Imaging for Identification and Follow‐Up of Cerebral Microbleeds After Brain Tumor Irradiation in Dogs

#### 19.1.1 C. Staudinger, Dr. Med. Vet,^1^ M. Dennler, Dr. Med. Vet., Dipl ECVDI,^2^ M. Körner, Dr. Med. Vet., DACVR (Radiation Oncology),^1^ V. Meier, Dr. Med. Vet., DACVR (RO), DipECVDI (Add Rad Onkol),^1^ K. Beckmann, Dr. Med. Vet., Dipl ECVN,^3^ C. Rohrer Bley, Prof. Dr. Med. vet., DACVR (RO), DipECVDI (Add Rad Onkol)^1^

##### 19.1.1.1 ^1^Division of Radiation Oncology, Vetsuisse Faculty, University of Zurich, Zurich, Switzerland; ^2^Clinic for Diagnostic Imaging, Department of Clinical Diagnostics and Services, Zurich, Switzerland; ^3^Division of Neurology, Vetsuisse Faculty, University of Zurich, Zurich, Switzerland

Introduction: Spontaneous vocalization has been anecdotally associated with cervical disc disease and more robustly associated with Chiari‐like malformation and syringomyelia. This study describes the signalment, localization, and diagnoses in a cohort of dogs presenting with spontaneous vocalization.

Introduction/Purpose: Advanced MRI techniques represent the current standard of care for monitoring treatment response after radiotherapy. Vascular injury, a possible side effect after radiotherapy can result in cerebral microbleeds (CMB) detectable with susceptibility‐weighted imaging (SWI), a MR‐sequence sensitive to the hemosiderin‐caused iron deposits. In humans, microbleeds seem to occur preferentially in the high‐dose area, increase over time, and are clinically associated with neurodegenerative decline. The aim of the study was to identify and quantify microbleeds at different dose levels and time points in dogs irradiated for brain tumors.

Methods: Retrospective, exploratory study. Dogs previously irradiated for brain tumors with a baseline and one follow‐up or at least two follow‐up scans with a SWI sequence were included. Extra‐tumoral signal voids consistent with CMB were identified and counted as total number as well as densities in 3 discrete peritumoral volumes: low (< 20 Gy), medium (20–30 Gy), or high (> 30 Gy) dose. A linear mixed‐effects model was applied to the data.

Results: Thirty dogs with in total 72 MRI‐scans were included. In 18 dogs (60%; 95% CI: 42%–76%), signal voids consistent with CMB were detected. The majority of absolute CMB as well as the highest CMB‐density was found in the high‐dose volume: at any given time‐point, dogs had 40% (95% CI: 8%–80%, *p* < 0.001) more CMB in the high‐ compared with the low‐dose volume. With each month after treatment, the count increased by 0.09 (95% CI: 0.07–0.11, *p* < 0.001).

Discussion/Conclusion: Vascular injury identifiable as cerebral microbleeds can be found in irradiated brain tissue of dogs is dose‐dependent and progressive over time.

## 20 (OP 19)

### 20.1 Cerebellar Degeneration and Myositis Complex in 4 Nova Scotia Duck Tolling Retriever Puppies of 2 Distant Litters: Clinical Presentation and Diagnostic Evaluation

#### 20.1.1 Q. van Koulil, Resident ECVN, DVM,^1^ I. van Soens, Dipl. ECVN Diplomat, DVM, PhD,^1^ ^,2^ S. Bhatti, DVM, PhD,^3^ S. Rupp, DVM, PhD,^4^ M. Beukers, Dipl. ECVDI, DVM, PhD,^1^ K. Matiasek, Dipl. ECVN, DVM, PhD^5^

##### 20.1.1.1 ^1^Department of Neurology, Dierenziekenhuis Hart van Brabant in Waalwijk, Waalwijk, The Netherlands; ^2^Companion Animal Department, Faculty of Veterinary Medicine Liege University, Liege, Belgium; ^3^Small Animal Department, Faculty of Veterinary Medicine Ghent University, Ghent, Belgium; ^4^Neurology Department, Tierklinik Hofheim, Hofheim, Germany; ^5^Section of Clinical and Comparative Neuropathology, Centre for Clinical Veterinary Medicine Ludwig‐Maximilians University Munchen, Munchen, Germany

An SLC25A12 Missense Variant in Nova Scotia Duck Tolling Retrievers (NSDTR) has been shown to cause signs of cerebellar ataxia with/without neuromuscular weakness. The aim of this case report is to detail the clinical presentation, results of diagnostic testing, and follow‐up of the 4 NSDTR from 2 related litters that were included in previous publications. Clinical signs appeared between 8 and 15 weeks of age, consisting of episodes of generalized ataxia, and intentional tremors, and 2 dogs experienced episodes of generalized muscle spasms/weakness. These progressed to ambulatory tetraparesis with generalized muscle atrophy. CPK was measured on several occasions, and increases up to 20‐fold of the upper limit were found. MR of the brain in two dogs showed bilateral symmetrical lesions in the cerebellar nuclei (hyperintense on T2W, hypointense on T1W). PCR analysis on CSF and serology for infectious agents in 3 dogs were within normal limits. EMG performed in 1 dog showed mild spontaneous activity in the appendicular muscles. Muscle and nerve biopsies performed in this dog revealed lymphohistiocytic myositis without evidence of intracellular infectious agents on histology and tissue PCR. Postmortem evaluation of 1 dog uncovered severe cerebellar nuclei degeneration. Two dogs were euthanized due to progressive signs of neuromuscular weakness and/or ataxia and 2 other dogs are still alive. SLC25A12 Missense Variant in NSDTR causes progressive cerebellar signs with/without neuromuscular signs caused by myositis. This pathology was named Cerebellar Degeneration—Myositis Complex (CDMC). The prognosis is, however, guarded due to the progressive nature and minimal response to supportive treatment.

## 21 (OP 20)

### 21.1 Steroid Monotherapy Versus Combined Cytarabine Continuous Rate Infusion and Steroid Therapy in Dogs With MUO: A Blinded, Randomized Controlled Trial

#### 21.1.1 B. Jones, BScVetSci MSc BVetMed PGDip DipECVN MRCVS,^1^ F. Liebel, DVM Dip. ECVN MRCVS,^1^ A. Fadda, DVM DrMedVet Dip ECVN PhD MRCVS,^1^ S. Martin, BVSc PGDip VCP MSc DipECVN MRCVS,^1^ R. Lawn, BVSc CertAVP (SAM) MRCVS,^1^ K. Lazzerini, DrMedVet MSc DipECVN,^1^ T. Harcourt‐Brown, MA VetMB CertVDI DipECVN CertVDI FRCVS^1^

##### 21.1.1.1 ^1^Langford Vets Small Animal Hospital, Bristol BS40 5DU, UK

Aims: To compare the short‐term outcome in dogs with meningoencephalitis of unknown origin (MUO) treated with corticosteroids alone or corticosteroids combined with cytosine arabinoside (CA).

Methods: Parallel, blinded, randomized controlled trial. Dogs with MRI and/or CSF features compatible with MUO were randomized to steroid‐only (4 mg/kg/day for 2 days followed by 2 mg/kg/day prednisolone or dexamethasone equivalent) or CA CRI and steroid (200 mg/m^2^ over 8 h combined with 2 mg/kg/day prednisolone) treatment groups. Assessors were blinded to the protocol using reversible redaction of clinical records and dogs were considered to have failed treatment if their neurological score increased or they died. Using intention‐to‐treat analysis, the primary objective of proportions failing treatment at 7 days was compared between groups using Fisher's‐exact test. Secondary objectives of treatment failure at 30‐ and 100‐days compared using Fisher's‐exact test and all‐cause mortality at 100 days using Log rank analysis of Kaplan–Meier survival curves.

Results: 35 dogs steroid‐only and 33 dogs combined CA and steroid. In the steroid‐only group, proportions failing treatment at 7‐, 30‐, and 100‐days were 7/35 (20%), 9/35 (26%), and 15/35 (43%). These proportions were no different to the steroid and CA CRI group: 7/33 (21%), 11/33 (33%), and 22/33 (67%). All‐cause mortality at 100 days was not significantly different between treatment groups (*p* = 0.62).

Conclusions: The addition of CA CRI to an immune‐suppressive dose of corticosteroids at the time of MUO diagnosis does not reduce the risk of deterioration at 7 days or improve survival at 30‐ or 100‐days compared with initial treatment with corticosteroids alone.

## 22 (OP 21)

### 22.1 Facial Myokymia in Six Dogs and Two Cats

#### 22.1.1 T. Elvira, Neurology Resident, LV GPCert (Neuro) MRCVS, A. Crawford, BVM&S BSc PhD DipECVN MRCVS,^2^ A. Fraser, BVSc (Hons) MVetStud MVSc DipECVN MRCVS,^3^ L. Mari, DVM DipECVN MRCVS,^4^ E. Ives, MA VetMB DipECVN MRCVS,^1^ A. Tauro, DVM GPCert (Neuro) DipECVN MRCVS,^5^ V. Indzhova, Neurology Resident, MVDr MRCVS,^6^ B. Lopes, DVM GPCert (Neuro) Clinician in Veterinary Neurology MRCVS,^7^ G. Corsini, DVM Clinician in Veterinary Neurology MRCVS,^1^ J. Brocal, Ldo Vet DipECVN MRCVS^1^

##### 22.1.1.1 ^1^Anderson Moores Veterinary Specialists, Hursley, Winchester, UK; ^2^Queen Mother Hospital for Small Animals (RVC), London, UK; ^3^Davies Veterinary Specialists, Hitchin, UK; ^4^The Ralph Veterinary Referral Centre, Marlow, UK; ^5^ ChesterGates Veterinary Specialists, Chester, UK; ^6^Willows Veterinary Centre and Referral Service, Birmingham, UK; ^7^Southfields Veterinary Specialists, Basildon, UK

Myokymia is a form of peripheral nerve hyperexcitability, which can be focal or generalized. There is limited information regarding focal myokymia (FM) in veterinary medicine. The purpose of this retrospective study was to describe the clinical presentation, diagnoses, treatment, and outcome in dogs and cats with focal FM. The clinical data of cases presenting with persistent worm‐like rippling movements of the facial muscles compatible with FM was collected. Six dogs and two cats were identified. Investigations included brain MRI (*n* = 7), electromyography (*n* = 5), and CSF analysis (*n* = 4). Electromyography confirmed myokymic discharges in all five cases. For the six dogs, the final diagnoses were facial and vestibular neuropathy of unknown origin (FVNUO) (*n* = 3), meningoencephalitis of unknown origin (*n* = 1), otitis media/interna with intracranial extension (*n* = 1), and otitis interna, facial neuritis and pituitary mass (*n* = 1). An infiltrative adenosquamous carcinoma with intracranial extension (*n* = 1) and orofacial trauma (*n* = 1) were diagnosed in the two cats. For the dogs with FVNUO, contrast enhancement of the facial (*n* = 3) and/or vestibulocochlear (*n* = 2) nerves was seen on MRI. Treatments differed depending on the diagnosis. All dogs with FVNUO were treated with prednisolone, and myokymia partially improved or completely resolved in these cases. Three patients were euthanized within 8 months of diagnosis. Five patients are still alive, 1–28 months after diagnosis. FM is a rare presentation in dogs and cats, with a range of structural disorders affecting the facial motor nucleus/nerve identified. Treatment and outcome depend on the underlying cause but outcomes can be good in dogs with FVNUO.

## 23 (OP 22)

### 23.1 Prospective Randomized Trial Comparing Relapse Rates in Steroid Responsive Meningitis‐Arteritis Dogs Treated With Either a 6‐Week or 6‐Month Oral Prednisolone Protocol

#### 23.1.1 J. Rose, MA, VetMB, DipECVN, MRCVS, C. Driver, BSc, BVetMed (Hons), MVetMed, PhD, DipECVN ^11^ FRCVS, L. Arrol, MA, VetMB, DipECVN, MRCVS, T. Cardy, BSc, BVetMed (Hons), MVetMed, PhD, DipECVN, MRCVS,^2^ J. Tabanez, DVM, MRCVS,^3^ A. Tauro, DVM, GPCert (Neuro), DipECVN, MRCVS,^4^ R. Fernandes, DVM, DipECVN, MRCVS,^5^ I. Schofield, BVSc, MSc (VetEpi), PhD, MRCVS,^6^ S. Adamantos, BVSc, CertVA, DACVECC, DipECVECC, MRCVS,^5^ N. Granger, DVM, PhD, DipECVN, FRCVS,^7^ T. Harcourt‐Brown, MA, VetMB, CertVDI, DipECVN, CertVDI, FRCVS^8^

##### 23.1.1.1 ^1^Lumbry Park Veterinary Specialists, Alton, UK; ^2^Cave Veterinary Specialists, Wellington, UK; ^3^Fitzpatrick Referrals, Godalming, UK; ^4^College of Veterinary Medicine, North Carolina State University, Raleigh, North Carolina, USA; ^5^Paragon Referrals, Wakefield, UK; ^6^ CVS, Diss, Norfolk, UK; ^7^Bristol Vet Specialists, Bristol, UK; ^8^Langford Vets, University of Bristol, Bristol, UK

Steroid‐responsive meningitis‐arteritis (SRMA) is a systemic inflammatory disease of dogs that particularly affects the leptomeninges and is usually treated with immunosuppression. Long courses of prednisolone have traditionally been used but this is associated with corticosteroid‐related adverse effects that impact quality of life. We aimed to compare the resolution of clinical signs and rate of relapse of SRMA with a “long” 6‐month prednisolone protocol and a “short” 6‐week protocol. Multi‐center, prospective, with allocation concealment, randomized trial with 12‐month follow‐up. The same prednisolone protocol restarted if relapse occurred. Analysis with logistic and Poisson regression modeling. A total of 20/44 cases were allocated to the 6‐month protocol and 24/44 to the 6‐week protocol. All cases responded to treatment on telephone follow‐up day 14. Relapses occurred in 6/20 (30.0%) of the 6‐month protocol and 9/24 (37.5%) of the 6‐week protocol. There was no statistical difference in the incidence risk of at least one relapse between the two groups (Odds ratio = 1.40, 95% CI: 0.40–4.96, *p* = 0.602). Between the 15 dogs with relapses, 10/15 (66.7%) relapsed once, 3/15 (20.0%) relapsed twice, and 2/15 (13.3%) relapsed three times (22 total relapses). No statistical difference in the incidence rate ratio (1.46; 95% CI: 0.61–3.48; *p* = 0.395) of relapse events between the two groups was observed. SRMA cases responded to both prednisolone protocols and there was no significant difference in relapse rates between the 6‐month and 6‐week courses. “Short” prednisolone protocols could be used for SRMA, thereby reducing the duration and severity of adverse corticosteroid side effects.

## 24 (OP 23)

### 24.1 Comparison of Non‐Invasive Intracranial Pressure Monitoring and High‐Field Magnetic Resonance Imaging for Presuming Intracranial Hypertension in Dogs

#### 24.1.1 I. S. Santos, DVM, MSc,^1^ M. V. Bahr Arias, DVM, PhD,^2^ A. G. Adeodato, DVM, PhD,^3^ T. F. Guimarães, DVM,^3^ M. C. Martins, DVM,^3^ R. A. F. Barros, DVM,^3^ O. F. de Brito, DVM,^3^ R. S. G. Dias, DVM, MSc,^3^ J. L. R. Marques, DVM, MSc^3^

##### 24.1.1.1 ^1^Postgraduate Program in Animal Health Science, Londrina State University, Londrina, Brazil; ^2^Professor, Department of Veterinary Clinical Sciences, Londrina State University, Londrina, Brazil; ^3^ CRV Imagem Centro de Referência Veterinária, Rio de Janeiro, Brazil

Due to the limitations of invasive monitoring intracranial pressure (ICP) methods, several noninvasive techniques are being investigated. This study aimed to compare high‐field magnetic resonance imaging (MRI) with noninvasive monitoring of ICP waves (ICP‐Ni) to indirectly assess ICP in 43 dogs with intracranial disorders. ICP‐NI was conducted before the scans. Dogs with P2/P1 waves ratio > 0.8 were classified as presumed intracranial hypertension (ICH). MRI findings were used to further classify dogs in a presumed ICH group. The optic nerve sheath diameter/weight (ONSD/weight) ratio was also analyzed. Twenty‐nine dogs were classified as having ICH based on ICP‐Ni, with a median P2/P1 ratio of 1.02 [0.93–1.23]. The normal ICP group (14 dogs) had a median P2/P1 ratio of 0.65 [0.54–0.75] (*p* < 0.001). Dogs with presumed ICH showed a larger ONSD ratio (left: 0.41 cm [0.24–0.63], right: 0.44 cm [0.25–0.69]). Dogs with presumed normal ICP had a smaller ONSD ratio (left: 0.19 cm [0.12–0.31], right: 0.18 cm [0.13–0.31]) (*p* < 0.05). When classified based on MRI findings, 19 dogs were identified with ICH, with a median P2/P1 ratio of 0.97 [0.88–1.11]. Dogs classified as having normal ICP (24 dogs) had a median P2/P1 ratio of 0.87 [0.64–1.12] (*p* = 0.322). Dogs with presumed ICH through MRI showed a larger ONSD ratio (left: 0.39 cm [0.23–0.64], right: 0.42 cm [0.22–0.61]), while dogs with presumed normal ICP had a smaller ONSD ratio (left: 0.28 cm [0.15–0.43], right: 0.28 cm [0.16–0.51]). However, there was no statistically significant difference (*p* > 0.05). In conclusion, noninvasive ICP waves monitoring and MRI analysis provide valuable information for assessing intracranial hypertension in dogs. However, the significance of P2/P1 ratio and ONSD ratio may vary depending on the classification method. Further research is needed to establish more accurate diagnostic criteria.

## 25 (OP 24)

### 25.1 Quantitative Proteomics of Cerebrospinal Fluid Reveals Potential Protein Biomarkers for Canine Non‐Infectious Meningoencephalomyelitis

#### 25.1.1 M. Aradillas Pérez, DVM,^1^ E. M. Espinosa López, MSc,^2^ A. Seisdedos Benzal, DVM, MSc, PhD,^1^ G. Gómez Baena, MSc, PhD,^2^ A. Galán Rodríguez, DVM, MSc, PhD^1^

##### 25.1.1.1 ^1^Department of Small Animal Medicine and Surgery, University of Cordoba, Cordoba, Spain; ^2^Department of Biochemistry and Molecular Biology, University of Cordoba, Cordoba, Spain

Canine non‐infectious meningoencephalomyelitis (NIME) encompasses a group of idiopathic, most likely immune‐mediated, inflammatory diseases of the central nervous system (CNS). Currently, accurate diagnosis of NIME represents a major challenge in veterinary medicine. This study is aimed to apply state‐of‐the‐art quantitative proteomic technology to identify a panel of novel biomarkers for NIME. Serum and cerebrospinal fluid (CSF) samples from 30 dogs were included in the study. As control groups, we included patients presenting idiopathic epilepsy (*n* = 11) and intervertebral disc disease (*n* = 13). The study group consisted of dogs suffering from NIME (*n* = 6). Mass spectrometry‐based quantitative proteomics was used to identify changes in protein abundance among the groups in both serum and CSF samples. A set of proteins with significant differential abundance were identified in CSF. Several of the proteins which abundance decreases in NIME have crucial roles in the protection and maintenance of the CNS and have been involved in some human autoimmune and neurodegenerative neurological disorders. The proteins which abundance increases in NIME are known to participate in inflammatory and immune response and their overexpression in CSF may indicate blood–brain barrier disruption. No differences in the serum profiles were observed among the groups, which could be explained due to the isolation of the nerve parenchyma from the systemic vascular bed. In summary, quantitative proteomics of CSF in dogs suffering from NIME allowed the identification of a panel of protein biomarker candidates and provided new insights into the pathogenesis of the disease, suggesting that neuronal dysfunction and immune dysregulation may be involved.

## 26 (OP 25)

### 26.1 A Pilot Trial of Epidural Anesthetic in Dogs Undergoing Lumbar Spine Hemilaminectomy

#### 26.1.1 A. M. K. Chan, BVSc,^1^ L. E. James, BSc, MSc,^1^ S. C. Grasso, DVM, MS, DACVAA^1^

##### 26.1.1.1 ^1^The VSCAN, Ottawa, Ontario, Canada

Epidural anesthesia (EA) is widely used in various canine surgical procedures and lumbar spinal surgeries in humans, but its application in canine hemilaminectomy is unreported. This prospective, assessor‐blinded, randomized pilot trial aims to assess the perioperative analgesic effects and complications of EA in dogs undergoing single or two‐site lumbar spine hemilaminectomy. In this study, four dogs received lumbosacral EA (bupivacaine 0.5%, 0.5 mg/kg, and morphine 0.1%, 0.1 mg/kg), while three dogs received 0.9% saline of the same volume (0.2 mL/kg) as control, in addition to a standardized gaseous and intravenous anesthesia protocol. The need for intraoperative rescue analgesia (fentanyl 2 mcg/kg bolus) was determined based on predefined cardiovascular responses. Intraoperative treatment failure is defined as when a subject requires more than three rescue boluses and is therefore excluded from further analysis. The EA and placebo groups (after excluding one placebo subject) required an average of 1.48 and 2.96 mcg/kg fentanyl, respectively. The EA group demonstrated a reduction in inhalant requirements compared with baseline, while the placebo group showed higher requirements. None of the remaining dogs required postoperative rescue analgesia, apart from standard oral analgesics. Voluntary micturition within 24 h of epidural injection was observed in both groups. Prolonged neural blockade was not evident based on the return of motor function in all dogs in the morning postsurgery. The duration of hospitalization did not differ between the groups. No perioperative complications were noted. This pilot trial suggests that EA may be used as a part of the perioperative multimodal analgesia approach for canine lumbar spinal surgery.

## 27 Poster Presentations

## 28 (P 01)

### 28.1 Acquired Tetraventricular Hydrocephalus Secondary to Obstruction of the Lateral Apertures After Surgical Treatment of Intracranial Empyema in a Dog

#### 28.1.1 K. Santifort, DVM, MSc, Board Eligible ECVN,^1^ ^,2^ E. Glass, DVM, MSc, DACVIM (Neurology)^3^

##### 28.1.1.1 ^1^ IVC Evidensia Small Animal Hospital Arnhem, Arnhem, The Netherlands; ^2^ IVC Evidensia Small Animal Hospital Hart Van Brabant, Waalwijk, The Netherlands; ^3^Section of Neurology and Neurosurgery, Red Bank Veterinary Hospital, Tinton Falls, New Jersey, USA

The fourth ventricle lateral apertures are main site of exit for CSF in the ventricular system of dogs. We report a case of acquired tetraventricular hydrocephalus due to lateral aperture obstruction in a dog. A 4‐month‐old dog was presented with multifocal neurological signs. MRI findings were consistent with intracranial subdural empyema (ICSE) with multiple types of brain herniation and secondary cervical spinal cord edema (no obstructive hydrocephalus or lateral aperture obstruction at this point). A three burr‐hole craniostomy (frontal, dorsal parietal, and ventral parietal bone), dura mater incision, and removal of mucopurulent material by copious lavage and drain placement were performed. Anaerobic culture identified *Bacteroides* spp. and antimicrobial therapy was initiated. The dog experienced multiple seizures postoperatively but was discharged on medical treatment 4 days later with improving mild neurological deficits. The dog remained initially neurologically stable but then deteriorated over the next 10 days. Repeat MRI was consistent with a resolution of ICSE. However, obstructive tetraventricular hydrocephalus and severe cervical spinal cord edema were present now. Medical treatment and follow‐up over the next 8 months yielded improvement and stabilization of neurological signs. Despite the persistence of neurological tetraparesis, ataxia, and seizures, the owner felt the dog's quality of life was good. Acquired, tetraventricular hydrocephalus due to obstruction of the lateral apertures is rarely described and the cause uncertain. The authors postulate that choroid plexitis secondary to intracranial bacterial infection and altered dynamics of CSF flow are the most likely explanations for the findings in this case.

## 29 (P 02)

### 29.1 Ventral Stabilization of Atlantoaxial Subluxation in a Toy Breed Dog With Bi‐Cortical Pedicle Screws and a Patient Specific 3D Printed Titanium Plate

#### 29.1.1 R. Clark, BVetMed (Hons) MRCVS,^1^ J. Elford, BVetMed DipECVN MRCVS,^1^ S. Behr, DVM (Hons) DipECVN MRCVS^1^

##### 29.1.1.1 ^1^Willows Veterinary Centre and Referral Service, Birmingham, UK

Various fixation techniques have been described for the ventral stabilization of atlantoaxial subluxation (AAS) in dogs, though few allow for fixation using bi‐cortical pedicle screws. The profile of bone cement (PMMA) used in previous ventral fixation systems may result in complications pertaining to implant size/position such as laryngeal irritation, dysphagia, regurgitation, and tracheal necrosis. A 6‐year to 8‐month‐old male‐neutered Maltese terrier weighing 2.3 kg was presented with a two‐week history of ambulatory tetraparesis and generalized proprioceptive ataxia. The spinal reflex and cranial nerve examinations were normal, resulting in a C1–C5 anatomic diagnosis. An AAS and synovial cyst were diagnosed following the MRI of the neck. Computed tomography (CT) was performed for pre‐surgical planning, allowing the design of 3D‐printed patient‐specific drill guides and a 3D‐printed titanium plate, contoured for optimal reduction. The plate was applied over C1–C2 allowing the safe placement of two bi‐cortical 2 mm screws in C1 followed by two bi‐cortical 2 mm screws placed cranially in C2, and then two mono‐cortical 1.5 mm screws placed caudally in C2. It was possible to close the longus colli muscle with no interference of local structures. Post‐operative CT displayed excellent reduction and optimal screw placement without pedicle or vertebral canal breach. The maximum profile of the implant was 4 mm from the ventral cortical surface. At 1 month follow up the patient was comfortable, ambulatory with moderate generalized proprioceptive ataxia, without evidence of complication. This report highlights the benefit of custom 3D‐printed titanium plates as an alternative to bone cement (PMMA) for ventral stabilization of AAS.

## 30 (P 03)

### 30.1 Clinical, MR Imaging, and Histological Description of a Choroid Plexus Papilloma With Disseminated Intraventricular and Spinal Cerebrospinal Fluid Drop Metastases in a Young Dog

#### 30.1.1 G. Albertini, DVM, MRCVS,^1^ A. Malbon, BSc (Hons), BVSc, DVM, PhD, DECVP, MRCVS,^2^ A. Staudacher, DVM, MRCVS, DECVDI,^1^ F. Stabile, DVM, PhD, MRCVS, DECVN^1^

##### 30.1.1.1 ^1^Southfields Veterinary Specialists, Basildon, UK; ^2^The Royal (Dick) School of Veterinary Studies and the Roslin Institute, Glasgow, UK

A 2‐year‐old male entire Cane Corso was presented for investigations into a 1‐week history of ambulatory paraparesis and ataxia in the pelvic limbs gradually deteriorating. Magnetic resonance imaging (MRI) revealed intraventricular space‐occupying lesions affecting the fourth ventricle and lateral apertures and intradural‐extramedullary space‐occupying lesions at the level of C7 vertebra, L4–L5, and L7–S1 intervertebral disc spaces. Due to poor quality‐of‐life, the patient was euthanized. Post‐mortem examination revealed partially encapsulated, multifocally infiltrative, and moderately cellular neoplastic masses. The histological description was similar for all masses. The cells appeared cuboidal with round central nuclei and a moderate amount of eosinophilic cytoplasm and appeared to have been arranged almost exclusively in single‐layered papilliform patterns supported by fibrovascular stroma. Mitoses were rarely observed. The neoplasms were most consistent with a choroid plexus papilloma and drop metastases. This is the first report of a histologically confirmed choroid plexus papilloma causing disseminated MRI‐apparent intraventricular and spinal drop metastases.

## 31 (P 04)

### 31.1 Concurrent Extradural and Intradural/Extraparenchymal Tumors in a Cat: Neuroimaging and Surgical Treatment of a Spinal Meningoma and Infiltrative Lipoma

#### 31.1.1 K. Santifort, DVM, MSc, Board‐Eligible ECVN,^1^ ^,2^ M. Plonek, DVM, PhD, Resident ECVN,^1^ L. Garosi, DVM, Dip. ECVN, FRCVS,^3^ G. Grinwis, DVM, PhD,^4^ D. Rissi, DVM, MSc, PhD, Dip. ACVP^5^

##### 31.1.1.1 ^1^ IVC Evidensia Small Animal Hospital Arnhem, Arnhem, The Netherlands; ^2^ IVC Evidensia Small Animal Hospital Hart van Brabant, Waalwijk, The Netherlands; ^3^Vet Oracle, Norfolk, UK; ^4^Veterinary Pathology Diagnostic Centre, Department of Biomedical Health Sciences, Faculty of Veterinary Medicine, Utrecht University, Utrecht, The Netherlands; ^5^Athens Veterinary Diagnostic Laboratory, Department of Pathology, College of Veterinary Medicine, University of Georgia, Athens, USA

Spinal tumors in dogs and cats can be extradural, intradural/extraparenchymal, or intraparenchymal. In cats, spinal meningiomas are less commonly described than intracranial meningiomas. Concurrent, distinct tumors affecting the spinal cord are rarely reported in human medicine and not reported in cats. A 13‐year‐old castrated male domestic shorthaired cat was presented with chronic progressive spastic paraparesis worsening to paraplegia. Neurological examination was consistent with a T3–L3 myelopathy. Radiographs revealed a lucent area in the dorsal aspect of the L3 vertebral body. CT and MRI revealed an intradural/extraparenchymal mass with secondary spinal cord compression and osteolysis of the L3 vertebral body. Additionally, a paravertebral, intramuscular infiltrative mass with attenuation (CT) and signal (MRI) characteristics of fat with vertebral canal invasion and spinal cord compression (extradural) was identified at L5–6. Surgical removal/debulking was performed via an L3 pediculectomy and partial corpectomy, and an L5–6 hemilaminectomy. Histologic examination was consistent with an L3 meningioma and an L5–6 infiltrative lipoma. The cat was ambulatory within 2 weeks after surgery. Local recurrence of the meningioma and non‐recurrence of spinal cord compression by the infiltrative lipoma was confirmed by repeat MRI at 3 months post‐surgery and the cat (ambulatory on oral prednisolone treatment 0.5 mg/kg q24h) was referred for radiotherapy. The possibility of concurrent, distinct tumors affecting the spinal cord is of clinical importance, especially for decision‐making regarding treatment. This is the first report of (1) concurrent, distinct tumors affecting the spinal cord and (2) an infiltrative lipoma causing spinal cord compression in a cat.

## 32 (P 05)

### 32.1 Erector Spinal Plane Block in Dog Undergoing Hemilaminectomy: Prospective Randomized Clinical Trial

#### 32.1.1 C. Bendinelli, DVM, GPCert (Anesth), Master II Livello Anestesia,^1^ M. D'Angelo, DVM,^1^ F. Leonardi, DVM,^2^ N. Verdier, DVM,^3^ M. Armellini, DVM,^1^ F. Cozzi, DVM, Dipl ECVN,^1^ M. Bonaldi, DVM,^1^ R. Lombardo, DVM, Dipl ACVIM Neurology,^1^ D. A. Portela, DVM, PhD, DACVAA^4^

##### 32.1.1.1 ^1^ NVA Neurologi Veterinari Associati, Milano, Italy; ^2^Department of Veterinary Science, University of Parma, Parma, Italy; ^3^Department of Anaesthesiology and Perioperative Intensive Care Medicine, University of Veterinary Medicine Vienna, Vienna, Austria; ^4^Department of Comparative, Diagnostic, and Population Medicine, College of Veterinary Medicine, University of Florida, Florida, USA

Objective: To compare the perioperative cumulative opioid consumption and the incidence of cardiovascular complications in dogs undergoing hemilaminectomy and receiving either an erector spinal plane (ESP) block or systemic opioids.

Methods: A total of 60 client‐owned dogs were randomized to receive an ESP block (ESP group), a continuous rate infusion of fentanyl (FNT group, positive control), or a single dose of methadone in premedication (MTD group, negative control). Intraoperative nociceptive response was treated with fentanyl (1 μg/kg, IV) boli. Before surgical site closure, morphine (0.1 mg/kg) was applied on the dura mater. The cumulative dose of opioids was recorded and compared among groups. The incidence of cardiovascular complications and the time to extubation were compared between groups. Postoperatively, methadone 0.2 mg/kg, IV was administered according to SF‐GCPS scores. The level of significance was *p* < 0.05.

Results: MTD group required more intraoperative rescue analgesia than groups ESP (*p* = 0.008) and FNT (*p* = 0.001). The total cumulative dose of fentanyl was higher in FNT (*p* < 0.0001) and MTD groups (*p* = 0.002) compared with ESP group. The incidence of cardiovascular complications was similar between groups. Extubation time was longer in group MTD (*p* = 0.03). Postoperatively, the time to first rescue analgesia was longer in ESP than in MTD (*p* = 0.03). The cumulative opioid consumption and pain scores were similar between groups.

Conclusions: The ESP block resulted in reduced intraoperative opioid consumption compared with positive and negative control groups.

## 33 (P 06)

### 33.1 Histopathological and Immunohistochemichal Evaluation of the Effect of Tacrolimus on Nerve Regeneration Following Crushed Sciatic Nerve in Mice

#### 33.1.1 H. Fattahian, DVSc, DVM,^1^ F. Khomejani Farahani, DVSc, DVM,^1^ A. Asghari, DVSc, DVM,^1^ P. Mortazavi, DVSc, DVM^2^

##### 33.1.1.1 ^1^Department of Clinical Science, Science and Research Branch, Islamic Azad University, Tehran, Iran; ^2^Department of Pathobiology, Faculty of Specialized Veterinary Science, Science and Research Branch, Islamic Azad University, Tehran, Iran

Studies have been conducted to find effective agents for nerve regeneration. The present study was conducted on 16 adult male Syrian mice with the aim of assessment of the effects of tacrolimus on crushed sciatic nerve injury and its comparison with spontaneous repair. After exposure of the left sciatic nerve, crushing was performed using a 2 mm wide mosquito hemostatic forceps tip for 10 s. Animals were randomized into two groups of treatment group (Group I), and the control (Group II) with eight mice each. The treatment group received tacrolimus (5 mg/kg, q24h, SC) until the end of the study, and the control group received no therapeutic agents. Histopathological and immunohistochemical assessment was performed on second and fourth post‐operative weeks, and suggested that perineurium formation did not show a significant difference between the study groups (*p* > 0.05). At the end of the second week in the treatment group, the reduction of axon inflammation and swelling, and the increase in axonal count showed a significant difference with the control group (*p* < 0.05). At the end of the fourth week, the difference in inflammation severity between the groups was not significant (*p* > 0.05) but the decrease in axonal swelling and increase in axonal count in the treatment group showed a significant difference with the control group (*p* < 0.05). Increase of glial fibrillary acidic protein (GFAP) expression showed a significant difference between the treatment group and the control group at the end of the study (*p* < 0.05). Based on the present results, the use of tacrolimus was responsible for the reduction of inflammation in a 2‐week period, and at the end of the fourth week, increase of axonal count, and edema suppression caused regeneration of injured nerve.

## 34 (P 07)

### 34.1 Intracranial Cystic Meningioma in a 4‐Month‐Old Dog

#### 34.1.1 E. Hidalgo Crespo, DVM, ECVN Neurology and Neurosurgery Resident,^1^ A. Farré Mariné, DVM DipECVN,^1^ M. Pumarola, DVM, PhD, Dip ECVP,^2^ A. Luján Feliu‐Pascual, DVM MRCVS DipECVN^1^

##### 34.1.1.1 ^1^Neurology/Neurosurgery Service, AÚNA Especialidades Veterinarias Hospital, Valencia, Spain; ^2^Murine and Comparative Pathology Unit, Department of Animal Medicine and Surgery, Universitat Autònoma, Barcelona, Spain

Meningiomas are the most common primary brain tumors in dogs, with 12.5%–32% subclassified as cystic meningiomas; however, this form has never been reported in puppies. A 4‐month‐old male entire Golden Retriever presented with 1‐week history of apathy, hyporexia, and cervical pain. Neurological examination showed obtundation, left head‐tilt, proprioceptive deficits in right limbs, vertical nystagmus, absent right menace response, and decreased on the left. Neurolocalization was multifocal, considering congenital malformation (hydrocephalus or dermoid, epidermoid, colloid, intra‐arachnoid cyst), immune‐mediated/infectious meningoencephalitis, or neoplasia. Blood tests and thoracic radiographs were unremarkable. Computed tomography revealed an intra‐axial, ill‐defined, heterogeneously attenuating, cystic lesion with hypoattenuating areas, affecting left temporal, parietal, and occipital lobes causing severe mass effect with left lateral ventricle collapse, caudal transtentorial and foramen magnum herniations. Isoattenuating areas enhanced after iodinated IV contrast. Surgical biopsy was undertaken through a left rostrotentorial approach, but intraoperative cytology of the infiltrative mass was consistent with malignant sarcoma and euthanasia was performed. Histopathology and immunohistochemistry revealed malignant anaplastic meningioma (grade III) with cystic features. In human and veterinary medicine, meningiomas are treated with surgical resection, radiation therapy, and rarely chemotherapy. Surgically treated meningiomas in children have good prognosis. In our case, intraoperative euthanasia was based on large size, infiltrative pattern, and results of intraoperative cytology, which made complete excision unfeasible. Although rare, cystic meningiomas should be included as differential diagnosis in puppies with intracranial masses with compatible imaging features.

## 35 (P 08)

### 35.1 The Effect of Exogenous Testosterone and Tacrolimus on Experimental Sciatic Nerve Injury in Rat

#### 35.1.1 M. Jazinidorcheh, DVM, DVSc,^1^ H. Fattahian, DVM, DVSc,^1^ A. Aliaghaei, PhD,^2^ M. Abdollahifar, PhD,^2^ H. Azarabad, DVM, DVSc,^3^ M. Ghanaatpisheh, DVM, DVSc,^4^ H. Salehpour, DVM,^4^ H. Sharifi Nistanak, DVSc^1^

##### 35.1.1.1 ^1^Department of Clinical Science, Faculty of Specialized Veterinary Sciences, Science and Research Branch, Islamic Azad University, Tehran, Iran; ^2^Department of Biology and Anatomical Sciences, School of Medicine, Shahid Beheshti University of Medical Sciences, Tehran, Iran; ^3^Department of Pathology, Hamidreza Fattahian Small Animal Hospital, Tehran, Iran; ^4^Department of Diagnostic Imaging, Hamidreza Fattahian Small Animal Hospital, Tehran, Iran

Introduction: Peripheral neuropathy with a prevalence of ~2.6% is one of the most common disorders and affects a large number of animals. A high percentage of traumatic injuries are caused by car accidents. This research evaluates the role of exogenous testosterone (Tes) and tacrolimus (Tac) alone and the combined form following the damage of sciatic nerve on uncastrated male rats experimentally.

Methods: Twenty adult male Wistar rats were randomly divided into four groups and 5 rats in each group. With standard approach, the sciatic nerve was crushed for 10 s in test groups. The rats of sham group did not receive any medicine; the rats of Tes group received Tes (5 mg/kg, Sc); the rats of Tac group received Tac (5 mg/kg, Po); the rats of “Tes and Tac” group received Tes (5 mg/kg, Sc) and Tac (5 mg/kg, po) daily for 4 weeks. After 4 weeks, gross observation, toe‐spreading reflex (TSR), toe out angle (TOA), open field Test (OFT), treadmill and rota‐rod performance test, sciatic functional index (SFI), Electromyogram evaluation (EMG) were investigated.

Results: The results of all tests of the Tes + Tac group were closer to the sham group. However, in the EMG and OFT tests, the results of the Tes group did not show a significant difference with the sham group, and with a slight difference, the Tes group was better than the Tes + Tac. Tac did not have a significant effect and alter the crushed nerve. Conclusion: In general, in all evaluations and gait tests, Tes + Tac group showed better sciatic nerve function after injury to the others test groups.

## 36 (P 09)

### 36.1 Surgical Treatment of an Intradural–Intramedullary Arteriovenous Malformation in a Rhodesian Ridgeback

#### 36.1.1 F. Stabile, DVM MRCVS PhD DECVN,^1^ J. M. French, BVSc MSc DACVR,^2^ E. Mahon, BVM BVS MRCVS^1^

##### 36.1.1.1 ^1^Southfields Veterinary Specialists Part of Linnaeus Ltd, Basildon, UK; ^2^Antech Imaging Service, 17 672‐B Cowan, Irvine, California, USA

The aim of this study is to report the successful outcome of surgical management for an intradural‐intramedullary arteriovenous malformation (AVM) in an 18‐month‐old, female, Rhodesian ridgeback. The dog presented for evaluation of a 2‐month history of progressive, non‐painful paraparesis and pelvic‐limb ataxia. Clinical examination was unremarkable. Neurological examination was compatible with a T3–L3 non‐painful myelopathy. Hematology and serum‐biochemistry were normal. MRI of the vertebral column from T3 vertebra to the sacrum revealed multifocal, tortuous, well‐defined, signal voids in T1W and T2W series, non‐to‐moderately heterogeneously contrast‐enhancing structures in the subarachnoid space, and intramedullary regions of abnormal signal intensity and heterogeneous contrast enhancement. Differential diagnoses included developmental abnormalities of vascular origin with flow‐related signal loss such as AVM and, aberrant parasitic migration. Cerebrospinal‐fluid analysis of a sample collected from the lumbar region revealed albumin‐cytological‐dissociation with a protein concentration of 0.64 g/L (ref < 0.45 g/L). Parasitology testing on were negative. The AVM was surgically removed via a continuous T6–T10 left‐sided dorsal‐laminectomy and T10–T11 hemilaminectomy. After durectomy the visible abnormal vascular structures were isolated, followed intramedullary, and removed via bipolar cauterization. Any intraoperative bleeding was controlled with application of oxidized cellulose material. Histopathology of the abnormal vascular structures was compatible with arterial wall. The dog recovered uneventfully from surgery and an 8‐month follow‐up examination revealed mild ataxia. Although MRI, CT, and post‐mortem features of a similar case have been previously reported this is the first report of successful surgical treatment for intradural and intramedullary AVM in a dog.

## 37 (P 10)

### 37.1 In Vitro Cytotoxicity Study on Canine Meningioma of Grade I With Somatostatin Analog (Octreotide). A Preliminary Study

#### 37.1.1 M. T. Mandara, Pr,^1^ A. Tognoloni, Dr,^1^ E. Chiaradia, Dr^1^

##### 37.1.1.1 ^1^Department of Veterinary Medicine, University of Perugia, Italy

Somatostatin is a hormone for which antimitotic and pro‐apoptosis properties are well known. Therefore, tumors expressing somatostatin receptors (SSTRs) can be responsive to treatments with somatostatin or its analogs. Human meningioma expresses SSTR2 although the correlation between SSTR2 expression and histological grade or subtype is still debated. Expression of SSTR2 has been reported also in the canine meningioma without significant differences between Grade I and Grade II, but much less in Grade III. In this study, we prepared cell cultures from four meningiomas of Grade I, of meningothelial, microcystic, rhabdoid, and angiomatous subtypes, respectively. The neoplastic cells were characterized by positive immunoreaction to vimentin, EMA, and SSTR2 antibodies. Cytotoxicity studies were performed with octreotide (OCT) at 0.1, 1, 10, and 100 nM concentrations for 24 and 48 h using the MTT test. Data obtained from four independent experiments performed in triplicate have been submitted to statistical analysis with ANOVA one way and then the Bonferroni test for multiple comparisons. *p* < 0.05 was assumed as a significance level. MTT vitality assay showed that OCT is able to decrease the number of viable cells in vitro of canine meningioma of Grade I, showing a dose and administration time‐dependent effect. In particular, doses of 10 and 100 nM produced statistically significant effects after 48 h (*p* < 0.05). Similar doses did not produce significant antiproliferative effects after 24 h. These results support further advanced studies with somatostatin analogs as a novel potential target therapy in canine meningioma, as an alternative, or in association with surgery excision.

## 38 (P 11)

### 38.1 Skin Biopsy as a Diagnostic Tool for Peripheral Neuropathies of Dogs. Set Up of an Indirect Immunofluorescence Protocol

#### 38.1.1 M. T. Mandara, Pr.,^1^ S. Arcaro, Dr.,^1^ I. Porcellato, Dr.,^1^ G. Giglia, Dr.^1^

##### 38.1.1.1 ^1^Department of Veterinary Medicine, University of Perugia, Italy

In veterinary medicine, the diagnosis of peripheral neuropathies is currently performed on semithin sections or nerve fiber teasing from nerve biopsy, which remains a diagnostic tool for specialized neurologists. Albeit they assure an accurate morphological study of axons and myelin, actually they fail to identify more specific length‐dependent and somatosensitive neuropathies. In humans, a skin biopsy is a tool of used in this purpose, identifying and counting the nerve fibers crossing dermo‐epidermal junction with indirect immunofluorescence (IIF). Unfortunately, the need for frozen samples limits the routinary application of this technique.

In this study, we set up an IIF protocol with epifluorescence microscopy in the dog. Starting from formalin‐fixed paraffin‐embedded (FFPE) skin biopsies performed with an 8 mm punch on animals died for causes not concerning skin diseases, and submitted to necropsy with owner's consent, we applied an anti‐PGP9.5 antibody, checking the thickening of sections, the magnification of observation, and the image capture modality (with co‐localization vs. without co‐localization). The best results in terms of clarity of fibers and the possibility of counting them on IIF were obtained on 10 μm sections, at a high‐power field (40×), without co‐localization for nuclei (DAPI) and epithelia (cytokeratin). To count the nerve fibers crossing the dermo‐epidermal junction per number of 40× fields, more than per length of sample avoids issues related to technical artifacts. This protocol supports us to promote FFPE skin biopsy in the diagnosis of selected peripheral neuropathies of the dog. Reference data concerning nerve fibers density in different skin regions from health animals of different ages need to be acquired for diagnostic purposes.

## 39 (P 12)

### 39.1 Concurrent Steroid‐Responsive Meningitis‐Arteritis and Immune‐Mediated Hypertensive Uveitis With Iridial Hemorrhages in a Dog

#### 39.1.1 L. Villalonga Rodríguez, Gdo. Vet.,^1^ M. Caro Suárez, Gdo. Vet.,^1^ F. Laguna Sanz, Ldo. Vet., DipECVO.,^1^ P. Amengual Batle, Ldo. Vet., DipECVN^1^

##### 39.1.1.1 ^1^Hospital Veterinario Puchol, Madrid, Spain

Physical and neurological examination revealed multiple hemorrhagic lesions affecting the iris and severe pain on palpation and manipulation of the neck, along with a rectal temperature of 40.5°C, but no neurological deficits were found. Ophthalmological exam was performed and showed iridial hemorrhages and positive aqueous flare in both eyes. The left eye had increased intraocular pressure. Fundic examination was normal. Full hematology and serum biochemistry revealed marked neutrophilic leucocytosis. C‐reactive protein (CRP) levels were also increased (138 mg/L, < 15 mg/L). MRI scan of the cervical region showed mild, diffuse meningeal enhancement. Cerebrospinal fluid analysis from the cisterna magna demonstrated marked non‐degenerated neutrophilic pleocytosis and increased protein levels, compatible with steroid‐responsive meningitis‐arteritis (SRMA). *Toxoplasma gondii* and *Neospora caninum* serologies were considered negative. Immunosuppressive treatment was started with an intravenous dose of 0.3 mg/kg of dexamethasone, followed by prednisolone at 2 mg/kg orally once daily. After 2 weeks, ophthalmic signs showed marked improvement, with negative aqueous flare and a reduction in iridial inflammation. After 4 weeks, clinical signs of fever and cervical pain were resolved, CRP levels were within normal limits (7.2 mg/L) and prednisolone tapering was attempted, with no signs of relapse to date. To the best of the author's knowledge, this is the first report of concurrent SRMA and bilateral hypertensive uveitis in a dog, with complete resolution of clinical signs with immunosuppressive therapy.

## 40 (P 13)

### 40.1 Association of Intervertebral Disc Degeneration in Magnetic Resonance Imaging and Computed Tomography

#### 40.1.1 F. Taylor‐Brown, BSc, BVetMed, MVetMed, DipECVN, MRCVS,^1^ S. Bertram, DVM, MVetMed, MVetMed, MRCVS^1^

##### 40.1.1.1 ^1^Cave Veterinary Specialists, Wellington, UK

Objective: The Pfirrmann system has been used to assess intervertebral disc (IVD) degeneration on magnetic resonance imaging (MRI). Computed tomography (CT) can also be used to assess IVD degeneration and is highly sensitive for the detection of calcification within the IVD. It is unclear how the Pfirrmann IVD grade relates to IVD calcification as detected by CT. The objective of the study was therefore to describe the association between the Pfirrmann IVD grade on MRI and the presence or absence of IVD calcification on CT.

Methods: Dogs that underwent both MRI and CT scan of the thoracolumbar vertebral column (T10‐L7) on the same day were included. IVD degeneration was graded on MRI using Pfirrmann system (adapted for veterinary application) and blinded to the results of the MRI assessment, a corresponding CT scan was also evaluated for the presence or absence of IVD calcification. Association between Pfirrmann grading and IVD calcification on CT scan was assessed.

Results: Ninety‐four dogs were included in the study with a total of 730 IVD reviewed on both MRI and CT. Of the IVD evaluated, 51 (7%) demonstrated calcification on CT. Pfirrmann Grades 1 and 5 were not associated with calcification. Pfirrmann Grades 2–4 were calcified in 2.5%, 8.8%, and 50% of the IVD assessed.

Conclusion: The association between Pfirrmann grade and IVD calcification is poor, except for Pfirrmann grade 4 where half of the IVD can be expected to be calcified on CT. Pfirrmann grading is not useful to assess presence or absence of IVD calcification in dogs.

## 41 (P 14)

### 41.1 Follow‐Up Study of Corpus Callosotomy in Dogs With Medication‐Resistant Epilepsy

#### 41.1.1 R. Asada, DVM, PhD,^1^ D. Hasegawa, DVM, PhD, AiCVIM (Neurology),^1^ Y. Yu, DVM, PhD,^1^ S. Mizuno, DVM,^1^ A. Shigeyoshi, DVM,^1^ Y. Hamamoto, DVM, PhD,^1^ ^,2^ Y. Kobatake, DVM, PhD^3^

##### 41.1.1.1 ^1^Laboratory of Veterinary Clinical Neurology, Nippon Veterinary and Life Science University, Tokyo, Japan; ^2^Companion Animal Surgery Unit, Rakuno Gakuen University, Hokkaido, Japan; ^3^Laboratory of Veterinary Internal Medicine, Gifu University, Gifu, Japan

Corpus callosotomy (CC) is one of the disconnection surgeries for generalized seizures or focal seizures evolving into generalized seizures. Since 2018 we have been performing CC in dogs with medication‐resistant epilepsy. Here we report the long‐term outcomes of 5 cases with over 1‐year follow‐up periods. To evaluate the acceptability and efficacy of CC, postoperative seizure frequency, changes in seizure type, complications, activities of daily living (ADL) and owner‐rated visual analog scale (VAS) scores, and MRI and EEG findings were obtained. The seizure reduction rates for each dog were; 80% over 4 years (Dog 1), 96% over 3 years (Dog 2), 50% over 2 years (Dog 3), and 0% and 52% over 1 year (Dogs 4 and 5), respectively. Median longest seizure‐free interval was 50 days (range: 12–199). None of the cases developed postoperative complications that worsened their QOL. Despite incomplete seizure control, all cases showed improved ADL and VAS scores after surgery, indicating high owner satisfaction. MRI showed small hemorrhagic or necrotic lesions around the surgical sites in 4 cases, and EEG showed desynchronized and independent epileptic discharges bilaterally in all cases. One case showed a transient cognitive decline, suspected to be separation syndrome.

Because CC is a palliative surgery, no cases achieved complete seizure freedom; however, the majority showed = 50% reduction. Furthermore, no serious postoperative complications were reported in any cases, and the owners expressed high satisfaction with CC. These results suggest that CC is an effective and acceptable treatment option for dogs with medication‐resistant epilepsy.

## 42 (P 15)

### 42.1 Clinical Presentation, Diagnostic Investigations, and Follow‐Up of an Ambulatory Tetraparetic Bengal Tiger ( *Panthera tigris tigris* )

#### 42.1.1 S. Mosconi, DVM,^1^ M. Morici, DVM, PhD, GPCert (ExAP),^2^ E. Auriemma, DVM, Dipl. ECVDI,^1^ S. Di Graci, DVM, GPCert (ExAP),^2^ A. Calloni, DVM,^1^ F. Tirrito, DVM, MRCVS, Dipl. ECVN^1^ ^,3^

##### 42.1.1.1 ^1^ AniCura Istituto Veterinario di Novara, Novara, Italy; ^2^Pombia Park S.R.L., Pombia, Italy; ^3^Studio Veterinario Associato Vet2Vet di Ferri e Porporato, Torino, Italy

An 11‐year‐old, male Bengal tiger ( *Panthera tigris tigris* ) was referred for a 2‐week history of ambulatory tetraparesis, generalized ataxia and hypermetric gait, associated with mild right head tilt and spontaneous proprioceptive deficit of the right forelimb. Neuroanatomical localization was C1–C5 myelopathy; a cerebellum–vestibular system involvement was also considered. Blood exams were unremarkable, serum vitamin A concentration (0.11 mg/L) was slightly below the reference range (0.17–0.36 mg/L). Indirect hemagglutination test for *Toxoplasma gondii* was indicative of parasite exposure (antibody titer 1:640). Pre and post contrast computed tomography of the head and the cervical column showed hypertrophic degenerative remodeling of the articular joint processes, causing severe vertebral canal stenosis and bilateral spinal cord compression at C2–C3 intervertebral space. In addition, bilateral otitis media was present, without imaging signs of intracranial extension of the inflammation. Video‐otoscopy documented the bilateral integrity of the tympanic membranes; the brainstem auditory evoked potential test was consistent with a partial conductive deafness.

Cerebrospinal fluid (CSF) analysis resulted normal (total nucleated cells count 0/μL, protein concentration 18 mg/dL); CSF PCR for *Toxoplasma gondii* was negative. A diagnosis of osseous‐associated cervical spondylomyelopathy (OA‐CSM) and concurrent bilateral otitis media was obtained. Glucocorticoids, movement restriction, and vitamin A supplementation were instituted; in addition, clindamycin was added to treat the bilateral otitis and because of the serological positivity to *Toxoplasma gondii*.

Follow‐up after 4 weeks showed mild improvement of the clinical signs, even though the ambulatory tetraparesis persisted. To the authors' knowledge this is the first report of OA‐CSM in a tiger.

## 43 (P 16)

### 43.1 Atlantoaxial Subluxation Stabilization Using Intervertebral Screws and Polymethylmethacrylate (PMMA) in Two Cats

#### 43.1.1 D. D. Lu, BVetMed, MVM, DipECVN, CVA^1^

##### 43.1.1.1 ^1^Neurology Service, CityU Veterinary Medical Centre, Kowloon, Hong Kong

Atlantoaxial subluxation (AAS) is rare in cats. This is a retrospective descriptive clinical case report, the first report using intervertebral screws and polymethylmethacrylate to stabilize AAS in two cats.

Both cats were middle aged (7 years old), shorthaired pedigree cats (Case 1 Exotic, Case 2 American), presented for paraparesis for 2 weeks (Case 2) to 2 months (Case 1), progressed to acute tetraparesis (Case 2) to tetraplegia (Case 1) following a fall. Neurological examination revealed non‐ambulatory tetraparesis, thoracic worse than pelvic limbs, upper motor neuron sign, deep pain positive and variable neck pain. Dynamic spinal radiography confirmed AAS in both cats. Dens is absent in Case 2. MRI detected severe extensive cord swelling in Case 1 and mild focal cord swelling in Case 2. CT identified abnormal congenital fusion of C1 to occipital bone (Case 2). Lumbar CSF showed moderate neutrophilic pleiocytosis (Case 1). Ventral approach to AA revealed moderately thickened joint capsule in both cats. Self‐tapping cortical screws (all 2 mm) were placed bilaterally in C1 pedicles, cranial aspect of C2 bilaterally and midline caudal aspect of C2. Two additional self‐drilling self‐tapping screws were drilled into C1 body for better bone cement anchor (Case 1). Cancellous bone autograft was harvested from both humeral heads and packed into the atlantoaxial joint. All screws were encased in PMMA. Postoperative radiography showed satisfactory AAS stabilization. Outcome is good as both cats regained unassisted locomotion within 2 weeks. Intravertebral screws and PMMA can be used to repair AAS in cats.

## 44 (P 17)

### 44.1 Presumptive Case of Prefixed Brachial Plexus in a Non‐Ambulatory Paraparetic Dog

#### 44.1.1 F. Tirrito, DVM, MRCVS, Dipl. ECVN,^1^ ^,2^ G. Costanzo, DVM,^1^ A. Nespro, DVM,^3^ B. Betti, DVM,^1^ M. Scuttari, DVM^1^ ^,3^

##### 44.1.1.1 ^1^ AniCura Istituto Veterinario di Novara, Novara, Italy; ^2^Studio Veterinario Associato Vet2Vet di Ferri e Porporato, Torino, Italy; ^3^Neurologica S.R.L., Torino, Italy

The brachial plexus originates just outside of the intervertebral foramina and is formed by the ventral branches of C5–C8 and T1–T2 spinal nerves.

Variations in the nerve roots contribution to the brachial plexus are described and the structure of the brachial plexus can occasionally originate more cranially or caudally; these exceptions are called prefixed and postfixed brachial plexus, respectively. In human medicine, the prevalence of prefixed brachial plexus is 10%–65%, while the postfixed brachial plexus is reported in 7.5% of cases.

In veterinary medicine, little is known about prefixed and postfixed brachial plexus and, to the authors' knowledge, no reports have been published in dogs. Our aim was to describe the neurological presentation, imaging findings, and outcome of a dog with a prefixed brachial plexus, affected by a disc herniation.

A 5 year‐old, male, Dachshund dog was referred for progressive history of non‐ambulatory paraparesis; postural reactions were normal in the thoracic limbs and segmental spinal reflexes were intact in all limbs. The neurological examination suggested a T3–L3 myelopathy. Computed tomography and magnetic resonance imaging studies of the thoracolumbar and cervical column demonstrated a C7–T1 intervertebral disc extrusion, causing severe spinal cord compression.

A diagnosis of intervertebral disc herniation in a dog affected by a prefixed brachial plexus was made.

The dog underwent a ventral slot and his neurological status quickly improved following surgery. Prefixed brachial plexus should be suspected when the neuroanatomical localization is T3–L3, but a more cranial spinal cord lesion is demonstrated with diagnostic imaging studies.

## 45 (P 18)

### 45.1 Novel MRI and Histopathological Findings in a Young Bullmastiff Crossbreed Dog With Mitochondrial Fission Encephalopathy

#### 45.1.1 E. Suiter, BSc BVetMed PGDip (VCP),^1^ K. Baiker, DVM ECVP SFHEA,^2^ A. Kaczmarska, DVM,^3^ M. Christen, DVM,^4^ T. Leeb, PhD,^4^ A. Orobia, DVM MSc,^1^ C. Anselmi, DVM DipECVDI,^1^ J. Minguez, DVM,^1^ R. Gutierrez‐Quintana, MVZ MVM DipECVN^3^

##### 45.1.1.1 ^1^Pride Veterinary Referrals, Derby, UK; ^2^Department of Veterinary Clinical Sciences, Hong Kong, Hong Kong; ^3^Small Animal Hospital University of Glasgow, Glasgow, UK; ^4^Institute of Genetics Vetsuisse Faculty University of Bern, Bern, Switzerland

Mitochondrial fission encephalopathy (MFE) has been described in the Bullmastiff with a proposed causative homozygous frameshift variant in the MFF gene. Bullmastiffs with this genetic variant share a similar clinical phenotype characterized by chronic and progressive ataxia, impaired vision, and behavior abnormalities. Magnetic resonance imaging (MRI) of these dogs consistently displays bilateral symmetrical lesions localized to the cerebellar nuclei and widening of the cerebral cortex sulci, with enlargement of the ventricular system. The present case report describes novel MRI and histopathological features in a young crossbreed dog testing homozygous for the same frameshift variant in the MFF gene. A 9‐month‐old male Bullmastiff crossbreed dog was presented with a history of progressive proprioceptive ataxia and behavior changes. The sire was a Bullmastiff, and the dam was a Bullmastiff crossed Staffordshire Bull Terrier. MRI showed diffuse cerebral sulci widening and dilation of the ventricular system consistent with brain atrophy that has previously been reported in the current literature. A second MRI performed 6 months later confirmed progression of brain atrophy as well as newly identified rounded, well‐defined, bilaterally symmetrical T2W, FLAIR, and T2* hyperintense intra‐axial lesions affecting the olivary nuclei. Histopathologically, the olivary nuclei had extensive disintegration and vacuolation of the neuropil with partial neuronal preservation. This case report documents diagnostic imaging and histopathological findings not previously reported in dogs affected with mitochondrial fission encephalopathy, suggesting a different selective regional vulnerability of the neurons. It could be hypothesized that this change in lesion distribution could be due to different genetic background, or alternatively diseases progression not observed in previous cases.

## 46 (P 19)

### 46.1 Dynamic Susceptibility Contrast Magnetic Resonance Imaging in Dogs With Ischemic Cerebrovascular Disease: A Study of 21 Cases

#### 46.1.1 R. Orlandi, DVM, FHEA, DipECVN,^1^ J. R. Mortier, DVM, CEAV (IM), CES (CP), CPS (HE), Di

##### 46.1.1.1 ^1^ Department of Veterinary Science, Small Animal Teaching Hospital, University of Liverpool, Neston, UK; ^2^ Department of Clinical Sciences, North Carolina State University College of Veterinary Medicine, Raleigh, USA

The aim of the study was to analyze brain perfusion in dogs with naturally‐occurring territorial ischemic strokes. Patients diagnosed with ischemic stroke between 2015 and 2020 which underwent dynamic susceptibility contrast magnetic resonance imaging were included. Relative cerebral blood flow (rCBF), relative cerebral blood volume (rCBV), mean transit time (MTT), and time to peak (TTP) were measured in several regions of interest (ROI) located in the following regions: T2w hyperintense lesion, corresponding contralateral region, and ROIs distant to the lesion. These perfusion parameters were also measured in 45 control dogs and compared with the study group. Twenty‐one dogs met the inclusion criteria. Eighteen dogs underwent MRI within 48 h of onset of clinical signs; the remaining three dogs within 3–7 days. Dogs with ischemic strokes had significantly lower rCBF and rCBV (*p* < 0.001) in the lesion compared with the corresponding contralateral region, areas distant to the lesion, and areas in control dogs. Most patients (*n* = 14) showed prolonged MTT and TTP in the lesion; however, seven patients showed similar or shortened MTT. Results suggest that the brain tissue of dogs with suspected ischemic strokes showed significant hypoperfusion in the T2w hyperintense lesion compared with other areas of the brain and compared with control dogs.

## 47 (P 20)

### 47.1 Ventral Fixation and Surgical Decompression of a Complex Occipito‐Atlanto‐Axial Malformation Using a Custom‐Made Plate in a Cat

#### 47.1.1 C. Aires Serrano, Resident of European College of Veterinary Neurology,^1^ A. Farré Mariné, DipECVN,^1^ A. Luján Feliu‐Pascual, DipECVN^1^

##### 47.1.1.1 ^1^ AÚna Especialidades Veterinarias IVC Evidensia, Valencia, Spain

Occipitoatlantoaxial malformations (OAAMs) are rare in cats with only one described case treated surgically. We report the first cat with an OAAM treated with a two‐stage surgery consisting of ventral fixation using a custom‐made plate based on a 3D‐printed reconstruction of the skull and cranial cervical vertebral column and a later decompressive dorsal C1 laminectomy. A 4‐month‐old neutered female Maine Coon cat was evaluated because of progressive non‐ambulatory tetraparesis. The neurological examination located the lesion between C1 and C5 spinal cord segments, and a myelo‐computed tomography (CT) showed left occipital condyle agenesis, C1 ventral arch thickening, atlanto‐axial joint hypertrophy and overlapping, and non‐union of the odontoid process, producing severe spinal cord compression. Using a 3D‐printed model of the malformed joints, a custom‐made plate was used to ventrally stabilize the occipital condyles, the body of the atlas, and the body of the axis with six cortical screws. The atlanto‐axial overlapping could not be resolved with traction, a control CT revealed remnant spinal cord compression and a dorsal laminectomy of the atlas was performed the following day achieving adequate decompression. The cat regained ambulation 3 weeks after surgery and although control radiographs revealed slight displacement of the right occipital condyle screw 3 months after surgery, the cat remains neurologically normal 4 years after surgery. The use of a custom‐made plate based on a 3D‐printed reconstruction can help to minimize the risks of nervous tissue injury during the surgical treatment of complex vertebral malformations with excellent long‐term results in this case.

## 48 (P 21)

### 48.1 Fly Catching Syndrome Responsive to Gluten‐Free Diet in a French Bulldog

#### 48.1.1 G. Galli, DVM, ECVN Resident,^1^ S. Uccheddu, DVM, PhD, Dipl. ECAWBM,^2^ M. Menchetti, DVM, PhD, Dipl. ECVN^1^

##### 48.1.1.1 ^1^ San Marco Veterinary Clinic and Laboratory, Neurology and Neurosurgery Division, Veggiano (PD), Italy; ^2^ San Marco Veterinary Clinic and Laboratory, Behavioral Medicine Division, Veggiano (PD), Italy

Fly‐catching syndrome (FCS) is a rare condition typically characterized by episodes during which affected dogs bite or lick the air and jump for no apparent reason. The etiology of FCS is still debated. Among veterinary literature, obsessive–compulsive disorders, focal epileptic seizures, and underlying gastrointestinal diseases were considered the most likely triggering causes. Recently, gluten‐sensitive dyskinesia has been described in dogs, but it has never been reported associated with FCS. A 6‐year‐old French Bulldog was presented for a 2‐months history of episodes characterized by sudden onset of jumping and turning around while trying to catch something in the air without consciousness impairment or autonomic signs. The episodes could be interrupted by the owner and lasted several minutes. The owners did not report any other abnormal behavior. The dog suffered from chronic gastrointestinal signs such as borborygmi and diarrhea. The neurological examination showed the presence of episodes suggestive of FCS during the consult. The serological test for anti‐gliadin and anti‐transglutaminase‐2 resulted above the reference range (3.092 and 0.929, respectively; normal range: < 0.8). Consequently, an exclusively gluten‐free diet was started. A complete resolution of episodes was reported during follow‐up after 3 months. To the author's knowledge, this is the first report of FCS associated with positive anti‐gliadin and anti‐transglutaminase‐2 antibodies responsive to a gluten‐free diet. The typical manifestation of the episodes and the response to diet support the hypothesis that FCS may be associated with gastrointestinal disorders. However, more studies are needed to confirm this hypothesis.

## 49 (P 22)

### 49.1 Ronidazole Neurotoxicity in a Boston Terrier

#### 49.1.1 G. Galli, DVM, ECVN Resident,^1^ M. Menchetti, DVM, PhD, Dipl. ECVN^1^

##### 49.1.1.1 ^1^ San Marco Veterinary Clinic and Laboratory, Neurology and Neurosurgery Division, Veggiano (PD), Italy

Ronidazole is an antiprotozoal drug used for the treatment of *Tritrichomonas foetus* and *Giardia* spp. infections. Ronidazole neurotoxicity has been reported in cats and seems to be related to the dosage used. Neurological signs usually appear 3–9 days after starting ronidazole therapy and most frequently include lethargy, pelvic‐limb weakness, limb tremors, and ataxia. An 8‐year‐old male Boston Terrier was referred because of the sudden onset of difficulties walking on the hindlimbs. The dog was receiving ronidazole 42 mg/kg every 12 h since a week because of a Giardiasis infection. The neurologic exam showed wide‐base stance, mild vestibulo‐cerebellar ataxia, tetraparesis worse in the hindlimbs with mild flaccidity, withdrawal hyporeflexia of hindlimbs, horizontal nystagmus to the left which became rotatory lifting the head. These findings were consistent with a multifocal neurolocalization involving the right vestibular system, the cerebellum, and possible neuromuscular involvement. Complete bloodwork and MRI of the brain and lumbosacral spinal cord did not reveal any relevant alterations, as well as the analysis of the cerebrospinal fluid. Electrodiagnostic tests, comprising electromyography, motor nerve conduction, and F‐waves, did not show any abnormal muscular activity and the motor nerve conduction was normal. Hence, ronidazole intoxication was considered more likely and its administration was interrupted. Neurological signs gradually improved over the next 10 days. To our knowledge, neurological signs due to ronidazole therapy were described only experimentally in dogs. This differential diagnosis should be considered in cases of the onset of neurological signs in dogs being administered with ronidazole.

## 50 (P 23)

### 50.1 Bacterial Venous Thrombosis in Internal Vertebral Venous Plexus as a Cause of Severe Painful Cervical Myelopathy in Dobermann Dog

#### 50.1.1 A. Olszewska, Dr.,^1^ A. Czerwik, PhD,^1^ A. Greshake, Doctoral student,^1^ C. Paulus, Doctoral student,^1^ M. J. Schmidt, Prof^1^

##### 50.1.1.1 ^1^ Department of Veterinary Clinical Sciences, Small Animal Clinic, Justus‐Liebig‐University, Frankfurter Str. 114, 35 392 Giessen, Giessen, Germany

To our knowledge, bacterial venous sinus thrombosis has only once been described in a dog as a cause of cervical myelopathy. Here, we present the first documented case of a dog with confirmed *Staphylococcus aureus* bacteremia, which resulted in the occlusion of the internal vertebral venous plexus, causing cervical myelopathy accompanied by fever. The 8‐year‐old intact male Dobermann was introduced with an acute onset of cervical hyperesthesia, ventroflexion of the head, kyphosis, and fever, persisting for 2 weeks. Blood analysis revealed a moderate increase in C‐reactive protein levels and leucocytosis. Magnetic resonance imaging of the cervical and thoracic spinal cord demonstrated enlargement of the right neuroforamen and the right spinal nerve at the level of Th1/2. Furthermore, the right internal vertebral venous plexus displayed a filling defect upon intravenous contrast administration, indicating the formation of a thrombus. Analysis of the cerebrospinal fluid revealed mild mononuclear pleocytosis. While urine culture yielded negative results, a venous blood sample obtained for blood culture indicated the presence of *Staphylococcus aureus* bacteremia. Thromboelastogram confirmed the presence of diffuse hypercoagulability. The treatment including administration of antibiotics, enoxaparin, and analgesics, resulted in an immediate resolution of neurological signs and fever within a day. The exact source of the bacterial thrombus remains unknown. A follow‐up neurological examination conducted after 4 weeks revealed no abnormalities. Bacterial venous thrombosis within the internal vertebral venous plexus is an infrequent yet significant cause of severe cervical myelopathy and fever in dogs.

## 51 (P 24)

### 51.1 Presumed Startle Disease in a Litter of Old English Sheepdogs

#### 51.1.1 M. Hermans, DVM, Dipl ECVN,^1^ B. De Jonge, DVM,^2^ B. Broeckx, DVM, PhD, Professor,^3^ F. Boeykens, DVM,^3^ L. Adant, DVM,^3^ S. Ooms, DVM,^1^ K. Bossens, DVM, Dipl ECVN^1^

##### 51.1.1.1 ^1^ Nesto Veterinary Referral Center Orion, Herentals, Belgium; ^2^ Department of Pathobiology, Pharmacology and Exotics, Faculty of Veterinary Medicine, Ghent University, Merelbeke, Belgium; ^3^ Laboratorium for Animal Genetics, Faculty of Veterinary Medicine, Ghent University, Merelbeke, Belgium

Startle disease or hyperekplexia is a hereditary glycine transmission disorder, characterized by general muscle hypertonia, progressive feeding difficulties, and apnea, triggered by noise and/or touch stimuli. In dogs, symptoms typically start 5–7 days after birth and are progressive. As there is no known treatment, the disease is fatal. Genetic mutations are known in dogs, cattle, mice, and humans. A 2‐week‐old litter of three Old English Sheepdog puppies was presented with complaints of episodic generalized muscle hypertonia and cyanosis since the moment of ambulation. The episodes were mainly touch‐ and noise‐induced. The first symptoms started around the age of 7 days and rapidly progressed. Clinical and neurological examination revealed no abnormalities. Limited bloodwork, including glucose, electrolytes, and calcium, was within normal limits. Creatinine kinase levels were mildly elevated. Electromyography in an awake pup revealed normal Muscle Action Potentials during muscle hypertonia and became electrically silent during muscle relaxation. Based on the neurological presentation and diagnostic findings, Startle Disease was suspected. Clonazepam treatment failed to give improvement. Due to the lack of response to the therapy and progression of clinical signs, the puppies were euthanized at the age of 5 weeks. A full necropsy of the central and peripheral nervous system showed microscopic perineuronal incrustations at the spinal cord, suggestive of ischemia or neuronal necrosis (Brown, 1977). Possibly related to the severe episodes of apnea and cyanosis. To our knowledge, this is the first case report describing a litter of Old English Sheepdogs with a neurological presentation like Startle Disease.

## 52 (P 25)

### 52.1 Dystonic Head Tremor as a Part of the Semiology of Paroxysmal Dyskinesia in Dogs: 17 Cases (2021–2023)

#### 52.1.1 T. Liatis, DVM, ECVN Resident,^1^ S. De Decker, DVM, PhD, DipECVN^1^

##### 52.1.1.1 ^1^ Royal Veterinary College, University of London, Hatfield, UK

Dystonic head tremor (DHT) has not been reported in dogs. The aim of this study was to identify the incidence of DHT in dogs diagnosed with paroxysmal non‐kinesigenic dyskinesia (PD) and describe its clinical features. A retrospective study between 2021 and 2023 included dogs diagnosed with PD, available video footage, and head tremor. Ethical approval was not required. Seventeen of 104 (43.6%) dogs diagnosed with PD manifested DHT. Poodle‐crosses were the most common breed (7/17). Dystonic head tremors were described as fine irregular head tremors accompanied by cervical dystonia (15/17), truncal (11/17) or head (10/17) sway, shifting limb (10/17) or single limb (6/17) dystonia, freezing (8/17), ataxia (6/17), ptyalism (5/17), falling (5/17), kyphosis (4/17), and prayer posture (4/17). Neurological examination and advanced imaging when available were within normal limits. Dystonic tremor can appear in any part of the body affected by dystonia, however, is most commonly seen as dystonic head tremor (DHT) in humans with the movement disorder of cervical dystonia. In dogs, although dystonia is a common component of the movement disorder paroxysmal dyskinesia (PD), tremor has also been reported within its clinical spectrum. This study confirms that dystonic head tremor exists in dogs with PD at a frequency rate (43.6%); it has characteristic features and should be considered in differential diagnoses in dogs with head tremors.

## 53 (P 26)

### 53.1 Evaluation of the Effects of Subarachnoid Iodinated Contrast Injection on Neurological Examination in Dogs Presumptively Diagnosed With Meningomyelitis of Unknown Origin

#### 53.1.1 I. Martínez García, GV, MRCVS,^1^ A. Farré Mariné, LV, DipECVN,^1^ A. Luján Feliu‐Pascual, LV, MRCVS, DipECVN^1^ ^1^ AÚNA Especialidades Veterinarias, Paterna (Valencia), Spain

A transitory neurological worsening due to chemical myelitis has been listed as one of the complications of subarachnoid iodinated contrast injection (SICI). Furthermore, the use of myelography has been historically contraindicated in patients with suspected meningomyelitis since it might lead to a fatal outcome. To the authors' knowledge, there are no scientific studies supporting this belief. The aim of the study was to prospectively evaluate the effects of SICI on neurological examination in patients presumptively diagnosed with meningomyelitis of unknown origin (MUO).

The study was reviewed by the Comité de Ética de Experimentación Animal (CEEA) CEU University. Dogs and cats presumptively diagnosed with MUO between January 2022 and January 2023 that received SICI 0.3 mL/kg as part of the diagnostic protocol for progressive myelopathy were included (Group A). Diagnostic criteria have been previously described: focal or multifocal lesions affecting the CNS during advance imaging, mononuclear pleocytosis, and negative PCR in CSF for infectious agents. Twenty‐one dogs and one cat matched the inclusion criteria. For comparative purposes, two control groups of patients evaluated during the same period were created: Group B included dogs and cats with the same SICI administration and diagnosed with spinal pathologies other than MUO (*n* = 155) and Group C included dogs diagnosed with MUO using intravenous iodinated contrast (*n* = 29). A total of 206 dogs and cats were evaluated before and 2 h after SICI. Twenty‐nine patients showed transient neurological worsening: 1 dog of Group A (1/22 = 4.5%) and 28 (26 dogs and 2 cats) of Group B (28/155 = 18%). Based on these results, there is not enough evidence to contraindicate the use of SICI in patients with suspected MUO.

## 54 (P 27)

### 54.1 Tranexamic Acid in Reducing Intraoperative Bleeding in Dogs Undergoing Thoracolumbar and Lumbar Hemilaminectomy and Intervertebral Disc Fenestration

#### 54.1.1 D. Ferrarin, BSc, MSc, PhD,^1^ M. Wrzesinski, BSc, MSc,^1^ M. Schwab, BSc, MSc, PhD,^1^ D. Beckmann, BSc, MSc, PhD,^2^ A. Ripplinger, BSc, MSc, PhD,^1^ J. Rauber, BSc, MSc,^1^ J. Chaves, BSc, MSc,^1^ A. Mazzanti, BSc, MSc, PhD^2^

##### 54.1.1.1 ^1^ Graduate Program in Veterinary Medicine, Serviço de Neurologia e Neurocirurgia Veterinária, Universidade Federal de Santa Maria, Santa Maria, Rio Grande do Sul, Brazil; ^2^ Department of Small Animal Clinic and Surgery, Serviço de Neurologia e Neurocirurgia Veterinária, Universidade Federal de Santa Maria, Santa Maria, Rio Grande do Sul, Brazil

Hemilaminectomy associated with intervertebral disc fenestration (HF) is the most used spinal decompression surgical technique by surgeons for the treatment of intervertebral disc extrusion (IVDE). The surgical procedure can be hampered by excessive bleeding from the venous sinuses; however, tranexamic acid (TXA) intravenously (IV) is a possible adjunct to hemostasis in these patients. This study aimed to verify the effectiveness of tranexamic acid in reducing intraoperative bleeding in dogs with thoracolumbar and lumbar IVDE submitted to HF. Sixteen dogs with IVDE undergoing HF were included. These were distributed into a TXA group (tranexamic acid 20 mg/kg IV bolus, followed by 2 mg/kg/h IV continuous infusion) (*n* = 8) and a control group, with saline solution (*n* = 8). Blood loss was measured using the gravimetric method. The difficulty of operative visualization due to bleeding was classified by the surgeon. Median blood loss (%) in patients in the TXA group was lower than those in the control group (2.75 ± 1.23 and 4.99 ± 4.44, respectively) (*p* = 0.028). Intraoperative visualization difficulty due to bleeding occurred in 10 patients in the control group and no patients in the TXA group. A severe arterial thromboembolic complication was recorded, potentially due to tranexamic acid. The use of intraoperative tranexamic acid was effective in reducing bleeding and facilitating operative visualization in dogs with IVDE undergoing hemilaminectomy and intervertebral disc fenestration.

## 55 (P 28)

### 55.1 Dropped Head Syndrome: An Unusual Presentation of Suspected Focal Myasthenia Gravis in Two Dogs

#### 55.1.1 A. Gardini, DVM, PhD, DipECVN, EBVS, ^1^ G. Galli, DVM,^1^ B. Bravaccini, DVM,^1^ M. Menchetti, DVM, PhD, DipECVN, EBVS^1^

##### 55.1.1.1 ^1^ Neurology and Neurosurgery Division, San Marco Veterinary Hospital, Veggiano (PD), Italy

In human medicine “dropped head syndrome” (DHS) is a condition defined by weakness of the cervical paraspinal muscles resulting in flexion of the head and cervical spine. DHS has been described associated to different pathologies such as myasthenia gravis (MG) and polymyositis.

Two dogs, an 8‐year‐old Basset Hound and a 3‐year‐old Zwergpincher, were referred because they held neck and head in a dropped position. Regurgitation and voice changes were not reported.

After mild exercise, the Bassett Hound developed progressive severe neck weakness. The Zwergpincher showed bilaterally decreased palpebral reflex and mild neck ventroflexion, which severely worsened after exercise. In both, the neck ventroflexion was so marked that forced them to rest their neck on the floor before being able to raise their head and walk again. In the Zwergpincher, chest X‐rays showed severe megaesophagus; in both cases, blood tests, infectious disease screening, computed tomography, electrodiagnostics, and acetylcholine‐receptor antibodies were within normal limits. After neostigmine administration, the Zwergpincher showed improvement in the neck weakness. Treatment with pyridostigmine led to complete clinical remission and normal neurological examination at 6 months follow‐up in both dogs. For these reasons, a focal MG associated with DHS as the main clinical sign was suspected in both patients. In humans, MG is the only condition known to cause DHS that can be reversed with medical therapy. Similarly, DHS was rapidly reversed following anticholinesterase treatment in our cases.

## 56 (P 29)

### 56.1 Removal of a Large Arachnoid Cyst Through Rostrotentorial and Left Suboccipital Craniotomy in an American Staffordshire Terrier

#### 56.1.1 A. Seisdedos Benzal, LV, MSc, PhD,^1^ A. Farré Mariné, LV, dipECVN,^1^ A. Luján Feliu‐Pascual, LV, MRCVS, dipECVN^1^

##### 56.1.1.1 ^1^ Neurology/Neurosurgery Service of AÚNA Especialidades Veterinarias Hospital IVCEVIDENSIA, Valencia, Spain

Intracranial arachnoid cysts (IAC) are developmental brain disorders characterized by cerebrospinal fluid (CSF) accumulation within a duplication of the arachnoid membrane. This rare condition is diagnosed in 0.7% of dogs undergoing an MRI scan due to intracranial signs. An 11‐month‐old male American Staffordshire terrier was referred with a few months' history of constant left thoracic limb and palpebral myoclonic movements. In addition, the neurological examination revealed delayed postural reactions in both pelvic limbs and reduced bilateral menace response localizing the lesion to the brainstem/cerebellum. A CT scan under general anesthesia revealed a large intraxial (3.74 × 3.74 × 3.71 cm) hypoattenuating cystic lesion in the caudal fossa causing severe compression of the caudal brainstem and cerebellum associated with obstructive hydrocephalus. A combined left rostrotentorial and suboccipital craniectomy was carried out to excise the cystic walls. The structure was divided into three different compartments and all adherences to the brain parenchyma were gently removed and submitted for histopathological analysis. The samples confirmed the presence of simple plane epithelium and a cyst wall related to the arachnoid. Three months later, mild cerebellar ataxia and mild palpebral myoclonus only during stressful situations remained. A follow‐up CT scan at the time revealed resolution of the cystic lesion and the hydrocephalus. After 7 months of follow‐up, the dog shows only facial tics sporadically.

Although IAC might represent an incidental finding, neurological signs can develop due to parenchyma compression. In the latter, excision as in our case, can improve the animal's neurological status in the long term.

## 57 (P 30)

### 57.1 Early‐Onset of Granuloprival Cerebellar Degeneration in a 4‐Months‐Old Yorkshire Terrier

#### 57.1.1 A. Recio Caride, DVM, Dip. ECVN,^1^ M. Pumarola, DVM, PhD, Dip ECVP^2^

##### 57.1.1.1 ^1^ Clínica Veterinaria Levante, San Javier (Murcia), Spain; ^2^ Department of Animal Medicine and Surgery UAB Barcelona, Barcelona, Spain

Cerebellar cortical degeneration in dogs has been described in many different breeds. In most cases, Purkinje cells are the most affected neurons. We describe a novel case of a granuloprival degeneration in a Yorkshire terrier puppy. A 4‐months‐old female Yorkshire terrier was referred for a chronic and progressive incoordination and tremor. Physical examination was normal. Neurological examination revealed an evident cerebellar ataxia, intention tremor, and absence of menace response, consistent with a diffuse cerebellar disease. Congenital, infectious, metabolic, and degenerative diseases was proposed as differential diagnosis. Ancillary tests were unremarkable. Magnetic resonance showed a widening of the cerebellar sulci as well as the fourth ventricle, more evident in sagittal images. Cerebrospinal fluid sampled did not show nucleated cells and the protein level was 22.3 mg/dL. Genetic tests for cerebellar degenerative diseases in other breeds were performed and were negative. Due to progressive worsening of the clinical signs, the dog was euthanized. Cerebellar histopathological findings showed symmetrical and extensive thinning of the cerebellar cortex, affecting mainly the molecular and granular layers. The main changes appeared in the granular layer, showing a generalized and massive loss of granular neurons associated with moderate gliosis, while the molecular layer neuropile was affected with multiple spongiotic foci. Purkinje layer, cerebellar nuclei as well as mesencephalic oculomotor and red nuclei showed pale neurons due to accumulation of pale granular material and associated diffuse gliosis. To our knowledge, this is the first case of a granuloprival cerebellar degeneration described in a Yorkshire terrier. Thus, cerebellar juvenile neurodegenerative disease, should be included in the differential diagnosis in this breed.

## 58 (P 31)

### 58.1 Lymphocytes Obtained From Dogs With Inflammatory Neurological Diseases Show Induction of the Transmembrane Receptor—TIRC7

#### 58.1.1 T. Löwe, veterinarian,^1^ S. Perret, veterinarian,^1^ R. Carlson, technician,^1^ J. Nessler, Dr. Med. Vet., Dipl. ECVN, EBVS,^1^ E. Packeiser, Dr. Med. Vet.,^1^ W. Baumgärtner, Prof., Dr. Med. Vet., PhD, Dipl. ECVP/ACVP,^3^ K. Hülskötter, PhD,^3^ N. Utku, PD, Dr. Med.,^2^ A. Tipold, Prof., Dr. Med. Vet., Dipl. ECVN^1^

##### 58.1.1.1 ^1^ Department of Small Animal Medicine and Surgery, University of Veterinary Medicine, Hanover, Germany; ^2^ Institute for Medical Immunology, Charité—Universitätsmedizin, Berlin, Germany; ^3^ Department of Pathology, University of Veterinary Medicine, Hanover, Germany

T‐cell immune response cDNA 7 (TIRC7) is a transmembrane receptor expressed predominantly in activated subsets of lymphocytes. The molecule contributes to regulation of lymphocytes via binding to its ligand HLADR‐alpha2, leading to activation of CTLA‐4 and inhibition of Th1 and Th17 responses. Targeting TIRC7 via antibodies selectively modulates immune responses in inflamed tissue without suppression of unrelated immune functions. The aim of the study was to elucidate TIRC7 expression in peripheral blood lymphocytes (PBL) in dogs with inflammatory diseases. PBL samples of 8 healthy beagles and 40 patients were stained intracellularly and examined by multicolor flow cytometry. The group of inflammatory diseases was divided into neurological inflammatory diseases, such as steroid‐responsive meningitis‐arteritis (SRMA) or meningoencephalitis of unknown origin (MUO), polyarthritis, and systemic inflammatory diseases. In addition, blood samples from patients with immunosuppressive therapy were evaluated. TIRC7 expression was induced in PBL obtained from dogs suffering from SRMA (median 601; range 250–3181) reflected by elevated mean fluorescence intensity (MFI) levels compared with dogs with MUO, polyarthritis (median 273; range 60–1094), healthy beagles (median 330; range 127–534), and systemic inflammatory diseases (median 540; range 52–1693). Glucocorticosteroid treatment resulted in reduced TIRC7 expression in PBL (median 486; range 175–731) compared with those without therapy (median 601; range 250–3181).

We report here for the first time increased TIRC7 expression in PBL from dogs suffering from different inflammatory diseases. Marked impact on TIRC7 expression was observed in SRMA cases and after glucocorticosteroid application.

## 59 (P 32)

### 59.1 Botulism in Pregnant Mares: Case Series

#### 59.1.1 M. Martin, LV, ^1^ J. Santana, DVM,^1^ A. Botting, BVSc,^1^ C. Embersics, DVM, ACVIM Residency Trained (Neurology),^1^ R. Baker, BVMS, MS, DACVIM (Equine Medicine)^1^

##### 59.1.1.1 ^1^ Carolina Veterinary Specialists, Matthews, North Carolina, USA

Botulism carries a survival rate of 48% in hospitalized adult horses.^1^ Scarce reports in pregnant women confirmed favorable outcomes for both mother and child,^2^ despite additional risks inherent to pregnancy, such as rapid progression to respiratory failure, difficult intubation,^3–5^ and preterm delivery.^4^ We aim to provide descriptive information on botulism in pregnant mares.

During a multi‐state epizootic of foodborne type‐C equine botulism, 5 pregnant mares were hospitalized at Carolina Veterinary Specialists due to muscle tremors and acute generalized weakness. Additional findings included decreased tongue, tail, and anal tone, and dilated pupils with slow pupillary light reflexes. All mares were prophylactically administered altrenogest and a double dose (1000 mL/mare) multi‐valent botulism anti‐toxin. Three mares developed sustained recumbency, which is strongly associated with poor prognosis,^1^ and were euthanized. Increased respiratory effort was appreciated in the recumbent mares. Two mares survived and delivered foals after normal gestational length, with appropriate strength for unassisted vaginal delivery. Of the two foals, one was born septic and the second was born healthy. In people, no evidence shows that botulinum toxin can cross the placenta. Both mares were able to stand and successfully nurse their foal, passed their placenta, and had normal uterine involution. Additionally, both underwent successful rebreeding. During hospitalization, respiratory status and strength to show signs of normal parturition should be closely monitored. Immediate assistance and/or induced delivery may be necessary. Additionally, the administration of a high dose of multi‐valent botulism antitoxin does not appear to be associated with any complications with either mare or foal.

## 60 (P 33)

### 60.1 Aneurysmal Bone Cyst in the Cervical Vertebral Body of a Young Dog: Clinical, Computed Tomography, Magnetic Resonance Imaging, and Pathological Findings

#### 60.1.1 P. Menendez, DVM, CertAVP, MRCVS,^1^ G. Leeming, BVetMed, MPhil, PhD, FHEA, FRCPath, MRCVS,^2^ S. Platt, BVM&S, Dip. ECVN, Dip. ACVIM (Neurology), FRCVS,^3^ P. Richards, BVSc, PhD, MRCVS,^2^ E. Alcoverro, DVM, Dip. ECVN, MRCVS^1^

##### 60.1.1.1 ^1^ Chestergates Veterinary Specialists, Chester, UK; ^2^ University of Liverpool, Neston, UK; ^3^ VetOracle Teleradiology, Norfolk, UK

A 6‐month‐old male Whippet was presented for investigation of neck pain and lethargy. Physical examination was within normal limits. On neurological examination, low head‐carriage, severe cervical hyperalgesia, and markedly stiff gait were detected. No signs of ataxia or paresis were noted. The postural reactions and spinal reflexes were intact in all limbs. On computed tomography (CT) examination, a lytic expansile lesion centered on the right side of the body of the fourth cervical vertebra (C4) with associated spinal cord compression was observed. The owner opted for euthanasia due to the lack of response to multimodal analgesia. Permission was obtained for post‐mortem magnetic resonance imaging (MRI) and post‐mortem examination. Post‐mortem MRI confirmed the presence of an infiltrative lesion involving the right side of the C4 vertebral body accompanied by severe compression of the spinal cord. On necropsy, a focal, severe, chronic proliferative lytic lesion with extension in the vertebral canal, resulting in right ventro‐lateral, focal, and severe spinal cord compression was detected. The findings were compatible with an aneurysmal bone cyst affecting the vertebral body of C4.

To the best of our knowledge, this is the first case report of a histopathologically‐confirmed aneurysmal bone cyst affecting the cervical vertebral column in a young dog with description of CT and MRI findings.

## 61 (P 34)

### 61.1 Case Report of a Dog With Tethered Cord Syndrome and Dilatation of the Conus Medullaris due to CSF Accumulation Managed With Internal and External Filum Terminale Detethering

#### 61.1.1 C. Toujas, DVM, MRCVS,^2^ D. E. Whittaker, BVetMed, PhD, MRCVS,^1^ C. Black, Mag. Med. Vet., MRCVS^1^

##### 61.1.1.1 ^1^ Fitzpatrick Referrals LTD, Eashing, Surrey, UK; ^2^ Animed Whitstable, Whitstable, Kent, UK

Tethered cord syndrome (TCS) is a rare cause of pain, orthopedic signs, and neurological deficits in dogs. It is most frequently associated with inelastic filum terminalis leading to excessive tension on the conus medullaris and spinal cord. A static position of the conus medullaris on dynamic MRI is suggestive of TCS. Reports in dogs are scarce and postoperative imaging not reported in those treated surgically. A 5‐year‐old female neutered Saluki presented with an 11‐month history of bilateral pelvic limb lameness and difficulty rising with no response to medical management. Bilateral pelvic limb lameness and lumbosacral (LS) hyperaesthesia were detected. Dynamic MRI of the LS spine confirmed absence of cranio‐caudal movement of the conus medullaris with moderate distension of the subarachnoid space. Surgical detethering and durotomy was performed; a L6/7 dorsal laminectomy and durotomy was performed and CSF‐like fluid drained. The internal and external filum terminale were transected. The fluid was CSF and analysis normal. The dog improved clinically and dynamic MRI 11‐weeks postoperatively identified failure of the surgery to restore cranio‐caudal movement of the conus medullaris despite occupying a more cranial position. Distension of the subarachnoid space had improved. Reaching a diagnosis of TCS is challenging due to lack of data about the physiological position and conformation of the conus medullaris. Failure of the surgery to lead to MRI detectable movement of the conus medullaris on dynamic sequences despite successful resection of the filum may suggest other tissue contributes to the tethering effect in dogs. Despite this the dog improved.

## 62 (P 35)

### 62.1 Perineural Ultrasound Guided Steroid Infiltration in Grade 1 Foraminal Intervertebral Disc Extrusion in Dogs: A Single Center Experience (2020–2023)

#### 62.1.1 L. Golini, Dr. Med. Vet., Msc, Dipl ECVN, MRCVS,^1^ A. Vigani, Dr. Med. Vet., PhD, Dipl ACVAA, Dipl ACVECC, Dipl ECVECC^2^

##### 62.1.1.1 ^1^ Section Neurology and Neurosurgery, Tierspital VetSuisse Faculty, University of Zurich, Zurich, Switzerland; ^2^ Section Emergency and Intensive Care, Tierspital VetSuisse Faculty, University of Zurich, Zurich, Switzerland

Introduction/Purpose: Perineural steroid infiltration is an effective treatment for pain syndrome due to IVDD and neuritis in humans. In veterinary medicine there is still lack of evidence and clinical experience.

Methods: Retrospective single center study. Dogs with single foraminal disc extrusion diagnosed on MRI who received perineural ultrasound‐guided steroid infiltrations. Dogs should only have pain and/or neurogenic lameness without any other neurological deficits on presentation.

Results: Eleven dogs have been identified. Nine dogs were diagnosed with a cervical an 2 with a lumbar foraminal disc extrusion. Three consecutive infiltrations, 1 month apart, were performed in all dogs.

On presentation, pain affected 11 dogs and neurogenic lameness 6. All dogs were discharged with rescue analgesia following the first perineural steroid infiltration. One month later, at the time of the second infiltration, 6 dogs had a partial response and 5 were pain and medication free. At the third infiltration, 10 dogs were pain free and only one dog still required rescue analgesia, which then required decompressive surgery.

At the 6 months recheck, 9 dogs were pain and medication free and 1 was showing sporadic episodes of pain.

Discussion/Conclusions: In our experience ultrasound guided perineural steroid infiltration for Grade 1 foraminal intervertebral disc extrusions is a viable therapeutic option.

## 63 (P 36)

### 63.1 Post‐Hemilaminectomy Analgesia With Intramuscular Methadone or Transdermal Fentanyl in Dogs

#### 63.1.1 D. Beckmann, BSc, MSc, PhD,^2^ L. Herculano, BSc, MSc,^1^ M. Schwab, BSc, MSc, PhD,^1^ D. Ferrarin, BSc, MSc, PhD,^1^ A. Ripplinger, BSc, MSc, PhD,^1^ M. Wrzesinski, BSc, MSc,^1^ C. Vacarin, BSc, MSc,^1^ J. Rauber, BSc, MSc,^1^ A. Mazzanti, BSc, MSc, PhD^2^

##### 63.1.1.1 ^1^ Graduate Program in Veterinary Medicine, Veterinary Neurology and Neurosurgery Service, Federal University of Santa Maria, Santa Maria, Rio Grande do Sul, Brazil; ^2^ Department of Small Animal Clinic and Surgery, Veterinary Neurology and Neurosurgery Service, Federal University of Santa Maria, Santa Maria, Rio Grande do Sul, Brazil

In spinal decompressive surgeries such as hemilaminectomy, the postoperative pain degree is considered intense, and there is growing interest in establishing adequate standards for analgesia in this period in dogs. This study aimed to evaluate analgesia using two μ receptor agonist opioids by two different routes of administration, transdermal for fentanyl (FT) and intramuscular for methadone (MI). Eight dogs from the hospital routine underwent thoracolumbar hemilaminectomy and intervertebral disc fenestration surgery to treat intervertebral disc extrusion. They were randomly assigned to two groups of equal number, transdermal fentanyl group (FT) and methadone group (MI). At the end of surgery, a fentanyl patch was applied to the animals in the FT group, remaining there for 72 h. In the MI group, post‐surgical analgesia was performed with intramuscular applications of methadone at intervals of 6 h until 72 h postoperatively. The animals were evaluated by the simplified form of the Glasgow Composite Pain Scale (SF‐GCPS) every 2 h in the first 24 h, every 4 h until completing 48 h, and then a last evaluation at 72 h postoperatively. The inter‐rater agreement for the SF‐GCPS was weak (0.32), and the linear correlation test was moderate (0.53). In the MI group there was need for three analgesic rescues, while in the FT group there was no rescue. In conclusion, both fentanyl and methadone provided satisfactory analgesia in the first three postoperative days in dogs undergoing thoracolumbar hemilaminectomy. Better stability in pain scores in the postoperative period was observed for animals treated with FT, as well as less occurrence of side effects in this group.

## 64 (P 37)

### 64.1 Radiofrequency‐Induced Thermal Burn Injury in a Dog After Magnetic Resonance Imaging

#### 64.1.1 E. Lichtenauer, DVM, Resident ECVN,^1^ K. Santifort, DVM, Resident ECVN,^1^ ^,2^ M. Beukers, DVM, Diplomat ECVDI,^1^ I. Carrera, DVM, MVM, PhD, Diplomat ECVDI,^3^ I. Van Soens, DVM, PhD, Diplomat ECVN,^1^ N. Bergknut, DVM, PhD, Diplomat ECVN^1^

##### 64.1.1.1 ^1^ Evidensia Dierenziekenhuis Hart Van Brabant, Waalwijk, The Netherlands; ^2^ Evidensia Dierenziekenhuis Arnhem, Arnhem, The Netherlands; ^3^ VetOracle Teleradiology, Norfolk, UK

Thermal burn injuries caused by radiofrequency (RF) pulses (“RF burns”) can occur when there is contact between skin and a conductive object or direct skin‐to‐skin contact. There are several mechanisms for the occurrence of RF burns, such as inductive heating, heating of a resonant loop and the “antenna effect.” RF burns are the most reported complication in humans undergoing MRI but have not been reported in veterinary patients. A 10‐year‐old male Shar Pei was referred for lethargy and proprioceptive deficits of the left thoracic limb. An MRI study of the brain and cervical spinal column was performed. The dog was placed in sternal recumbency with the thoracic limbs positioned caudally without touching the trunk. A left‐sided C3–C4 intervertebral disc extrusion with spinal cord compression was diagnosed. Medical treatment was elected. Within a week after the MRI study, the dog presented with deep partial‐thickness skin burn wounds in both axillae. Since the specific absorption rate had not exceeded the safety limits during any of the scans and there was no inductive heating or heating of a resonant loop via limbs contacting the trunk, the burns were diagnosed as RF burns caused by direct skin‐to‐skin contact during MRI. The excessive skin folds in the axillae of this Shar Pei likely facilitated the occurrence of the RF burns. The wounds healed by secondary intent over the next month. Clinicians and technicians must be aware of the risk for RF burns in veterinary patients and take precautions regarding positioning of the patient.

## 65 (P 38)

### 65.1 Eosinophilic Pleocytosis With Microfilariae Secondary to Aberrant Dirofilaria Immitis Migrans

#### 65.1.1 M. Martin, LV,^1^ M. Sachse, DVM,^1^ K. Metcalf, DVM,^1^ S. Dehghanpir, DVM MS DACVP,^1^ C. Embersics, DVM ACVIM (Neurology) Residency trained^1^

##### 65.1.1.1 ^1^ Department of Veterinary Clinical Sciences, LSU School of Veterinary Medicine, Louisiana State University, Baton Rouge, Louisiana, USA

Eosinophilic pleocytosis is classically associated with migrating helminth infection. Aberrant migration of adult‐stage heartworms causing compressive myelopathy in dogs was previously characterized on MRI. We report a case of eosinophilic pleocytosis with microfilariae secondary to aberrant Dirofilaria immitis migrans.

A 1‐year‐old female Golden Retriever was evaluated for a 1‐week history of ambulatory tetraparesis and apparent cervical pain. Proprioceptive placing of all limbs was absent. CBC and biochemistry panel revealed no abnormalities, with exception of microfilaremia. The dog was positive for circulating Dirofilaria immitis antigen (SNAP4Dx). MRI of the cervical spinal cord revealed no abnormalities. Cerebrospinal fluid showed a total nucleated cell count of 2643/μL (RI < 5/μL), total erythrocyte count of 3/μL (RI < 5/μL), and total protein concentration of 152 mg/dL (RI < 30 mg/dL). Cytopathologic evaluation revealed marked eosinophilic pleocytosis with microfilariae. Treatment included ivermectin, doxycycline, and anti‐inflammatory dose of prednisone, after which the clinical signs resolved. Additionally, adulticide treatment with melarsomine was initiated. Although MRI was unremarkable, clinical signs were attributed to local inflammation and vascular damage caused by parasitic migrans. Efficacy of microfilaricide treatment in the CSF is unknown. There is a risk of the patient remaining as a reservoir of infection if therapeutic concentration of ivermectin is not achieved despite blood–brain barrier disruption. In other species, eosinophilic pleocytosis is more commonly associated with a parasitic or fungal etiology, whereas in dogs, idiopathic eosinophilic meningoencephalitis appears to be more prevalent, raising the concern that parasitic causes might be underdiagnosed. Therefore, dirofilariasis should be considered in dogs with eosinophilic pleocytosis.

## 66 (P 39)

### 66.1 Canine Chronic Idiopathic Pyogranulomatous Pachymeningitis. Diagnosis, Management, and Clinical Outcome

#### 66.1.1 C. Santos Hernandez, GV, GPCert (Neuro),^1^ C. I. Serra Aguado, LV, PhD, Res. ECVS,^1^ C. Naranjo Freixa, LV, PhD, Dipl. ACVP, Dipl. ECVP.,^2^ M. Pérez, LV, CCRP, ACVIM (Neurology) eligible^3^

##### 66.1.1.1 ^1^ Universidad Católica de Valencia, Valencia, Spain; ^2^ IDEXX Laboratories S.L., Barcelona, Spain; ^3^ Hospital Veterinario Fénix, Elche, Spain

This case report aimed to describe the case of a dog with chronic idiopathic pyogranulomatous pachymeningitis (CIPP). A 7‐year‐old female neutered Labrador Retriever was presented for investigation of progressive non‐ambulatory tetraparesis. On presentation, neurological examination also revealed absent postural reactions in thoracic limbs and decreased in pelvic limbs with normal spinal reflexes in all four limbs. Cranial nerve exam was unremarkable. Full‐body computed tomography (CT) scan showed a 2.6 × 0.87 cm compressive extradural contrast‐enhanced lesion dorsally to the spinal cord at the level of C3. Cerebellomedullary cerebrospinal fluid (CSF) analysis showed a mild neutrophilic pleocytosis with slight increase in protein levels. Dorsal laminectomy from the caudal aspect of C2 to the cranial aspect of C4 and durotomy along C3 were performed to excise the mass. Biopsy was consistent with CIPP and associated fibrosis. Culture and PCR for *Leishmania* spp., *Toxoplasma* spp., *Neospora* spp., and *Ehrlichia* spp. were negative, as well as immunohystochemistry (IHC) for *Leishmania* spp. Methylprednisolone 0.5 mg/kg twice daily with tapper dose and gabapentin 10 mg/kg three times a day were initiated. After 3 months, the dog showed an improved neurological exam with ambulatory tetraparesis. Follow‐up CT scan was performed at this time, revealing a minimal displacement of the spinal cord and CSF analysis was within normal limits. Six months after surgery, the dog was on methylprednisolone 0.25 mg/kg every 3 days, showing only mild proprioceptive ataxia. CIPP is a rare inflammatory disorder in small animals. It can be diagnosed with CT and biopsy. Surgical decompression and immunosuppressant therapy were effective to control the disease in this case.

## 67 (P 40)

### 67.1 Diffusion Tensor‐Based Analysis of White Matter in Canine Idiopathic Epilepsy

#### 67.1.1 K. Beckmann, Dr. Med Vet., Dipl ECVN,^1^ I. Carrera, Phd, Dipl ECVDI,^2^ A. Wang‐Leandro, PhD, Dipl ECDVI,^3^ ^,4^ S. Haller, Prof. Dr.^5,6^

##### 67.1.1.1 ^1^ Section of Neurology, Department of Small Animals, Vetsuisse Faculty Zurich, University of Zurich, Zurich, Switzerland; ^2^ Vet Oracle Teleradiology, Norfolk, UK; ^3^ Clinic for Diagnostic Imaging, Department of Diagnostics and Clinical Services, VetsuisseFaculty, University of Zurich, Zurich, Switzerland; ^4^ Department of Diagnostic Imaging, Clinic for Small Animals, University of Veterinary Medicine Hannover, Hannover, Germany; ^5^ University of Geneva, Faculty of Medicine, Geneva, Switzerland; ^6^ Uppsala University, Department of Surgical Sciences, Radiology, Uppsala, Sweden

The understanding of pathogenesis of epileptic seizures has evolved over time and it is now generally accepted that not only cortical and subcortical areas are involved, but also the connection of these regions in the white matter. Recent neuroimaging studies confirmed involvement of the white matter in several human epileptic syndromes. A large multicentric human study found lower fractional anisotropy in most fiber tracts, especially in the corpus callosum, cingulum and external capsule across all epileptic syndromes. Neuroimaging studies investigating the white matter integrity in canine idiopathic epilepsy are lacking. This studied aimed to test the hypothesis that white matter diffusion changes can be found in dogs suffering from generalized idiopathic epilepsy.

Twenty‐six dogs with idiopathic Epilepsy (15 Border Collies, 11 Greater Swiss Mountain dogs) and 24 healthy control dogs (11 Beagle dogs, 5 Border Collies, and 8 Greater Swiss Mountain dogs) were prospectively enrolled. Diffusion Tensor Imaging with 32 diffusion directions (low *b* value = 0 s/mm^2^; maximal *b* value = 800 s/mm^2^) was performed in a 3 T scanner. Tract Based Spatial Statistics (TBSS) approach was used to investigate changes in fractional anisotropy and mean diffusivity in epileptic dogs compared with healthy control dogs. This study failed to identify significant changes in fractional anisotropy and mean diffusivity between idiopathic epileptic dogs and healthy control dogs. Whether white matter is less involved in canine idiopathic epilepsy or if we simply have not been able to detect these differences remains open.

## 68 (P 41)

### 68.1 Acute Hydrated Nucleus Pulposus Extrusion in Dogs With Severe Neurological Status: Comparison Between Conservative and Surgical Treatment

#### 68.1.1 S. Pompilio, DVM,^1^ C. Toni, DVM, MRCVS, Dipl. ECVN,^2^ F. Cozzi, DVM, PhD, Dipl. ECVN,^3^ M. Menchetti, DVM, PhD, Dipl. ECVN,^4^ K. Marioni‐Henry, DVM, MRCVS, PhD, Dipl. ECVN, Dipl. ACVIM (Neurology), SFHEA,^5^ B. Contiero, MSc in Statistics,^6^ B. Betti, DVM,^1^ F. Tirrito, DVM, MRCVS, Dipl. ECVN^1^ ^,7^

##### 68.1.1.1 ^1^ AniCura Istituto Veterinario di Novara, Granozzo Con Monticello, Novara, Italy; ^2^ Neurology and Neurosurgery Service, Willows Veterinary Centre and Referral Centre, Solihull, West Midlands, UK; ^3^ Clinica Neurologica Veterinaria NVA, Milano, Italy; ^4^ Neurology and Neurosurgery Division, San Marco Veterinary Clinic, Veggiano, Padova, Italy; ^5^ Vets Now Glasgow Hospital, Glasgow, UK; ^6^ Department of Animal Medicine, Production and Health (MAPS), University of Padova, Legnaro, Padova, Italy; ^7^ Studio Veterinario Associato Vet2Vet di Ferri e Porporato, Torino, Italy

Dogs diagnosed with hydrated nucleus pulposus extrusion (HNPE) have a favorable outcome following either conservative treatment or decompressive surgery. However, the optimal treatment for dogs presenting severe neurological status remains unclear. This retrospective, multicentric study aimed to compare the outcome of non‐ambulatory paretic/plegic client‐owned dogs affected by HNPE, after conservative treatment or decompressive surgery. Eighty‐six dogs with magnetic resonance imaging diagnosis of HNPE were included. The neurological status was graded on presentation and during follow‐up (up to 6 months); the time required to gain ambulation (recovery time) was also recorded. Sixty (70%) dogs received conservative treatment (analgesia, cage rest, and physiotherapy), while 26 (30%) cases underwent surgical decompression (ventral slot, hemilaminectomy, or mini‐hemilaminectomy). HNPE was identified in the cervical and thoracolumbar regions in 83% and 17% of cases, respectively. The neurological status at 1 week was more favorable in the medical than surgical group (*p* = 0.014) however, after 4 weeks no significant differences were recorded (*p* = 0.501). Long‐term outcome was available in 26 dogs; full recovery was achieved in 83% and 75% of cases with medical and surgical treatment, respectively. Median recovery time was 7 days in the medical group and 9 days with surgery, without significant differences between treatments (*p* = 0.452). Factors associated with longer recovery time included non‐chondrodystrophic breed (*p* = 0.037), intramedullary hyperintensity (*p* < 0.001), and undergoing physiotherapy (*p* = 0.003). In addition, for every additional year of age, the recovery time increased by 14% (*p* = 0.007). In conclusion, conservative treatment is a viable option in cases of HNPE even in dogs with severe neurological status.

## 69 (P 42)

### 69.1 Idiopathic Oculomotor Neuropathy in a Cat: A Case Report

#### 69.1.1 F. Soares Marques de Almeida, MRCVS,^1^ V. Kumaratunga, BVSc, MRCVS,^1^ O. Marsh, BVM BVS MRCVS^2^

##### 69.1.1.1 ^1^ Southfields Veterinary Specialists, Basildon, UK; ^2^ Dick White Referrals, Cambridgeshire, UK

The aim of this report is to describe the clinical and imaging findings, and outcome, in a cat presumptively diagnosed with idiopathic oculomotor neuropathy.

A 12‐year‐old, male neutered, domestic shorthair cat was presented for evaluation of bilateral mydriasis, unresponsive to light, and photophobia. Ophthalmological examination confirmed internal ophthalmoparesis but identified no evidence of ocular disease. Neurological examination revealed bilateral mydriasis and absent pupillary light reflex and was otherwise normal. The neuroanatomical localization was to the parasympathetic portion of both oculomotor nerves or their nuclei. Diagnostic investigations, including hematology and biochemistry profiles, FIV and FeLV testing, MRI of the head, cerebrospinal fluid analysis, and bicavitary imaging, were all unremarkable. A presumptive diagnosis of idiopathic oculomotor neuropathy was therefore reached. No treatment was initiated, and the clinical signs remained unchanged with a 4‐month follow‐up. The patient was considered to be enjoying a good quality of life. Oculomotor dysfunction in cats has been previously reported to be frequently associated with intracranial neoplasia, and therefore with a guarded prognosis. In contrast, both dogs and humans can be affected by idiopathic oculomotor neuropathy, which has a good prognosis, without significant adverse effects on the patient's quality of life. This case report demonstrates that idiopathic oculomotor neuropathy can occur in cats, and should be considered as a differential diagnosis, especially in patients presenting with oculomotor deficits as the only abnormality. In this case, the bilateral mydriasis remained stable over several months and did not appear to negatively impact on the cat's quality of life.

## 70 (P 43)

### 70.1 Contralateral Medial Strabismus (Esotropia) Associated With Paramedian Thalamic Ischemic Infarct in Two Dogs

#### 70.1.1 T. Liatis, DVM ECVN Resident,^1^ D. Whittaker, DVM DipECVN^1^

##### 70.1.1.1 ^1^ Royal Veterinary College, University of London, Hatfield, UK

Pseudoabducens palsy resulting in contralateral esotropia (medial strabismus) is a rare consequence of a paramedian thalamic infarct in people. To date, esotropia has been reported in dogs in association with ipsilateral abducens neuropathy or extraocular myopathy, but not secondary to thalamic lesions. A 7‐year‐old male neutered Border Collie (dog 1) and a 12‐year‐old female neutered crossbreed (dog 2) were presented with peracute non‐progressive vestibular ataxia. Neurological examination of dog 1 identified right esotropia with non‐ambulatory tetraparesis, right head tilt, right vestibular ataxia, right Horner's syndrome, and vertical nystagmus. Neurological examination of dog 2 revealed right esotropia with non‐ambulatory tetraparesis and right postural reaction deficits, right head tilt, right vestibular ataxia, rotatory nystagmus, and absence of vestibulo‐ocular reflex to the right. Both dogs were localized to right central vestibular system with thalamic involvement. Hematology, biochemistry, urinalysis, and blood pressure were unremarkable. MRI of the brain revealed a left paramedian thalamic lacunar ischemic infarct. Computed tomography of thorax and abdomen were unremarkable (dog 1). Thyroid profile and viscoelastic coagulation monitoring were unremarkable, while multiple electrode aggregometry analysis showed increased platelet activation suggestive of thrombosis (dog 1). Antithrombin III was normal (dog 2). Pseudoabducens paralysis is suspected to occur due to interruption of descending inhibitory pathways that traverse the paramedian thalamus, decussate in the subthalamic region and innervate the contralateral third oculomotor nucleus, which results in tonic activation of the medial rectus. This study suggests that contralateral esotropia is a rare but highly localizing sign in dogs with thalamic infarcts.

## 71 (P 44)

### 71.1 Investigation on Canine MUO at Veterinary Referral Care in England (2017–2021): A Multicentric Retrospective Cohort Study

#### 71.1.1 Y. Choi, PhD,^1^ T. Harcourt‐Brown, MA, VetMB, CertVDI, DipECVN, CertVDI, FRCVS,^1^ E. Ives, MA, VetMB, DipECVN, MRCVS,^2^ B. Lopes, DVM, MSc,^2^ L. C. Alves, DVM, PhD, AFHEA, DipECVN, MRCVS,^3^ T. Cardy, BSc, BVetMed (Hons), MVetMed, PhD, DipECVN, MRCV,^4^ M. Ruggeri, DVM, MRCVS,^5^ A. Tauro, DVM, GPCert (Neuro), DipECVN, MRCVS,^5^ G. B. Cherubini, DVM, DECVN, FRCVS,^6^ M. Lowrie, MA, VetMB, MVM, DipECVN, MRCVS,^7^ L. Saunders, BSc,^8^ M. Stefaniuk, DVM, MRCVS,^9^ R. Cappello, PhD, DipECVN, MRCVS,^9^ R. Trevail, DipECVN, MRCVS,^10^ S. Adamantos, BVSc, CertVA, DACVECC, DipECVECC, MRCVS, FHEA, ^11,12^ R. Goncalves, DVM, DipECVN, MVM, FHEA, MRCVS, S. DeDecker, DVM, PhD, MvetMed, DipECVN, FHEA, PGCert Veted, MRCVS,^13^ A. Fadda, DVM, Dr. Med. Vet., DECVN, PhD, MRCVS^1^

##### 71.1.1.1 ^1^ Bristol Veterinary School, University of Bristol, Langford, UK; ^2^ Anderson Moores Veterinary Specialists, Winchester, UK; ^3^ Department of Veterinary Medicine, University of Cambridge, Cambridge, UK; ^4^ Cave Veterinary Specialists, Wellington, UK; ^5^ ChesterGates Veterinary Specialists, Neurology Service, Chester, UK; ^6^ Veterinary Teaching Hospital “Mario Modenato,” Department of Veterinary Sciences, University of Pisa, Pisa, Italy; ^7^ Movement Veterinary Referrals, Preston Brook, UK; ^8^ Highcroft Veterinary Referrals, CVS, Bristol, UK; ^9^ North Downs Specialist Referrals, Bletchingley, UK; ^10^ Southern Counties Veterinary Specialists, Ringwood, UK; ^11^ Paragon Veterinary Referrals, West Yorkshire, UK; ^12^ University of Liverpool School of Veterinary Science, Liverpool, UK; ^13^ Royal Veterinary College, London, UK

Although a considerable body of research exists on canine meningoencephalitis of unknown origin (MUO), many questions remain unanswered. For example, the number of cases diagnosed per year and the diagnostic criteria most frequently used in practice are unknown, but essential for both funding research on MUO and design of clinical therapeutic trials. This study evaluated the incidence of MUO in dogs referred to specialistic veterinary care across 13 centers in England between 2017 and 2021 and methods used for diagnosis. Additionally, information on signalment was compared with existing literature. Full medical records from MUO cases were available for review at 6 centers (574 cases), 7 centers provided summary data of MUO cases (583). MUO represented 2.3% (95% CI: 2.15–2.41) of new neurological referrals (1157/50 721), with a mean of 232 cases/year. Diagnosis peaked at 4 years‐of‐age, with French Bulldogs (12%) and Chihuahuas (11%) the most common breeds affected. No sex or neuter status was more affected. Both MRI and CSF analysis were performed in 1060/1157 cases (91.6%). Upon direct records' review of 574 cases, MRI and CSF results indicative of MUO were identified in 81% of the cases, abnormal MRI but normal CSF in 9%, normal MRI and abnormal CSF in 6%, and normal MRI and CSF in 3%. This information may aid estimation of disease incidence for research funding and future studies.

## 72 (P 45)

### 72.1 Central Cord Syndrome in a Cat With Bilateral Brachial Plexus T‐Cell Lymphoma

#### 72.1.1 H. Gunovska, MRCVS,^1^ S. R. Platt, Professor, BVM & DipACVIM (Neurology), DipECVN, FRCVS,^3^ H. Ferreira, DVM, FRCPath, Diplomate, ACVP, MRCVS,^2^ A. Bach, MRCVS,^4^ E. Alcoverro, DipECVN, MRCVS^5^

##### 72.1.1.1 ^1^ Dovecote Veterinary Hospital, Castle Donington, UK; ^2^ Axiom Laboratories, Devon, UK; ^3^ Vet Oracle, Norfolk, UK ^4^ Private Practitioner, UK; ^5^ Chestergates Veterinary Specialists, Chester, UK

Central cord syndrome (CCS) defines a clinical presentation of patients with more severe paresis in the thoracic limbs than in the pelvic limbs. According to recent literature, CCS is observed in dogs and cats with C1–C5 or C6–T2 myelopathies. A 9‐year‐old male neutered domestic short hair cat was referred for progressive bilateral thoracic limb dysfunction. Neurological examination revealed non‐ambulatory gait due to bilateral thoracic limb paresis, with markedly delayed postural reactions and absent withdrawal reflexes. The pelvic limbs were normal. Bilateral Horner syndrome was detected. A C6–T2 myelopathy or a bilateral brachial plexus neuropathy was suspected. Magnetic resonance imaging (MRI) of the cervico‐thoracic spine and brachial plexi revealed T2W hyperintense, T1W iso‐ to mildly hyperintense and strongly contrast‐enhancing, ill‐defined, irregular, bilateral and symmetrical enlargement of the C8, T1, and T2 nerves extending from their foramina into the brachial plexus. Cerebrospinal fluid (CSF) analysis revealed lymphocytic pleocytosis. Cytological examination of the CSF and of the brachial plexus (obtained via ultrasound‐guided fine needle aspirate) revealed lymphoblasts with malignant features. Polymerase chain reaction for antigen receptor rearrangement (PARR) on CSF indicated presence of a clonal T lymphocyte population. A T‐cell lymphoma bilaterally affecting the brachial plexus was diagnosed. The owner decided for euthanasia without further treatment. There is limited information about CCS in small animals. Published literature only describes cases of CCS with lesions affecting the cervical spinal cord segments. This case indicates that bilateral brachial plexus lesions should also be considered in cases of CCS.

## 73 (P 46)

### 73.1 Volumes of Intracranial Lateral Ventricles: Comparison Between French Bulldog and Dachshund

#### 73.1.1 J. Dietzel, DVM,^1^ S. Kohl, DVM,^1^ C. Köhler, DVM,^1^ T. Siegel, DVM,^1^ T. Flegel, Diplomate ECVN, Diplomate ACVIM (Neurology)^1^

##### 73.1.1.1 ^1^ Department for Small Animals, Faculty of Veterinary Medicine, University of Leipzig, Leipzig, Germany

Subjective ventriculomegaly is frequently observed on MRI in French bulldogs (FB). We measured the volume of the lateral ventricles (LV) of neurologically unremarkable FB and compared it to those of dachshunds (DH). The influence of the signalment was investigated and ventricular asymmetry assessed. Threshold for subjective detection of asymmetric ventricles was calculated. Exactly 40 FB and 15 DH were included and classified as without intracranial pathology based on an owner questionnaire and a complete neurological examination. Transversal, T1‐weighted MRI brain images (slice thickness = 2.0 mm) were obtained. Incidental findings on diagnostic imaging which could influence the volume of LV lead to exclusion. LV were manually segmented, quantitative volumetric measurements were obtained and assessment of LV asymmetry was done using the software ITK‐Snap. Total median LV volume of the FB was 785.7 and 1141.0 mm^3^ in DH. There was no statistic significant difference of LV volume between both breeds. Age, sex, neutering status and body weight had no significant influence on LV volume. A total of 50% of FB and 60% of the DH had a LV asymmetry. The left LV was larger than the right in 50% of FB and 66.7% of DH (no statistical significance). A difference in volume of 22% between both LV was calculated as threshold for visual detection of asymmetries. The FB had lower volumes of the LV compared with the values of DH. Ventriculomegaly should not be considered physiological in FB, but LV asymmetry seems to be common in neurologically unremarkable FB.

## 74 (P 47)

### 74.1 Neurological Signs of Disseminated *Scedosporium* spp. Infection in a German Shepherd

#### 74.1.1 S. Moya, MSc,^1^ S. Rodenas, Dip. ECVN,^1^ N. Delgado, DVM,^1^ J. C. Cartagena, PhD,^1^ V. Moya, DVM,^2^ F. Fariñas, PhD^3^ ^,1^

##### 74.1.1.1 ^1^ Bluecare Partners Hospital, Mijas Costa, Spain; ^2^ Hospital De Dia Dr. Moya, Torremolinos, Spain; ^3^ Inmunestem Terapia Celular E Inmunologia, Malaga, Spain

Scedosporiosis in dogs has often been associated with localized infection or, in rare cases, disseminated infections. The aim of this paper is to describe the neurologic signs, MRI features and histopathological findings of a rare systemic and fatal fungal infection in a dog caused by *Scedosporium* spp. A 5‐year‐old female neutered German Shepherd was presented to Neurology service with fever and lethargy. Neurologic exam revealed obnubilation, non‐ambulatory tetraparesis, miosis, and absent oculocephalic reflex. Neurologic examination was consistent with a brainstem localization. Blood test showed high kidney values and hypertension was also present.

Intracranial MR revealed an intra‐axial lesion affecting the brainstem and cerebellum (hyperintense lesion on T2WI and FLAIR images, isointense on T1WI) There was no contrast enhancement. Inflammatory/infectious or vascular encephalopathy were the main differential diagnoses. Postmortem examination revealed the presence of a large infarcted area with hemorrhagic necrosis in the brainstem. Multiple fungal pyogranulomas were observed upon histological examination with many vascular walls have been practically replaced by fungal structures with an intense pyogranulomatous inflammatory infiltrate. In addition, necrosis and inflammatory foci scattered throughout the kidney parenchyma and fibrino‐hemorrhagic necrosis in vessels were observed. Characteristics of the colonies in culture, the microscopic morphology and the identification done by molecular techniques allowed to identify this fungus within the genus Scedosporium (*Scedosporium* spp.). This case report shows that *Scedosporium* spp. may act as a primary pathogen in dogs and represents an important pathogen that should be considered during the diagnostic in neurological problems, particularly when a fungal infection is suspected.

## 75 (P48)

### 75.1 Suspected Acquired Paroxysmal Dyskinesia as Initial Manifestation of Meningoencephalitis of Unknown Origin in Two Dogs

#### 75.1.1 A. Pereira, DVM,^1^ S. Añor, PhD, Dipl. ECVN, Dipl. ACVIM (Neurology),^2,3^ C. de la Fuente, PhD, Dipl. ECVN,^1,2^ M. Reus, DVM,^1^ I. Montes de Oca, DVM^1^

##### 75.1.1.1 ^1^ Hospital Veterinari Montjuïc (Vetpartners), Barcelona, Spain; ^2^ Fundación Hospital Clínic Veterinari, Universitat Autònoma de Barcelona, Barcelona, Spain; ^3^ Departament de Medicina i Cirurgia Animals, Facultat de veterinària, Universitat Autònoma de Barcelona, Barcelona, Spain

Inherited or presumably inherited paroxysmal dyskinesias (PxD) are increasingly recognized in veterinary medicine but information about acquired PxD is scarce. A 5‐year‐old Miniature Schnauzer and a 3‐year‐old Maltese were referred for acute onset of clusters of abnormal movements (8 and 10 episodes < 24 h). The neurological examination was normal in both patients. The episodes were characterized by normal mentation, dyskinesia (flexion and extension of all limbs) and dystonia of the trunk (kyphotic posture) and tail. A dystonic head tremor was also noted in Case 2. During the time elapsed between the onset of clinical signs and the diagnostic protocol (48 h) both dogs kept suffering episodes and the second patient developed a mild cerebellar ataxia. Complete blood cell count, serum biochemistry, ionized calcium, thoracic radiographs, abdominal ultrasound and gluten sensitivity test were unremarkable. MRI (including DWI) showed meningeal contrast enhancement in both cases. CSF analysis demonstrated lymphocytic pleocytosis (case 1: 174 cells/μL, 88% lymphocytes; case 2: 110 cells/μL, 97% lymphocytes). Testing for infectious diseases and PARR were negative and a presumptive diagnosis of MUO was made. Treatment with corticosteroids and cytosine arabinoside resulted in a complete resolution of clinical signs. During the follow‐up period of 15 and 8 months, respectively no other episodes occurred. These are the first reported cases of canine PxD associated with MUO. The authors are aware of the lack of a cause–effect relationship but highlight that a diagnostic criteria tier system for PxD similar to that described for idiopathic epilepsy would be helpful.

## 76 (P49)

### 76.1 Nerve Root Signature Caused by Intervertebral Disc Disease in 83 Dogs

#### 76.1.1 E. Burbaite, DVM,^1^ M. Menchetti, DVM, PhD, Dipl. ECVN^1^

##### 76.1.1.1 ^1^Neurology and Neurosurgery Division, San Marco Veterinary Clinic, Veggiano, Italy

The nerve root signature (NRS) is a form of lameness usually caused by neurologic lesions, such as attenuation or inflammation of the nerve root/spinal nerve. However, it can be confused with a sign of orthopedic diseases, as NRS can be caused by both orthopedic and neurologic pathologies. Etiologies involve degenerative diseases such as arthropathies, intervertebral disc disease (IVDD), or neoplasia and inflammation. The purpose of this study is to analyze and present the features of NRS caused by IVDD in 83 dogs. A retrospective case collection was performed. Cases with neurologic NRS and Magnetic Resonance Imaging‐diagnosed IVDD were collected. Dogs with suspected or confirmed orthopedic diseases were excluded. In total, 162 cases were found; of those, 83 met the inclusion criteria. The most commonly affected spinal cord segment was cervicothoracic (C6–T2) (45/83) with a tendency to involve C6–C7 intervertebral disc space (24/83). The onset of the symptoms was acute in 53/83. Protrusions (43/83) were slightly more frequent than extrusions (39/83). Surgical treatment was chosen in 26/83 cases. Of those treated surgically, a ventral slot (16/26) or hemilaminectomy (8/26) was performed. Clinical signs resolved within 72 h in only 4/71 (5.6%) of dogs, within 3–7 days in 19/71 (26.8%), within 1–4 weeks in 28/71 (39.4%) and in 20/71 (28.2%) of dogs, clinical sign resolution was not reached within months or remained stable. We present a common syndrome that is difficult to manage and often results in unsatisfying outcomes. If our findings are confirmed by future studies, therapeutic protocols should be reevaluated.

## 77 (P 50)

### 77.1 Clinical Presentation, Diagnostic Findings, and Outcome in 27 French Bulldogs Diagnosed With Presumed Meningoencephalomyelitis of Unknown Origin

#### 77.1.1 E. Burbaite, DVM,^1^ E. Fiorentino, DVM, Dipl. ECVN,^1^ A. Gallucci, DVM, PhD, Dipl. ECVN,^2^ F. Tirrito, DVM, MRCVS, Dipl. ECVN,^3^ G. Gandini, DVM, PhD, Prof., Dipl. ECVN,^4^ M. Menchetti, DVM, PhD, Dipl. ECVN^1^

##### 77.1.1.1 ^1^ Neurology and Neurosurgery Division, San Marco Veterinary Clinic, Veggiano, Italy; ^2^ Neurological Veterinary Center La Fenice, Selargius, Italy; ^3^ AniCura Istituto Veterinario di Novara, Novara, Italy; ^4^Department of Veterinary Medical Science, University of Bologna, Bologna, Italy

Meningoencephalomyelitis of Unknown Origin (MUO) is an inflammatory‐immune‐mediated disease of the central nervous system frequently observed in French Bulldogs (FB). The purpose of this study was to provide details about neurological condition, magnetic resonance imaging (MRI) findings, and outcome in a population of FB affected by MUO. Twenty‐seven FB with a consistent diagnosis of MUO were retrospectively enrolled. Inclusion criteria comprised FB with complete medical records, detailed neurological examination, MRI findings and outcome. When available, the cerebrospinal fluid (CSF) results were also described. The mean age at the onset of clinical signs was 3.7 years. The most common neurological signs at presentation were acute blindness, seizures or cervical pain (it was present in 25.9%, 7/27 of each sign). On MRI, 66.7% (18/27) of the dogs had detectable lesions in the forebrain. The spinal cord and brainstem were affected in 40% and 37% of cases respectively. In 59.3% (16/27) of dogs, the CSF was examined and in 81.3% (13/16) of them, a mild lymphocytic pleocytosis was confirmed (median 18 cells/μL; range 2–262). 55.6% (15/27) of dogs relapsed. During the study, 17 dogs were still alive and their median survival time at the last follow‐up was 620 days (60–2190). The median survival time in 6 deceased animals was 60 days (7–2555). Our results provide additional information regarding breed‐specific disease findings. MUO in FB mostly affected the forebrain and brainstem with predominantly forebrain neurological signs. A high percentage of relapses, but an overall good survival time was observed.

## 78 (P 51)

### 78.1 Survey of Tick‐Borne Encephalitis Virus‐Associated Neuroinflammation: A Multicenter Study of 34 Dogs in Germany

#### 78.1.1 N. Schneider, Dr. Med. Vet.,^1^ A. Blutke, Prof., Dr. Med. Vet.,^2^ P. Quitt, Dipl. ECVN,^3^ F. Wieländer, Dr. Med. Vet., Dipl. ECVN,^3^ G. Buhmann, Dr. Med. Vet.,^3^ A. Fischer, Prof., Dr. Med. Vet., Dipl. ECVN, Dipl. ACVIM (Neurology),^3^ K. Jurina, Dr. Med. Vet., Dipl. ECVN,^4^ T. Steinberg, Dr. Med. Vet., Dipl. ECVN,^4^ J. Dietzel, Med. Vet.,^5^ S. Loderstedt, Dr. Med. Vet., Dipl. ECVN,^5^ C. Tästensen, Dr. Med. Vet.,^5^ T. Flegel, Prof., Dr. Med. Vet., Dipl. ECVN, Dipl. ACVIM (Neurology),^5^ D. Henke, Dr. Med. Vet., Dipl. ECVN,^6^ F. Mühlhause, Dr. Med. Vet., GPCert Neurology,^7^ M. Kronberg, Dr. Med. Vet., Dipl. ECVN,^7^ A. Olszewska, DVM, Dr. Med. Vet.,^8^ M. Schmidt, Prof., Dr. Med. Vet., Dipl. ECVN,^8^ B. Parzefall, Dr. Med. Vet., MVetMed, Dipl. ECVN^1^

##### 78.1.1.1 ^1^ Small Animal Clinic Oberhaching, Oberhaching, Germany; ^2^ Institute of Veterinary Pathology, Centre for Clinical Veterinary Medicine, Ludwig‐Maximillians‐University Munich, Munich, Germany; ^3^ Clinic of Small Animal Medicine, Centre for Clinical Veterinary Medicine, Ludwig‐Maximillians‐University Munich, Munich, Germany; ^4^ AniCura Tierklinik Haar, Haar, Germany; ^5^ Department of Small Animal Medicine, University of Leipzig, Leipzig, Germany; ^6^ Tierklinik Stuttgart‐Plieningen, Stuttgart, Germany; ^7^ AniCura Tierklinik Trier, Trier, Germany; ^8^ Department of Veterinary Clinical Sciences, Small Animal Clinic, Neurosurgery, Neuroradiology and Clinical Neurology, Justus‐Liebig‐University Giessen, Giessen, Germany

Tick‐borne encephalitis (TBE) is an emerging viral disease with increasing incidences, affecting humans, as well as dogs. The present study characterizes clinical signs, diagnostic findings, and outcomes in TBE‐infected dogs in Germany. Thirty‐four TBE‐infected dogs which presented to seven different referral clinics in Germany between 2011 and 2022 were analyzed retrospectively. Inclusion criteria were defined as follows: (1) clinical signs of central nervous system (CNS) disease, (2) cerebrospinal fluid (CSF) pleocytosis or magnetic resonance imaging‐, computed tomography‐, or histopathology‐findings consistent with CNS inflammation, and (3) the presence of either TBE‐virus CSF antibodies, serum IgM, or a four‐fold increase of IgG antibodies in paired serum samples, or a positive TBE‐virus PCR in CNS tissue. The patient collective included dogs of various breeds with a median age of 5.7 years of which the majority presented with fever between May and September. The most common neuroanatomical localization was multifocal CNS and included signs of abnormal behavior/consciousness (71%), ataxia (50%), flaccid paralysis (47%), cerebellar signs (32%), and tremor (29%). Neuroimaging showed CNS lesions in almost 80% of the cases and involved mostly the cerebral cortex, cervical spinal cord, thalamus, and brainstem but also the cerebellum (18%). Most patients were treated with corticosteroids with a survival rate of approximately 70% of which about 60% made a full recovery. The clinician should be aware of an increasing incidence of TBE in dogs in Germany and therefore should include TBE as a possible differential diagnosis for febrile multifocal CNS disease.

## 79 (P 52)

### 79.1 Using OpenAI'S ChatGPT‐4 to Build a Mobile Phone “App” for Bedside Calculation and Monitoring of Bladder Volume in Dogs

#### 79.1.1 J. Prager, BSc, BVSc, PhD, MRCVS,^1^ N. Granger, DVM, PhD, DipECVN, FHEA, FRCVS^1^

##### 79.1.1.1 ^1^ Bristol Vet Specialists, Bristol, UK

Estimating bladder volume in hospitalized companion dogs is a daily need for veterinary surgeons. There are many instances where this is crucial (e.g., oligo‐anuric patients) but is most common for paraplegic dogs requiring regular help to empty the bladder. The ideal timing of emptying is poorly defined and is often based on an estimation of bladder size by manual palpation or ultrasound. Formulas to calculate bladder volume from ultrasound measurements exist, however, performing these calculations and tracking bladder volume is cumbersome. The recent availability of generative artificial intelligence large language models has massively lowered the threshold of access to writing custom programs. We aimed to leverage OpenAI's ChatGPT‐4 to create a mobile phone “app” enabling quick calculation of bladder volume and tracking of volume over time. We started with a prompt asking ChatGPT‐4 to write Kotlin code for Android devices, and Swift code for iOS devices, providing “input boxes on one page … results on an output page … for the calculation: bladder volume = *L* × *W* × ([DL + DT]/2) × 0.625, where *L* is longitudinal bladder length, *W* is transverse bladder width, DL is longitudinal bladder depth, and DT is transverse bladder depth.” With iteration and debugging using ChatGPT‐4, such an app was developed. We present an Android and iOS app for the veterinary clinic, written in an estimated 10 h by non‐experts in coding. People with limited training in programming can therefore quickly write basic applications. Such tools could become more widely used in veterinary medicine for animal care.

## 80 (P 53)

### 80.1 Clinical and MRI Features of Hemorrhagic Transformation After Ischemic Stroke in a Dog

#### 80.1.1 A. Bellomo, DVM,^1^ C. Mattei, DVM, Dipl. ECVDI,^1^ M. Bernardini, DVM, Dipl. ECVN,^1^ ^,2^F. Balducci, DVM, Dipl. ECVN^1^

##### 80.1.1.1 ^1^ Anicura “Veterinary Hospital I Portoni Rossi,” Zola Predosa (BO), Italy; ^2^ Department of Animal Medicine, Production and Health, University of Padua, Legnaro, Italy, Legnaro (PD), Italy

Introduction: Hemorrhagic transformation (HT) is a well described complication of ischemic stroke in humans, associated with increased morbidity and mortality, secondary to blood–brain barrier disruption and reperfusion. Aim of this case report is to describe the MRI features of a clinically suspected HT after ischemic stroke in a dog.

Methods: An 8‐year‐old female neutered mixed‐breed dog presented with acute onset of intracranial clinical signs consistent with a left‐sided forebrain disease.

Results: MRI, performed within 12 h from the onset of clinical signs, revealed a large well‐defined area of high diffusion‐weighted imaging (DWI) signal and corresponding low apparent diffusion coefficient (ADC) map values, indicative of abnormal restricted diffusion, encompassing the majority of the vascular territory supplied by the left middle cerebral artery. The lesion was not visible in any other sequence, suggesting a peracute ischemic infarct. Cerebrospinal fluid examination was unremarkable. Concurrent medical conditions associated with the cerebrovascular accident were not identified. In the following 24 h the dog showed severe clinical deterioration, suggesting a brainstem involvement. A 40‐h follow‐up MRI revealed mixed T2‐weighted hyper‐ and hypointense areas in the same vascular territory, different combinations of DWI signal and ADC map values, and a severe mass effect. Extensive susceptibility artifacts in Flow Sensitive Black Blood imaging were detected, indicating HT of the previous ischemic infarct.

Discussion: HT may represent a sequela of ischemic infarcts in dogs as described in humans. Rapid and severe clinical deterioration in a patient previously diagnosed with ischemic stroke should raise suspicion and warrant further MRI evaluation.

## 81 (P 54)

### 81.1 An External Hydrocephalus Secondary to Meningoencephalitis of Unknown Origin (MUO) Treated With Negative Pressure

#### 81.1.1 N. Della Camera, DVM, Resident ECVN,^1^ C. Falzone, DVM, DECVN^1^

##### 81.1.1.1 ^1^ Diagnostica Piccoli Animali s.r.l, Zugliano (VI), Italia

The aim of this case report was to evaluate the efficacy of negative constant pressure as alternative treatment in course of external hydrocephalus. A 2‐year‐old female Maltese dog was presented with cluster of epileptic seizure. At the neurological examination a multifocal brain localization was performed. Inflammatory (sterile‐autoimmune or infective forms), vascular, metabolic and neoplastic forms were included in differential diagnosis. Magnetic resonance imaging (MRI) of the brain and cerebrospinal fluid (CSF) were compatible with an inflammatory form. A diagnosis of MUO was performed after ruled out infective disorders. Immunosuppressive treatment was undertaken but after 5 days the neurological condition worsened dramatically, and a new MRI examination revealed moderate external hydrocephalus with consequent brain compression predominantly on left side. A different craniotomy approach was performed with two calvaria sulci, after durotomy a low vacuum drainage system was bilaterally placed into the sulci and fixed to the skull with a sterile bone wax. After 24 h the clinical condition improved, and at 6th day the drainages were removed. The MRI follow‐up at this time showed the reduction of external hydrocephalus with right side was completely disappeared and a mild hydrocephalus still presents in the left hemisphere, but we decided to leave it without another drainage. One month later seizure activity relapses and owner request euthanasia. Different approaches were previously proposed to the surgical treatment of external hydrocephalus but in our opinion the reduced permanence of drainage associated to time of hydrocephalus reduction could prevent risk associated to this surgical procedure.

## 82 (P 55)

### 82.1 Comprehensibleness of Discharge Instruction for Small Animals in a Veterinary Neurological Referral Center

#### 82.1.1 T. Flegel, Dr. Med. Vet., DECVN, DACVIM,^1^ K. Dobersek, student,^1^ S. Bayer, student,^1^ V. Weiß, DVM,^1^ L. F. Becker, DVM,^1^ S. Loderstedt, Dr. Med. Vet., DECVN,^1^ C. Tästensen, Dr. Med. Vet.,^1^ J. Dietzel, DVM,^1^ T. Kalliwoda, DVM,^1^ I. C. Böttcher, Dr. Med. Vet., DECVN,^1^ A. Kühnapfel, Dr. Med,^2^ M. A. Harkenthal, Psychotherapist,^3^ S. Gutmann, Dr. Med. Vet^1^

##### 82.1.1.1 ^1^ Department for Small Animals, Leipzig University, Leipzig, Germany; ^2^ Institute for Medical Informatics, Statistics and Epidemiology, Leipzig University, Leipzig, Germany; ^3^ Department of Child and Adolescent Psychiatry, Faculty of Medicine, Technische Universität Dresden, Dresden, Germany

The aim of this study was to investigate the comprehensibleness of discharge instructions and influencing factors. Clients were asked to answer questionnaires regarding the disease of their pet, diagnostic tests performed, prognosis, and discharge instructions at time of discharge and 2 weeks later. The same questions were answered by discharging veterinary surgeons at time of discharge. Clients had to answer additional question regarding the subjective feelings during discharge communication. In addition, the following data were collected: data describing the discharging veterinarian (age, gender, years of clinical experience, and specialist status), data describing the client (age, gender, and educational status). The general agreement rate (AR) between answers of clinicians and clients as well as factors potentially influencing the AR were evaluated. Of 230 clients being approached 151 (65.7%) and 70 (30.4%) clients responded to the first and second questionnaire, respectively (132 dog owners and 28 cat owners). The general AR between clinician's and client's responses over all questions together was 69.4% and 66.6% at the two time points. Questions regarding side effects of medication (36.6%), exercise restriction (43.5%), and residual clinical signs (36.1%) had the lowest AR. The only factor affecting the total AR was the age of clients, with lower AR at higher ages (*p* = 0.008). In addition, there was a positive trend for clients with a medical profession (*p* = 0.057). Clients are only partially able to reproduce information provided at discharge. The only factors affecting that ability was an increasing client's age. Instructions deemed to be important should be specifically stressed during discharge.

## 83 (P 56)

### 83.1 Non‐Ambulatory Paraparesis Secondary to Neoplastic Invasion of the Ventral Vertebral Venous Sinus Plexus

#### 83.1.1 J. M. Segura Navarro, LV,^1^ A. Soler, LV,^1^ V. Cervera, LV, Dip ACVR, Dip ECVDI,^2^ S. Róedenas, LV, MRCVS, Dip ECVN^3^ ^,4^

##### 83.1.1.1 ^1^ Menescal Veterinary Hospital, Novelda, Spain; ^2^ AniCura Valencia Sur Veterinary Hospital, Valencia, Spain; ^3^ Bluecare Veterinary Hospital Vetpartners, Malaga, Spain; ^4^ AniCura Aitana Veterinary Hospital, Valencia, Spain

Pheochromocytomas and paragangliomas are rare neoplasms in dogs, with a high capacity for local invasion and a moderate capacity for distant metastases. These tumors are usually associated with the presence of tumor thrombi, forming non‐vascular tumors that extend into the vessel. A 7.5 years old male castrated Siberian Husky presented for further investigation after 2 months of abnormal gait in pelvic limbs. The signs had progressed until non ambulatory paraparesis. Neurological examination revealed delayed postural reactions and decreased spinal reflexes in both pelvic limbs. These clinical signs were consistent with a L4–S2 neurolocalization. Lumbar and abdominal pain on palpation were present with normal pulse in both pelvic limbs. Comprehensive blood tests revealed leukocytosis with neutrophilia and mild anemia. Biochemistry showed elevated liver enzymes. Computed tomography showed a retroperitoneal mass with vascular and bilateral ureteral invasion (with sever right and mild left pyelectasis) and invasion of the vertebral canal through the basivertebral canals/veins from L2 to L4 with dilation of the ventral vertebral internal venous plexuses, suspicious of a neuroendocrine neoplasia such as pheochromocytoma or paraganglioma arising from the left adrenal gland. Cytology of the abdominal mass was performed with a result compatible with a neuroendocrine tumor such as pheochromocytoma versus paraganglioma. Other less frequent tumors such include neuroblastomas or ganglioneuromas. Histopathology could not be performed. The particularity of the present case is the presence of non‐ambulatory paraparesis secondary to vascular invasion of the tumor, with dilatation of the ventral vertebral venous plexus and associated secondary spinal cord compression.

## 84 (P 57)

### 84.1 Case Report: Sensory Polyneuropathy of Leonberger Diagnosed in a Germanic Bear Dog

#### 84.1.1 V. Weiss, DVM,^1^ T. Flegel, Prof., Dr., DipECVN, DipACVIM,^1^ J. Dietzel, DVM,^1^ K. Matiasek, Prof., DVM, AM‐ECVN^2^

##### 84.1.1.1 ^1^ Department for Small Animals, Faculty of Veterinary Medicine, Leipzig University, Leipzig, Germany; ^2^ Section of Clinical & Comparative Neuropathology, Institute of Veterinary Pathology, Centre for Clinical Veterinary Medicine, Ludwig‐Maximilians‐Universitaet‐München, Munich, Germany

This case report describes a 5‐year‐old Germanic bear dog that presented due to gait abnormalities in the pelvic limbs that had been observed for 6 months. Clinical neurological examination revealed a high steppage pelvic limb gait, hindlimb muscle atrophy, generalized reduced segmental spinal reflexes, generalized delayed paw positioning and clinical signs of laryngeal paralysis.

The following diagnostics were performed: MRI examination of the thoracic and lumbar spine, electrodiagnostic and muscle nerve biopsies. The MRI examination was unremarkable, while electrodiagnostic studies showed mild spontaneous activity due to positive sharp waves and fibrillation potential in the tibialis cranialis muscle, gastrocnemius muscle, and extensor carpi radialis muscle. Motor nerve conduction velocity could not be derived in all four limbs. Muscle biopsy of the tibialis cranialis muscle and gastrocnemius muscle showed diffuse, chronic, severe denervation‐type muscle atrophy. Nerve biopsy of the common peroneal nerve showed diffuse, chronic, severe instability of the myelin sheath, confirming an intermediate neuropathy. These findings are comparable to findings in Leonbergers with breed specific sensory polyneuropathy. Most of the lesions of myelinated fibers were consistent with the changes of Leonberger neuropathy. The diagnosis of Leonberger sensory polyneuropathy was confirmed by positive genetic testing for the mutation in the ARGHGEF10 gene. A retrospective pedigree analysis revealed a previous crossbreeding with Leonbergers. These findings indicate that the Germanic bear dog is carrying the affected gene for Leonberger sensory polyneuropathy. Consequently, genetic testing should be considered in Germanic bear dogs if compatible clinical findings are present.

## 85 (P58)

### 85.1 Occult Tethered Cord Syndrome in a Dog With Abnormal Electromyography of the Tail

#### 85.1.1 K. Lam, BVetMed, MRCVS,^1^ J. J. Minguez, DVM, MRCVS,^1^ C. Fina, DEDV, DipECVDI, MRCVS,^1^ J. J. Camarasa, DVM, MSc, MRCVS,^1^ C. Posporis, DVM, PGCertVSM, DipECVN, MRCVS^1^

##### 85.1.1.1 ^1^ Pride Veterinary Referrals, IVC Evidensia Group, Derby, UK

A 4‐year‐old male neutered crossbreed dog was presented with pelvic limb and tail allodynia/paraesthesia since a puppy. Progressive pelvic limb stiffness and lameness associated with painful low tail carriage exacerbated by exercise were also reported. There was no fecal/urinary incontinence.

Neurological examination revealed a stilted pelvic limb gait, mildly reduced pelvic limb withdrawal reflexes and pain on tail and lumbosacral palpation. Pelvic limb orthopedic and radiographic examinations were unremarkable. Hematology, biochemistry and CSF analysis were normal. Dynamic lumbosacral MRI revealed a fixed position of the conus medullaris at L7–S1 without other abnormalities. Electromyography showed fibrillation potentials and positive sharp waves in the muscles of the tail base. Analgesia with gabapentin, amitriptyline, and carprofen failed to prevent further neurological deterioration. Surgery was elected as treatment trial for presumptive occult tethered cord syndrome. Pre‐operative electrodiagnostic testing showed persistence of previous findings. A dorsal laminectomy was performed at L7–S2. The filum terminale externum was under mild tension and was resected. The dura of the conus medullaris appeared thickened and exploratory durotomy revealed intradural adhesions which were removed together with the filum terminale internum. Histopathology of the filum terminale revealed mature collagenous tissue arranged longitudinally at the periphery. Significant improvement in gait, exercise capacity, pain and allodynia/paresthesia was observed immediately, 2 and 5 weeks post‐operatively.

This case report reiterated the value of dynamic lumbosacral MRI and introduced the use of electromyography as potential diagnostic tools for occult tethered cord syndrome. To the authors' knowledge, electromyography has not been reported for this condition in dogs.

## 86 (P 59)

### 86.1 Neurological Examination in Guinea Pigs—What is Feasible, Reliable, and Generates Little Stress

#### 86.1.1 I. C. Boettcher, DVM, Dipl. ECVN,^1^ T. Grochow, DVM,^2^ ^,3^ J. Dietzel, DVM^1^

##### 86.1.1.1 ^1^ Department for Small Animals, Leipzig University, Leipzig, Germany; ^2^ Institute of Anatomy, Histology and Embryology, Leipzig University, Leipzig, Germany; ^3^ Institute of Parasitology, Leipzig University, Leipzig, Germany

Aim of the study was to perform a standard neurological examination in guinea pigs according to dogs and cats and to evaluate cooperation, responsiveness and associated stress level. Twenty‐five healthy guinea pigs were examined by two different neurologists in their familiar surroundings including hands‐off observation (mentation, behavior, posture, face symmetry, and gait), proprioceptive testing, cranial nerve tests, and spinal reflex tests. With hands‐off, no guinea pig showed signs of stress and all cooperated except for gait as some animals refused to walk. All postural reaction tests induced stress in 8%–48% of the animals with varying cooperation. Minor stressful tests with excellent cooperation were wheelbarrowing, hopping reactions and paw replacement in thoracic limbs. Hind limb postural reaction tests were stressful and up to 1/3 of the animals refused cooperation. Hemiwalking was the only reliable test for the hind limbs. All spinal reflex tests induced stress in 60%–100% of the animals and were either negative or unreliable except for withdrawal reflexes. Cranial nerve tests were the least stressful tests with excellent cooperation for most reflexes. Menace response, testing for mandibular sensation, and vertical oculocephalic reflex were negative, however. Most tests showed less pronounced responses than in dogs and cats. The two examiners diverged in interpretation of responsiveness. Front limb postural reaction tests, hemiwalking and most cranial nerve tests can be used routinely in guinea pigs. Most reflex tests and hind limb postural reaction tests lack a consistent response. Withdrawal tests are the only reliable reflex tests, but highly elicit stress.

## 87 (P 60)

### 87.1 Magnetic Resonance Imaging Findings in of the Peripheral Nerves That Compose the Brachial Plexus in 27 Dogs and Cats

#### 87.1.1 V. Rodriguez Gomez, DVM MRCVS,^1^ A. Caine, MA VetMB DipECVDI CertVDI FRCVS,^1^ G. Bruto Cherubini, DVM DipECVN FRCVS EBVS & RCVS Professor Veterinary University^1,2^

##### 87.1.1.1 ^1^ Dick White Referrals, Cambridgeshire, UK; ^2^ University of Pisa, Pisa, Italy

Objective: To identify the pattern of MRI changes associated with an imaging diagnosis of neoplastic, inflammatory or traumatic causes of brachial plexus pathology.

Methods: This is a descriptive retrospective study. Brachial plexus cases were identified from the MRI database from 2011 to 2017. Cases with muscle, bone or purely spinal cord were excluded.

Cases were divided into an imaging diagnosis of neoplasia, inflammatory or traumatic etiology. Any case with an open imaging diagnosis was excluded from the analysis. MRI findings were reviewed and correlated with the diagnosis.

Results: A total of 27 cases: 22 dogs, 5 cats. Neoplastic: (16/27) 59.3% had a mean age of 9.44 (3–14). On MRI, (12/16) 75% unilateral, (4/16) 25% bilateral. They presented with a mass in (7/16) 43.8%, and thickening in (9/16) 56%. There was contrast enhancement in (13/16) 81.3%. Muscle atrophy was seen in (11/16) 68%, lymphadenopathy (6/16) 37.5%. Inflammatory: (9/27) had a mean age of 7.44 (range 2–11). On MRI, (7/9) 77.8% unilateral, (2/9) 22.2% bilateral. All 9 presented with diffuse thickening of the nerve. There was contrast enhancement in (9/9) 100% Muscle atrophy was seen in (2/9) 22.2%, lymphadenopathy (1/9) 11.1%. Trauma: (2/27) had a mean age of 4.5 (range 3–6). Both cases were unilaterally affected and had diffuse nerve thickening. The contrast was administered in one, with perineural contrast uptake. Neither had muscle atrophy nor lymphadenopathy.

Conclusion: Diagnosis of a neoplastic lesion on MRI was more likely with a mass lesion, and muscle atrophy, however there is a large overlap in appearance of neoplastic and inflammatory brachial plexus pathology.

## 88 (P 61)

### 88.1 Neurological Manifestations as the Initial Presentation in Dogs With Acute Leukemia

#### 88.1.1 F. Lyseight, MVSc, MRCVS,^1^ C. Pittaway, BSc (Hons), BVSc (Hons), Dip ECVIM‐CA (Onc), MRCVS,^1^ R. Dennis, MA, VetMB, DVR, DipECVDI, FRCVS,^2^ G. Bruto Cherubini, DVM, DECVN, FRCVS^3^ ^,4^

##### 88.1.1.1 ^1^ Oncology Service, Dick White Referrals, Cambridgeshire, UK; ^2^ Diagnostic Imaging Service, Dick White Referrals, Cambridgeshire, UK; ^3^ Neurology and Neurosurgery Service, Dick White Referrals, Cambridgeshire, UK; ^4^ Department Veterinary Sciences University Pisa, Pisa, Italy

The aim of this retrospective study is to describe the clinical and MRI findings in dogs diagnosed with acute leukemia (AL) presenting with neurological deficits. Medical records of dogs presenting to the neurology service of a private referral hospital between January 2010 and December 2022 were reviewed for dogs with an abnormal neurological examination ultimately diagnosed with AL. Six dogs were included. All had an abnormal gait (paresis or ataxia) on assessment. Five dogs had a focal neuroanatomical localization: brainstem and/or cerebrum (*n* = 3), C6–T2 (*n* = 1), and L4–S3 (*n* = 1). Two dogs with vestibular dysfunction were euthanized following the diagnosis of AL. The remaining four dogs underwent MRI of the affected region of the nervous system. All dogs showed meningeal contrast enhancement and three dogs also had diffuse, ill‐defined, intra‐axial, hyperintense patches (T2W/STIR) in the cortical gray and white matter, and/or spinal cord. The bone marrow of the calvarial bones, manubrium and scapulae showed abnormal signal intensity in three dogs (TS T1/C), characterized by loss of fat opacity and contrast enhancement. Other findings included spinal nerve thickening with myositis (*n* = 2; TS T2W). Four dogs with CSF analysis had increased protein; and three had pleocytosis between 9 and 27 μL (ref.: 0–5; mixed *n* = 1, lymphocytic *n* = 1, neutrophilic *n* = 1). No atypical cells were observed. The changes described could be due to “leukemic meningitis,” secondary to paraneoplastic syndromes (e.g., hyperviscosity), other inflammatory or infectious conditions. This is the first study in which bone marrow changes on MRI in dogs with hematopoietic cancer are reported.

## 89 (P 62)

### 89.1 Mycobacterium Affecting the Brainstem in a Cat

#### 89.1.1 C. Cardoso Diogo, DVM, MSc, MSci, PhD,^1^ D. Gunn‐Moore, MACVSc, RCVS Specialist, FHEA, FRSB, FRCVS,^2^ C. O'Halloran, BVSc, MSc, PhD,^2^ F. Raimondi, DVM, DipECVN, EBVS^1^

##### 89.1.1.1 ^1^ Neurology Department of Southern Counties Veterinary Specialists, Ringwood, UK; ^2^ Feline Medicine Department of University of Edinburgh, Edinburgh, UK

*Mycobacterium avium* is a gram‐positive bacillus, which produces bacteremia and occasionally disseminates systemically. However, this bacterium usually does not affect the central nervous system (CNS) in cats. A 10‐year‐old female Domestic Short Hair presented with a 2‐week history of progressive proprioceptive ataxia and hemiparesis in the left limbs. Three years previously, lymphadenopathy caused *Mycobacterium* was diagnosed, recovering completely after treatment. The cat showed normal mentation, ambulatory tetraparesis, more affected in the left side, reduced postural reactions in the left thoracic and pelvic limbs and absent menace response in the left side. The physical examination was unremarkable apart of enlarged submandibular lymph node. A multifocal neurolocalization was suspected. MRI revealed a right‐sided intra‐axial mass lesion in the midbrain, T2W, FLAIR, and T1W isointense with strong homogenous contrast enhancement, associated with extensive perilesional edema, resulting in displacement of the interthalamic adhesion, mild impingement of the cerebellum and caudal transtentorial brain herniation. The right submandibular lymph node was significantly enlarged, contrast enhanced. CSF analysis revealed increased protein. Cytology from lymph node showed *Mycobacterium*, Ziehl–Neelsen positive stained. Antibiotic therapy (pradofloxacin, rifampicin, and azythromycine) was performed for 3 months. The cat showed gradual neurological improvement. MRI was repeated 1 month after finished the treatment and the lesion was not visualized anymore. Six months later, the cat was still clinically stable, without neurological signs. *Mycobacterium avium* affecting the CNS has rarely been reported. This report highlights the importance of this disease in regards of an unusual and atypical presentation of this infection.

## 90 (P 63)

### 90.1 Canine Vestibular Paroxysm or Suspected Vestibular Epilepsy? A Study on Eleven Dogs

#### 90.1.1 T. Al Kafaji, DVM, ECVN Resident,^1^ F. Tocco, DVM,^1^ S. Okonji, DVM, ECVN Resident,^2^ A. Gallucci, DVM, PhD, ECVN Diplomate, EBVS European Specialist^1^

##### 90.1.1.1 ^1^ Centro Neurologico Veterinario “La Fenice,” Selargius (CA), 09047, Italy; ^2^ Department of Veterinary Medical Sciences, University of Bologna, Ozzano Emilia (BO), 40 064, Italy

In humans' vestibular epilepsy (VE) is described as focal seizures with transient vestibular signs. The cortical representations of the vestibular system may be involved in this kind of epilepsy. In dogs, only two cases with vestibular episodes called vestibular paroxysmia (VP) are reported. To the authors knowledge this is the first attempt to define the clinical features, phenotypical manifestation, and outcome of canine suspected VE. Medical records of dogs between 2009 and 2023 were retrospectively reviewed. Inclusion criteria were a neurological examination, a history of transient vestibular signs, unremarkable blood exams, and a minimum 6 month follow‐up. Clinical improvement was defined as a reduction in frequency or cessation of clinical signs after the onset of antiepileptic drugs (AED). Eleven dogs were included. Pug dogs were the most represented breed 7/11 (63%). In three cases additional generalized tonic–clonic (GTC) seizures were reported. Basing on advanced diagnostic imaging and cerebrospinal findings, vestibular episodes were classified as suspected idiopathic in 9/11 (82%) patients. In the other two (18%), vascular lesions and brain atrophy were identified. Electroencephalography (EEG) performed in two dogs showed interictal spikes at the level of the fronto‐temporal and fronto‐parietal areas. All patients received AED with clinical improvement in 10/11 dogs (90%). Although differentiation between VE and VP is challenging, in this study presence of GCT seizures, EEG interictal spikes and responsiveness to AED therapy, supported the hypothesis of an epileptic origin of vestibular episodes and thus the existence of a canine vestibular epilepsy, with an idiopathic cause in the majority of dogs.

## 91 (P 64)

### 91.1 Personality Contributors to Veterinarians' Perspectives of Neurology

#### 91.1.1 A. Shea, BVSc DipECVN PGCertVetEd,^1^ E. Norman, EdD, MVM, BVSc,^1^ N. Cogger, BSc (Hons), PhD^1^

##### 91.1.1.1 ^1^ School of Veterinary Science Tawharau Ora, Massey University, Palmerston North, New Zealand

Introduction: Poor perspectives of neurology among doctors are common and may compromise patient care. Veterinarians' perspectives of neurology are unknown.

Methods: In a mixed‐method study, 18 veterinarians with differing attitudes towards neurology were interviewed in‐depth about their experiences of neurology. Systematic thematic analysis then informed a survey of veterinarians. Attitudes to neurology were categorized and their relationship with potentially‐related factors was tested using Pearson's chi‐squared test for categorical data and Kruskal–Wallis ANOVA for continuous data. Differences were considered significant at *p* < 0.05.

Results: Of the 111 surveyed veterinarians, 44% disliked/avoided neurology, 25% were indifferent, and 30% enjoyed neurology. Interview and survey respondents who disliked/avoided neurology tended to focus on solutions and definitive answers, and internalize failure, while respondents who enjoyed neurology focused on intellectual interest. Survey respondents who enjoyed neurology were significantly more likely to display adaptive perfectionism (*p* = 0.03), greater tolerance of uncertainty (*p* = 0.00001), and had more of a growth mindset (*p* = 0.04) than respondents who disliked/avoided neurology. Respondents with more exposure, logic‐based understanding, and/or enjoyment of challenge, often expressed more positive perspectives of neurology even if self‐confidence was low.

Discussion: Response to internal conflict—inability to act in a way perceived correct—seemed pivotal to changes in attitude towards neurology. Failure to address this conflict resulted in persistent negative perspectives of neurology, often driven by emotional rather than intellectual factors. Personality traits such as perfectionism, intolerance of uncertainty, and mindset appear to affect the ability to resolve internal conflict associated with neurology.

## 92 (P 65)

### 92.1 Contributors to Veterinary Students' Perspectives of Neurology

#### 92.1.1 A. Shea, BVSc DipECVN PGCertVetEd,^1^ E. Norman, EdD, MVM, BVSc,^1^ N. Cogger, BSc (Hons), PhD^1^

##### 92.1.1.1 ^1^ School of Veterinary Science Tawharau Ora, Massey University, Palmerston North, New Zealand

Introduction: Medical students have been suggested to have predominantly poor perspectives of neurology. Little is known regarding veterinary students' perspectives of neurology.

Methods: Students at a single veterinary school were surveyed to determine their attitude to neurology and investigate factors potentially related to their attitude such as study approach, motivational factors, tolerance of uncertainty and tendency to perfectionism. The relationship between potential factors and attitude to neurology were tested using Pearson's chi‐squared test for categorical data and Kruskal–Wallis ANOVA for continuous data. Differences were considered significant at *p* < 0.05.

Results: Of the 151 responding students, 34% disliked/avoided neurology, 34% were indifferent, and 32% enjoyed neurology. The most frequently selected aspects of neurology that students liked or disliked related to intellectual ease or difficulty. Students who disliked/avoided neurology reported significantly more emotional difficulties learning neurology (e.g., “overwhelming,” feeling “stupid”), higher maladaptive perfectionism scores, and were more likely to focus on outcome measures and study by memorization than students who enjoyed neurology. In contrast, students who enjoyed neurology had significantly higher adaptive perfectionism scores, tolerance of uncertainty, and internal motivation. They were more likely to focus on gaining knowledge, adopt deeper learning approaches, and retain more of what they learnt.

Discussion: Personality factors and emotional responses appeared most strongly tied to veterinary students' attitude towards neurology despite students' primarily intellectually‐based descriptors of what they liked or disliked about neurology. Reaction to challenge and failure, type of motivation, and study approach likely create cycles of enjoyment or dislike of neurology.

## 93 (P 66)

### 93.1 Cervical Intradural‐Intramedullary Disc Extrusion: A Case Report With Post‐Surgical and Long Term Magnetic Resonance Follow‐Up

#### 93.1.1 M. Baccolini, DVM,^1^ R. Lombardo, DVM,^1^ F. Iocca, DVM,^1^ A. Terraneo, Dr,^1^ M. Bonaldi, DVM,^1^ F. Cozzi, DVM^1^

##### 93.1.1.1 ^1^ Clinica Neurologica Veterinaria, Milano, Italy

Intradural‐intramedullary disc extrusion (IIVDE) is a rare and poorly described condition in dogs. The purpose of this case report is to describe the clinical and MRI follow‐up in a dog with IIVDE. A 10 years old mix breed dog was referred for subacute and progressive onset of right hemiparesis. Right ambulatory hemiparesis with marked proprioceptive deficits in the right forelimb and decreased withdrawal reflex were noticed. No neck pain was present. Cervical MRI showed a massive spinal cord compression at the C3–C4 level, with right lateralization. The signal characteristics and peculiar location of the compressive material were compatible with IIVDE. The dog then underwent ventral slot surgery: a moderate amount of extruded extradural material was found within the vertebral canal. During its removal, CSF leaked from the torn dura mater. There was no clear demarcation between intramedullary material and the nervous tissue, therefore it was decided to remove the extradural material and to end the surgery. A post‐operative MRI showed that the extradural disc material had been removed, but a significant amount of disc material was still present within the spinal cord. Marked improvement was noticed after surgery, and after 1 week only mild monoparesis in the right forelimb was present. Exactly 40 days after surgery the dog had a normal neurological examination. A control MRI was performed, and no changes were noticed compared with the first postoperative MRI.

Despite the persistence of disc material within the spinal cord the dog is clinically normal at 2 months from surgery.

## 94 (P 67)

### 94.1 A Case of Stiff Dog Syndrome in a Maltese Dog

#### 94.1.1 V. Buffagni, Neurology Resident,^1^ D. Dell'Apa, Neurology Resident,^1^ F. Fidanzio, DVM, PhD,^1^ R. Ciccimarra, Biotech, PhD,^1^ F. Ravanetti, Professor, DVM, PhD,^1^ F. Tummolo, Biotech, PhD,^2^ E. Bianchi, Professor, DVM, Dipl. ECVN^1^

##### 94.1.1.1 ^1^ Department of Veterinary Science, University of Parma, Parma, Italy; ^2^ Euroimmun Italia, Padova, Italy

A 9‐year‐old, male, Maltese dog was presented with gait abnormalities which had progressively worsened over the last 3 years. Neurological examination revealed tetraparesis, exercise intolerance, and a generalized increase in limb muscles tone, which was exacerbated by stress and manipulation. The patient also presented generalized muscle hypertrophy and orthopedic problems. Blood count, biochemistry and urinalysis were unremarkable. Neospora caninum serology was negative. Electromyography showed continuous motor unit action potentials (MUAPs) both in agonist and antagonist muscles in the awake patient, and no spontaneous pathologic activity during general anesthesia. Brain and cervical MRI were not consistent with clinical presentation. Cerebrospinal fluid (CSF) analysis was unremarkable. Indirect immunofluorescence technique (IFT) based on transfected cells detected the presence of Glutamic Acid Decarboxylase (GAD) autoantibodies in serum (Euroimmun, Germany). These results were consistent with a human condition called “Stiff Person Syndrome” (SPS). The SPS is an immune‐mediated disease with different clinical forms. The classic form is characterized by an insidious onset and progression over months of trunk muscle rigidity, which spreads to the limbs, often leading to orthopedic problems. A diagnosis of SPS is made through clinical presentation, neurological examination, exclusion of other neurological disorders, electromyographic detection of simultaneous MUAPs in agonist and antagonist muscles, and the presence of GAD‐autoantibodies in serum or CSF. A disorder similar to SPS has been previously described only once in a mixed‐breed dog (“Stiff Dog Syndrome” [SDS]). To the best of the authors' knowledge, this is the first report describing a case of SDS in a Maltese dog.

## 95 (P 68)

### 95.1 Surgical Treatment of Iatrogenic Meningoencephalocele and Pneumocephalus in a Cat

#### 95.1.1 R. Herzig, Dr. Med. Vet.,^1^ P. Natsios, Dr. Med. Vet.,^2^ K. Beckmann, Dr. Med. Vet., Diplomate ECVN^1^

##### 95.1.1.1 ^1^ Division of Neurology, Department for Small Animals, Vetsuisse Faculty, University of Zurich, Zurich, Switzerland; ^2^ Clinic of Small Animal Surgery, Vetsuisse Faculty, University of Zurich, Zurich, Switzerland

Introduction: Transfrontal meningoencephalocele (MEC) has been reported as consequence of transfrontal craniotomy in cats. MEC is rarely complicated by pneumocephalus in dogs. There is no detailed description of the clinical signs and MRI findings of pneumocephalus in cats. Surgical correction has been described in a few cases using various approaches.

Purpose: We report the diagnosis, surgical treatment, and outcome of an intranasal MEC and intraventricular tension pneumocephalus in a cat after transfrontal craniotomy.

Methods: An 8‐year‐old cat was referred for resection of an intracranial mass. Surgical resection was performed using a transfrontal approach. Dural and bony defects were left open. Additionally, radiation therapy was performed. Seven weeks after surgery, the cat presented due to progressive deterioration, including sneezing attacks and decerebrate rigidity. An intranasal meningoencephalocele complicated by intraventricular tension pneumocephalus was identified on MRI. Surgical repair of the defect was attempted using artificial dura mater and an autologous fascial graft of the temporal muscle. Follow‐up MRI was performed 5 weeks after surgery. Results Surgery resulted in rapid neurological improvement. MRI revealed complete resolution of the pneumocephalus. However, closure of the defect was incomplete. A small chronic bleeding within the right frontal area remained. Proprioceptive deficits contralateral to the MEC and nasal discharge remained as long‐term sequelae.

Discussion/Conclusion: MEC might occur as complication after transfrontal craniotomy in association with pneumocephalus in cats. Although complete resolution of pneumocephalus was achieved, the herein described surgical technique was not sufficient for complete closure of the bony and meningeal defect. The optimal technique remains to be established.

## 96 (P 69)

### 96.1 MRI Findings of a Dog With Suspected Steroid‐Responsive‐Meningitis‐Arteritis (SRMA) Presented With A T3–L3 Myelopathy Secondary to Intradural‐Extramedullary Hemorrhage

#### 96.1.1 G. Vitello, MRCVS, DVM,^1^ R. Fernandes, MRCVS, DipECVN,^1^ A. Holmes, MRCVS, EBVS, DipECVIM‐CA,^1^ M. Mariscoli, MRCVS, DVM, EBVS, DipECVN^1^

##### 96.1.1.1 ^1^ Paragon Veterinary Referrals, Wakefield, UK

Objectives: To describe MRI findings of intradural‐extramedullary hemorrhage secondary to SRMA leading to severe thoracolumbar myelopathy.

Methods: A 16‐months‐old, male neutered, crossbreed dog was presented with acute onset of inappetence, lethargy, pyrexia and cervical hyperaesthesia. Computer tomography (CT) of the head and cervical spine; and cerebrospinal fluid analysis (CSF) were initially performed. Three days later, MRI scanning of the thoracolumbar spine was performed due to progression to paraplegia with absent nociception.

Results: CT scan was unremarkable. CSF analysis revealed a marked neutrophilic pleocytosis (455/μL; reference range < 6) and elevated CSF proteins (115 mg/dL; reference range 5–27). CSF culture, PCRs for protozoal, and viral diseases were negative. Angiostrongylosis and coagulopathies were ruled out. MRI revealed a tubular well delineated lesion located laterally to the spinal cord at the level of T12–T13 extending dorsally and caudally to the level of T13–L1, causing severe spinal cord compression. The lesion was markedly T2‐hyperintense, T1‐hypointense, delineated by a thick irregular hypointense rim generating a moderate signal void on T2* and faintly enhanced by gadolinium. The subarachnoid space from T8 to L4 showed heterogenous T2 signal and was irregularly shaped. Prednisolone (2.5 mg/kg SID) and mycophenolate (12.3 mg/kg SID) were administered. Lack of improvement led to humane euthanasia 13 days after presentation.

Statement (Conclusions): To the authors knowledge this is the first case reporting intradural‐extramedullary hemorrhage resulting in severe thoracolumbar spinal cord compression and associated myelopathy secondary to SRMA.

## 97 (P 70)

### 97.1 Nasopharyngeal Obstruction Secondary to a Basisphenoid Meningocoele in a Crossbreed Dog Managed by Surgical Excision

#### 97.1.1 J. Deacon, BVMS, CertSAS, MRCVS^1^

##### 97.1.1.1 ^1^ Moorview Referrals, Cramlington, UK

Case report documenting an unusal presentationg clinical signs for a canine congenital meningocele.

A 7‐month, female entire crossbreed dog presented with a history of recurrent, progressive, and stertorous breathing. Investigations including retroflexed nasopharyngoscopy and contrast computed tomography documented a smoothly marginated, fluid filled structure occluding the nasopharynx. A defect in the basisphenoid bone was identified communicating with the calvarium. Contrast magnetic resonance imaging was most consistent with a meningocoele.

Surgical resection was performed via a ventral median approach through the hard and soft palate with the patient in dorsal recumbancy to reduce the risk for overdrainage of the ventricular system. The meningocoele was decompressed by aspiration and ablated with electrocoagulation. The basisphenoid defect was debrided with a high‐speed burr to expose the cancellous bone. The defect was filled with cancellous autograft harvested from the proximal humerus and covered with a titanium mesh and mucosal flap. Histopathology confirmed the resected tissue as a meningocele.

No recurrence of the clinical signs was observed in the initial 20 months post‐operative follow up.

This case demonstrates an unusual congenital anomaly to add to the differential diagnosis list for upper respiratory obstruction and an option for surgical management.

## 98 (P 71)

### 98.1 Eosinophilic Cerebrospinal Fluid Pleocytosis in a Dog With Idiopathic Hypertrophic Pachymeningitis

#### 98.1.1 D. Dauden, DVM, MRCVS,^1^ E. Davies, BVS, MRCVS,^2^ S. Platt, BVM&S, Dip. ACVIM (Neurology), dip. ECVN, FRCVS,^3^ H. Crosby‐Durrani, BVMS (Hons), Dipl. ECVP‐CFVP, AFHEA, MRCVS,^4^ E. Ricci, MVB, PhD, Dip. ACVP, FHEA, MRCVS,^4^ A. Aworinde, DVM, MSc, MVM, FRCPath, MRCVS,^5^ E. Alcoverro, DVM, Dip. ECVN, MRCVS^1^

##### 98.1.1.1 ^1^ ChesterGates Veterinary Specialists, Chester, UK; ^2^ Severn Edge Veterinary Group, Ludlow, UK; ^3^ VetOracle, Norfolk, UK; ^4^ University of Liverpool, Neston, UK; ^5^ Axiom Laboratories, Devon, UK

An 8‐year‐old Whippet crossbreed was presented with a month's history of collapsing episodes and altered mentation in between them. Clinical improvement was noted upon administration of immunosuppressive doses of prednisolone before referral. Neurological examination revealed mildly obtunded mentation with no gait or postural abnormalities. Moderate left‐sided masticatory muscle atrophy was noted. Postural reactions were mildly delayed in the left thoracic and right pelvic limbs. On the left eye, the palpebral reflex was absent, and the corneal reflex and menace response were markedly reduced. Left sided facial paraesthesia (reduced sensation and allodynia) was noted. The findings were compatible with a disease affecting the brainstem (pons and medulla oblongata). Magnetic resonance imaging revealed marked, diffuse T2W hyperintense and contrast‐enhancing thickening of the dura mater around the left parietal and temporal cerebral cortices, left piriform lobe, and cerebellar hemispheres. Cerebrospinal fluid (CSF) analysis revealed marked eosinophilic pleocytosis with increased protein concentration. Infectious diseases potentially causing eosinophilic pleocytosis tested negative. Computed tomography of thorax and abdomen did not reveal any significant abnormalities. The owners elected euthanasia 4 days after diagnosis. Post‐mortem examination revealed diffuse, severe, subacute to chronic eosinophilic pachymeningitis. Eosinophilic myositis was found in the temporal and masseter muscles. Eosinophilic CSF pleocytosis has been reported in a number of canine inflammatory (sterile and infectious) and neoplastic encephalopathies. Idiopathic hypertrophic pachymeningitis should be considered a differential diagnosis in dogs with eosinophilic pleocytosis.

## 99 (P 72)

### 99.1 The Use of Patient Specific 3D Printed Guides and Implants to Perform a Seventh Lumbar Vertebrectomy in a Cat

#### 99.1.1 J. Deacon, BVMS, CertSAS, MRCVS,^1^ J. Bongers, DVM, MRCVS,^1^ B. Oxley, MA, VetMB, DSAS (Orth), MRCVS^2^

##### 99.1.1.1 ^1^ Moorview Referrals, Cramlington, UK; ^2^ Vet3D, Cumbria, UK

A case report demonstrating the feasibility and surgical outcome for feline vertebrectomy using 3D printed patient specific guides and implants. A 7‐year, neutered male, domestic long haired cat presented with a 2 year history of progressive ambulatory paraparesis, tail plegia, urinary incontinence, and intermittent fecal incontinence consistent with an L6–S3 neurolocalization. Radiographs had documented an expansile lesion within the L7 vertebral body enlarging over an 18 month period. At the time of presentation, the patient was receiving oral phenylpropanolamine and manual bladder expression three times daily.

Contrast computed tomography documented a significant enlargement of the entire vertebral body, pedicles, and dorsal lamina with effacement of the spinal canal. A core bone biopsy was most consistent with an osteosarcoma. No distant metastasis were identified. Custom cutting and drilling guides were printed to facilitate a left iliac osteotomy and placement of a temporary acrylic plate to support the L6–S123 unit while the vertebra was resected. A custom support was used to protect and suspend the corda equina while bilateral 316 L printed plates were implanted to replace the vertebral body, lateral and dorsal lamina. The iliac osteotomy was reduced with a custom reduction guide and stabilized with a 2.4 mm LCP. Histopathology confirmed a giant‐cell osteosarcoma. The patient was discharged with ambulatory paraparesis and a continuing requirement for manual bladder expression and oral phenylpropanolamine. This case demonstrates the opportunities for accuracy and stabilization afforded by patient specific printed guides and implants. The surgical approach demonstrates an opportunity for successful vertebral resection and could be considered in appropriate patients with local disease.

## 100 (P 73)

### 100.1 Management of L7 Osteochondrosis With Secondary Lumbosacral Degenerative Stenosis by Vertebral Distraction and Dorsal Fusion in a Dog

#### 100.1.1 H. Thatcher, BVM BVS MRCVS (Hons),^1^ C. Driver, BSc BVetMed (Hons) MVetMed PhD DipECVN MRCVS^1^

##### 100.1.1.1 ^1^ Section of Neurology and Neurosurgery, Small Animal Clinic, Vetsuisse Faculty, University of Zurich, Zurich, Switzerland

This report describes a case of osteochondritis dissecans (OCD) in a 3‐year‐old, female neutered, Labrador Retriever. The OCD lesion uncommonly affected L7, resulting in lumbosacral degenerative stenosis with marked asymmetric radiculitis. Surgical intervention involved indirect radicular decompression by vertebral distraction and dorsal fusion.

Presenting signs included poor exercise tolerance, inability to stand for long periods and intermittent lameness. Imaging revealed an OCD lesion of the right caudodorsal end‐plate of L7. There was an elevated fibro‐osseous flap causing marked foraminal stenosis and compressive radiculitis. Due to conservative treatment failure and concerns over progressive radiculopathy, surgical management was indicated. The surgery aimed to remove the protrusive portion of the intervertebral disc, then distract and fuse the vertebrae in a flexed position, thus indirectly decompressing the L7 nerve root. This was achieved by dorsal laminectomy, partial annulectomy, and vertebral stabilization with paired 3.5 mm poly‐axial titanium alloy pedicle screws (2 in L7 and 4 in the sacrum) linked with 3.2 mm rods and cross‐bars. Fusion was assisted by curettage of the articular facet cartilage then deposition of cancellous autograft harvested from the proximal humeri mixed with 2 cc of demineralized bone matrix sculpted into two columns alongside the laminectomy defect. Long‐term follow‐up, 1 year from the initial surgery, revealed dorsal columnar fusion and a positive clinical outcome with normal gait and exercise ability. At 15 months, a mild relapse of signs including difficulty sitting was reported. Follow‐up CT revealed sacroiliacitis, which successfully responded to medical management and the patient again returned to normal exercise.

## 101 (P 74)

### 101.1 Behavioral Disorder in Dogs: A Retrospective Study Based on EEG, MRI Results, and Temporal Lobe Epilepsy—Preliminary Study

#### 101.1.1 P. Drobot, PhD Candidate, DVM,^1^ ^,2^ A. Banasik, PhD Candidate, DVM,^1^ ^,2^ L. Brewinska, Neurology Resident, DVM,^1^ ^,2^ K. Owsinska‐Schmidt, PhD Candidate, DVM,^1^ ^,2^ P. Podgórski, MD,^3^ M. Wrzosek, ECVN Diplomate, DVM, PhD, Dr Hab. Prof. UPWr^1^ ^,2^

##### 101.1.1.1 ^1^ Department of Internal Medicine, University of Environmental and Life Sciences, Wroclaw, Poland; ^2^ NeuroTeam Veterinary Speciality Clinic, Wroclaw, Poland; ^3^ Department of General Radiology, Interventional Radiology and Neuroradiology, Wroclaw Medical University, Wroclaw, Poland

Introduction and Aim: Temporal lobe epilepsy (TLE) in humans often has a focal onset which in dogs may go unnoticed. The aim of the study was evaluation of the EEG, MRI, and temporal lobe (TL) volumetry findings in dogs with paroxysmal behavioral disorders (PBD) referred for neurological assessment after behavioral consultation due to poor response to previous management.

Methods: A retrospective analysis of 10 dogs with clinical symptoms of PBD is presented. Analysis included EEG examinations in 10 cases and head MRI based on an epileptic‐specific protocol in 8 cases. Volumetric analysis included manual segmentation and then correlation of the data with the Stereotactic Cortical Atlas of the Domestic Canine Brain using SPM12 and 3D Slicer software. Obtained Jacobian matrix was used in an inverse matching process to match each ROI with the corresponding canine brain.

Results: Among 10 cases interictal EEG revealed epileptic discharges in 6/10 dogs in which 4/6 showed a reduction of seizure frequency above 50% after antiepileptic therapy. Of these 4 dogs, two showed an asymmetric ratio above 6%, where the side of TL reduction correlated with lateral ventricle enlargement and EEG discharges.

Conclusions: Upon the results animals with PBD may be associated with focal epileptic seizures, suggesting that diagnosis in such patients be expanded to include EEG. AR > 6% may be associated with TLE, as indicated by changes in the volumetry of the TL's, but these may also be post‐ictal changes and patients would require a follow‐up MRI scan.

## 102 (P 75)

### 102.1 Therapeutic Effect of Hyaluronic Acid Hydrogel as an Addition to Standard Glucocorticoid Epidural Injection in Dogs With Cauda Equina Syndrome. Preliminary Study

#### 102.1.1 L. Brewinska, Neurology Resident,^1,2^ T. Serzysko, DVM,^2^ P. Drobot, PhD Candidate, DVM,^1^ ^,2^ M. Plociennik, PhD Candidate, DVM,^1^ ^,2^ J. Sobczynski, DVM, M. Wrzosek, ECVN Diplomate, DVM, PhD, Dr Hab. Prof. UPWr^1^ ^,2^

##### 102.1.1.1 ^1^ Department of Internal Medicine, University of Environmental and Life Sciences, Wroclaw, Poland; ^2^ NeuroTeam Veterinary Speciality Clinic, Wroclaw, Poland; ^3^ Bemowo Clinic, Warsaw, Poland

Introduction/Purpose: Cauda equina syndrome (CES) is a common condition affecting canines. CES manifests primarily with pain secondary to degenerative lumbosacral stenosis (DLSS). Apart from surgical techniques, conservative treatment is also available. One of the pharmacological therapies described in dogs are glucocorticoid (GC) epidural injections. The data on epidural use of other therapeutic agents, such as hyaluronic acid (HA), in canines are lacking, despite promising results obtained in humans and rats. HA is well known for its anti‐adhesive, anti‐inflammatory, and lubricating properties. The aim of the study was to evaluate the effectiveness of combined HA and GC when compared with standard GC epidural injections.

Methods: Retrospective review of medical records of dogs diagnosed with CES secondary to DLSS and treated with epidural injections. Twenty‐five dogs met the inclusion criteria and were enrolled in the study. Seven of them were treated with GC epidural injection, 18 obtained GC and HA.

Results: Improvement was seen in 24/25 (96%) dogs with 7/24 (29%) relapsing within 2 months. The recurrence rate within 2 months of treatment initiation was higher in the GC group (Mann–Whitney *U* test [*p* < 0.05]). The long‐term therapeutic effect (> 6 months) was maintained in 4/18 (22%) of patients undergoing GC + HA therapy and in none of the GC group, however the difference was not statistically significant.

Discussion/Conclusion: Addition of HA to standard GC epidural treatment might be beneficial in CES patients in terms of early recurrence of clinical signs.

## 103 (P 76)

### 103.1 Disseminated *Rasamsonia argillacea* Complex Infection Presenting as Intraventricular Hemorrhage Into the Brain in a German Shepherd Dog in Australia

#### 103.1.1 C. Thomson, BVSc (Hons), PhD, DACVIM (Neurol), DECVN,^1^ C. Skinner, BVSc (Hons),^2^ K. Hoffmann, BVSc, MVSc, PhD, Dip Vet Clin Stud, Diplomate ECVDI,^3^ M. Kelly‐Bosna, BVSc,^4^ S. Kidd, PhD,^5^ R. Allavena, BVSc (Hons), BVBiol, MANZCVS, PhD, DACVP^4^

##### 103.1.1.1 ^1^ Animal Referral Hospital, Brisbane, Australia; ^2^ Small Animal Hospital, Glasgow University, Glasgow, UK; ^3^ Imaging Vets, Sydney, Australia; ^4^ School of Veterinary Science, University of Queensland, Gatton, Australia; ^5^ National Mycology Reference Centre, Adelaide, Australia

Disseminated fungal infections can affect the central nervous system in dogs, with German shepherd dogs (GSD) being predisposed. *Rasamsonia argillacea* complex, thus far, have only identified as pathogenic in humans and animals in the northern hemisphere (Europe, North America, and South Korea). Intraventricular hemorrhage has not been reported in fungal encephalitis cases. This is a descriptive case report of both aspects in a GSD. A 7 year old, FN GSD presented acutely with spinal hyperpathia, decreased mentation, and anisocoria. An MRI depicted obstructive generalized hydrocephalus with hemorrhagic sediment in the ventricles and cranial cervical subarachnoid space. Due to progression of signs, the patient was euthanized. Post‐mortem examination revealed multisystemic lesions, characterized by necrotizing, granulomatous vasculitis with intralesional fungal hyphae. The fungus was strongly angiotropic. Samples were sent to the National Mycology Reference Centre in South Australia and, when cultured, revealed penicillus‐like conidiophores. DNA sequencing was performed and compared with the MycoBank and GenBank databases, revealing a 99.8% match to *R. argillacea*. *Rasamsonia argillacea* is an emerging pathogen in human medicine, particularly for immunocompromised patients. *Rasamsonia argillacea* species complex has been reported in in Northern hemisphere dogs in which the German Shepherd breed predominates (7/11 cases). The German shepherd dogs are over‐represented for disseminated mycoses, which may be associated with an inherited abnormality in IgA. The marked angiotropism of this fungus explains the intraventricular hemorrhage in this patient. *Rasamsonia argillacea* is globally distributed and should be considered as a potential etiological cause of fungal encephalitis in dogs, especially when associated with vascular lesions.

## 104 (P 77)

### 104.1 Suspected Hypophysitis in a Boxer Mimicking A Macroadenoma Successfully Treated With Corticosteroids, Cytosine Arabinoside, and Cyclosporine

#### 104.1.1 N. Delgado, DVM, MSc,^1^ S. Moya, DVM, MSc,^1^ J. C. Cartagena, DVM, PhD, MSc,^1^ S. Ródenas, DVM, Dip ECVN^1^

##### 104.1.1.1 ^1^ HV Bluecare Partners, Mijas, Málaga, Spain

Only a few cases of adenohypophysitis have been reported in dogs. The aim of this case report is to describe the clinical signs, MRI features and successful treatment with corticosteroids, cytosine arabinoside and cyclosporin in a dog with suspected adenohypophysitis. A 6‐year‐old female Boxer was presented to the neurology service for apathy and obnubilation. Neurological examination was consistent with a prosencephalic location. Intracranial MRI was revealed a well‐defined rounded extra‐axial lesion at the level of the pituitary fossa measuring 18 mm × 17 mm of 1.8 cm. The mass was hyperintense on T2WI and FLAIR, hypointense on T1WI with marked and homogeneous contrast enhancement. The main differential diagnoses were pituitary macroadenoma and adenohypophysitis. Cerebrospinal fluid tap was not performed because of the owner's request. Prednisolone 0.5 mg/kg BID was prescribed. Six months later, neurological examination was unremarkable. A recheck MRI was performed and revealed a marked reduction of the size of the mass. CSF analysis revealed mild increase in protein concentration (53 mg/dL). ACTH stimulation was normal. Due to adverse effects of the corticosteroids a treatment with cytosine arabinoside was initiated (50 mg/m^2^; SC injection/12 h/2 days, every 3 weeks), cyclosporine 5 mg/kg SID and corticosteroids were tapered progressively. Follow up 3 months the neurological and physical examination were unremarkable but for mild dermatologic signs (calcinosis cutis). Presumptive diagnosis was adenohyphysitis. To the author's knowledge no cases of suspected adenohypophysitis treated successfully with cytosine arabinoside and cyclosporine have been reported.

## 105 (P 78)

### 105.1 A New Neurological Disease in Greyhound, Show Type

#### 105.1.1 J. Hultman, DVM,^1^ K. Matiasek, Dr. Med. Vet. Habil, AM‐ECVN,^2^ E. Dell'Era, DVM, MSc,^2^ B. Kessler, Dr. Med. Vet.,^3^ K. H. Jäderlund, DVM, DipECVN, PhD^1^

##### 105.1.1.1 ^1^ Department of Companion Animal Clinical Sciences, Faculty of Veterinary Medicine, Norwegian University of Life Sciences, Ås, Norway; ^2^ Section of Clinical & Comparative Neuropathology, Institute of Veterinary Pathology, Centre for Clinical Veterinary Medicine, Ludwig‐Maximilians‐Universitaet Muenchen, Munich, Germany; ^3^ Institute of Molecular Animal Breeding and Biotechnology, Gene Centre and Department of Veterinary Sciences, Ludwig‐Maximilians‐Universitaet Muenchen, Munich, Germany

During recent years, neurological signs indicating episodic hyperesthesia in the thoracic limbs was reported in closely related European dogs of the Greyhound breed, show type. Affected dogs seemed painful during the episodes and some of them were euthanized due to this. The aim of the study was to describe the phenotype of affected dogs. Affected and control dogs of the same breed were prospectively included. Inclusion criteria for affected Greyhounds were a minimum of two typical episodes, clinical examination and routine hematology and serum biochemistry. Control Greyhounds should not have had any previous neurological signs. Clinical and neurological examination, MRI, blood analysis and urinalysis were performed in several of the included dogs. Dogs euthanized during the study period were autopsied.

Fourteen affected and 13 control dogs were included. Age at onset varied from 3 months to 9 years. Clinical signs consisted of episodes of frenetic biting of mostly a thoracic limb or paw, simultaneously scratching with the ipsilateral pelvic limb against the thoracic wall and screaming. Each episode lasted from a couple of seconds up to 45 min. Frequency of episodes varied between dogs from once every other year to daily. In between the episodes the dogs were neurologically normal. In vivo diagnostics were unremarkable. Histopathology of affected dogs revealed cervically predominant syringohydromyelia with subsequent dorsal funiculus degeneration. Dogs of the greyhound, show type breed have a neurological disease characterized by a myelopathy, clinically recognized by episodic hyperesthesia of the thoracic limbs which appears painful.

## 106 (P 79)

### 106.1 The Prevalence of Seizures and Sleep Disorders in a Cohort of “Brachycephalic Obstructive Airway Syndrome” (BOAS) Affected Dogs That Underwent Computed Tomography

#### 106.1.1 S. Monforte Monteiro, DVM, MSc,^1^ L. Alves, DVM, PhD^1^

##### 106.1.1.1 ^1^ Department of Veterinary Medicine, School of the Biological Sciences, University of Cambridge, Cambridge, UK

Introduction/Purpose: The prevalence of brain‐related disorders in dogs with brachycephalic airway obstructive syndrome (BOAS) has not been described. Studies in people with obstructive sleep apnea (OSA), the human equivalent to BOAS, have shown higher prevalence of epilepsy, brain tumors, and Alzheimer's compared with the general population. This retrospective study describes the prevalence of seizures and sleep disorders in a cohort of brachycephalic dogs with BOAS.

Methods: Baseline characteristics of the cohort, history of seizure activity and sleep disorders, and BOAS grading data were retrieved from a population of brachycephalic dogs for which BOAS grading is validated (Pugs, English bulldogs, and French bulldogs) from the hospital's database. All dogs presented for BOAS evaluation between 2009 and 2018, and underwent clinical assessment, whole‐body barometric plethysmography and computed tomography (CT).

Results: One hundred forty‐two animals were included. Twelve were described as presenting with seizure activity (8%). Mean BOAS grade for BOAS dogs with seizures was 2.5 (range 1–3), compared with a mean BOAS grade of 2.21 (range 1–3) in BOAS dogs without seizures. Eighty‐two of 142 dogs (58%) had sleep disorders, including 7 of 12 dogs (58%) with seizures.

Discussion/Conclusion: Brachycephalic dogs with BOAS have a higher prevalence of seizures compared with the reported prevalence of seizures for these breeds. This association between BOAS‐induced chronic hypoxia and seizures/sleep disorders being higher than reported represents crucial information to calculate significant sample size in prospective studies, necessary to clarify underlying pathophysiological mechanisms.

## 107 (P 80)

### 107.1 Do Not Give Up on Old Brain Samples—Modified Golgi Staining Reveals Neuronal Details After Long‐Time Fixation

#### 107.1.1 C. Kaufhold, DVM (Equ),^1^ K. Matiasek, DrMedVetHabil FTA Path&Neuropath AM‐ECVN^1^

##### 107.1.1.1 ^1^ Section of Clinical & Comparative Neuropathology, LMU Munich, Munich, Germany

Diagnosis of developmental and maladaptive changes in the brain may require staining of dendrite details, including that of dendritic spines. These structures have been highlighted traditionally by Golgi staining on fresh tissue. As clinical samples often undergo formalin fixation, this study was initiated to allow for evaluation of dendritic specificities in archived material. Common Golgi protocols were modified (mRGS) for use in fixed brains by postfixation, pH‐adaption, agar‐embedding and optimized incubation times. Thereafter sections of cerebellum and hippocampus were cut, and cells of interest (COI) were counted per cerebellar folium and cornu ammonis segment. Results were compared with conventional rapid Golgi stain on fixed tissue (fRGS).

Altogether, 112 specimens were generated, 106 of which stained successfully. Representation of folia (43.9 ± 3.9 vs. 33.0 ± 3.3) and yield of stained COI (75.5 ± 15.7 vs. 35.3 ± 8.2) were significantly better on mRGS (*p* = 0.05). Likewise, mRGS stained hippocampi were significantly better preserved (*p* = 0.04). Within preserved samples, COI density was within the same range between both groups (5.68 ± 1.04 vs. 4.94 ± 0.81; *p* > 0.05). Age of the individual and immersion times (1 day to 89 months) had no influence on the outcome. The mRGS developed herein allows for detailed staining of neuronal dendrites within cerebellum and hippocampus even after several years of formalin fixation. Postfixation maneuvers further preserve the tissue architecture significantly better than hitherto published techniques and the staining procedure is much quicker. Hence, archived material readily.

## 108 (P 81)

### 108.1 The Idiopathic Epilepsy Mystery: A Multimatrix Metabolomics Approach

#### 108.1.1 F. Verdoodt, MVetMed,^1^ ^,2,3^ L. van Hemeryck, PhD,^2^ L. Vanhaecke, Prof.,^2^ L. van Ham, Prof.,^3^ M. Hesta, Prof.,^1^ S. Bhatti, PhD^3^

##### 108.1.1.1 ^1^ Equine and Companion Animal Nutrition, Department of Morphology, Imaging, Orthopedics, Rehabilitation and Nutrition, Faculty of Veterinary Medicine, Ghent University, Merelbeke, Belgium; ^2^ Laboratory of Integrative Metabolomics, Department of Translational Physiology, Infectiology and Public Health, Faculty of Veterinary Medicine, Ghent University, Merelbeke, Belgium; ^3^ Small Animal Department, Faculty of Veterinary Medicine, Ghent University, Merelbeke, Belgium

Better understanding of pathways involved in canine idiopathic epilepsy (IE) is primordial to improving its management. An untargeted metabolomics approach was therefore applied to study differences in fecal and plasma metabolome of healthy dogs (*n* = 39) versus dogs with IE Tier I or II (De Risio et al., 2015) (*n* = 50) on the same maintenance diet for at least 21 days. Dogs with IE were classified as drug‐sensitive (*n* = 23) or drug‐resistant (*n* = 27) (Potschka et al., 2015). Samples were analyzed using liquid chromatography coupled to mass spectrometry (UHPLC‐HRMS) (De Paepe et al., 2018). Data were normalized, log‐transformed and Pareto scaled before multivariate modeling (OPLS‐DA). The fecal and plasma metabolome of healthy dogs could be significantly discriminated from that of dogs with IE (feces: R2y = 0.95, Q2 = 0.57, CV‐ANOVA *p* < 0.05; plasma: R2y = 0.89, Q2 = 0.60, CV‐ANOVA *p* < 0.05) and healthy dogs from dogs with drug‐resistant IE (feces: R2y = 0.99, Q2 = 0.70, CV‐ANOVA *p* < 0.05; plasma: R2y = 0.91, Q2 = 0.77, CV‐ANOVA *p* < 0.05). Drug‐sensitive IE could only be discriminated from healthy dogs in the plasma metabolome (R2y = 0.98, Q2 = 0.55, CV‐ANOVA *p* < 0.05). From these models, discriminative metabolic features were derived and putatively annotated, including coenzyme Q10, which was lower in the plasma of dogs with IE. This study is the first to describe the metabolome of dogs with IE in feces and plasma, taking into account drug sensitivity and dietary influence. Further data mining will allow confident identification of driver metabolites and elucidation of pathways involved.

## 109 (P 82)

### 109.1 Clinical Features, Treatment, and Outcome of Juvenile Dogs With Meningoencephalitis of Unknown Etiology

#### 109.1.1 J. Galer, BVetMed, MRCVS,^1^ A. Forward, BVSc, MSc, MVetMed, DipECVN, MRCVS,^1^ J. Hughes, BVetMed (Hons), DipECVDI, MRCVS,^1^ A. Crawford, BVM&S, BSc, PhD, DipECVN, MRCVS,^2^ S. Behr, DVM (Hons), DipECVN, MRCVS,^3^ G. Bruto Cherubini, DVM, DipECVN FRCVS,^4^ ^,5^ I. Cornelis, DVM, PhD, DipECVN, EBVS,^6^ E. Royaux, DVM, DipECVN, MRCVS^1^

##### 109.1.1.1 ^1^ Davies Veterinary Specialists, Hitchin, UK; ^2^ Department of Clinic Science and Services, Queen Mother Hospital for Animals, Royal Veterinary College, Potters Bar, UK; ^3^ Willows Veterinary Centre, Solihull, UK; ^4^ Department of Veterinary Sciences, University of Pisa, Pisa, Italy; ^5^ Dick White Referrals, Newmarket, UK; ^6^ Small Animal Department, Faculty of Veterinary Medicine, Ghent University, Ghent, Belgium

Meningoencephalitis of unknown etiology (MUA) can affect any breed and age of dog. The youngest previously reported age is 4 months old, while an age of older than 6 months is typically used as a cut off for inclusion criteria in studies on MUA. The clinical presentation, diagnostic findings, treatment, and outcome has not been reported specifically for juvenile dogs. The aim of this multicenter retrospective study was to describe these findings in a population of dogs less than 12 months old. After searching the records of 1 Belgian and 4 UK referral centers, 38 dogs met the inclusion criteria with a mean age of 32 weeks (SD 11 weeks), 12 (32%) of which were < 6 months old at presentation. Clinical presentation was highly variable but an altered mentation (71%), ataxia (50%), and seizures (31%) were the most common presenting complaints. The neuroanatomical localizations were forebrain (37%), multifocal (26%), brainstem (24%), and cerebellum (13%). Immunosuppressive corticosteroid monotherapy was used in 45% of cases, corticosteroid plus cytarabine in 40% of cases, and varying combinations of immunosuppressive medications in the remainder. Five dogs (13%) did not survive to discharge. Follow up data was available for 28 cases, 10 dogs (36%) were alive at the time of writing. Of the 18 dogs that died, 17 died or were euthanized because of MUA. Kaplan–Meier survival analysis demonstrated a median survival time for all‐cause mortality of 104 days. The prognosis for MUA in this subset of dogs remains guarded.

## 110 (P 83)

### 110.1 Occurrence of Osteoradionecrosis in a dog After Surgical Removal and Adjunctive Radiation Therapy for a Spinal Nephroblastoma

#### 110.1.1 N. Burger, DVM,^1^ A. Spillebeen, DVM, DECVS,^1^ J. Benoit, DVM, DECVDI, ACVR‐RO, MRCVS,^2^ E. Stock, DVM, PhD, DECVDI,^3^ I. Cornelis, DVM, PhD, DECVN,^1^ K. Stee, DVM, DECVN,^1^ S. Bhatti, DVM, PhD^1^

##### 110.1.1.1 ^1^ Small Animal Department, Faculty of Veterinary Medicine, Ghent University, Merelbeke, Belgium; ^2^ Oncovet, Avenue Paul Langevin, 59 650, Villeneuve d'Ascq, France; ^3^ Department of Morphology, Imaging, Orthopedics, Rehabilitation and Nutrition, Faculty of Veterinary Medicine, Ghent University, Merelbeke, Belgium

Nephroblastoma is a rare spinal neoplasia diagnosed in young dogs, occurring as a solitary intradural‐extramedullary mass, between T10 and L2 vertebrae. Radiation therapy following cytoreductive surgery is associated with improved neurologic function and longer survival times compared with palliative treatment. A 5‐year‐old female entire American Staffordshire Terrier presented with progressive paraparesis and ataxia, suggestive for a T3–L3 myelopathy, 4 years after the surgical excision of a histopathologically confirmed T12 spinal nephroblastoma that was additionally treated with external beam radiation therapy (total of 5000 cGy over 20 fractions of 250 cGy each) 3 months post‐surgery. CT of the thoracolumbar vertebral column showed hypoattenuating lesions in the spinous process and vertebral body of T11. Spinal MRI revealed an intradural, homogenous, rounded T2W‐hyperintense, and T1W‐hypointense lesion at T12. CT‐guided biopsies of T11 vertebra confirmed extensive osteonecrosis, without neoplastic features. The vertebral column was successfully stabilized using two locking reconstruction plates fixated on the vertebral spinous processes under distraction (T7–L1) and left‐sided at the region of the pedicles (T8–L1), resulting in significant postoperative improvement. Six months post‐operatively the dog presented again with acute pelvic limb paresis and ataxia. CT of the thoracolumbar vertebral column showed progressive hypoattenuating lesions of the T10 and T11 spinous processes and T11 vertebral body, with no indications of implant failure. Considering the poor prognosis, the dog was euthanized. Osteoradionecrosis is a severe but rare complication of radiation therapy in dogs. This is the first case report describing the occurrence of vertebral osteoradionecrosis in a dog 4 years after surgical removal with adjunctive radiation therapy of a spinal nephroblastoma.

## 111 (P 84)

### 111.1 Anchored Intervertebral Titanium Device (C‐LOX) and Combined Dorsal Transarticular Screws in 5 Dogs With Caudal Cervical Spondylomyelopathy: A Case Series

#### 111.1.1 F. Iocca, DVM,^1^ R. Lombardo, ECVN, ACVN,^1^ F. Cozzi, ECVN,^1^ M. Bonaldi, DVM,^1^ M. Baccolini, Resident ECVN^1^

##### 111.1.1.1 ^1^ Neurologi Veterinari Associati (NVA), Milano, Italia

Objective: To report clinical outcome of six dogs treated for caudal cervical spondylomyelopathy (CCSM) with anchored intervertebral titanium device (C‐LOX) and dorsal transarticular screws.

Study Design: Short case series.

Animals: Five large breed dog (1 Dobermann; 1 Rottweiler; 3 Weimaraner).

Methods: In all the dogs was performed neurologic examination, MR of cervical spinal cord in neutral and post‐traction position, radiography of the neck, surgery (ventral distraction‐stabilization with C‐LOX system and dorsal transarticular screw), immediately post‐surgical MRI and radiography and 1 month post‐surgical radiography an clinical follow up. Clinical or in some cases telephonic follow up 3/6 month after surgery was performed.

Results: In all five dogs there was a neurological improvement (according to neurologic scale from Grade 1 to Grade 5) at 2 weeks post op clinical evaluation; at 1 month post‐op radiogram we observed in one case breaking of one caudal screw and in one case there was a breaking of the caudal portion of the CLOX device with consequent mild migration of two screw; at 1 month clinical follow up we had not observed deterioration of neurological condition; we observed subsidence in all 5 cases but more evident in the two cases with partial implant failure.

Conclusion: we observed clinical improvement of all dogs despite the evidence of subsidence/break of screws. The limit of this case series was the small number of cases, the shortly time follow up and the application of two different type of C‐LOX system.

## 112 (P 85)

### 112.1 Fecal Microbial Transplantation and Microbiota Analysis in a Cavalier King Charles Spaniel With Drug‐Resistant Idiopathic Epilepsy

#### 112.1.1 L. Ledeganck, DVM,^1^ F. Verdoodt, DVM,^2^ E. Goossens, DVM,^3^ M. Hesta, DVM, PhD, Dip ECVCN, Prof,^2^ L. van Ham, DVM, PhD, Dipl ECVN, Prof,^1^ S. F. M. Bhatti, DVM, PhD^1^

##### 112.1.1.1 ^1^ Small Animal Department, Faculty of Veterinary Medicine, Ghent University, Merelbeke, Belgium; ^2^ Department of Morphology, Imaging, Orthopedics, Rehabilitation and Nutrition, Faculty of Veterinary Medicine, Ghent University, Merelbeke, Belgium; ^3^ Department of Pathobiology, Pharmacology and Zoological Medicine, Faculty of Veterinary Medicine, Ghent University, Merelbeke, Belgium

The management of dogs with idiopathic epilepsy (IE) remains challenging. Therefore, targeting the microbiota–gut–brain axis via a fecal microbial transplantation (FMT) in a canine IE patient with a severe and drug‐resistant (DRE) phenotype was performed. A 1.5‐year‐old female entire Cavalier King Charles Spaniel was diagnosed with IE (Tier II) resistant to phenobarbital/potassium bromide and an MCT‐included diet. The dog had a mean seizure frequency (SF) of 1.7 ± 1.1 seizures/week and was given FMT through naso‐esophageal route. Follow‐up management included oral administration of enteric coated capsules with lyophilized feces from the same donor, starting 3 weeks after the initial FMT, for a period of 12 weeks. Fecal samples from the receptor were collected before (R0), 3 weeks (R1), and 6 weeks after FMT (R2) for 16sDNA sequencing. From R0 till 25 weeks after FTM, the mean seizure frequency decreased to 1.3 ± 0.9 seizures/week (*p* = 0.0359). Alpha diversity, measured with Chao1 index, was higher in the donor sample (153) compared with the samples from the receptor at all timepoints (101–118). In our receptor dog, at the family level, the relative abundance of Bacteriodaceae and Lactobacillaceae decreased markedly, while Prevotellaceae, Ruminococcaceae, and Selenomonadaceae increased markedly at R1 and R2. Similar to the DRE dogs in a recent pilot study (Garcia‐Belenguer, 2023) our receptor dog showed a higher relative abundance of the genera Bacteroides at R0 (32.67%), which markedly decreased at R1 (11.37%) and R2 (4.75%). In conclusion, this case report indicates that FMT may alter the baseline microbiota composition in dogs with DRE. Future directions include studies in a larger dog population and the measurement of bacterial metabolites in feces and/or serum as markers for the appropriate function of the microbiome.

## 113 (P 86)

### 113.1 Clinical Response to PEGylated L‐Asparaginase in a Case of Feline Spinal T‐Cell Lymphoma

#### 113.1.1 L. Ledeganck, DVM,^1^ A. Krupa, DVM, PhD,^1^ I. Cornelis, DVM, PhD, DiplECVN,^1^ K. Stee, DVM, DiplECVN,^1^ E. Stock, DVM, PhD, DiplECVDI,^2^ S. F. M. Bhatti, DVM, PhD^1^

##### 113.1.1.1 ^1^ Small Animal Department, Faculty of Veterinary Medicine, Ghent University, Merelbeke, Belgium; ^2^ Department of Morphology, Imaging, Orthopedics, Rehabilitation and Nutrition—Faculty of Veterinary Medicine, Ghent University, Merelbeke, Belgium

Feline spinal lymphoma can be managed by different treatment protocols, no clear treatment modality has been proven superior so far. An 11‐year‐old male castrated Siamese cat was presented with a 1‐week history of progressive hind limb weakness and incontinence. Physical examination, CBC and biochemistry did not reveal any significant abnormalities. Neurological examination confirmed ambulatory paraparesis, spinal ataxia and decreased withdrawal reflexes suggestive of a L4–S3 myelopathy. Spinal CT showed an extramedullary, elongated, well‐delineated, heterogeneously contrast‐enhancing mass within the right iliocostalis lumborum muscle, invading the vertebral canal through the right L5‐L6 intervertebral foramen and extending from L4 to L7 and an additional left‐sided extramedullary elongated mass extending from L1 to L3. Ultrasound‐guided Tru‐cut biopsy (16G) from the paravertebral muscles of right lumbar region showed an intermediate‐grade peripheral T‐cell lymphoma, not otherwise specified. The cat was given cyclophosphamide, vincristine and prednisolone (COP protocol), but showed only mild neurological improvement the next 4 weeks while developing constipation and loss of appetite. A follow‐up spinal CT revealed complete remission of the mass at L4–L7 and partially remission of the second mass at L1–L3, but an additional extramedullary mass was seen at the right intervertebral foramina of L6–L7 invading the vertebral canal. Chemotherapy was discontinued and treatment with PEGylated‐L‐asparaginase was commenced, which resulted in rapid improvement and complete clinical remission. Six months later, the cat is still asymptomatic. This report suggests that a combination of prednisolone and PEGylated L‐asparaginase may be a very effective treatment for feline spinal lymphoma.

## 114 (P 87)

### 114.1 “Pediatric” Gliomas in Dogs

#### 114.1.1 A. Oevermann, Prof., Dr., DipECVP,^1^ N. Shihab, MA, VetMB, DipECVN, MRCVS,^2^ D. Schweizer, Prof., Dr., DipECVDI,^3^ A. Maiolini, PhD, DipECVN^4^

##### 114.1.1.1 ^1^ Division of Neurological Sciences, Department of Clinical Research and Veterinary Public Health, Vetsuisse Faculty, University of Bern, Bern, Switzerland; ^2^ Southern Counties Veterinary Specialists, Hampshire, UK; ^3^ Division of Clinical Radiology, Department of Clinical Veterinary Medicine, Vetsuisse Faculty, University of Bern, Bern, Switzerland; ^4^ Division of Clinical Neurology, Department of Clinical Veterinary Medicine, Vetsuisse Faculty, University of Bern, Bern, Switzerland

Introduction: In contrast to the occurrence of pediatric gliomas in humans, gliomas in puppies have been very rarely reported. The aim of this study is to describe the clinicopathological features of “pediatric” canine gliomas based on a case series of six dogs < 1 year of age.

Methods: Clinicopathological data of six dogs < 1 year of age diagnosed with glioma were collected and analyzed.

Results: All 6 dogs were purebred (Boxer, Doberman, Newfoundland, Cavalier King Charles Spaniel, Jack Russel Terrier, Chihuahua) and the age at first presentation ranged from 5 to 9 months. The most commonly reported clinical signs were ataxia, cranial nerve deficits, obtundation, recumbency and reduced proprioception. Individual dogs exhibited pyrexia, strabismus, nystagmus, opisthotonos, abnormal behavior, epileptic seizures, facial twitching, and pain. Five tumors were anaplastic oligodendrogliomas, 1 anaplastic astrocytoma. On MRI and neuropathology, three oligodendrogliomas appeared to originate near the midline (septum/septal nuclei) and extended extensively into the lateral ventricles. One oligodendroglioma occurred in the brainstem and another in the temporal lobe. The astrocytoma extended from the midbrain to the thalamus.

Discussion: The high proportion of anaplastic oligodendrogliomas in puppies reflects the high prevalence of oligodendrogliomas in adult dogs. Known predisposed breeds were not overrepresented in this puppy population. Puppy gliomas occurred in the caudal brain structures and near the midline rather than in adult glioma predilection sites (frontal, parietal, and temporal lobes). These observations suggest differences in oncogenesis between childhood and adult gliomas in dogs.

## 115 (P 88)

### 115.1 Anatomical Description of the Ligamentous Supporting Structures of the Canine Cranial Cervical Spinal Cord

#### 115.1.1 A. Nagendran, BVSc, DipECVN, MRCVS,^1^ M. Pumarola Battle, DVM, Dip. ECVP,^2^ V. Aige Gil, DVM, PhD^2^

##### 115.1.1.1 ^1^ Department of Clinical Sciences, College of Veterinary Medicine, North Carolina State University, Raleigh, North Carolina, USA; ^2^ Facultad de Veterinaria, Universidad Autónoma de Barcelona, Barcelona, Spain

Introduction: The cranial cervical vertebral column carries a unique range of mobility with the addition of dorsal and ventral flexion to lateral flexion and rotation. The denticulate ligament provides a support and protection of the spinal cord, but little is known about the adaption of this apparatus at the cranial cervical portion.

Methods: Vertebral columns removed from neurologically normal dogs and dissected for gross and histological examination. Magnetic resonance imaging was retrospectively evaluated in dogs to assess topography of relevant areas.

Results: All examined specimens had paired isolated dorsolateral attachments at the level of C1–C2 spinal cord; connecting dura mater, through the subarachnoid space, to the pia mater. Histological staining of the dorsolateral attachments was consistent with collagen. The cranial portion of the denticulate ligament runs caudo‐ventrally from the dura mater at the level of the atlas to the pia mater of the spinal cord between the C2 and C3 spinal cord segments.

Discussion: We have identified a pair of dorsolateral ligaments suspending the spinal cord within the meninges at the level of C1–C2 spinal cord segments. We suspect that this new discovery may provide dorsal support to the spinal cord, in the near absence of strong lateral support by the denticulate ligamentous structures at this level, especially in dorsal and ventral flexion of the head and neck. Such ligamentous structures, alongside nerve rootlets, may also play a role in cerebrospinal fluid dynamics of the cervical spinal cord. Further studies are necessary to determine further anatomical variances in this area.

## 116 (P 89)

### 116.1 Characterization of Post‐Ictal Signs in Dogs With Idiopathic Epilepsy: A Questionnaire‐Based Study

#### 116.1.1 A. Nagendran, BVSc, DipECVN, MRCVS,^1^ J. Nettifee, BS, RVT, VTS,^1^ D. Carter, DVM student,^1^ K. Muñana, DVM, MS, DACVIM (Neurology)^1^

##### 116.1.1.1 ^1^ Department of Clinical Sciences, North Carolina State University, Raleigh, North Carolina, USA

Introduction: Post‐ictal (PI) clinical signs are a key defining stage of seizure manifestation in dogs. However, this phase remains an enigma. The aim of our study was to further characterize PI signs, evaluate how they relate to other parts of the seizure and understand owners' perception on how PI signs affect the quality of life of the dog.

Methods: An online questionnaire was formulated and distributed to clients of dogs at the time of diagnosis (2021–2022), or historically diagnosed, with a Tier II level of confidence of idiopathic epilepsy. Recipients voluntarily completed the questionnaire. Forms were excluded if incomplete.

Results: Eighty‐six patients met inclusion. PI signs were identified in 77/86 (90%) dogs. Median duration of PI signs was 30 min (range 5–4320 min). The most common PI signs reported were disorientation (50/77) and unsteadiness (49/77). There was no significant relationship between the presence of pre‐ictal signs and presence of PI signs [*p* = 0.111], or between ictal duration and PI duration [*p* = 0.285]. PI duration was shown to be significantly associated with change in seizure frequency [*p* < 0.001] and duration [*p* < 0.001]. The administration of benzodiazepines was associated with a significant change in severity of PI signs [*p* = 0.037]. Compared with ictal signs, the impact of PI signs on quality of life was considered similar in 37 dogs, greater in 13 dogs and less in 27 dogs.

Discussion: This study sheds light on the characterization and relevance of PI signs and how it may impact the overall quality of life of the idiopathic epileptic patient.

## 117 (P 90)

### 117.1 Correlation Between Semiautomated Volumetry of Cingulate Gyrus and EEG in Dogs With Idiopathic Epilepsy—Preliminary Study

#### 117.1.1 A. Banasik, DVM,^1^ P. Drobot, DVM,^1^ P. Podgórski, MD,^2^ K. Owsinska‐Schmidt, DVM,^1^ A. Zimny, MD, Dr Hab., Wroclaw Medical University Associate Professor,^2^ M. Wrzosek, DVM, Dr Hab., Dipl. ECVN, UPWr Associate Professor^1^

##### 117.1.1.1 ^1^ Department of Internal Diseases With a Clinic for Horses, Dogs and Cats, Faculty of Veterinary Medicine, Wroclaw University of Environmental and Life Sciences, Wroclaw, Poland; ^2^ Department of General and Interventional Radiology and Neuroradiology, Wroclaw Medical University, Wroclaw, Poland

Cingulate gyrus (CG) was identified as an area that could incur seizure‐induced changes. The aim of this study was to find a correlation between volumetric assessment of CG and EEG findings in dogs with idiopathic epilepsy (IE). It was a retrospective study. Twenty dogs in the study group (10 with EDs in T3, C3 leads, and 10 with EDs in T4, C4 leads), mean 34.9 months of age, mean 21 kg and 13 dogs in the control group were qualified for the research. All animals underwent the same study protocol that included a clinical, neurological examination, blood, and urine testes followed by EEG and MRI examination. To assess the volumetry of CG and process the data MRIcron, SPM12 and 3D Slicer software were used. The obtained Jacobian matrix was employed in an inversive fitting process to fit each region of interest to the appropriate dog brain. Statistical analysis showed significant differences between the volumes of the right and left CG in dogs with EDs in T3, C3 leads (*p* = 0.02) and with EDs in T4, C4 leads (*p* = 0.04). It also has been found that animals with EDs in T3, C3 had significantly smaller left cingulate, and animals with EDs in T4, C4 had smaller right cingulate volumes, correlating with EEG discharge side. The control group showed no statistically significant differences in right and left CG volume values (*p* = 0.74). Research suggests that there are changes in the volume of the CG in the course of IE in dogs, which correlates with the side of EDs.

## 118 (P 91)

### 118.1 Paroxysmal Motor Disorder During Sleep: Use of Video Electroencephalography to Discriminate Between Epileptic or Non‐Epileptic Event in 3 Cats and 1 Dog

#### 118.1.1 E. Lyon, DVM,^1^ ^,2^ T. Troupel, DVM, Dip ECVN,^4^ N. Van Caenegem, DVM,^4^ H. Pochat, CEO,^3^ S. Besnard, MD, PhD,^2^ S. Blot, DVM, PhD, Dip ECVN,^4^ C. Escriou, DVM, PhD^1^

##### 118.1.1.1 ^1^ Unité de Neurologie, CHUVAC, VetAgro Sup, Marcy l'Etoile, France; ^2^ Vertex Laboratory, UFR STAPS Department, Caen, France; ^3^ Amset Medical, Saint‐Maur‐Des‐Fossés, France; ^4^ Unité de Neurologie, CHUV‐AC, ENVA, Maisons‐Alfort, France

The diagnosis of paroxysmal events in sleep represents a significant challenge for the clinician, with the distinction of sleep‐associated epilepsy from non‐epileptic sleep disorders often the primary concern. In humans medicine non‐epileptic motor disorders of sleep are common and take a wide variety of forms grouped into parasomnias, sleep‐related movement disorders, and “others.” To date, in veterinary medicine non‐epileptic motor disorders of sleep are poorly described and characterized. We report a series of 4 cases (3 cats and 1 dog) presented for recurrent paroxysmal motor disorder during sleep where video electroencephalography (EEG) was a decisive and discriminating factor. Video EEG was performed with gel electrodes in a routine context without anesthesia, in presence of owners during 35–76 min with 8 EEG gel electrodes and 2 reference electrodes. Photic stimulation was performed at the beginning of the 4 exams. Spontaneous and natural drowsiness and sleep were obtained for all animals. Paroxysmal events identical to those observed at home by owners were observed during the 4 records. One cat and 1 dog showed epileptic patterns with spikes and polyspikes on the EEG leading to the diagnosis of epilepsy. For 2 cats paroxysmal motor event was observed during rapid eye movement sleep (attested by the EEG) without any epileptic pattern leading the diagnosis to rapid eye movement sleep behavior disorder. Our case series illustrates rapid eye Movements sleep behavior disorder in two cats and the utility of non‐anesthetized EEG with natural sleep recording to discriminate between epileptic from non‐epileptic sleep paroxysmal events.

## 119 (P 92)

### 119.1 Myelinolitic Leukodystrophy in a Cat

#### 119.1.1 R. Fusté, DVM,^1^ M. Pumarola, DVM, PhD, Dip. ECVP,^2^ S. Ortiz, DVM, Board Elegible ECVN,^1^ J. González‐Caramazana, DVM,^1^ M. Al‐Abrash, DVM,^1^ T. Heredia, DVM,^1^ S. Ródenas, DVM, Dip. ECVN^3^

##### 119.1.1.1 ^1^ Anicura Valencia Sur Veterinary Hospital, Valencia, Spain; ^2^ Unit of Murine and Comparative Pathology (UPMIC), Department of Animal Medicine and Surgery, Faculty of Veterinary, Autonomous University of Barcelona, Barcelona, Spain; ^3^ Referral Hospital Animal Bluecare, Malaga, Spain

Leukodystrophy is a defined heritable disorder that affects the white matter of the CNS. This report describes the clinical signs, histopathological, and MRI findings of a cat with myelinolytic leukodystrophy (ML). A 2‐month‐old female shorthair cat was referred to the Neurology service due to a history of progressive cerebellar ataxia, generalized tremors, blindness, and epileptic seizures since birth. The neurological examination was consistent with a multifocal localization affecting the forebrain, cerebellum, and brainstem. Physical examination, blood tests, abdominal ultrasound, thoracic radiography, and CSF analysis, including PCR for infectious diseases, were unremarkable, except for a positive result for feline leukemia virus. Brain MRI showed widened cerebral sulci, hydrocephalus, and cerebellar atrophy. Due to the worsening of neurological signs, the animal was euthanized. The histological examination revealed bilateral and symmetrical pallor in the cortical cerebral white matter, affecting the corona radiata of the frontal, parietal, and occipital areas, as well as the white matter of the cerebellar cortex. The affected areas exhibited hypercellularity, hypertrophic astrocytes, and disorganized nerve fibers without myelin sheath. Adjacent cortical white matter showed moderate pallor and abundant astrocytes. All these findings were confirmed by immunohistochemical techniques. This report describes a case with ML due to a suspected defect of oligodendrocytes responsible for the absence of myelin. Toxic agents should not be ruled out. To our knowledge, this is the first case report describing the clinical signs, MRI, and neuropathological findings in a cat with ML. Further cases and studies are necessary to better understand this pathology in cats.

## 120 (P 93)

### 120.1 Paroxysmal Exertion‐Induced Dyskinesia as a Clinical Sign of Tick‐Born Encephalitis in Dog: Case Report

#### 120.1.1 M. Bajtoš, Intern,^1^ P. Šrenk, Dipl. ECVN,^1^ M. Prikryl, ECVN resident^1^

##### 120.1.1.1 ^1^ VETINO Jaggy Prague, Prague, Czech Republic

The objective of this case report is to describe paroxysmal exertion‐induced dyskinesia as a rare clinical sign of tick‐born encephalitis in a dog. Paroxysmal exertion‐induced dyskinesia (PED) is characterized by episodes of involuntary, abnormal self‐limiting movement triggered by physical activity. A 2 years old Australian shepherd male was presented because of 5 days lasting hind limb incoordination, weakness, episodic cramps and one generalized tonic–clonic seizure based on the owner ´s description. Clinical examination revealed only mildly enlarged popliteal lymph nodes. Neurological examination was normal at rest but showed exercise induced impairment of gait pattern starting with mild hind limb stiffness, progressing to mild generalized ataxia, and exercise‐induced thoracolumbar dystonia with dyskinetic movements within hind limbs. After a short period, the dog recovered to nearly normal gait. Neuroanatomical localization was presumed to be multifocal, intracranial involving forebrain, brainstem, and cerebellum. Serum biochemistry (including TT4, CRP), hematology and a low‐field brain MRI (0.2 T) were unremarkable. CSF analysis detected mild mononuclear pleocytosis and was further evaluated for infectious diseases, which detected a TBE IgG‐Ab elevation along with IgA and CRP. Initially, patient was treated with phenobarbital due to seizure activity and was discharged. After receiving the results, low dose prednisone protocol was initiated. After 9 months, none concerns were reported and the patient continues with sole anticonvulsive treatment. Involuntary movement disorders are uncommon clinical presentation even in humans with CNS inflammatory conditions such as multiple sclerosis, however both PKD and PNKD have been described.

## 121 (P 94)

### 121.1 Brain Features in Sodium Imbalance: Magnetic Resonance and Histopathologic Findings in Four Dogs With Hypernatremia

#### 121.1.1 J. González‐Caramazana, DVM,^1^ T. Heredia, DVM,^1^ M. Pumarola, DVM,^2^ S. Ortiz, DVM, Board Elegible ECVN,^1^ J. J. Sánchez, DVM,^4^ S. Ródenas, DVM, Dip ECVN^3^

##### 121.1.1.1 ^1^ AniCura Valencia Sur Veterinary Hospital, Valencia, Spain; ^2^ Unit of Murine and Comparative Pathology (UPMiC), Department of Medicine and Surgery, Faculty of Veterinary, Autonomous University of Barcelona (UAB), Barcelona, Spain; ^3^ Blue Care Veterinary Hospital, Málaga, Spain; ^4^ La Rambla 7 Palmas, Las Palmas, Spain

Hypernatremia is a life‐threatening event caused by water and sodium imbalances. Aim of this paper is to describe magnetic resonance imaging (MRI) findings, histopathologic findings, and long‐term prognosis in patients with neurologic signs associated with hypernatremia. Four adult dogs were evaluated for the appearance of neurological signs associated with severe hypernatremia (sodium > 180). Four patients presented with obtunded mental status and three of them with epileptic seizures (ES). MRI was performed (pre and post contrast T1W, T2W, FLAIR, and STIR sequences) in all cases, showing bilateral and symmetrical hyperintense lesions in T2W and FLAIR without contrast enhancement affecting in all cases pyriform lobes, thalamus, and caudate nucleus. Frontal and parietal lobes were also affected in two cases. Three patients were euthanized, and histopathology was performed in one case determining necrotizing polyencephalomalacia, probably secondary to metabolic process. One case was discharged with follow‐up MRI 6 months later without neurological signs in which bilateral symmetrical lesions were still observed in both frontal lobes without more lesions. To the author's knowledge, changes in the brain related to sodium disturbance have been described in cases of rapid correction of hyponatremia. Changes in cases in which patients had a rapid increase in sodium, being within the normal range initially are here presented. The lesions pattern raises the question if some of these features were also postictal changes associated with the metabolic imbalance. However, the changes observed in patient without ES and in the follow‐up, MRI support the hypothesis of changes associated with sodium mismatches.

## 122 (P 95)

### 122.1 New Case of Viral Encephalomyelitis in a Dog Positive for Rustrela Virus

#### 122.1.1 E. Dell'Era, DVM (Equ),^1^ S. Engelhardt, DVM (Equ),^2^ M. Gentil, DrMedVet,^3^ D. Rubbenstroth, DrMedVetHabil,^4^ K. Matiasek, DrMedVetHabil^1^

##### 122.1.1.1 ^1^ Section of Clinical & Comparative Neuropathology, Centre for Clinical Veterinary Medicine, LMU, Munich, Germany; ^2^ Tierklinik Dresdner Heide, Dresden, Germany; ^3^ LABOKLIN Labor für Klinische Diagnostik GmbH & Co. KG, Bad Kissingen, Germany; ^4^ Institute of Diagnostic Virology, Friedrich‐Loeffler‐Institut, Greifswald‐Insel Riems, Germany

Rustrela virus (RusV) has been detected in diverse mammals with meningoencephalomyelitis, but never reported in Canidae. Herein, we present the first dog confirmed with encephalomyelitis caused by RusV. A 2‐year‐old female Malinois from eastern Germany was treated for a suspected immune‐mediated chronic encephalomyelitis using corticosteroids with partial response. Upon deterioration, the owners elected euthanasia and consented to full postmortem examination. Histopathology of brain and spinal cord examination revealed a multifocal, polio‐predominant angiocentric encephalomyelitis with extensive lymphoproliferation, multifocal neuronal degeneration, and neuronophagia, suggestive of neuronotropic pathogens.

Immunohistochemical testing for CD3 and CD20 revealed a mixed inflammatory/reactive pattern. On examination for regional pathogens, the tissue tested positive for RusV RNA and antigen. RusV has been demonstrated to cause meningoencephalitis in multiple placental zoo mammals and more recently it has been confirmed to infect cats suffering from staggering disease. The distribution of infected animals appears to be associated with the occurrence of the suspected reservoir hosts, wild yellow‐necked field mouse, and wood mouse. This is the first report of an infected canid and should lead to the inclusion of RusV as a candidate infectious agent when investigating inflammatory CNS diseases in dogs.

## 123 (P 96)

### 123.1 Amantadine for the Neuropathic Pain due to Degenerative Lumbosacral Stenosis in Canine Patients: Preliminary Results

#### 123.1.1 C. Caterino, DVM, PhD,^1^ G. Della Valle, DVM, PhD, RTDB,^1^ F. Aragosa, DVM, PhD,^1^ G. Fatone, DVM, PhD, Associate Professor^1^

##### 123.1.1.1 ^1^ Department of Veterinary Medicine and Animal Production, University of Naples “Federico II,” Naples, Italy

Conservative treatment with NSAIDs for canine degenerative lumbosacral stenosis (DLSS) may not provide complete efficacy for all patients. This study aimed to evaluate the potential pain‐relieving effect of amantadine through the analysis of ground reaction forces (GRFs), including Peak of Vertical Force (PVF), Vertical Impulse (VI), and stance time (ST). The study included patients which underwent neurological examination, CT scans, and force gait plate analysis. Dogs were randomly assigned to two groups: Group A received a combination of meloxicam for 7 days (0.1 mg/kg PO q24h after a loading dose of 0.2 mg/kg OS) and amantadine for 21 days (3 mg/kg PO q24h), while Group B received amantadine only, for 21 days (3 mg/kg PO q12h). Clinical re‐examinations and force gait plate analysis were performed at days 7 (T1) and 21 (T2). The final sample included 10 dogs (five in each group). No significant differences were found in age, body weight, and GRFs at baseline (T0) between the two groups. Comparing T0 and T2 within each group revealed a significant increase in PVF%BW (*p* < 0.005 for both groups) and a significant increase in VI%BW (*p* < 0.002) in group A. Nevertheless, there were no statistically significant differences in GRFs at T2 between the two groups. These preliminary findings suggest that administering amantadine effectively increased the GRFs. In the examined sample, we detected an effectively pain‐relieve effect in dogs with DLSS. Further research is warranted to validate these results and explore the long‐term effects of amantadine as a treatment option.

## 124 (P 97)

### 124.1 Brain Acute Ischemic Lacunar Infarcts Secondary to Suspected Primary Systemic Vasculitis in a Dog

#### 124.1.1 G. Brunati, DVM, GPcert (Neuro),^1^ B. Lombardo, DVM, Msc,^2^ E. Bersan, DVM, Dip ECVN, FHEA, RCVS & MRCVS^1^

##### 124.1.1.1 ^1^ Neurology Neurosurgery Unit Clinica Veterinaria Malpensa‐Anicura, Samarate, Italy; ^2^ Internal Medicine Unit Clinica Veterinaria Malpensa‐Anicura, Samarate, Italy

Primary systemic vasculitis relates to a heterogeneous group of autoimmune diseases widely described in human medicine. However, reports in the veterinary literature are scarce. A 7‐year‐old spayed female, White Swiss Shepherd Dog, presented with a 2‐day history of moderate obtundation, head pressing, ambulatory tetraparesis and inability to eat. A general physical exam revealed diffuse blackish erosive, necrotic lesions on the tongue. Neurolocalization indicated multifocal intracranial lesions involving the forebrain and brainstem. MRI of the brain demonstrated bilateral lesions at the level of the caudate nuclei and right parieto‐occipital lobe, compatible with acute ischemic lacunar infarcts (striate arteries and right middle cerebral artery). Abdominal ultrasonography revealed thrombosis of the splenic vein. Hematology and serum biochemistry revealed an increase in CK, AST, ALT, ALP, and CRP; neutrophilic leucocytosis, lymphopenia, monocytosis, and thrombocytopenia (probably due to destruction or consumption); screening for endocrine and infectious diseases was unremarkable; urine analysis was normal. There was no apparent predisposing disease for multi‐organ ischemic infarcts; therefore, a primary systemic vasculitis was suspected. The dog was started on immunomodulatory therapy (prednisolone and leflunomide) and supportive care. In the upcoming weeks, the dog showed constant improvement in the neurological exam and could eat unassisted, despite losing the left side distal tip of the tongue. A control MRI, performed 2 months later, showed the resolution of the acute brain lesions. To the authors' knowledge, this is the first report and successful treatment of suspected primary systemic vasculitis leading to multifocal ischemic infarcts in the brain in the veterinary literature.

## 125 (P 98)

### 125.1 Comparison of Cerebrospinal Fluid Neurofilament Light Chain Between Dogs With Cognitive Dysfunction Syndrome and Dogs With a Cerebral Mass Lesion

#### 125.1.1 C. Wu, BVM, MVM,^1^ Y. Chang, BVM, MVM, DipECVN^1^ ^,2^

##### 125.1.1.1 ^1^ National Taiwan University Veterinary Hospital, National Taiwan University, Taipei, Taiwan; ^2^ School of Veterinary Medicine, National Taiwan University, Taipei, Taiwan

Cognitive dysfunction syndrome (CDS) and brain tumor are two important differential diagnoses when a geriatric dog presents with progressive cerebral dysfunction. Owners often are reluctant for intracranial investigations concerning anesthetic risks in geriatric animals. Neurofilament light chain (NF‐L) has been reported to correlate with cerebral degenerative and inflammatory diseases in dogs. However, its application in other canine cerebral pathologies has not been investigated. We hypothesize that NFL is a sensitive biomarker in dogs with cerebral structural damage, and it can assist in differentiating CDS from brain tumors. Cerebrospinal fluid (CSF) was retrospectively analyzed from the following patients: dogs diagnosed with CDS based on clinical signs and intracranial investigation, dogs with MRI findings indicating a mass lesion, and dogs presenting self‐limiting neurologic manifestation with no MRI structural abnormalities (control dogs). Intracranial lesions were categorized into three types: mass lesions, degenerative changes, and mixed type, in which degenerative changes accompanied other structural abnormalities. NF‐L in CSF from 43 dogs was analyzed using the single molecule array digital enzyme‐linked immunosorbent assay. The NF‐L concentration was higher in dogs with intracranial lesions than in control dogs. In the pairwise comparisons, the NF‐L concentration differed between the degenerative versus control groups and mixed type versus control groups. The NF‐L concentration was similar among dogs with intracranial lesions.

In conclusion, our results showed that NF‐L concentration in CSF could be a sensitive biomarker for detecting cerebral structural lesions. However, it cannot serve as a test to differentiate between CDS and mass lesions.

## 126 (P 99)

### 126.1 Clinical Presentation and Computed Tomographic Findings in a Donkey Foal and Goat With Atlantooccipital Subluxation

#### 126.1.1 J. Waelkens, DVM,^1^ E. Raes, DVM, Diplomate ECVDI, PhD,^2^ E. de Bruijn, DVM,^3^ P. Vandemarcke, DVM,^4^ S. Bhatti, DVM, PhD,^1^ K. Stee, DVM, Diplomate ECVN,^1^ I. Cornelis, DVM, Diplomate ECVN, PhD^1^

##### 126.1.1.1 ^1^ Small Animal Department, Faculty of Veterinary Medicine, Ghent University, Merelbeke, Belgium; ^2^ Department of Veterinary Medical Imaging and Small Animal Orthopedics, Faculty of Veterinary Medicine, Ghent University, Merelbeke, Belgium; ^3^ Department of Large Animal Internal Medicine, Faculty of Veterinary Medicine, Ghent University, Merelbeke, Belgium; ^4^ Department of Surgery and Anesthesia of Domestic Animals, Faculty of Veterinary Medicine, Ghent University, Merelbeke, Belgium

Atlantooccipital subluxation (AOS) is a rare disease that has been previously described in horses secondary to congenital occipitoatlantoaxial malformations and in a foal with concurrent atlantoaxial luxation. Additionally, AOS has been diagnosed in a 13‐year‐old Thoroughbred gelding after trauma without congenital occipitoatlantoaxial malformations on post‐mortem examination. An 8‐days old donkey foal was presented with non‐ambulatory tetraparesis, ataxia, and cervical hyperesthesia since birth. Additionally, an 11‐month‐old goat was presented with ataxia and cervical hyperesthesia since 2 weeks. After neurological examination of both animals, revealing a C1–C5 myelopathy, a computed tomography (CT) scan of the head and cervical vertebral column was performed. CT revealed a hypoplasia of the occipital condyles and articular fovea of the atlas in the donkey foal, resulting in severe AOS with presence of part of the right wing of the atlas in the foramen magnum, whereas the goat's CT revealed an AOS, but no congenital abnormalities could be detected at the bony structures of the atlantooccipital junction. However, no trauma was witnessed in the goat, this could not be fully excluded. Both animals were initially treated with a cervical splint to immobilize the atlantooccipital junction, pending the owner's decision for surgical stabilization. Both the foal and the goat were eventually euthanized within 7 days after diagnosis because of lack of clinical improvement and decline of surgical stabilization by the owner. No post‐mortem examination was performed.

## 127 (P 100)

### 127.1 Clinical and MRI Findings of Suspected Cerebrospinal Nematodiasis (CN) in an Alpaca

#### 127.1.1 A. Gasser, Med. Vet.,^1^ M. Meylan, Prof. Dr. Med. Vet.,^2^ A. Durand, Dr. Med. Vet. DECVDI And DACVR,^3^ S. Veronika, Prof. Dr. Med. Vet., PhD, Dipl. ECVN,^1^ M. Arianna, Med. Vet., PhD, Dipl. ECVN^1^

##### 127.1.1.1 ^1^ Division of Clinical Neurology, Department of Clinical Veterinary Medicine, Vetsuisse Faculty, University of Bern, Bern, Switzerland; ^2^ Clinic for Ruminants, Department of Clinical Veterinary Medicine, Vetsuisse Faculty, University of Bern, Bern, Switzerland; ^3^Division of Clinical Radiology, Department of Clinical Veterinary Medicine, Vetsuisse Faculty, University of Bern, Bern, Switzerland

Introduction: Cerebrospinal nematodiasis (CN) refers to an aberrant migration of nematode larvae to the central nervous system. Clinical signs include paresis/paralysis, ataxia, and cranial nerve deficits. To the authors' knowledge, this is the first case report of an alpaca with suspected CN undergoing cervical and thoracolumbar magnetic resonance imaging (MRI).

Case Description: A 5‐year‐old male non‐castrated alpaca was presented with progressing gait abnormalities without signs of pain nor history of trauma. Neurological examination showed a severe pelvic limb ataxia and paresis with a wide‐based stance, spontaneous knuckling of the pelvic limbs, and delayed to absent proprioception.

Diagnostic Findings: Pre‐contrast MRI of the thoracolumbar and cervical regions did not evidence spinal cord lesions. Focal thoracic spondylosis deformans was considered incidental. CSF analysis showed an eosinophilic (> 80%) pleocytosis suggesting a parasitic infection, presumably attributable to *Elaphostrongylus cervi*. Insufficient CSF volume prevented protein analysis.

Treatment and Outcome: Treatment for parasitic infection was initiated with fenbendazole, flunixine, and ketoprofen. The alpaca was discharged 10 days after initial presentation. The pelvic limbs ataxia and paresis had improved slightly, and the stud was able to graze on the pasture.

Conclusion: This case suggests that CN is not necessarily detected on pre‐contrast MRI scan. However, CN remains the most likely differential given the CSF eosinophilic inflammation and neurological improvement under antiparasitic treatment. For a definitive diagnosis of CN, necropsy and microscopic detection of larvae in the spinal cord are mandatory.

## 128 (P 101)

### 128.1 Same Old Virus, New Victim—Bornavirus in a Hedgehog

#### 128.1.1 E. Michelakaki, DVM (Equ),^1,2^ A. Blutke, DrMedVetHabil,^2^ F. Pfaff, DrMedVet,^3^ D. Rubbenstroth, DrMedVetHabil,^3^ F. Liesche‐Starnecker, DrMedHabil,^4^ E. Dell'Era, DVM (Equ),^1^ K. Matiasek, DrMedVetHabil^1^

##### 128.1.1.1 ^1^ Section of Clinical and Comparative Neuropathology, Centre for Clinical Veterinary Medicine, LMU, Munich, Germany; ^2^ Institute of Veterinary Pathology, LMU, Munich, Germany; ^3^ Institute of Diagnostic Virology, Friedrich‐Loeffler‐Institut, Greifswald‐Insel Riems, Germany; ^4^ Institute of Pathology, University of Augsburg, Augsburg, Germany

Borna disease virus‐1 (BoDV‐1) infection causes one of the most common fatal meningoencephalitides in people, horses and alpacas in endemic areas. Until now, only shrews have been identified to host the virus as a reservoir. Herein, we describe the first encounter of BoDV‐1 in another insectivorous species affected by severe encephalitis. A captured European hedgehog ( *Erinaceus europaeus* ) was presented to a veterinary clinic due to behavioral, gait abnormalities and incoordination. As the clinical course appeared advanced, euthanasia was elected, and the carcass was submitted for postmortem examination. The animal underwent a full necropsy including histopathology of the nervous system. Thereby, a widespread lymphoplasmohistiocytic meningoencephalomyelitis was identified predominantly affecting the gray matter of cortices, diencephalon and brainstem. Moreover, the peripheral nervous system revealed a marked ganglioradiculoneuritis of similar leukocytic composition. PCR, RNAscope in situ hybridization and immunohistochemistry identified BoDV‐1 RNA and antigen within the most affected areas.

Neurological signs in this individual occurred as a consequence of BoDV‐1 infection of the central and peripheral nervous system. The lesion pattern and distribution of viral antigen fairly differs from those described in other mammalian end hosts. Despite belonging to the same order (Eulipotyphla) as the bicolored white‐toothed shrew (*Crocidura leukodon*), the extent of the lesions contradicts the possibility of this affected individual, to have served as a reservoir host. This does not exclude the possibility of hedgehogs serving as sporadic spreaders of the virus. The distribution of the virus within the general hedgehog population is yet unknown.

## 129 (P 102)

### 129.1 Magnetic Resonance Imaging Pre and Post Treatment of Sphenoid Bone Osteomyelitis in a 2‐Year‐Old Dog

#### 129.1.1 E. Mahon, BVM, BVS, MRCVS,^1^ A. Staudacher, DVM, MRCVS, DipECVDI,^2^ F. Stabile, DVM, MRCVS, PhD, DipECVN^1^

##### 129.1.1.1 ^1^ Department of Neurology and Neurosurgery, Southfields Veterinary Specialists, Linnaeus Ltd., Basildon, UK; ^2^ Department of Imaging, Southfields Veterinary Specialists, Linnaeus Ltd., Basildon, UK

Sphenoid bone osteomyelitis has been rarely described in veterinary literature. A 2‐year 5‐month male‐neutered cockerpoo was started on 0.7 mg/kg/12 h prednisolone by the referring vet following acute bilateral blindness which resolved in the left eye following treatment. Three‐months later, the dog developed third eyelid protrusion, exophthalmos of the right eye and discomfort. Neurological examination showed right‐sided absent menace response, absent direct pupillary light reflex (PLR) and hyperaesthesia of the face. MRI of the head revealed aggressive, osteolytic, partly cavitary bone lesions of the presphenoid and basisphenoid bones with marked stenosis of the right optic canal, compression of the frontal and olfactory lobes, and marked meningeal contrast enhancement of the right cerebral and cerebellar hemispheres and right optic nerve. The right‐sided retrobulbar and masticatory muscles were enlarged, T2W‐hyperintense and contrast enhancing. A presumptive diagnosis of sphenoid bone osteomyelitis, meningitis, right‐sided optic neuritis, retrobulbar, and masticatory muscle myositis was made. The dog was discharged on clindamycin 13 mg/kg/8 h, amoxicillin‐clavulanate 25 mg/kg/8 h, gabapentin 10 mg/kg/8 h, paracetamol 15 mg/kg/8 h, and prednisolone 0.5 mg/kg/24 h. Paracetamol, gabapentin, and prednisolone were gradually stopped. A 3‐month follow‐up revealed right‐eye blindness with absent menace response and PLR. MRI showed improvement in osteomyelitis, resolution of meningitis, and right optic nerve atrophy. Clindamycin was stopped. At 6‐month follow‐up neurological examination was unchanged and MRI showed only right optic nerve atrophy. Amoxicillin‐clavulanate was stopped and at 12‐month follow‐up there had been no relapses. Serial MRI to aid with tapering of antibiotic treatment has not been previously described for the treatment of sphenoid bone osteomyelitis in the dog.

## 130 (P 103)

### 130.1 Polymicrogyria and Craniosynostosis in a Golden Retriever Dog

#### 130.1.1 N. West, BVMS MRVS,^1^ C. Black, Mag. Med. Vet MRCVS,^2^ M. Ipavec, DVM MRCVS,^2^ J. Tabanez, DVM MRCVS,^2^ D. Whittaker, BSc (Hons), BVetMet (Hons), MVetMed, PhD, DipECVN, MRCVS^2^

##### 130.1.1.1 ^1^ North Downs Specialist Referrals, Part of Linnaeus Veterinary Limited, Bletchingley, Surrey, UK; ^2^ Fitzpatrick Referrals Ltd., Halfway ln, Godalming, UK

Introduction: Polymicrogyria is a cortical malformation characterized by excessive, irregular cortical gyration with shallow cerebral sulci. Polymicrogyria has been reported to occur in isolation or with other neurodevelopmental anomalies in dogs. To the authors' knowledge, co‐occurrence with other congenital anomalies, other than hypertrichosis, has not been previously reported in dogs.

Methods: We describe a case of suspected polymicrogyria and craniosynostosis in a 1‐year‐old male Golden Retriever dog. The dog presented with a 7‐month history of abnormal behavior, difficulty in training and an abnormal gait. Central blindness was noted on neurological examination and the dog localized to the forebrain. An MRI of the brain revealed an increased number of gyri and excessive cerebral convolution caudodorsally, in the region of the occipital lobes and adjacent to the falx cerebri. The skull was an abnormal shape with asymmetry of the cranial and caudal fossae resulting in corresponding deformation and asymmetry of the brain, consistent with a diagnosis of craniosynostosis. These findings were compatible with an imaging diagnosis of polymicrogyria with craniosynostosis.

Discussion: In humans, polymicrogyria has been reported to occur with craniosynostosis secondary to an underlying genetic cause, therefore further genetic studies may be warranted in dogs.

Conclusions: Polymicrogyria represents a possible differential in a young dog presenting with a gait abnormality, behavioral changes and blindness, and may occur alongside other developmental disorders in dogs.

## 131 (P 104)

### 131.1 Intracranial Secretory Meningioma in a Dog: Clinical, Imaging, Histological, and Immunohistochemical Studies

#### 131.1.1 A. Campos, PhD,^1^ M. Pumarola, PhD^2^

##### 131.1.1.1 ^1^ Hospital Veterinari les Alfàbegues, Bétera (Valencia), Spain; ^2^ Unit of Murine and Comparative Pathology, Department of Animal Medicine and Surgery, Barcelona, Spain

Meningiomas are the most common intracranial primary neoplasm in humans and dogs. However, secretory meningioma is a rare histologic variant representing 3% of human meningiomas. In animals, secretory meningiomas have been rarely described in dogs. A 10‐year‐old 19.8 kg male neutered mixed breed dog was presented in the Neurology Service with 2‐month history of chronic and progressive ambulatory tetraparesis. The physical examination was clinically unremarkable. The neurological examination was consistent with a lesion localized at C1–C5 spinal cord segments. A CT scan of the cervical spine revealed a focal, extra‐axial, well‐defined, hypodense, and homogeneously contrast enhancing lesion at the level of the caudal cerebellum, attached to the dura mater and causing marked caudal cerebellum and spinal dorsal cord compression. The mass was removed via suboccipital craniotomy. The patient significantly improved after surgery and was discharged 2 days later Histopathological examination revealed neoplastic polygonal cells organized in a cordonal pattern were observed. They showed an eosinophilic, granular, cytoplasm, and discreet hyaline inclusions. Immunohistochemical study confirm the leptomeningeal origin (EMA, Laminin) and its neurosecretory activity (Chromogranin). Taking together all these features our diagnose was intracranial secretory meningioma. To the authors' knowledge, the presenting abstract reports an intracranial secretory meningioma in a dog for the first time in the veterinary literature.

## 132 (P 105)

### 132.1 Aphonia in Dogs With Acute Onset of Severe Cervical Myelopathy Caused by Intervertebral Disc Extrusion

#### 132.1.1 E. Fiorentino, DMV, Dip. ECVN,^1^ C. Falzone, DMV, Dip. ECVN^2^

##### 132.1.1.1 ^1^ Clinica Veterinaria Privata San Marco Srl, Veggiano, Italia; ^2^ Diagnostica Piccoli Animali Srl, Zugliano, Italia

Objective: To report aphonia as a clinical sign in dogs with severe cervical myelopathy after intervertebral disc extrusion.

Methods: Medical records of dogs with severe cervical myelopathy due to intervertebral disc extrusion confirmed by MRI were retrospectively reviewed, with the main aim to look for aphonia.

Results: Four dogs with aphonia and severe cervical myelopathy due to intervertebral disc extrusion were documented. Three patients were small breed dogs and 1 medium size cross‐breed dog; 3 were males while a mean age of 8.7 years. All dogs presented with aphonia and tetraplegia with intact nociception. Exactly 3/4 had generalized decreased spinal reflexes, possibly due to concurrent spinal shock. A total of 2/4 dogs showed also dyspnea while only 1/4 was painful. MRI revealed C3–C4 intervertebral disc extrusion in all cases and 2/4 were HNPE. According to the severity of spinal cord compression 2/4 dogs underwent surgical decompression, 1/4 was treated conservatively, 1/4 was euthanized immediately after the diagnosis. All patients recovered their own tone of voice in a mean time of 13 days, whereas the improvement of the deambulation took longer; however, all dogs had full recovery at 2 months follow‐up.

Conclusion: although rare, this case series reported aphonia as a possible clinical sign in dogs affected by severe cervical myelopathy due to an acute intervertebral disc extrusion. Unlike respiratory compromise, aphonia does not seemed to be related to a poor prognosis; however, further studies are needed to fully understand its relevance and etiopathogenesis.

## 133 (P 106)

### 133.1 Prevalence, Clinical Presentation, and Outcome in Dogs With Suspected Spinal Shock Secondary to Acute Cervical Disc Herniation

#### 133.1.1 E. Fiorentino, DMV, Dip. ECVN,^1^ C. Falzone, DMV, Dip. ECVN^2^

##### 133.1.1.1 ^1^ Clinica Veterinaria Privata San Marco Srl, Veggiano, Italia; ^2^ Diagnostica Piccoli Animali Srl, Zugliano, Italia

Objective: to characterize prevalence, clinical presentation and outcome in dogs with acute cervical disc herniation and suspected spinal shock.

Methods: medical records of dogs presented with an acute onset of cervical myelopathy between 2015 and 2023 were reviewed. All patients should have had an acute onset of tetraparesis/tetraplegia with decreased/absent spinal reflexes on all four limbs and an MRI diagnosis of acute cervical disc herniation.

Results: Exactly 430 patients with MRI consistent with acute cervical disc herniation were reviewed. Five dogs (1.16%) met the inclusion criteria; 5/5 patients were small breed dogs and 3 were males, with mean age being 10.2 years; 3/5 patients showed tetraplegia with intact nociception, 2/5 non‐ambulatory tetraparesis; 3/3 tetraplegic dogs had also aphonia while 1/3 was dyspnoic. All patients had generalized decreased/absent spinal reflexes. MRI confirmed the diagnosis of disc extrusion in all 5 dogs, 3 of which had a HNPE. Two dogs underwent surgical decompression, 2 were treated conservatively and 1 was euthanized after the diagnosis. The follow‐up at 2 months demonstrated a complete recovery in all dogs, regardless the therapy.

Conclusion: Despite the prevalence of spinal shock is low in acute cervical disc herniation, it should be considered in dogs presenting with an acute onset of severe tetraparesis or tetraplegia, that can mimic an acute neuromuscular syndrome. Although the small sample size, spinal shock due to acute cervical disc herniation does not seem to represent a poor prognostic sign.

## 134 (P 107)

### 134.1 Elevated Interleukin‐31 Levels in Serum of Dogs With Steroid‐Responsive Meningitis‐Arteritis Suggest an Involvement in its Pathogenesis

#### 134.1.1 L. Lemke, Dr,^1^ R. Carlson,^1^ K. Warzecha, Dr,^1^ A. V. Volk, Dr., DipECVD,^1^ A. Tipold, Prof, Dr., DipECVN,^1^ J. Nessler, Dr., DipECVN^1^

##### 134.1.1.1 ^1^ Department of Small Animal Medicine and Surgery, University of Veterinary Medicine Hannover, Hanover, Germany

The exact pathomechanism of Steroid‐Responsive Meningitis‐Arteritis (SRMA) has not been finally clarified, but a Th‐2 immune mediated reaction is most likely. The cytokine Interleukin‐31 (IL‐31) affects different immune and non‐immune cells and is predominantly produced by T cells with a Th‐2 phenotype. It is part of the IL‐6 cytokine family and expressed during proinflammatory conditions. Therefore, we hypothesize that IL‐31 might be a further component leading to an aberrant immune reaction in SRMA. IL‐31 was measured in archived samples (31 serum and 31 CSF samples) of 40 dogs with SRMA, with atopic dermatitis and of healthy control dogs using a competitive canine IL‐31 ELISA. Mean serum IL‐31 level in dogs with SRMA was 260.7 pg/mL (*n* = 18, 10.78–1050 pg/mL) and therefore mildly higher compared with the mean IL‐31 level of dogs with atopic dermatitis of 228.3 pg/mL (*n* = 3, 62.97–437.9 pg/mL) and markedly higher than in healthy control dogs (80.7 pg/mL; *n* = 10, 42.67–137.2 pg/mL). Especially dogs with SRMA without any pre‐treatment showed markedly higher IL‐31 levels than patients pre‐treated with non‐steroidal anti‐inflammatory drugs or steroids. Highest values were measured in dogs in the peracute stage of the disease. Mean CSF IL‐31 value was 183.5 pg/mL (*n* = 23) in dogs with SRMA and did not differ significantly from the mean IL‐31 levels of the healthy control dogs (*n* = 8). Based on this study, an involvement of IL‐31 in the pathogenesis of the Th‐2 immune mediated immune response in SRMA can be assumed, especially the systemic changes of the disease and the proinflammatory action of the cytokine can be well explained.

## 135 (P 108)

### 135.1 Surgical Treatment of Occipital Dysplasia and Occipitoatlantoaxial Malformations Using a Custom‐Made Three‐Dimensional Occipital Titanium Mesh in a Dog

#### 135.1.1 N. West, BVMS MRCVS,^1^ B. Jones, BScVetSci MSc BVetMed PGDip DipECVN MRCVS,^2^ B. Oxley, MA VetMB DSAS (Orth) MRCVS,^3^ R. Cappello, DVM PhD DipECVN MRCVS RCVS^1^

##### 135.1.1.1 ^1^ North Downs Specialist Referrals, Part of Linnaeus Veterinary Limited, Bletchingley, UK, Bletchingley, Surrey, UK; ^2^ Department of Neurology and Neurosurgery, Langford Vets Small Animal Hospital, Bristol, UK; ^3^ Vet3D, Coventry, West Midlands, UK

Introduction: Occipitoatlantoaxial malformation (OAAM) is a developmental defect resulting in craniovertebral junction malalignment. In this dog a severe occipital dysplasia was present with compression of the cerebellum.

Methods: An 11‐month‐old male neutered Pomeranian dog presented with an acute history of cervical hyperesthesia and progressive hypermetric tetraparesis. An MRI and CT scan confirmed the presence of OAAM; namely occipital dysplasia, hypoplasia of the dorsal arch of C1, atlantoaxial instability together with syringomyelia.

Results: A dorsal approach was made over the skull and the first two cervical vertebrae. A custom‐made three‐dimensional titanium mesh implant was implanted over the occipital bone defect. The implant was formed to augment the caudal fossa allowing the cerebellum to expand to sit in its natural position. The mesh was secured with bilateral bi‐cortical screws inserted into the pedicle of C1. Bilateral bi‐cortical screws were also placed in the pedicle and transverse process of C2. Polymethylmethacrylate was used to enshroud the bi‐cortical screws together with the caudo‐ventral aspect of the titanium mesh, as well as to implant the titanium mash to the dorsal occipital bone. At the 4‐week follow up, the dog was comfortable upon cervical palpation and was ambulatory with no deficits. At 12‐month follow up the dog was neurologically normal, however had infrequent, intermittent episodes of phantom scratching.

Discussion/Conclusion: This report describes a unique dorsal surgical technique for management of OAAM. This method is a viable alternative for the correction of caudal fossa defects.

## 136 (P 109)

### 136.1 Suspected Temporal Lobe Epilepsy in a Dog With Prevailing Gastrointestinal Signs

#### 136.1.1 S. Diop, DVM, ECVN Resident,^1,2^ N. Van Caenegem, DVM, ECVN Board Eligible candidate,^3^ V. Freiche, DVM, PhD, DESV‐MI,^4^ E. Lyon, DVM, PhD student,^5^ C. Escriou, DVM, PhD,^5^ S. Blot, DVM, PhD, ECVN diplomate^1,2^

##### 136.1.1.1 ^1^ Unité de Neurologie, CHUV‐AC, École Nationale vétérinaire d'Alfort, Maisons Alfort, France; ^2^ Univ Paris Est Créteil, INSERM, U955 IMRB “Biology of the Neuromuscular System” Team, Maisons Alfort, France; ^3^ Clinique Vétérinaire AniCura TRIOVet, Rennes, France; ^4^ Unité de Médecine Interne, CHUV‐AC, École Nationale vétérinaire d'Alfort, Maisons Alfort, France; ^5^ Univ Lyon, VetAgro Sup, Marcy‐l'Etoile, France

In human temporal lobe epilepsy, focal seizures may clinically express as various autonomic signs, such as gastrointestinal symptoms (retching, nausea, vomiting, abdominal pain, rumbling, and diarrhea), cardiovascular changes, variation of pupillary size (mydriasis and myosis), and others. Focal cognitive seizures are also reported with, for example, altered awareness, and déjà vu sensation. In dogs, temporal lobe epilepsy was suspected in phenobarbital responsive dysphagia syndrome, but other manifestations are scarcely described. We report a 5‐years‐old intact male Lhassa Apso with paroxysmal gastro‐intestinal episodes (rumbling, nausea, vomiting without food content, and epigastric pain), lasting several hours, occurring two to three times per month, since his adoption 3 years ago. The dog also showed cluster episodes of impaired awareness, chewing, and licking his paws since adoption, especially during rest. All possible causes of reported gastro‐intestinal signs were excluded by extensive work‐up and the recurrent digestive episodes occurred despite conventional treatment of chronic gastroenteritis. Presumptive diagnosis of idiopathic epilepsy with Tier I confidence level was reached based upon young age at onset, unremarkable inter‐ictal general and neurological examination for 3 years, and no clinically significant abnormalities on minimum data base blood tests. Electroencephalography showed multiple spikes and waves of moderate amplitude during rest. The disease responded partially to levetiracetam monotherapy while adjunction of phenobarbital led to reduction of 80% in all type of episodes frequency. This case report supports the occurrence of temporal lobe epilepsy‐like syndrome in dogs.

## 137 (P 110)

### 137.1 Fractionated Megavoltage Radiation Therapy of a Canine Cervical Spinal Cord High‐Grade Astrocytoma With Secondary Seeding: One Year Follow‐Up

#### 137.1.1 G. Scherf, DVM, Neurology Resident in Training,^1^ P. De Fornel, DVM, DESV‐MI,^1^ C. Benzimra, DVM, Dip. ECVDI,^1^ C. Lassaigne, DVM, Dip. ECVDI,^1^ R. François, BSc,^1^ J. Deniaud, BSc,^2^ M. Colle, DVM, PhD, Pr., Dip. ECVP,^2^ ^,3^ J. Thibaud, DVM, Dip. ECVN^1^

##### 137.1.1.1 ^1^ MICEN Vet, Créteil, France; ^2^ UMR 703 INRAE/Oniris, Animal Pathophysiology and Biotherapy for Muscle and Nervous System Diseases, Nantes, France; ^3^ Department of Veterinary Anatomic Pathology, Nantes Atlantic College of Veterinary Medicine, Food Science and Engineering (Oniris), Nantes, France

Spinal cord astrocytomas represent 24%–32% and 13% of human and canine intramedullary tumors respectively. In humans, the preferred therapeutic approach consists of surgery followed by radiation therapy (RT) ± chemotherapy. When tumor seeding is present craniospinal irradiation is recommended. According to the US SEER data, the 5‐year overall survival rate of human spinal cord astrocytomas is 58%. In veterinary medicine no specific guideline exists. In particular, there is no reported outcome following sole RT. We describe the case of a canine high‐grade cervical intramedullary astrocytoma treated by fractionated megavoltage RT (6‐MV linear accelerator), with a one‐year clinical and imaging follow‐up. A 3‐year‐old ambulatory hemiparetic English Bulldog was diagnosed with a C7–T1 intramedullary mass lesion. A glial tumor was suspected and treated by focal 3D‐conformal fractionated megavoltage RT (15 × 3 Gy sessions over 5 weeks). The patient improved clinically and on control MRI upon completion of RT. Symptoms relapsed 4.5 months later. Complete CNS MRI revealed lesions caudal to the cervical lesion suggestive of tumor seeding. Craniospinal irradiation was performed, excluding the previously irradiated lesion (intensity‐modulated RT, 12 × 3 Gy over 4 weeks, plus 3 × 3 Gy over the metastatic lesions). The neurological deficits disappeared. The patient relapsed clinically and on imaging 5.5 months later and was euthanized. Survival time from diagnosis was 423 days. A high‐grade spinal cord astrocytoma with leptomeningeal and intramedullary metastasis was histologically confirmed post‐mortem. This is the first report of a canine spinal cord high‐grade astrocytoma with tumor seeding treated by craniospinal fractionated megavoltage RT without clinical side effect.

## 138 (P 111)

### 138.1 Genetic Analyses Reveal MECR and ATG4D Variants in Eurasier Dogs With a Presumed Polioencephalopathy

#### 138.1.1 F. Rawson, BVetMed PGDipVCP MRCVS,^1^ M. Christen, Dr. Med. Vet.,^2^ J. Rose, BA VetMB DipECVN AFHEA MRCVS,^3^ E. Paran, DVM DipECVDI MRCVS,^1^ T. Leeb, Prof. Dr. Rer. Nat.,^2^ A. Fadda, DVM, Dr. Med. Vet., DECVN, PhD, MRCVS^1^

##### 138.1.1.1 ^1^ Langford Veterinary Services, University of Bristol, Bristol, UK; ^2^ Institute of Genetics, Vetsuisse Faculty, University of Bern, Bern, Switzerland; ^3^ Lumbry Park Veterinary Specialists, Alton, UK

Introduction/Purpose: Polioencephalopathies are characterized by multifocal, bilateral, and symmetrical central nervous system lesions and are typically manifestations of selected susceptibility of cellular sub‐populations to metabolic aberrations. We aimed to characterize a presumed polioencephalopathy in a family of Eurasier dogs.

Methods: Three Eurasier dogs from the same litter presented with movement disorders with preserved consciousness. Progressive gait abnormalities were later detected in 2 of the dogs, and a persistent divergent strabismus in 1. Video footage was assessed in all dogs, and Dogs 1 and 2 had clinical investigations. Whole genome sequencing of Dog 1 together with further genetic analyses in the family were performed. A cohort of 115 Eurasier controls was genotyped for specific variants.

Results: The dogs presented with periods of generalized ataxia and a hypermetric thoracic limb gait, dystonia, choreoathetosis and thoracic limb ballism. Two dogs both had an abnormal gait, which appeared progressive, particularly in dog 1. MRI of the brain in Dog 1 and 2 documented symmetrical, bilateral T2/FLAIR hyperintense, T1 hypo to isointense, non‐enhancing lesions at the level of the dorsolateral aspect of the caudate nucleus, lateral and medial geniculate nuclei, thalamus, hippocampi, and rostral colliculus and mild generalized brain atrophy. Genetic analyses of littermates and parents revealed a homozygous MECR missense variant in all 3 affected dogs, and a homozygous ATG4D missense variant in Dog 1 and 2.

Discussion/Conclusion: We present a presumed hereditary and progressive polioencephalopathy in a litter of Eurasier dogs; research is needed to establish a pathogenic effect of the detected genetic variants.

## 139 (P 112)

### 139.1 Surgical Management of a Subdural Empyema Along the Brainstem and the Cerebellum in a 4‐Year‐Old Cat

#### 139.1.1 M. Moinard, DVM, Res. ECVS,^1^ N. Van Caenegem, DVM, Res. ECVN,^1^ B. Riedinger, DVM, Dipl. ECVS^1^

##### 139.1.1.1 ^1^ Veterinary Clinic AniCura TRIOVet, Rennes, France

Introduction: Subdural empyema (SE) is a rare condition in cats considered as an emergency. We describe the surgical management of SE by drainage through a single hole in the basioccipital bone.

Methods/Case Description: A 4‐year‐old neutered male Domestic Short‐haired cat was treated for a chronic right bacterial otitis externa, media and interna associated with a focal meningitis by total ear canal ablation and lateral bulla osteotomy after failure of appropriate medical therapy. Three weeks after surgery, the cat was presented with a worsening general condition, persistent hyperthermia, right vestibular syndrome, and right facial nerve dysfunction. Previous surgical site was unremarkable. Magnetic Resonance Imaging (MRI) examination revealed a right otitis media, otitis interna, meningitis, and extensive SE associated with a dorsal mass effect on the adjacent brainstem and cerebellum. Surgical management by ventral bulla osteotomy (VBO) and drainage of the SE was decided. Lavage and drainage of the SE was performed through a burr hole in the basioccipital bone.

Results: One month post‐operatively, the cat showed a good general condition with partial regression of neurological signs. Control MRI showed the resolution of the SE. The right tympanic bulla was filled with fibrotic material.

Discussion/Conclusion: To our knowledge this is the first case report of surgical management of feline SE by VBO and a single drainage hole in the basioccipital bone. This procedure is feasible with a simple approach and allows decompression, drainage, and collection of material for bacterial culture.

## 140 (P 113)

### 140.1 Anti‐Ganglioside Antibodies in Nordic Horses With Acquired Equine Polyneuropathy

#### 140.1.1 F. S. Skedsmo, PhD,^1^ K. H. Jäderlund, Professor,^2^ G. Gunnes, Professor,^1^ S. Hanche‐Olsen, PhD,^2^ E. F. Kvigstad, PhD Student,^1^ K. Matiasek, Professor,^4^ H. Reineck‐Bosaeus, Medical Laboratory Scientist,^3^ G. Gröndahl, Acting State Veterinarian, Equine Diseases^3^

##### 140.1.1.1 ^1^ Department of Preclinical Sciences and Pathology, Norwegian University of Life Sciences, Ås, Norway; ^2^ Department of Companion Animal Clinical Sciences, Norwegian University of Life Sciences, Ås, Norway; ^3^ Department of Animal Health and Antimicrobial Strategies, National Veterinary Institute, Uppsala, Sweden; ^4^ Clinical and Comparative Neuropathology, Centre for Clinical Veterinary Medicine, Ludwig Maximilians University, Munich, Germany

Acquired equine polyneuropathy (AEP) in Nordic horses, a schwannopathy with inflammatory demyelination and predominantly sensory dysfunction, appears as farm outbreaks. Affected nerves have harbored immunoglobulin deposits inside the myelin spirals. Gangliosides are sialic acid‐containing glycosphingolipids. The aim was to study serum anti‐gangliosides in horses during outbreaks, and to detect gangliosides in healthy equine nerves. With a modified ELISA, IgM and IgG antibodies to six gangliosides were analyzed in sera (*n* = 54–118) from healthy control horses in different farms without AEP. Sera (*n* = 49–167) from, at sampling, symptomatic and asymptomatic horses in 30 different farms with active outbreaks of AEP were also analyzed. Cut‐off for positive reaction was mean OD + 2SD of controls and a two‐sample independent *t*‐test was used for statistical analysis. Immunofluorescence and immune‐EM for GM1 and GM2 was performed on nerves from non‐neurological horses. For all tested gangliosides, ODs of IgM antibodies were higher in both symptomatic and asymptomatic horses (to the same extent) compared with control horses (*p* < 0.05). The proportion of IgM positive horses in AEP farms for the different gangliosides was: GM1—61%, GM2—55%, GD1a—53%, GD1b—82%, GQ1b—56%, and MAG—38%. For IgG anti‐ganglioside antibodies, 98%–100% of these sera were below cut‐off. By in situ testing, the equine nerves demonstrated GM1 and GM2 in myelin sheaths. IgM anti‐gangliosides appear to be biomarkers for AEP on farm level. Notably, human anti‐GD1b IgM and anti‐GQ1b IgM have been associated with sensory polyneuropathies. In addition, human anti‐GM1 IgM have been associated with multifocal motor neuropathy.

## 141 (P 114)

### 141.1 Holocord Syringomyelia: Clinical and MRI Findings in 18 Dogs

#### 141.1.1 D. Douralidou, DVM, MRCVS,^1^ L. Mari, DVM, DipECVN, MRCVS,^1^ J. J. Minguez, DVM, MRCVS,^2^ P. Alvarez Fernandez, DVM, DipECVN, MRCVS,^2^ C. Anselmi, DVM, DipECVDI, MRCVS,^2^ A. Wessmann, DrMedVet, PGCertAcPrac, FHEA, DipECVN, MRCVS,^2^ S. Wyatt, BVetMed (Hons), MVetMed, DipECVN, MRCVS,^3^ C. Posporis, DVM (Hons), PGCertVSM, DipECVN, MRCVS^1^ ^,2^

##### 141.1.1.1 ^1^ The Ralph Veterinary Referral Centre, Marlow, UK; ^2^ Pride Veterinary Referrals, IVC Evidensia Group, Derby, UK; ^3^ Queen Mother Hospital for Animals, Royal Veterinary College, Hatfield, UK

Holocord syringomyelia (HSM) represents a continuous spinal cord cavitation, usually extending from C2 to L4 vertebrae. The purpose of our study is to describe the signalment, neurological signs, MRI findings, and clinical evolution of dogs with HSM. Medical records of dogs diagnosed with HSM on MRI were reviewed retrospectively from the database of three referral hospitals. Eighteen dogs with median age of 82 months were included. French Bulldogs were overrepresented (50%). Sixteen dogs (89%) had signs associated with HSM. Six dogs (33%) were presented for unrelated concurrent conditions. Signs of myelopathy were detected in 13 dogs (72%), 12 localizing to C1–T2 spinal cord segments. Eight dogs (44%) manifested neuropathic pain. On MRI, HSM extended from C1/C2 vertebrae in 18 dogs (100%) to L3 in four (22%), and L4 or caudally in 14 (78%). Seventeen dogs (94%) had Chiari‐like and/or other intracranial/craniocervical malformations. Ten dogs (56%) were treated medically, one (5%) surgically and seven (39%) received no treatment for HSM. Follow‐up information was available in 16 dogs with a median of 12 months. Two dogs (12.5%) improved, eight (50%) deteriorated, and six (37.5%) remained static. Long‐term treatment in 11 dogs (69%) included gabapentin (11 dogs), corticosteroids (five dogs) or non‐steroidal anti‐inflammatories (four dogs), paracetamol (two dogs), and/or amitriptyline (one dog). To the authors' knowledge, this is the first case series describing the holocord variant of syringomyelia in dogs. French Bulldogs were overrepresented, signs of myelopathy and disease progression were common. Further research into the underlying etiopathogenesis and genetic implications is required.

## 142 (P 115)

### 142.1 Canine Brain Localization of Chronic Myelomonocytic Leukemia

#### 142.1.1 I. Tartari, DVM,^1^ F. Tocco, DVM,^1^ G. Cancedda, DVM,^2^ V. Angioni, DVM,^3^ M. Podda, DVM,^4^ A. Gallucci, DVM, PhD, ECVN Diplomate, EBVS specialist^1^

##### 142.1.1.1 ^1^ Veterinary Neurological Center “La Fenice,” Selargius (CA) 09047, Italy; ^2^ Veterinary Clinic Cancedda, Carbonia (SU) 09013, Italy; ^3^ Veterinary Clinic “Baroni Rossi,” Cagliari 09125, Italy; ^4^ Veterinary Clinic “San Francesco,” Assemini (CA) 09032, Italy

An 8‐years‐old, male, mixed breed dog was presented with a 2‐day history of reluctance to move and ataxia of all limbs. Physical examination was normal while neurological examination showed proprioceptive ataxia and proprioception deficits in all four limbs. Menace response was reduced on the right eye and discomfort was detected on the neck manipulation. A multifocal localization with involvement of left forebrain and cervical spinal cord was considered. The main differentials were inflammatory or neoplastic disease.

Hematologic abnormalities included moderate non‐regenerative anemia, severe thrombocytopenia and moderate‐to‐marked leukocytosis characterized by marked monocytosis. The magnetic resonance imaging (MRI) scan revealed intra‐axial multifocal lesions localized at the level of left thalamus and bilateral parietal lobes with inhomogeneous lesions contrast acquisition and diffuse meningeal enhancement. In the spinal cord a C2–C3 intramedullary fusiform lesion with the same contrast enhancement was found. Cerebellomedullary cerebrospinal fluid (CSF) tap showed a marked mononuclear pleocytosis characterized by myeloid blast with similar cells observed in peripheral blood and in bone marrow preparations. The dog was diagnosed with chronic myelomonocytic leukemia (CML) confirmed by flow cytometry. Prednisolone 1 mg/kg twice daily and toracenib 2.7 mg/kg twice per week were administered with marked improvement of the neurological signs, still in remission during the first 1‐month follow‐up. For the authors' knowledge this is the first in vivo report of CML with central nervous system involvement detected by MRI and CSF examination.

## 143 (P 116)

### 143.1 Intraventricular Hemorrhage in a Case of Fungal Ventriculitis and Meningoencephalitis in a Dog

#### 143.1.1 J. González‐Caramazana, DVM,^1^ M. Pumarola, DVM, PhD, Dip ECVP,^2^ S. Ortiz, DVM, Board Elegible ECVN,^1^ R. Fusté, DVM,^1^ M. Al‐Abrash, DVM,^1^ E. Blasco, DVM,^2^ T. Heredia, DVM,^1^ J. J. Sánchez, DVM,^4^ S. Ródenas, DVM, Dip ECVN^3^

##### 143.1.1.1 ^1^ AniCura Valencia Sur Veterinary Hospital, Valencia, Spain; ^2^ Unit of Murine and Comparative Pathology (UPMiC), Department of Animal Medicine and Surgery, Faculty of Veterinary of the Autonomous University of Barcelona, Barcelona, Spain; ^3^ Blue Care Veterinary Hospital, Málaga, Spain 4 La Rambla 7 Palmas Veterinary Clinic, Las Palmas, Spain

This case describes a dog with pyogranulomatous fungal ventriculitis, and meningoencephalitis (PFVM) and intraventricular hemorrhage (IH) detected by intracranial magnetic resonance imaging (MRI). To the author's knowledge, IH associated with PFVM has not been previously described. A 5‐year‐old spayed female American Staffordshire Terrier was presented to the Neurology service with a one‐day history of obtundation and non‐ambulatory tetraparesis. Neurological examination revealed a widespread forebrain involvement. Intracranial MRI (1.5 T) demonstrated multiple intra‐axial lesions in the left temporal and piriform lobes (hyperintense on T2WI and FLAIR, isointense on T1WI without contrast enhancement). Additionally, at the level of the caudate nuclei, the left lateral ventricle appears iso/hypointense on T2WI and FLAIR with signs of hemorrhage on MERGE and SWAN images. Periventricular contrast enhancement was noticed around the ependyma/choroid plexus of the lateral ventricles. Analysis of cerebrospinal fluid (CSF) revealed a mixed pleocytosis (14 WBC/μL) with mononuclear cells (68%) and neutrophils (32%). Histopathological examination confirmed the presence of PFVM with filamentous structures consistent with the fungus Zygomycetes. These structures were observed within the ventricular lumen in association with a pyogranulomatous inflammatory infiltrate. MRI findings in dogs with PFVM typically show intra‐ or extra‐axial masses that may exhibit variable contrast enhancement, and usually involve nasal or retrobulbar extension with cribriform plate destruction. To our knowledge this is the first report of IH associated with PFVM.

In line with the reported findings, PFVM should be considered as a potential differential diagnosis when intraventricular hemorrhage (IH) is detected on MRI in dogs presenting with neurological signs.

## 144 (P 117)

### 144.1 Window Entrapment Trauma in Cats: Correlation Between Clinical, Neurological, and Laboratorial Findings

#### 144.1.1 F. Graciolli Tomazi, Med. Vet.,^1^ A. Maiolini, PhD, Dipl. ECVN,^1^ V. Stein, PhD, Dipl. ECVN,^1^ T. Von Klopmann, Dr. Med. Vet., Dipl. ECVN,^2^ J. Hauer, Dr. Med. Vet.,^2^ L. Peters, Dr. Med. Vet., Dipl. DACVP,^3^ F. Steffen, Dr. Med. Vet., Dipl. ECVN^4^

##### 144.1.1.1 ^1^ Division of Clinical Neurology, Department of Clinical Veterinary Medicine, Vetsuisse Faculty, University of Bern, Bern, Switzerland; ^2^ Department of Neurology, Small Animal Clinic Hofheim, Hofheim am Taunus, Germany; ^3^ Clinical Diagnostic Laboratory, Department of Clinical Veterinary Medicine, Vetsuisse Faculty, University of Bern, Bern, Switzerland; ^4^ Division of Neurology, Department for Small Animals, Vetsuisse Faculty, University of Zurich, Zurich, Switzerland

Window entrapment in cats causes reduced perfusion of the spinal cord, muscles, and nerves leading to ischemic neuromyelomyopathy. Depending on severity and duration of entrapment, clinical and neurological status as well as prognosis vary widely. Few reports about window‐entrapment‐trauma are available, mostly comparing neurological status and outcome. The aim of this retrospective (2000–2022) multicenter study was to evaluate neurological status of cats with bottom‐hung window entrapment at admission and discharge and correlate with several blood parameters. We hypothesize that Creatine kinase (CK), alanine aminotransferase (ALAT), aspartate transaminase (ASAT), creatinine and/or urea (BUN) values correlate with severity of neurological findings and prognosis for recovery of motor function and might therefore have a predictive value for the outcome. Patients with clinical and neurological examination (at admission and discharge) and blood analysis of at least one of the previously mentioned parameters were included in the study. Seventy‐two cats met the inclusion criteria, of which 54 presented paraparesis (32 ambulatory and 22 non‐ambulatory) and 18 paraplegia (8 with and 10 without deep pain sensation). CK, ASAT, and ALAT were elevated in most of the cases, while creatinine and BUN only in few cases. No correlations between neurological status and outcome were found. This is the first retrospective study correlating blood parameters with neurological outcome in window entrapment trauma. The results indicate that examined blood parameters are not helpful to predict outcome in cats with window‐entrapment‐trauma.

## 145 (P 118)

### 145.1 Afferent Pathways of the Urinary Bladder in Sheep: Recordings From the Extradural Sacral Roots

#### 145.1.1 J. Prager, BSc, BVSc, PhD, MRCVS,^1^ ^,2^ M. Leonhardt, MEng,^3^ L. Jabban, MEng,^3^ M. Petrou, MEng,^3^ N. Granger, DVM, PhD, DipECVN, FHEA, FRCVS,^1^ ^,2^ J. Taylor, BSc, PhD,^3^ N. Donaldson, MA, PhD,^4^ B. Metcalfe, MEng, PhD^3^

##### 145.1.1.1 ^1^ The Royal Veterinary College, London, UK; ^2^ Bristol Vet Specialists, Bristol, UK; ^3^ Department of Electronic and Electrical Engineering, University of Bath, Bath, UK; ^4^ Department of Medical Physics and Biomedical Engineering, University College London, London, UK

Implantable sacral anterior root stimulation (SARS) allows voluntary urinary voiding after severe supra‐sacral spinal cord injury (SCI) in people and dogs. To prevent incontinence due to reflex bladder contractions, anterior roots are cut. In rats, detection of afferent bladder activity allows electrical blocking of bladder contractions. Translation requires non‐invasive implants on as few roots as possible. Six anesthetized ewes had urethral catheterization allowing bladder filling and intravesical pressure (PVes) measurement. The extradural S1, S2, and S3 roots were exposed and electrically stimulated in turn. Book electrodes, similar to SARS devices, were implanted bilaterally. UK Home Office license and ethical approval was obtained. Electroneurogram (ENG) data during repeat bladder filling (*n* = 3–5) were amplified, recorded and processed to calculate correlation to PVes. Correlation of ENG to PVes significantly differed between sheep [*F*(5,60) = 15.5, *p* < 0.0001, 2‐way ANOVA] and root [*F*(2,60) = 11.9, *p* < 0.0001] with interaction effect [*F*(10,60) = 8.33, *p* < 0.0001]. Mean ± SD correlations: S1 = 0.41 ± 0.37; S2 = 0.64 ± 0.13; S3 = 0.5 ± 0.25; significant difference between S1 and S2 [*p* < 0.001, post hoc Tukey] and S2 and S3 [*p* = 0.016] but not S1 and S3 [*p* = 0.12]. Electrical stimulation of roots increased PVes. PVes increase significantly differed between sheep [*F*(5,17) = 36.1, *p* < 0.0001, 2‐way ANOVA] and root [*F*(2,17) = 25.8, *p* < 0.0001] with interaction effect [*F*(10,17) = 3.6, *p* = 0.01]. Mean ± SD PVes increase: S1 = 5.7 ± 7.6 cmH_2_O; S2 = 14.5 ± 8.3 cmH_2_O; S3 = 12.0 ± 9.3 cmH_2_O; significant difference between S1 and S2 (*p* < 0.0001, post hoc Tukey) and S1 and S3 (*p* = 0.0002) but not S2 and S3 (*p* = 0.15). This suggests S2 root has greatest afferent bladder activity, but efferent activity may be primarily via S2 and S3. Interaction effects suggest individual differences in bladder innervation, important for neuroprosthesis design for bladder control after SCI.

## 146 (P 119)

### 146.1 Biomechanical Comparison of Unicortical Versus Bicortical Polyaxial Screws for Stabilization of the Canine Lumbar Vertebrae

#### 146.1.1 J. Guevar, DVM, MVM, DECVN, MRCVS,^1^ B. Voumard, PhD,^2^ R. Bergman, DVM DACVIM (Neurology),^3^ F. Franck, Dr. Med. Vet. DECVS^1^

##### 146.1.1.1 ^1^ Vetsuisse Faculty Bern, Small Animal Surgery Department, Bern, Switzerland; ^2^ ARTORG Center Musculoskeletal Biomechanics, Bern, Switzerland; ^3^ Synapse Veterinary Services, Fort Mill, South Carolina, USA

Introduction/Purpose: The use of polyaxial screws is emerging in veterinary spinal surgery for the purpose of vertebral stabilization. However, there is a lack of information on biomechanics of these implants in dogs. The purpose of this study was to evaluate and compare biomechanical proprieties of a single vertebral motion unit between various 2 screws/1 rod constructs in the canine lumbar spine.

Methods: L1–L2 vertebral motion unit (VMU) of thoracolumbar (T13–L3) vertebral specimens were tested in flexion/extension and lateral bending with several 2 screws/1 rod constructs. Testing was performed in the following conformations: intact, then after unilateral monocortical stabilization (iUniMo), then bilateral monocortical stabilization (iBiMo) and finally in a destructive mode with the bilateral construct (dBiMo) followed by unilateral construct (dUniMo). The same sequence was performed with bicortical screws (iUniBi, iBiBi, dBiBi, and dUnibi). Destructive mode represented a 3‐column fracture model (intervertebral disc, all ligaments, and articular processes transected).

Results: During destructive mode testing, two samples failed in flexion/extension with unicortical screws, and two samples failed in lateral bending with unicortical screws. When compared with the intact VMU in flexion extension, the relative range of motion (rROM) to intact was always over 20% with unilateral and bilateral constructs; rROM to intact was always decreased with bilateral constructs but not always with unilateral construct in lateral bending.

Discussion/Conclusion: In destructive mode, 2 screws/1 rod montage leads to decrease ROM of the VMU in lateral bending using bilateral constructs but leads to a significant increase of ROM in flexion/extensionindependently of the construct. Any 2 screws/1 rod construct is not recommended for stabilization of a 3‐column fracture in the canine lumbar spine.

## 147 (P 120)

### 147.1 Successful Multimodal Therapy of a C1–C2 Extradural Atypical Mesenchymal Neoplasm

#### 147.1.1 S. Sánchez Briones, DVM, MRCVS,^1^ R. M. Rabanal, BSc, PhD,^2^ C. Posporis, DVM (1st Hons), DipECVN, PGCertVSM, MRCVS,^1^ P. Álvarez Fernández, DVM, MRCVS, DipECVN, EBVS^1^

##### 147.1.1.1 ^1^ Neurology and Neurosurgery Service, Pride Veterinary Referrals IVC Evidensia Group, Derby, UK; ^2^ Unitat de Patologia Murina i Comparada, Departament de Medicine i Cirurgia Animals, University Autonomous of Barcelona, Barcelona, Spain

Mass lesions in the vicinity of the spinal nerve root with associated foraminal widening raise the suspicion of a peripheral nerve sheath tumor (PNST). A definitive diagnosis can only be reached with histopathology but treatment guidelines are lacking and prognosis remains uncertain. A 6‐year‐3‐month‐old male neutered cocker spaniel dog was referred with a 2‐week history of progressive severe neck pain without neurological deficits. MRI revealed an extradural mass occupying the right ventrolateral aspect of the vertebral canal at C1–C2. Spinal cord compression, thickening of the dorsal spinal nerve root and foraminal widening were also detected. PNST, round cell neoplasia, or sarcoma were considered as possible differential diagnoses. A C1–C2 right‐sided hemilaminectomy was performed via dorsal approach and revealed an extradural red gelatinous mass located ventrolateral to the spinal cord. The mass was causing severe right C2 spinal nerve root stretching without infiltrating adjacent nervous tissue. Complete surgical excision achieved immediate relief of the presenting signs. Histopathology and immunohistochemistry were consistent with a malignant atypical mesenchymal neoplasm, namely a telangiectatic osteosarcoma or a highly vascular poorly differentiated sarcoma. Surgery was combined with 3 × 30 mg/m^2^ doxorubicin/epirubicin and a 5 × 4 Gy radiotherapy protocol resulting in a long‐term successful outcome. The patient remains neurologically normal at the time of writing, 1 year after surgery. This case report demonstrates the value of surgery as a diagnostic tool in facilitating the histopathological examination of a spinal mass. Despite the suspected malignancy, surgery combined with chemotherapy and radiotherapy led to a successful outcome in our patient.

## 148 (P 121)

### 148.1 Congenital Internal and External Hydrocephalus in a Young Red Fox ( *Vulpes vulpes* ): CT Features and Post‐Mortem Findings

#### 148.1.1 F. Decrop, DVM, MRCVS,^1^ A. Ororbia Aranguren, DVM, MSc, MRCVS,^2^ C. Anselmi, DVM, DipECVDI, MRCVS,^2^ P. Álvarez Fernández, DVM, DipECVN, MRCVS, EBVS^1^

##### 148.1.1.1 ^1^ Neurology and Neurosurgery Service, Pride Veterinary Referrals, IVC Evidensia Group, Derby, UK; ^2^ Diagnostic Imaging Service, Pride Veterinary Referrals, IVC Evidensia Group, Derby, UK

Hydrocephalus is a common congenital malformation of the central nervous system in canines. The concurrent presence of external and internal hydrocephalus is rare in the veterinary literature and to our knowledge, this is the first case described in a fox diagnosed by CT study. A rescued estimated 2‐month‐old, male entire red fox, was presented with bilateral ventrolateral strabismus and domed‐shaped head. Neurological examination revealed a quiet mentation and bilateral absent menace response. CT study of the head demonstrated an abnormal expanding appearance of the calvarium with loss of the normal convolutional margins and open fontanelle in the right frontoparietal bone. There was bilateral widening of the subarachnoid space within the rostral cranial fossa, consistent with external hydrocephalus. A diffuse severe atrophy of the cerebral cortex and a defect of the roof of the left lateral ventricle suggested a potential communication with the subarachnoid space. There was bilateral marked, irregular, and asymmetrical enlargement of the lateral and third ventricles, compatible with internal hydrocephalus, and the absence of septum pellucidum. Three weeks after presentation, the fox acutely developed respiratory dyspnea and was euthanized. Post‐mortem examination confirmed the presence of transforaminal caudal cerebellar vermis herniation, diffused thickening of the dura mater, moderate bilateral subdural accumulation of xantochromic cerebrospinal fluid, with negative culture and marked diffuse cerebral cortical atrophy. A cleft was observed in the left parietal cortex connecting to the lateral ventricle. Congenital internal and external hydrocephalus should be considered a differential diagnosis in young foxes presenting with abnormal mentation and macrocephaly.

## 149 (P 122)

### 149.1 Surgical Resolution of Posttraumatic Frontoethmoidal Mucocele With Brain Involvement in a Cat

#### 149.1.1 M. Pérez Soteras, DVM,^1^ C. Morales Moliner, DVM,^1^ I. Velázquez‐Urgel, DVM, EVDC,^1^ C. Maeso Ordás, DVM^1^

##### 149.1.1.1 ^1^ Anicura Ars Veterinaria Hospital Veterinari, Barcelona, Spain

Sinusal mucoceles (SM) are epithelial‐lined mucus‐containing cysts filling nasal sinus, which can show an intracranial extension, and secondary to ostium obstruction, avoiding the drainage of mucus from the sinus. Description of SM is limited to few reports in both dogs and cats, with only two cats attributed to an idiopathic etiology. This case report describes presentation, findings and surgical outcome in a cat with a suspected post traumatic intracranial mucocele. A 3‐year‐old neutered male domestic short‐haired cat was presented for obtundation, and incoordination. History highlighted a previous cranial trauma 20 months before with deformation of craniofacial structures (absent ocular globes and frontoethmoidal defects). Neurological examination revealed an obtunded mental status, and proprioceptive ataxia in all four limbs. Neuroanatomic localization was forebrain. Computed tomography and magnetic resonance of the head showed multiple bony abnormalities as likely of a previous trauma, as well as a well delineated lesion involving the entire frontal sinus with intracranial extension to the frontal cortex, consistent with pyocele/mucocele. A transfrontal craniotomy was subsequently performed, removing a mucous filled capsule, enlarging the communication though the left ostium, and peeling the epithelium of the frontal sinus. Histopathologic analysis revealed an intense interstitial fibrosis with focal osteolysis and epithelial hyperplasia. Bacterial and fungal cultures were negative. A SM with intracranial extension intracranial was confirmed. No relapses have been reported after 2 years. In conclusion and to the authors' knowledge, this is the first case report describing the presentation, diagnosis, and surgical outcome of a SM with intracranial mucocele and traumatic etiology.

## 150 (P 123)

### 150.1 Treatment of Immune‐Mediated Polyneuropathy in Cats With Human Intravenous Immunoglobulins—A Case Series

#### 150.1.1 A. Schmitz, Resident ECVN,^1^ J. Van Renen, Resident ECVN,^1^ G. Buhmann, Dr. Med. Vet., Resident ECVN,^1^ S. Dörfelt, Dr. Med. Vet., Diplomate ECVN,^1^ A. Fischer, Prof., Dr. Med. Vet., Dr. Med. Vet. Habil. Diplomate ECVN^1^

##### 150.1.1.1 ^1^ Centre for Clinical Veterinary Medicine, Ludwig‐Maximilians‐University Munich, Munich, Germany

Introduction/Purpose: Immune mediated polyneuropathy (IMPN) is a common disease of the peripheral nervous system in young cats leading to generalized weakness. The clinical course is initially progressive. Affected cats develop paraparesis up to non‐ambulatory tetraparesis with reduced withdrawal reflexes. Most cats recover within 3–10 weeks, but longer recovery times may occur. Parallels to acute polyradiculoneuritis in dogs and Guillain‐Barrè syndrome (GBS) or juvenile chronic inflammatory demyelinating polyneuropathy (CIDP) in humans are discussed. Current reference treatments in humans with GBS and CIDP include intravenous immunoglobulins (IVIg). The study aim was to describe the clinical course of IMPN in cats following IVIg treatment.

Methods: Medical records were searched for cats with IMPN and IVIg treatment (2018–2022).

Results: Six cats (6 months to 2.5 years) with a clinical diagnosis of IMPN and treatment with IVIg because of severe or prolonged paresis were identified. All 6 cats were hospitalized and treated with human IVIg 0.5 g/kg/day intravenously following informed consent of the owners. IVIg were given as CRI for 6 h on 2 to 4 consecutive days. No side effects occurred. All 6 cats showed rapid clinical improvement (median: 2 days) after IVIg treatment, but 3 cats relapsed after 4–6 weeks.

Discussion/Conclusion: IVIg therapy may be considered as a rescue therapy in severely or chronically affected cats with IMPN. We cannot rule out that the cats would have recovered without treatment. Future studies with a larger cohort and controls could investigate the potential benefit of this therapy.

## 151 (P 124)

### 151.1 CT Characteristics of Piriformis Syndrome in a 3‐Year‐Old Whippet

#### 151.1.1 K. Bossens, MVM, Dipl. ECVN,^1^ M. Hermans, MVM, Dipl. ECVN,^1^ J. Van Ommen, DVM,^1^ S. Ooms, MVM,^1^ I. Alaerts, MVM,^1^ K. Kromhout, DVM^2^ ^,3^

##### 151.1.1.1 ^1^ Nesto Veterinary Referral Center Orion, Herentals, Belgium; ^2^ Department of Morphology, Imaging, Orthopedics, Rehabilitation and Nutrition Ghent University, Merelbeke, Belgium; ^3^ Kromhout Medical Imaging, Turnhout, Belgium

Piriformis syndrome is defined as non‐discogenic sciatic nerve entrapment in the subgluteal space and is thought to represent about 6%–8% of all cases of sciatica in human patients. This condition has not yet been described in dogs. The piriformis muscle contracts when dogs extend their hip while running and jumping. The overuse and secondary changes of this muscle can lead to irritation of the sciatic nerve due to its anatomical location deep in the muscle. A 3‐year‐old Whippet presented with monoparesis of the right pelvic limb. The symptoms started as acute lameness after strenuous activity 4 weeks earlier and slowly progressed to a painful monoparesis. Neurological examination showed proprioceptive deficits, a decreased withdrawal reflex, marked muscle atrophy and pain on hip extension of the right pelvic limb. Computed tomography showed an enlargement of the piriformis, gemelli, and internal obturator muscles with secondary impingement of the sciatic nerve. This caused a focal nerve swelling at the level of the ilium/coxo‐femoral joint region. This is in agreement with findings in humans where the piriformis muscle on the symptomatic side is significantly larger than on the asymptomatic side. To our knowledge this is the first report describing CT characteristics of piriformis syndrome in dogs. In conclusion, the close anatomical relationship between these deep gluteal muscles and the sciatic nerve may be a predisposing factor for neuritis in active dogs (e.g., agility or working dogs). Piriformis syndrome could be an overlooked condition and should be considered in dogs with pelvic limb lameness or paresis.

## 152 (P 125)

### 152.1 Prevalence of Spinal Disease Among Pugs and French Bulldogs in a UK Referral Setting

#### 152.1.1 S. Kerr, BVetMed MRCVS,^1^ L. Maskell, BVetMed MRCVS,^1^ E. Skovola, DVM MRCVS PgCert,^1^ S. De Decker, DVM PhD MVetMed DipECVN FHEA PgCert VetEd MRCVS^1^

##### 152.1.1.1 ^1^ Royal Veterinary College, University of London, Hatfield, UK

The aims of this study were to determine and compare the prevalence of the most common spinal disorders in Pugs and French bulldogs. This was a retrospective study including Pugs and French Bulldogs presenting to the Royal Veterinary College between January 2010 and March 2022 for investigations into clinical signs consistent with spinal disease. Patients were excluded if there were incomplete medical records, advanced imaging was not performed, clinical signs were not related to primary spinal disease or if the patient had multiple unrelated diagnoses. Exactly 439 French bulldogs with 449 diagnoses and 106 Pugs with 118 diagnoses were included in the study. Of these patients, 276 (63%) French Bulldogs had an acute onset of clinical signs, and 76 Pugs (72%) had a chronic onset of clinical signs. The most common disease in the French Bulldogs was Hansen type I intervertebral disc disease, (*n* = 377, 84.2%). The most common diagnosis among Pugs was spinal arachnoid diverticula (*n* = 30, 25.42%), followed by Hansen type II intervertebral disc disease (*n* = 24, 20.34%). The median age of French Bulldogs was 44 months, compared with a median of 71.5 months in Pugs. A significant association between both age and onset was found between breeds (*p* ≤ 0.001 for both). In conclusion, French Bulldogs are most commonly affected by acute spinal disorders, such as Hansen type I intervertebral disc disease, whereas Pugs are more often diagnosed with chronic spinal diseases, including spinal arachnoid diverticula and Hansen type II intervertebral disc disease. In addition, French Bulldogs presented younger than Pugs.

## 153 (P 126)

### 153.1 Radiographic and Computed Tomography of a Case of Equine Intervertebral Disc Disease and Cervical Vertebral Malformation With Postmortem MRI

#### 153.1.1 M. Mól, DVM, GPCert (Neuro), GPCert (DI), MRCVS,^1^ K. Garrett, DVM, DACVS‐LA,^2^ R. Ruby, MSc, BVSc, Diplomate ACVP, ACVIM‐LAIM,^3^ J. Janes, DVM, PhD, Dipl. ACVP,^3^ S. Reed, DVM, Diplomate ACVIM^2^

##### 153.1.1.1 ^1^ Paragon Veterinary Referrals, Wakefield, UK; ^2^ Rood & Riddle Equine Hospital, Lexington, Kentucky, USA; ^3^ Department of Veterinary Science, University of Kentucky, Lexington, Kentucky, USA

The aim of this study was to describe the presence of intervertebral disc herniation and cervical vertebral stenotic myelopathy, diagnosed with radiographic and CT myelography, confirmed with post‐mortem MRI and histopathology. A 3‐year‐old Saddlebred gelding presented with progressive tetraparesis, ataxia and cervical hyperaesthesia. Radiographic myelography showed spinal cord compression at C6–7 which was confirmed with CT myelography. Following euthanasia, post‐mortem MRI of the cervical vertebral column and histopathologic examination of the spinal cord and intervertebral disc was performed. CT myelography revealed ventral–dorsal myelocompression at C6–7, with enlarged articular processes and presumptive disc herniation. Post‐mortem MRI, confirmed myelocompression at C6–7, severe disc protrusion, articular process malformation, and secondary focal intramedullary changes. These changes were confirmed at autopsy with histologic evidence of compressive myelopathy at C6–7. This case report describes CT myelography, post‐mortem MRI, and autopsy findings in a horse with neurologic disease due to intervertebral disc degeneration and herniation causing compressive myelopathy. The authors believe this is the first case of equine intervertebral disc degeneration described with post‐mortem MRI and confirmed histopathologically, corresponding well to both radiographic and CT myelographic suspicion of intervertebral disc disease. This report provides the groundwork for future studies using MRI to evaluate horses with equine intervertebral disc degeneration, a condition that is thought to be rare.

## 154 (P 127)

### 154.1 Low‐Field Magnetic Resonance Imaging Patterns of Deep Surgical Site Infection After Decompressive Spinal Surgery in Dogs

#### 154.1.1 A. To, BVetMed,^1^ G. Bruto Cherubini, DVM DECVN,^2^ A. Caine, MA VetMB DECVDI^1^

##### 154.1.1.1 ^1^ Dick White Referrals, Newmarket, United Kingdom ^2^ University of Pisa, Pisa, Italy

Introduction: Surgical site infection (SSI) following spinal surgery is a daunting complication with significant morbidity to the animal and financial burden for the owners and the institution. The study aims to evaluate the low‐field magnetic resonance imaging pattern of deep surgical site infection after decompressive spinal surgery in dogs.

Method: Retrospective review of animal medical record and magnetic resonance imaging (MRI) of dogs with dogs that had MRI repeated after decompressive spinal surgery (hemilaminectomy or ventral partial corpectomy) and confirmed deep surgical site infection through positive bacterial culture from intra‐operative sampling of deep tissue were identified. The MRI from these patients was retrospectively reviewed by a single author for abnormalities that were not present on the initial MRI before surgery.

Results: Twenty‐three dogs met the inclusion criteria, MRI findings included T2‐weighted (T2W) hyperintensity of the subcutaneous (22/23) and muscle layers (23/23), T2W signal voids at the surgical site (19/23), T2W hyperintense fluid pockets (9/23), intervertebral disc space changes (4/23) and vertebral endplate changes (8/23). Length of intramedullary T2W hyperintensity on sagittal plane was increase in 10/23 cases when compared with pre‐operative MRI. Short‐tau inversion recovery sequence (STIR) was available in 16/23 cases; all cases had hyperintensity of the subcutaneous and muscle layers, in addition, 12/16 cases had evidence of hyperintense signal tracking through fascial planes.

Conclusion: A variety of MRI features may indicate deep surgical site infection after spinal surgery, these need to be interpreted carefully as some overlap with normal post‐operative MRI changes. Soft tissue adjacent the surgical field is more commonly affected than vertebral body or disc space on MRI.

## 155 (P 128)

### 155.1 Clinical Validation of a New Canine Radioimmunoassay for the Diagnosis of Myasthenia Gravis in Dogs

#### 155.1.1 H. Bauer, Neurologist,^1^ A. Schmitz, Resident ECVN,^1^ A. Pankraz, Dr., Diplomate ECVCP,^2^ S. Wildt, Scientist,^3^ T. Steinberg, Dr. Med. Vet., Diplomate ECVN,^4^ G. Buhmann, Dr. Med. Vet, Resident ECVN,^1^ J. Van Renen, Resident ECVN,^1^ A. Fischer, Prof., Dr. Med. Vet., Dr. Med. Vet.,^1,5,6^ Habil. Diplomate ECVN, K. Putschbach, Dr. Med. Vet., Diplomate ECVN, M. Kornberg, Dr. Med. Vet., Dipolomate ECVN, K. Jurina, Dr. Med Vet., Diplomate ECVN,^4^ H. Schenk, Dr. Med. Vet., Diplomate ECVN,^7^ M. Soeder, Neurologist,^8^ F. Wieländer, Dr. Med. Vet, Diplomate ECVN^9^

##### 155.1.1.1 ^1^ Centre for Clinical Veterinary Medicine, Ludwig‐Maximilians‐University Munich, Munich, Germany; ^2^ Biocontrol, Veterinary Division of Bioscientia Healthcare GmbH, Ingelheim, Germany; ^3^ Bioscientia Institute for Medical Diagnostics, Ingelheim, Germany; ^4^ AniCura Veterinary Hospital Haar, Haar, Germany; ^5^ Veterinary Health Center Piding, Piding, Germany; ^6^ AniCura Veterinary Hospital Trier, Trier, Germany; ^7^Veterinary Hospital Lüneburg, Lüneburg, Germany; ^8^ Veterinary Hospital Lademannbogen, Hamburg, Germany; ^9^ Veterinary Hospital Oberhaching, Oberhaching, Germany

Introduction/Purpose: Acquired myasthenia gravis is an autoimmune disease of the neuromuscular endplate. The characteristic sign is exercise‐induced weakness, frequently in association with megaesophagus. Affected dogs can present as neurologic emergencies. The diagnostic gold standard is the measurement of serum acetylcholine‐receptor‐antibodies by radioimmunoassay. The availability of this test is limited in Europe leading to unnecessary delay in diagnosis and treatment. The aim of this study was to provide data on the performance of a new canine radioimmunoassay for measurement of acetylcholine receptor antibodies in dogs.

Methods: Acetylcholine receptor antibodies were measured in serum samples from 238 dogs (16 myasthenia gravis, 222 controls) with a radioimmunoassay designed for use in dogs (Canine ACHRAB RIA; DLD Diagnostika, Hamburg, Germany). Samples from 40 dogs with other neuromuscular diseases, 147 dogs with other diseases and 35 healthy dogs served as controls. Serum acetylcholine receptor antibodies were also measured in the reference laboratory in 70 dogs (16 myasthenia gravis, 54 controls; neuromuscular diagnostic laboratory, San Diego, USA).

Results: ROC analysis identified an optimal cut‐off of 2.0 nmol/L, whereby the Canine ACHRAB RIA showed a sensitivity of 100%, a specificity of 99.6%, a PPV of 94.1%, and a NPV of 100.0%. A cut‐off of 1.3 nmol/L showed a sensitivity 100%, specificity of 98.2, PPV 80.0%, and NPV 100.0%.

Discussion/Conclusion: The new Canine ACHRAB RIA is well suited for the diagnosis of myasthenia gravis in dogs. The availability of this test improves diagnosis and treatment of dogs with myasthenia gravis in Germany.

## 156 (P 129)

### 156.1 Dropped Jaw Secondary to Masticatory Muscle Myositis in a Cat

#### 156.1.1 E. Robinson, BVetMed MRCVS,^1^ C. Briola, DVM MRCVS DipECVDI,^2^ M. I. De Freitas, MVMed DVM MRCVS^1^

##### 156.1.1.1 ^1^ Neurology‐Neurosurgery Service, The Ralph Veterinary Referral Centre, Marlow, UK; ^2^ Diagnostic Imaging Service, The Ralph Veterinary Referral Centre, Marlow, UK

A 3‐year‐old male‐neutered domestic shorthair cat was presented for inability to fully close the mouth, impaired food prehension and weight loss. Neurological examination revealed markedly reduced temporal and masseter muscle mass bilaterally and reduced jaw tone with a dropped jaw position. Creatinine Kinase (CK) was significantly elevated at > 20 000 U/L (range 0–360 U/L). The MRI showed atrophy of the masseter and temporal muscles, diffuse bilateral and symmetric T2W, FLAIR, and STIR hyperintensity, T1W isointensity, and marked contrast‐enhancement of the temporal, masseter, digastric, and pterygoideus medialis muscle, most likely compatible with masticatory muscle myositis (MMM). Serology for Feline Leukemia Virus, Feline Coronavirus, and Toxoplasma gondii was negative and cerebrospinal fluid analysis was unremarkable. Electromyography was normal when assessing the axial and appendicular muscles and showed reduced insertional activity in the masticatory muscles. Histopathological analysis of masseter and temporal muscle biopsies revealed severe, multifocal, chronic, atrophic, lymphoplasmacytic myositis, in addition to the presence of neutrophils and eosinophils. These results were also highly suggestive of primary immune‐mediated MMM. Immunosuppressive treatment was instigated with a tapering course of prednisolone (2 mg/kg q24h), resulting in the resolution of clinical signs within 72 h. To the author's knowledge there are currently only two reports of feline masticatory myositis: one presenting with trismus, and one with bilateral exophthalmos and normal jaw mobility. To the author's knowledge, this is the first report of a cat with MMM presenting with dropped jaw, which should be considered as a differential for this condition.

## 157 (P 130)

### 157.1 Clinical Resolution With Clindamycin of a Large Intra‐Axial Thalamic Serologically Suspected Toxoplasma Granuloma in a Cat

#### 157.1.1 E. Robinson, BVetMed MRCVS,^1^ V. Volckaert, DVM MRCVS PhD DipECVDI,^2^ L. Mari, DVM MRCVS Dip. ECVN^1^

##### 157.1.1.1 ^1^ Neurology Department, The Ralph Veterinary Referral Centre, Marlow, UK; ^2^ Diagnostic Imaging Service, The Ralph Veterinary Referral Centre, Marlow, UK

Toxoplasma gondii has a predilection for the central nervous system and can respond to appropriate antibiotics. However, only one report has previously documented clinical and MRI resolution of a cerebral extra‐axial suspected toxoplasma granuloma in a medically treated cat. A 3‐year‐old male‐neutered domestic shorthair cat presented with acute vestibular ataxia. Neurological examination showed obtunded mentation, left head‐tilt, right circling, decreased proprioception on the left limbs and absent left menace response. Neuroanatomical localization was right forebrain, suspected diencephalon. Hematology and serum biochemistry were unremarkable. MRI revealed a large, spherical (1 cm‐diameter), well‐defined, intra‐axial mass within the right diencephalon, with perilesional oedema causing severe mass effect. The mass was isointense to gray matter in T2W, FLAIR and GRE, and hypointense on T1W, with granular appearance and marked homogeneous contrast enhancement. Thoracic and abdominal CT was unremarkable. Serology for cryptococcus and coronavirus was negative. Toxoplasma serology showed markedly positive IgG titer (1:6400, maximum dilution tested) and absent IgM. Treatment for suspected toxoplasma granuloma was instigated with clindamycin (18 mg/kg q12h) and a short course of prednisolone (0.5 mg/kg q24h, tapered over 2 weeks). Following a marked initial deterioration in the first 24 h, the cat improved to discharge and was neurologically normal at his 3‐week‐recheck, where IgG remained positive to maximum dilution tested. Repeat MRI after 8 weeks of treatment confirmed significant reduction of the lesion (3 mm‐diameter). Clindamycin was continued, with ongoing follow‐up. This is the first case documenting clinical resolution and MRI improvement of an intra‐axial suspected toxoplasma granuloma with medical management in a cat.

## 158 (P 131)

### 158.1 Spinal Cord Dysfunction Caused by *Cladophialophora bantiana* Infection in a Dog and Cat

#### 158.1.1 L. McLeay, BVSc, BVMS,^1^ P. Kenny, BVSc (Hons), DipACVIM (Neurology), DipECVN, FHEA, MRCVS,^1^ G. Child, BVSc, DACVIM (Neurology),^1^ S. Donahoe, DVM, PhD, DACVP,^3^ A. Lam, BVSc (Hons),^1^ MANZCVS, MRCVS, FANZCVS (SA Medicine),^1^ E. Jenkins, BVSc (Hons),^1^ PGCertVS, MVS, MANZCVS,^2^ A. Taylor, BVSc, FANZCVS (SA Medicine),^2^ M. Krockenberger, BSc (Vet), BVSc, PhD, FANZCVS (GradCertEdStud),^3^ P. Martin, BVSc, MVSc^3^

##### 158.1.1.1 ^1^ Small Animal Specialist Hospital, Sydney, Australia; ^2^ Sydney School of Veterinary Science, University of Sydney, Sydney, Australia; ^3^ Veterinary Pathology Diagnostic Services, Sydney School of Veterinary Science, The University of Sydney, Sydney, Australia

*Cladophialophora bantiana* is a neurotropic phaeohyphomycotic organism. The most common manifestation of *C. bantiana* infection is brain abscessation or systemic phaeohyphomycosis. *C. bantiana* infection with spinal cord involvement has not yet been reported in domestic animals. This report describes two cases of spinal cord dysfunction caused by *C. bantiana* infection in a dog and a cat. A 10‐month‐old male cavalier was presented for acute onset tetraparesis and cervical pain. Magnetic resonance imaging identified a focal ring contrast enhancing intramedullary lesion in the cervical spinal cord. The dog was treated empirically with antibiotics and corticosteroids but clinically deteriorated and was humanely euthanized. PCR of tissue samples of the lesion collected via post‐mortem examination identified *C. bantiana*. The second case, a 4‐year‐old male neutered domestic short hair cat was presented with non‐ambulatory paraparesis. The cat was humanely euthanized and post‐mortem examination revealed an extramedullary mass effacing the dorsal process of the 12th thoracic vertebra causing spinal cord compression. Lesions were also found in the kidneys, spleen, and lungs. Fungal hyphae were identified in the urine, and PCR of all tissue samples identified *C. bantiana*. These cases demonstrate two manifestations by which *C. bantiana* infection may cause spinal cord dysfunction: primary spinal cord lesion or vertebral lesion causing spinal cord compression. The route of infection is most likely via inhalation of fungal spores or inoculation of wounds. *C. bantiana* should be considered as a possible cause of spinal cord dysfunction in cats and dogs.

## 159 (P 132)

### 159.1 Can ChatGPT Diagnose my Collapsing Dog?

#### 159.1.1 S. Abani, DVM,^1^ ^,2^ S. De Decker, DVM, PhD, MvetMed, DipECVN, FHEA, PGCert Veted, MRCVS,^3^ A. Tipold, Prof., Dr. Med. Vet., Dipl ECVN,^1^ J. Nessler, Dr. Med. Vet., Dipl ECVN,^1^ H. Volk, Prof., PhD, PGCAP, DipECVN, FHEA, MRCVS^1^ ^,2^

##### 159.1.1.1 ^1^ Department of Small Animal Medicine and Surgery, University of Veterinary Medicine Hanover, Hannover, Germany; ^2^ Centre for Systems Neuroscience, University of Veterinary Medicine Hannover, Hannover, Germany; ^3^ Department of Clinical Sciences and Services, Royal Veterinary College, London, UK

The study aimed to discuss the potential advantages and challenges if artificial intelligence‐powered Language Models like ChatGPT can be used as a diagnostic tool by pet owners or veterinarians.

Data of twenty dogs with episodic disorders (idiopathic epilepsy, structural epilepsy, paroxysmal dyskinesia, and syncope, each *n* = 5) were randomly selected for stepwise evaluations. First only signalment and case history was entered. In the second step additional clinical examination was given by conducting a separate chat sessions. In the first evaluation, based solely on case histories, ChatGPT correctly identified idiopathic epilepsy *n* = 2/5, structural epilepsy *n* = 5/5, paroxysmal dyskinesia *n* = 0/5, and syncope *n* = 4/5. In the subsequent evaluation, when clinical examinations were included, it correctly identified idiopathic epilepsy *n* = 4/5, structural epilepsy *n* = 5/5, paroxysmal dyskinesia *n* = 0/5, and syncope in *n* = 3/5. In a second evaluation the grammar and choice of words from the medical records were slightly modified and the same approach was conducted again. Sole on signalement and history, ChatGPT correctly identified idiopathic epilepsy *n* = 0/5, structural epilepsy *n* = 1/5, paroxysmal dyskinesia *n* = 1/5, and syncope *n* = 5/5. When clinical examinations were included, it correctly identified idiopathic epilepsy *n* = 3/5, structural epilepsy *n* = 1/5, paroxysmal dyskinesia *n* = 1/5, and syncope *n* = 4/5. ChatGPT's diagnostic capabilities rely on factors like information presentation, disease, and online data availability. Diseases that are better known to neurologists than to the general public, such as dyskinesia, are particularly problematic. Nevertheless, there is considerable potential for utilizing Language Models to improve clinical decision‐making and optimize the clinical workflow in veterinary medicine.

## 160 Flash Presentations

## 161 (FP 01)

### 161.1 Modified Unilateral Dorsal Laminectomy for Lateralized C2–T1 Intervertebral Disc Extrusions in Dog

#### 161.1.1 K. Santifort, DVM, MSc, ECVN Eligible,^1,2^ M. Plonek, DVM, PhD, Res ECVN, ^1^ E. Glass, DVM, MSc, Dipl. ACVIM (Neurology)^3^

##### 161.1.1.1 ^1^ IVC Evidensia Small Animal Referral Hospital, Arnhem, The Netherlands; ^2^ IVC Evidensia Small Animal Referral Hospital Hart Van Brabant, Waalwijk, The Netherlands; ^3^Red Bank Veterinary Hospital, Section of Neurology and Neurosurgery, Tinton Falls, New Jersey, USA

Several surgical approaches have been described for the treatment of C2–T1 intervertebral disc extrusions (IVDE), including a ventral approach and median manubriotomy or manubriectomy to perform a ventral slot technique; a dorsal approach and dorsal laminectomy or hemilaminectomy; a lateral approach and hemilaminectomy; and a modified dorsal approach and hemilaminectomy. We describe the application of a dorsal approach and modified unilateral dorsal laminectomy for lateralized C2–T1 IVDE (LIVDE) in dogs. Patients from two institutions in which a cervical LIVDE was diagnosed by MRI and where a modified unilateral dorsal laminectomy was performed were included in this descriptive study. Briefly, the technique involves the removal of the (dorsal) lamina of one or more cervical vertebrae where the medial landmark is the spinous process or midline raphe and the lateral landmark is the tubercle on the caudal articular process. Three dogs (age 4–8.5 years, weight 19–23 kg) were presented for acute‐onset upper motor neuron tetraparesis, general proprioceptive ataxia, and severe cervical hyperesthesia persisting despite medical treatment. Respective diagnoses were: C3–C4, C6–C7, and C7–T1 LIVDE with or without hemorrhage. All three dogs recovered uneventfully and were ambulatory the day after surgery. This surgical technique provides excellent access to lateralized compressive lesions of the spinal cord such as LIVDE. Specific benefits include: avoidance of injury to the vertebral artery; only soft tissue dissection is required; and drilling over the compressive lesion which decreases the risk of injuring the spinal cord. It can be considered an alternative to previously described techniques.

## 162 (FP 02)

### 162.1 Clinical Signs of 166 Beagles With Different Genotypes of Lafora Disease

#### 162.1.1 J. Dietzel, DVM, ^1^ C. Dirauf, MSc, ^2^ A. Kehl, Biologist, ^2^ A. Holtdirk, Statistician,^3^ I. Langbein‐Detsch, Biologist,^2^ E. Müller, DVM, ^2^ T. Flegel, Diplomate ECVN, Diplomate ACVIM (Neurology)^1^

##### 162.1.1.1 ^1^Department for Small Animals, Faculty of Veterinary Medicine, University of Leipzig, Leipzig, Germany; ^2^Department of Molecular Biology, Laboklin GmbH & Co. KG, Bad Kissingen, Germany; ^3^ CRO Dr. Med. Kottmann GmbH & Co. KG, Hamm, Germany

Lafora disease (LD) is a genetic disorder with autosomal recessive inheritance affecting the NHLRC1 gene. Affected beagles can show a variety of neurological symptoms. The aim of this study was to describe the phenotypic expression of different NHLRC1 genotypes in beagles. Based on this, we analyzed whether the clinical signs can be used to draw conclusions about the Lafora genotype. Owners of 166 Beagles that had been tested for the presence of the NHLRC1 gene defect participated in an online survey about clinical signs of LD in their dog. Questions focused on: myoclonic or generalized epileptic seizures, neurological deficits, changes in mental function, and behavioral changes. Answers were recorded for the three possible genotypes (L/L, L/N, N/N) in two age groups (< 6 years and = 6 years). The extent to which a combination of clinical signs can indicate the presence of LD was investigated. Most clinical signs of LD were observed regardless of the NHLRC1 genotype. In the age group = 6 years they were significantly more frequent in beagles with the L/L genotype compared with L/N and N/N genotypes. A total of 99.4% of beagles aged = 6 years were correctly assigned to the L/L genotype if all of the following three clinical signs occurred: head jerking, photosensitivity, and loss of learned skills. If one or two of these signs were missing, correct classification decreased to 92.1% and 13.2%, respectively. The presence of these signs can give a strong presumptive diagnosis but a definitive confirmation can only be provided via genetic testing.

## 163 (FP 03)

### 163.1 Focal Epilepsy or Movement Disorders? Interictal Paroxysmal Discharges During STI‐EEG Recordings in 4 Dogs

#### 163.1.1 M. Opreni, DVM, GpCert, ^1^ A. Di Paola, DVM, ^1^ A. Sala, DVM, ^1^ P. Favole, DVM, ^1^ A. Cauduro, DVM, DiplECVN^1^

##### 163.1.1.1 ^1^Neurovet, Milano, Italia Epilepsy (IE) in the Clinical Form of Focal Seizures Can Be Challenging to Distinguish From Other Paroxysmal Events of Movement Disorders (MD)

The aim of this work is to share the authors' experience in some clinical cases with the short‐time interictal EEG (STI‐EEG), where the detection of paroxysmal signs even during the interictal phase (paroxysmal discharge, PD) can help direct the diagnosis of IE versus MD. Between August 2022 and January 2023 dogs meeting the following inclusion criteria were selected: diagnostic tests according to the TIER 2 confidence level for IE, availability of multiple videos with episodes consistent with each other and compatible with focal epileptic seizure. In the cases included, a sti‐EEG was acquired using sedation with dexmedetomidine, IV anesthesia with propofol, and the method described above. Qualitative analysis of STI‐EEG traces was performed by a board‐certified neurologist. Four cases were selected. During the episodes (attached videos), the mental status was normal or with questionable depression, and no autonomic or postictal signs were identified. Three dogs showed involuntary muscle contractions associated with postural alterations of limbs and trunk, in one case a dog showed a sudden muscle tone loss. The qualitative STI‐EEG trace (attached photos) analysis evidenced paroxysmal alterations in all patients suggesting the introduction of an antiepileptic treatment. The patients' follow‐ups (5–9 months) evidenced a reduction of episodes in intensity and frequency. The use of a TIER 3 confidence level method, as the STI‐EEG, can be useful and should be recommended in the interpretation of episodes of doubtful nature such as focal epileptic seizures. Future controlled and standardized studies are required to emphasize the different patterns in IE.

## 164 (FP 04)

### 164.1 Focal Spinal Hyperesthesia as a Prognostic Factor in Paraplegic Dogs Without Deep Pain Perception

#### 164.1.1 M. Wrzesinski, BSc, MSc, ^1^ M. Schwab, BSc, MSc, PhD, ^1^ D. Ferrarin, BSc, MSc, PhD, ^1^ D. Beckmann, BSc, MSc, PhD, ^2^ J. Rauber, BSc, MSc, ^1^ J. Chaves, BSc, MSc, ^1^ R. Schamall, BSc, MSc, ^3^ A. Mazzanti, BSc, MSc, PhD^2^

##### 164.1.1.1 ^1^Graduate Program in Veterinary Medicine, Veterinary Neurology and Neurosurgery Service, Universidade Federal de Santa Maria, Santa Maria, Rio Grande do Sul, Brazil; ^2^Department of Small Animal Clinic and Surgery, Veterinary Neurology and Neurosurgery Service, Universidade Federal de Santa Maria, Santa Maria, Rio Grande do Sul, Brazil; ^3^Clínica Veterinária Petrópolis, Petrópolis, Rio de Janeiro, Brazil

Intervertebral disc extrusion (IVDE) is the most common cause of compressive injuries in the spinal cord of dogs, whose prognosis is variable and depends on several factors, being deep pain perception (DPP) is considered the main parameter. Studies on new prognostic factors are assessed to provide more accurate estimates of functional recovery. Therefore, this study aimed to evaluate whether spinal hyperesthesia (SH) at the site of compression can be used as a prognostic factor for functional recovery of dogs with IVDE without DPP undergoing thoracolumbar hemilaminectomy. Among the 68 dogs included in this retrospective study, 73.5% (50/68) presented SH, and in 26.5% (18/68), the pain was not identified. Recovery was satisfactory in 60% (30/50) of dogs with SH, and only in 27.7% (5/18) of dogs without SH, indicating that paraplegic dogs without DPP, but with the presence of SH, have 2.2× more chances of recovering when compared with dogs without SH. The presence of SH in dogs without DPP may suggest the existence of sensory tracts, as well as preserved motor tracts at the injury site, which increase the chances of functional recovery. No studies were found that evaluated the SH measuring palpation of the spine as a prognostic factor, which reinforces the relevance of this study. In conclusion, spinal hyperesthesia at the site of compression in paraplegic dogs with thoracolumbar IVDE, without DPP, can be used as a possible prognostic indicator of functional recovery.

## 165 (FP 05)

### 165.1 Central Nervous System Vascular Complications Associated With the Acute Form of Steroid Responsive Meningitis‐Arteritis

#### 165.1.1 C. Mayor, DVM,^1,2^ C. de la Fuente, PhD, Dipl. ECVN,^1,3^ M. Reus, DVM, ^3^ S. Añor, PhD, Dipl. ACVIM (Neurology), Dipl. ECVN^1^ ^,2^

##### 165.1.1.1 ^1^Fundació Hospital Clínic Veterinari, Universitat Autònoma de Barcelona, Barcelona, Spain; ^2^Departament de Medicina i Cirurgia Animals, Facultat de Veterinària, Universitat Autònoma de Barcelona, Barcelona, Spain; ^3^Hospital Veterinari Montjuïc (VetPartners), Barcelona, Spain

This retrospective study aims to describe vascular complications within the central nervous system (CNS) associated with the acute form of steroid‐responsive meningitis‐arteritis (SRMA), to compare clinical features of dogs with and without such complications, and to potentially identify predisposing factors. Dogs with a presumptive diagnosis of SRMA visited between 2018 and 2023 with full medical records that underwent neurological examination, blood testing, cervical computed tomography or magnetic resonance imaging (MRI), and cerebrospinal fluid (CSF) analysis were included. The diagnosis was reached by the presence of consistent clinical signs, neutrophilic pleocytosis, and/or compatible MRI findings and favorable outcomes with corticosteroid immunosuppressive treatment. Thirty dogs were included and divided into two groups. Group 1 included 5 (17%) cases with vascular complications secondary to SRMA (spinal cord ischemic and/or hemorrhagic infarcts, spinal cord subdural hematomas, intracranial subarachnoid hemorrhages), and Group 2 included 25 (83%) non‐complicated SRMA cases. Age, breed, sex, duration of clinical signs before diagnosis, presence of neurologic deficits, CSF pleocytosis and xanthochromia, presence of relapses or comorbidities, and treatment duration were factors evaluated for potential association with vascular complications of SRMA. Neurologic deficits (*p* = 0.003) and xanthochromic CSF (*p* = 0.00) were significantly associated with the occurrence of vascular complications. Although the breed was not found to be statistically significant (*p* = 0.119), Golden Retrievers were overrepresented in group 1 (4/5 dogs). Hemorrhagic or ischemic lesions within the CNS occurred in 17% of all SRMA cases, which presented with neurologic deficits upon examination and xanthocromic CSF. Larger studies are needed to ascertain breed predisposition or other associated factors.

## 166 (FP 06)

### 166.1 Phenotypic and Genetic Characterization of a Novel Hypomyelinating Leukodystrophy Affecting Cocker Spaniels

#### 166.1.1 R. Gutierrez Quintana, MVZ, MVM, MRCVS, ^1^ P. Montague, PhD, ^2^ A. Rupp, DVM, PhD, ^1^ J. Penderis, BVSc, MVM, PhD, ^3^ N. Byrne, DVM, ^4^ V. Gonzalo Nadal, DVM, ^1^ J. Edgar, PhD,^2,5^ M. McLaughlin, PhD^1^ ^,5^

##### 166.1.1.1 ^1^School of Biodiversity, One Health and Veterinary Medicine, College of Medical, Veterinary and Life Sciences, University of Glasgow, Glasgow, UK; ^2^School of Infection and Immunity, College of Medical, Veterinary and Life Sciences, University of Glasgow, Glasgow, UK; ^3^Vet‐Extra Neurology, Stirling, UK; ^4^Oban Vets, Oban, UK; ^5^Shared Senior Authorship, Glasgow, UK

Leukodystrophies are hereditable disorders of humans and animals that affect white matter. Here we describe a family of Cocker Spaniels with a hypomyelinating leukodystrophy caused by a novel variant in the proteolipid protein 1 (Plp1) gene. A litter of Cocker Spaniels presented with severe generalized coarse body tremors affecting three of five puppies. Tremors started at 2 weeks of age and disappeared at rest. One male and two females were affected. The male dog was significantly smaller and had more severe tremors. Neurological examination showed mild hypermetria with the rest being unremarkable for the age. Magnetic resonance imaging in the three affected puppies showed diffuse white matter hyperintensity in T2WI. Both female puppies gradually improved with no more tremors observed from 2 months of age, but the male puppy continued deteriorating and was euthanized at 5 weeks of age. The post‐mortem examination revealed a decrease in central nervous system myelin combined with a decrease in white matter cellularity, compatible with hypo‐/dysmyelination. The spinal cord was more affected than the brain, while the myelin in the peripheral nervous system was normal. Preliminary analysis of myelin failed to detect myelin proteolipid protein (PLP‐1/DM20), with low levels of myelin basic protein although PLP/DM20 mRNA was detected. X‐linked recessive inheritance was suspected and the Plp1 gene was considered a candidate gene. A novel likely pathogenic variant in the Plp1 gene was identified, NM_001013834 (cT>A66). To our knowledge, this is the second report of a Plp1‐related disease in dogs, representing a potential spontaneous large animal model.

## 167 (FP 07)

### 167.1 Non‐Invasive Monitoring of Intracranial Pressure Waveforms in Cats Submitted to Three Anesthetic Protocols

#### 167.1.1 G. D. L. Bernardes, MSc, ^1^ M. V. Bahr Arias, PhD, ^2^ L. M. Matsubara, PhD, ^2^ G. S. Cardoso, PhD, ^2^ I. S. Santos, MSc, ^1^ D. N. Smanioto, MSc, ^2^ K. A. Yaekashi, BSc^3^

##### 167.1.1.1 ^1^Postgraduate Program in Animal Health Science, Londrina State University, Londrina, Brazil; ^2^Professor, Department of Veterinary Clinical Sciences, Londrina State University, Londrina, Brazil; ^3^Small Animal Surgery Veterinary Residency Program, Londrina State University, Londrina, Brazil

The Brain4care® BcMM 2000, a non‐invasive intracranial pressure waveform monitor (ICP‐NI), has shown promise in humans with intracranial disorders, but its application in cats is limited. This study aimed to evaluate the ICP‐NI and P2/P1 wave ratio in healthy cats undergoing elective orchiectomy. Twenty‐five client‐owned cats were divided into three groups: inhalation anesthesia (INA) with eight cats, dissociative anesthesia (DIS) with eight cats, and propofol anesthesia (PRO) with nine cats. Monitoring was performed with a strain gauge sensor during the preoperative period (M1) in the PRO group, and both pre‐ and postoperative periods (M1 and M2) in the INA and DIS groups. Complete data on the P2/P1 ratio values were obtained for 17 cats. In the M1 period, the P2/P1 ratios were 0.882 ± 0.268 (INA group), 1.030 ± 0.17 (DIS group), and 1.16 ± 0.28 (PRO group). The INA group showed a significant increase in the P2/P1 ratio during the M2 period (M1: 0.88 ± 0.26 vs. M2: 1.23 ± 0.09; *p* = 0.016). Failure to collect data from one‐third of the cats was attributed to technical issues with the monitor. The DIS group exhibited less variation in wave patterns and brain compliance, but all three anesthesia protocols affected ICP wave dynamics. Since there are no established values for the P2/P1 ratio in cats using this device, the data obtained in this study are likely normal or elevated due to the anesthetic protocols. These findings can serve as reference parameters for future studies involving cats with intracranial disorders using this monitor.

## 168 (FP 08)

### 168.1 Clinical Outcome of Pugs and French Bulldogs With Thoracolumbar Spinal Deformities Causing Neurological Signs Teated With Spinal Stabilization Using 3D Printed Patient‐Specific Drill Guides

#### 168.1.1 F. Violini, DVM MRCVS, ^1^ J. H. Elford, BVetMed MRCVS DipECVN, ^1^ S. Bertram, DVM MVetMed DipECVN MRCVS, ^2^ T. J. A. Cardy, BSc BVetMed (Hons) MVetMed PhD DipECVN MRCV, ^2^ B. Oxley, MA VetMB DSAS (Orth) MRCVS, ^3^ A. Craig, VSc PGCert (SAS) DipECVS MRCVS, ^4^ S. Behr, DVM DipECVN MRCVS^1^

##### 168.1.1.1 ^1^Willows Veterinary Centre and Referral Service, Solihull, UK; ^2^Cave Veterinary Specialists, Somerset, UK; ^3^Vet 3D, Cumbria, UK; ^4^North Downs Specialist Referrals, Bletchingley, UK

The aim of the study was to describe the clinical outcome of Pugs and French Bulldogs with congenital vertebral malformations undergoing thoracolumbar spinal stabilization surgery using 3D‐printed patient‐specific drill guides. Pugs and French Bulldogs presented to three referral centers (August 2018 to March 2021) diagnosed with congenital vertebral abnormalities via MRI scan and CT scan causing T3–L3 myelopathy signs that underwent spinal stabilization surgery using 3D printed drill guides followed by a post‐operative CT scan were included. Data from the post‐operative follow‐up was used to evaluate the short‐term outcome of these patients; the long‐term outcome was obtained via an online questionnaire. A total of 22 dogs met the inclusion criteria. A total of 196 pedicle screws were placed and no major perioperative complications were reported. Optimal pedicle screw placement was obtained in 82% of screws. Exactly 24 h after the surgery 82% of dogs were categorized as stable. Short‐term outcome was available for 20 dogs and revealed a stable (17) or improved (2) neurological grade. Twelve owners participated in the online questionnaire. All patients were reported to be ambulatory, however, 8 dogs displayed abnormal gait. Most owners felt their dog's neurological signs had remained stable (50%) or improved (33%) since their last follow‐up appointment. Thoracolumbar spinal stabilization using 3D‐printed patient‐specific drill guides is a safe technique to treat brachycephalic breeds affected by spinal deformities. Dogs in this study had a good short‐term outcome and long‐term outcome evaluation revealed a stable/improved neurological status.

## 169 (FP 09)

### 169.1 Medium‐Term Recurrence in Arachnoid Diverticula Undergoing Durotomy Alongside Subdural Shunt‐Placement

#### 169.1.1 S. A. Gomes, DVM MRes DipECVN MRCVS, ^1^ M. Targett, MA VetMB PhD DipECVN SFHEA MRCVS, ^2^ S. Longo, DVM MRCVS, ^1^ M. Green, BVM BVS MRCVS, ^1^ T. Mignan, BVM BVS DipECVN MRCVS, ^1^ H. Gunovska, DVM MRCVS, ^1^ M. Lowrie, MA VetMB MVM DipECVN MRCVS^3^

##### 169.1.1.1 ^1^Dovecote Veterinary Hospital, Castle Donington, UK; ^2^School of Veterinary Medicine and Science, University of Nottingham, Sutton Bonington, UK; ^3^Movement Referrals: Independent Veterinary Specialists, Preston Brook, UK

Introduction/Purpose: Subdural shunt‐placement may have utility in the management of arachnoid diverticula (AD). Recurrence postoperatively is a recognized problem, with rates reported from 20% to 86%. Shunt‐placement could potentially prevent a recurrence. The aim of this study is to report recurrence in AD surgically managed cases when shunt‐placement is employed.

Methods: Retrospective non‐randomized case–control study. Medical records of a single referral hospital were searched for AD surgically managed cases with durotomy alone (named controls) and with shunt‐placement, with over 6 months of follow‐up time. Recurrence was defined as neurological signs attributable to a suspected or confirmed AD reformation, after documented improvement or stable neurological status after surgery. Recurrence information was collected through telephonic interviews with the owners or the referring veterinarians.

Results: Twenty‐three dogs were included; 13 controls and 8 with shunt‐placement. Control‐dogs median age at presentation was 79 months (16–118); breed distribution was 8 pugs, 4 French bulldogs, and 1 crossbreed. Shunt‐placement dogs median age at presentation was 67.5 months (47–84); breed distribution was 4 pugs and 4 French bulldogs. Median follow‐up time was 24 months (8–55) for controls and 17.5 months (12–48) for shunt. The recurrence rate was 6/13 (46.15%) in controls and 1/8 (12.5%) with a shunt. Median time to recurrence was 24.5 months (9–62) for controls and 29 months with shunt.

Discussion/Conclusion: Recurrence is less prevalent in cases with AD where durotomy and debridement of subdural adhesions alongside shunt‐placement has been undertaken. Despite the limited number of cases, shunt‐placement has very likely a positive role in preventing recurrence in cases of AD.

## 170 (FP 10)

### 170.1 Lateralized Herniation of Intervertebral Disc Material Through Previous Prophylactic Fenestration Site Could Prevent Recurrence of TL‐IVDE

#### 170.1.1 G. Albertini, DVM, MRCVS, ^1^ O. Marsh, BVM, BVS, MRCVS, ^2^ A. Uriarte, DVM, MRCVS, DECVN, ^1^ F. Stabile, DVM, PhD, MRCVS, DECVN^1^

##### 170.1.1.1 ^1^Southfields Veterinary Specialists, Basildon, UK; ^2^Dick White Referrals, Six Mile Bottom, UK

Prophylactic intervertebral disc (IVD) fenestration (PIVDF) has been reported to reduce the risk of recurrence of thoracolumbar‐IVD extrusion (TL‐IVDE) in chondrodystrophic breeds. Currently, no follow‐up information is available on IVD that undergoes prophylactic fenestration. The purpose of this retrospective case series is to assess if further IVD material herniates through the PIVDF site and evaluate its importance in the prevention of TL‐IVDE recurrences. Medical records of a single institution from January 2020 to March 2023 were retrospectively reviewed for dogs who underwent repeated thoraco‐lumbar MRI for a suspected recurrence of IVDE, after a previous decompressive thoracolumbar spinal surgery and PIVDF. Nine dogs met the inclusion criteria. IVDE recurrence occurred once in 6/9 (66.7%) dogs, twice in 2/9 (22.2%) dogs, and three times in 1/9 (11.1%) dogs. A total of 42 IVDs were fenestrated. Of these, 33/42 (78.6%) IVDs were included on repeated MRIs. The mean time between repeated MRIs was 4.5 months (0.5–23 months). IVDE recurrence occurred at a fenestrated IVD in 5/13 (38.5%) IVDEs, at an IVD adjacent to a previously fenestrated IVD in 2/13 (15.4%) IVDEs and > 1 IVD away from a previously fenestrated IVD in 6/13 (46.2%) IVDEs. No herniation of IVD material through the PIVDF site was seen in 13/33 (24.2%) IVDs. In 5 of these (38.5%) an acute IVDE was observed dorsally to the IVD causing spinal cord compression. In 20/33 (60.6%) IVDs, IVD material was herniated laterally through the previous PIVDF site with no spinal cord/spinal nerve compression. PIVDF often resulted in lateralized IVD material herniation through the PIVDF site likely preventing TL‐IVDE recurrence at that level.

## 171 (FP 11)

### 171.1 Dorsal Laminectomy for the Treatment of Lateralized Cervical Intervertebral Disc Extrusions in Dogs—Complications and Prognosis

#### 171.1.1 D. Gouveia, DVM, ^1^ G. Cherubini, DVM DECVN FRCVS^1^ ^,2^

##### 171.1.1.1 ^1^Dick White Referrals, Six Mile Bottom, Cambridgeshire, UK; ^2^Veterinary Teaching Hospital “Mario Modenato” Department of Veterinary Sciences University of Pisa, Pisa, Italy

Purpose: Describe the complication rate, expected hospitalization time, and outcome associated with dorsal laminectomy in the treatment of lateralized cervical intervertebral disc extrusion (IVDE) in dogs.

Methods: This is a single‐center retrospective case series study. Databases were reviewed from 2012 to 2022 for dogs that had a dorsal laminectomy to treat a lateralized cervical IVDE. Dogs were excluded if additional surgery techniques were performed, or other comorbidities were found on MRI.

Results: A total of 52 dogs were included in the study. French bulldogs represented 28.8% of the cohort. Patient average age was 5.75 years and the average weight 15.9 kg. Thirty‐five dogs (67.3%) presented clinical signs for < 3 days and almost half (44.2%) were ambulatory but presented cervical pain and neurological deficits. The average surgical time was 96.25 min. Minor intraoperative complications were reported in 22 (42.3%), with hypothermia being the most common. Thirteen (25%) needed revision surgery due to persistent cervical pain with (8/13) or without (4/13) neurological deficits. Re‐extrusion or persistent extrusion was found in 92.3% of cases needing revision. The average hospitalization time was 6.2 days. Forty‐seven (90.4%) cases had a good outcome.

Conclusion: Despite the relatively high rate of intraoperative complications and cases needing revision surgery, dorsal laminectomy used to treat lateralized cervical IVDE is still associated with good long‐term prognosis in the great majority of cases. The prognosis is good even when revision surgery is necessary.

## 172 (FP 12)

### 172.1 Effect of Gadolinium Contrast Medium Administration on Susceptibility‐Weighted Imaging (SWI) of the Canine Brain

#### 172.1.1 M. Mugnai, DVM, ^1^ E. Auriemma, DVM, Dipl. ECVDI, ^1^ B. Contiero, MSc in Statistics, ^2^ D. Franchini, DVM, PhD, ^3^ F. Tirrito, DVM, MRCVS, Dipl. ECVN^1^ ^,4^

##### 172.1.1.1 ^1^ AniCura Istituto Veterinario di Novara, Granozzo Con Monticello, Novara, Italy; ^2^Department of Animal Medicine, Production and Health (MAPS), University of Padova, Legnaro, Padova, Italy; ^3^Department of Veterinary Medicine, University of Bari, Valenzano, Bari, Italy; ^4^Studio Veterinario Associato Vet2Vet di Ferri e Porporato, Torino, Italy

Susceptibility‐weighted imaging (SWI) is a gradient echo (GE) magnetic resonance imaging (MRI) sequence. Intravenous administration of gadolinium (Gd) may affect the GE images but its effect on SWI has not been investigated in veterinary medicine. This prospective study aimed to evaluate the effects of Gd on SWI sequences. Seventy‐one dogs that underwent brain MRI were included and distributed in two groups. SWI was performed pre‐ and post‐contrast, the latter obtained immediately after Gd administration (Group A: 35 dogs) or delayed (Group B: 36 dogs). The pre‐ and post‐Gd SWI sequences were analyzed for signal intensity changes in the lentiform nuclei of the gray matter (GM), in the semioval center of the white matter (WM), and brain lesions. Overall brain lesions were identified in 29 (41%) cases with 17% and 83% showing, respectively, intra‐lesion signal void and contrast enhancement. No difference in GM signal intensity was identified in both groups (Group A, *p* = 0.395; Group B, *p* = 0.895) between pre‐ and post‐contrast images. In Group A, the WM signal intensity was lower in the pre‐ compared with the post‐Gd sequences (*p* = 0.019). For both groups, the signal intensity of the intracranial lesions was significantly lower in pre‐ than in post‐Gd images (*p* < 0.001); the number of lesions influenced the signal intensity difference in Group B (*p* = 0.043). Susceptibility artifacts did not change in appearance between pre‐ and post‐contrast images. In conclusion, Gd may modify the signal intensity of WM and brain lesions, but it doesn't affect the susceptibility artifacts and does not interfere with SWI interpretation.

## 173 (FP 13)

### 173.1 Magnetic Resonance Image (MRI) Pattern Recognition of Metabolic and Degenerative Encephalopathies in Dogs and Cats

#### 173.1.1 M. Miguel‐Garcés, MRCVS, Resident ECVDI, ^1^ E. Ives, MA, VetMB, DipECVN, MRCVS, ^2^ R. Gutierrez‐Quintana, MVZ, MVM, DipECVN, MRCVS, ^3^ K. Beckmann, DMV, DipECVN, ^4^ E. M. Galban, DVM, MS, DACVIM Neurology,^5^ P. Álvarez, DVM, DipECVN, MRCVS, EBVS,^6^ ^,7^ A. Vanhaesebrouck, DVM, CEAV (Int Med), DipECVN, DPhil, MRCVS, R. T. Bentley, BVSc (Dist), MRCVS, ACVIM Neurology, K. M. Santifort, MRCVS, Resident ECVN, S. Gomes, DVM, MRes, DipECVN, MRCVS, E. Alcoverro, DVM, DipECVN, MRCVS, K. Matiasek, DVM, DrMedVetHabil, FTA‐PATH‐NEUROPATH, AM‐ECVN,^12^ I. Carrera, DVM, MVM, PhD, DipECVDI, MRCVS^13^

##### 173.1.1.1 ^1^Department of Diagnostic Imaging, Southern Counties Veterinary Specialists, Ringwood, UK; ^2^Anderson Moores Veterinary Specialists, Winchester, UK; ^3^Small Animal Hospital, School of Biodiversity, One Health and Veterinary Medicine, University of Glasgow, Glasgow, UK; ^4^Vetsuisse Faculty, University of Zurich, Zurich, Switzerland; ^5^School of Veterinary Medicine, University of Pennsylvania, Philadelphia, USA; ^6^Pride Veterinary Centre, Derby, UK; ^7^Queen's Veterinary School Hospital, Cambridge University, Cambridge, UK; ^8^Department of Veterinary Clinical Sciences, Purdue University, West Lafayette, USA; ^9^Neurology Section, Evidensia Hospital Arnhem, Arnhem, The Netherland; ^10^Dovecote Veterinary Hospital, Derby, UK; ^11^ ChesterGates Veterinary Specialists, Chester, UK; ^12^Section of Clinical and Comparative Neuropathology, Institute of Veterinary Pathology, Centre for Clinical Veterinary Medicine, Ludwig‐Maximilians‐Universitaet‐Muenchen, Munich, Germany; ^13^Vet Oracle Teleradiology, Norfolk, UK

Metabolic brain diseases encompass a wide list of conditions that share similar clinical and magnetic resonance imaging (MRI) characteristics, challenging the diagnostic process and resulting in numerous tests performed to reach a definitive diagnosis. This multicentric, retrospective study evaluated a total of 65 patients with confirmed metabolic brain diseases. The aim was to describe the specific anatomic region affected by MRI and classify the conditions according to the involvement of gray matter (GM), white matter (WM), or both. These included lafora (9), thiamine deficiency (9), mielinolysis (3), hepatic encephalopathy (14), neuronal ceroid lipofuscinosis (NCL) (9), gangliosidosis (3), L‐2‐hydroxyglutaric aciduria (10), fucocidosis (1), exogenous intoxications (4), and cerebellar abiotrophy (3). Thiamine deficiency affected numerous deep gray nuclei with novel involvement of the oculomotor nuclei (3/9). All cases of myelinolysis had T2W hyperintensities of the thalamic nuclei and cerebrocortical GM/WM junction lateral to the internal capsule. None of the hepatic encephalopathies showed the previously described T1W hyperintensity of the lentiform nuclei. Instead, T2W hyperintensity of the WM (specifically corona radiata) was seen in 8/14 with/without brain atrophy (10/14) and concomitant cerebellar atrophy (4/10). All cases of NCL presented severe cerebral and cerebellar atrophy with diffuse T2W hyperintensity of the WM (7/9). L‐2‐hydroxyglutaric aciduria had the characteristic T2W hyperintense swelling of the cerebral and cerebellar cortices in all cases. All intoxications had increased brain volume. This study showed the important role of MRI in distinguishing different metabolic disorders according to specific imaging characteristics.

## 174 (FP 14)

### 174.1 Titanium Cranioplasty Following Head Trauma in Two Growing Dogs

#### 174.1.1 J. Abouzeid, BVM BVS BVMedSci,^1^ A. Anson, DVM PhD DECVDI,^2^ A. Uriarte, Dip ECVN MRCVS DVM^1^

##### 174.1.1.1 ^1^Neurology and Neurosurgery Department, Southfields Veterinary Specialists, Linnaeus Ltd, Basildon, UK; ^2^Clinical Sciences Department, Cummings School of Veterinary Medicine at Tufts University, North Grafton, USA

In pediatric neurosurgery, titanium implants in cranioplasty have been shown to be appropriate in cases with large bone defects. However, the use of such rigid implants in growing skulls remains controversial. In growing rabbits, it has been shown that mini‐plate fixation across the coronal suture results in restricted growth of the calvarium. Similar findings have been reported in infant rhesus monkeys. Therefore, it is recommended to avoid important suture lines when performing cranioplasty in skeletally immature patients. While autografts and demineralized bone grafts for cranioplasty have been reported in the growing canine skull, rigid alloplastic materials such as titanium have not. Currently, no guidelines exist for using rigid implants in cranioplasty in growing dogs. Here we present two cases of skeletally immature dogs that underwent cranioplasty to repair skull fractures following traumatic dog bites. In both cases, a large titanium implant was used and fixed in place using cortical screws within the same bone plate, thus respecting important suture lines while allowing defect coverage as the skull continued to grow. At the 4‐week post‐operative assessment, no significant complications were reported in either case. One case is reported by their owner to be normal with no cosmetic deformities of the skull 4 years after surgery. As far as the authors are aware, this is the first report of titanium mesh implant used for cranioplasty in skeletally immature dogs. The use of titanium implants maybe suitable for growing dogs with large defects, provided caution is exercised to avoid suture lines, preventing possible growth defects.

## 175 (FP 15)

### 175.1 En Bloc L3 Vertebrectomy for a Bone Tumor in a dog. Surgical Technique, Reconstruction, and Stabilization of the Dorsal and Ventral Compartment Using a Tailor‐Made Titanium Implant

#### 175.1.1 R. Jiménez González, DVM PgDipSAS GPCertSAS GPCertNeuro MRCVS,^1^ A. Wessmann, DrMedVet DipECVN PGCertAcPrac FHEA MRCVS,^1^ R. Vallefuoco, DVM DESV DECVS MRCVS,^2^ J. J. Minguez, DVM Acr AVEPA in Neurology MRCVS^1^

##### 175.1.1.1 ^1^Neurology and Neurosurgery Department, Pride Veterinary Centre Derby, Part of IVC Evidensia Group, Derby, UK; ^2^Surgery Department, Pride Veterinary Centre Derby, Part of IVC Evidensia Group, Derby, UK

The presenting case report aims to describe a detailed surgical technique of an en bloc L3 vertebrectomy, followed by reconstruction and stabilization of the vertebral segment using a tailor‐made titanium implant to treat an L3 osteosarcoma in a dog. An 8‐year‐old 27.8 kg male neutered American Staffordshire Terrier Cross with a 4‐week history of chronic, progressive, painful, symmetrical ambulatory paraparesis and proprioceptive ataxia was diagnosed with a monostotic, lytic, expansile, solid lesion in the L3 vertebral body and left vertebral arch suggestive of a bone tumor. The surgical procedure was planned in three different stages: (1) vertebral skeletonization, (2) dorsal and ventral compartment removal, and (3) vertebral segment reconstruction with a vertebral spacer with an integrated bone plate and locking screws, a contralateral locking plate, and a titanium mesh with locking screws for the dorsal compartment. Immediate postoperative CT scan showed complete L3 vertebra removal, accurate vertebral segment reconstruction, and correct placement of all implants. There was no deterioration after surgery; the patient remained ambulatory and was discharged 3 days later. Histopathological examination revealed osteosarcoma. Carboplatin was administered as adjuvant chemotherapy. En bloc vertebrectomy was elected due to its potential curative effect compared with other palliative treatments. Although a challenging surgical procedure, it provides better local tumor control and better outcome in people with malignant vertebral tumors. En bloc vertebrectomy represents a therapeutic option in current veterinary medicine. To the authors' knowledge, this case report is the first en bloc vertebrectomy and vertebral reconstruction with a tailor‐made titanium implant performed in the United Kingdom.

## 176 (FP 16)

### 176.1 Prevalence, Clinical Presentation, and Etiology of Myelopathies in 224 Juvenile Dogs

#### 176.1.1 E. Pilkington, MA VetMB, PGDipVCP, AFHEA, MRCVS,^1^ S. De Decker, DVM, PhD, MVetMed, DipECVN, FHEA, PGCert VetEd, MRCVS,^2^ E. Skovola, DVM, MRCVS,^2^ A. Cloquell Miro, DVM, PGCert, MSc, MRCVS,^3^ R. Gutierrez Quintana, MVZ, MVM, DipECVN, FHEA, MRCVS,^3^ A. Aguilera Padros, DVM, MRCVS,^4^ K. Faller, DVM, DPhil, DipECVN, MRCVS,^4^ R. Goncalves, DVM, MVM, DipECVN, FHEA, MRCVS^1^

##### 176.1.1.1 ^1^Small Animal Teaching Hospital, Institute of Veterinary Science, University of Liverpool, Neston, UK; ^2^Department of Clinical Science and Services, Royal Veterinary College, University of London, Hatfield, UK; ^3^Small Animal Hospital, School of Biodiversity, One Health and Veterinary Medicine, Univerity of Glasgow, Glasgow, UK; ^4^The Royal (Dick) School of Veterinary Studies, The University of Edinburgh, Edinburgh, UK

Intervertebral disc herniation is widely recognized as the most common cause of myelopathy in dogs older than 2 years. However, the prevalence of various causes of myelopathies in younger dogs has not been previously described. This multicenter retrospective study reviewed medical records of dogs presenting with myelopathic signs at 18 months of age or less. Dogs were excluded if they displayed spinal pain only or if a final diagnosis could not be reached. Multivariate logistic regression was used to determine clinical features associated with a diagnosis of the two most common causes of myelopathy. Two hundred twenty‐four dogs were included, with French bulldogs (*n* = 51, 22.8%), pugs (*n* = 18, 8.0%), crossbreeds (*n* = 12, 5.4%), and bulldogs (*n* = 11, 4.9%) the most frequently affected breeds. Myelopathies were most commonly chronic (*n* = 123, 54.9%), and had a thoracolumbar neuroanatomic localization (*n* = 103, 46.0%). In total 32 different diagnoses were reached. The five most frequent were vertebral malformation (*n* = 42, 18.8%), spinal arachnoid diverticulum (*n* = 28, 12.5%), traumatic fracture of the vertebral column (*n* = 22, 9.8%), atlantoaxial instability (*n* = 18, 8.0%) and osseous‐associated cervical spondylomyelopathy (*n* = 17, 7.6%). Intervertebral disc extrusion accounted for 4.5% of diagnoses (*n* = 10). Duration of clinical signs (days) before diagnosis was the only clinical feature that reached statistical significance (*p* = 0.03, OR = 0.991, 95% CI: 0.982–0.999) when comparing vertebral malformation and spinal arachnoid diverticulum, with median values of 21 and 60 days, respectively. Juvenile canine myelopathies were reported most frequently in small brachycephalic breeds. Vertebral malformation and spinal arachnoid diverticulum were the two most common etiologies.

## 177 (FP 17)

### 177.1 Predicting Survival at 6 Months in Canine Meningoencephalitis of Unknown Origin

#### 177.1.1 R. Goncalves, DVM, MVM, DipECVN, FHEA, MRCVS,^1^ S. De Decker, DVM, PhD, MvetMed, DipECVN, FHEA, PGCert Veted, MRCVS,^2^ T. Maddox, BVSc, PhD, PGCertHE, CertVDI, DipECVDI, MRCVS,^1^ G. Walmsley, MA, VetMB, PhD, DipECVN, FHEA, MRCVS^1^

##### 177.1.1.1 ^1^Department of Veterinary Science, University of Liverpool, Neston, Cheshire, UK; ^2^Department of Clinical Science and Services, Royal Veterinary College, University of London, Hatfield, UK

This study aimed to identify clinical prognostic variables associated with survival at 6 months after diagnosis and/or long‐term relapse in dogs with suspected meningoencephalitis of unknown origin (MUO). A retrospective study of 447 dogs presented to two UK referral hospitals and diagnosed with presumptive MUO using multivariable logistic regression for the identification of risk factors. The median age at presentation was 48 months (IQR 26–73) and 55% of dogs were female. The mean neurodisability scale (NDS) score at presentation (calculated retrospectively based on clinical records) was 6 ± 3.1. Eighty‐two percent (366/447) of dogs survived to discharge and 63.5% of dogs (284/447) were alive at 6 months. The median follow‐up time was 11 months (IQR 1–24) and 50.6% of dogs (160/316) relapsed during treatment (median time to relapse 7 months, IQR 2–15). Seizures (*p* < 0.001, OR = 0.318, 95% CI: 0.199–0.507), paresis (*p* = 0.001, OR = 0.44, 95% CI: 0.276–0.7), and higher NDS score (*p* < 0.001, OR = 0.818, 95% CI: 0.755–0.886) at presentation were associated with decreased survival to 6 months. Lower weight (*p* = 0.023, OR = 0.969, 95% CI: 0.943–0.996) and higher NDS score (*p* = 0.001, OR = 1.162, 95% CI: 1.064–1.270) were associated with an increased risk of relapse. Cytarabine was administered at the time of diagnosis in 74% (subcutaneous injection in 40% and constant rate infusion in 34%) and neither was associated with survival or relapse. Knowledge of risk factors associated with survival and relapse in MUO can help guide monitoring and treatment and improve owner communications regarding the prognosis for this debilitating disease.

## 178 (FP 18)

### 178.1 Phenotypic Characterization of a Novel Episodic Ataxia in Young Working Cocker Spaniels

#### 178.1.1 C. Sarro, DVM MRCVS,^1^ C. Stalin, MA VetMB MEd (HE) DipECVN MRCVS,^1^ R. Gutierrez‐Quintana, MVZ MVM DipECVN MRCVS,^1^ A. Cloquell, DVM PGCert MSc MRCVS^1^

##### 178.1.1.1 ^1^Small Animal Hospital, School of Veterinary Medicine, University of Glasgow, Glasgow, UK

In human medicine, paroxysmal movement disorders are divided into paroxysmal dyskinesia or episodic ataxia (EA). Episodic ataxia is a heterogeneous group of disorders characterized by recurrent and paroxysmal cerebellar dysfunction presenting as spells of truncal ataxia and discoordination. Primary EA has been anecdotally described in veterinary literature. Our objective is to describe the phenotypic characteristics in a cohort of seven young working cocker spaniels (WCS) presenting with neurological signs compatible with EA. Clinical signs, video footage, and investigations performed in seven WCS with episodic ataxia were reviewed. Moreover, owners of affected dogs were invited to complete a specifically designed questionnaire. The mean age of clinical onset was 4 months (from 3 to 5 months). In all patients, the episodes were characterized by sudden body and head swaying and cerebellar ataxia with normal mentation and posture. Duration of episodes was variable (usually a few hours), with frequency varying widely between dogs. Neurological examination was normal in all dogs between episodes. Hematology and biochemistry were normal in all dogs. When MRI, CSF analysis, cardiac scan, and urinary organic acids measurement were performed, the results were unremarkable. Episodes were decreasing in frequency without treatment or after a strict gluten‐free diet. This descriptive study aims to provide a precise description of a recently recognized movement disorder in WCS and draw the attention of other clinicians to promote collecting information from new cases. A breed predisposition and potential genetic origin are hypothesized, but future studies are needed.

## 179 (FP 19)

### 179.1 Safe Corridors for the Treatment of Vertebral Fractures and Luxations With Screws and Polymethylmethacrylate via Lateral Approach

#### 179.1.1 R. Lamère, DVM,^1^ S. Scotti, DVM, PhD,^1^ M. Saccone, DVM, PhD,^2^ C. Ragetly, DVM, PhD, DACVS DECVS^1^

##### 179.1.1.1 ^1^Private Practitioner, L'Isle Adam, France; ^2^Private Practitioner, Caserte, Italy

Introduction: Surgical treatment of vertebral fractures and luxations is challenging due to the positioning of the implants. The objectives of the study were to assess for safety and learning curve of a lateral vertebral corridor with an inclination angle of 90° within the vertebral body for screws and polymethylmethacrylate implantation and to evaluate the prognosis in a clinical setting. Materials and methods Medical records of 30 dogs and cats surgically treated for vertebral injuries were reviewed (2008–2022). Twenty CT scans of the canine spine were assessed to define the safe implantation corridors in the transverse plane. One hundred and four corridors were drilled on 10 dog cadavers by a novice neurosurgeon and deviation from the angle of 90° was evaluated on CT in chronological order to assess the learning curve.

Results: Overall, 17/30 animals (56.7%) regained the ability to walk without assistance post‐operatively, and 3/11 animals (27.3%) had lost deep pain sensation. There were 3/30 (10%) minor complications and 8/30 (26.7%) major complications, including peri‐operative deaths and euthanasia. CT revealed that lateral corridors were safe and effective. A progressive reduction of the deviation between the angle measured and the ideal insertion angle was observed in the cadaveric part of the study with significative results from the fifth case (*p* < 0.001).

Discussion/Conclusions: This lateral approach with screws embedded in polymethylmethacrylate to treat vertebral injury is safe and effective in a clinical setting, and the learning curve is short.

## 180 (FP 20)

### 180.1 Fluttering Ears: A New Form of Myoclonic Epilepsy in Dogs?

#### 180.1.1 E. Lyon, DVM,^1^ ^,2^ H. Pochat, CEO,^3^ S. Besnard, MD, PhD,^2^ C. Escriou, DVM, PhD^1^

##### 180.1.1.1 ^1^Unité de Neurologie, CHUVAC, VetAgro Sup, Marcy l'Etoile, France; ^2^Vertex Laboratory, UFR STAPS Department, Caen, France; ^3^Amset Medical, Saint‐Maur‐Des‐Fossés, France

We present a case of focal myoclonus resulting in abnormal ear and neck movements in a 1‐year‐old pregnant Dalmatian bitch. The dog was presented for abnormal and continuous ear movements from 1 week evolution. She may have been exposed to Datura in the previous week and she had been pregnant for 35 days at the time of onset of symptoms. Clinical and neurological examination was normal except for ear fluttering and intense myoclonic bursts on the neck. The dog remained bright and alert with normal behavior. Hematology and biochemistry were normal. Due to the pregnancy of their dog, the owner refused brain MRI and CSF analysis necessitating anesthesia. A video electroencephalogram (EEG) was performed without anesthesia using 8 gel electrodes. Spikes and sharp waves localized in the temporo‐rolandic region and diffusing to the right hemisphere were observed simultaneously with clonic ear movements and were evocative of myoclonic epilepsy. Phenobarbital was initiated and a reduction of myoclonic ear movements was immediately observed, confirmed 15 days later by a follow‐up EEG. Increased dosage of phenobarbital allowed for complete remission of myoclonic ear movements. Phenobarbital dosage was gradually reduced after 6 months of complete remission and completely stopped after 1 year without relapse. We concluded to an unusual form of myoclonic epilepsy. Its origin remains undetermined (link with pregnancy? Datura toxicity? Idiopathic?). To our knowledge, this type of myoclonic epilepsy has never been reported in dogs, but we have been informed by their attending veterinarian of 3 similar cases in Golden Retrievers responding to phenobarbital treatment suggesting that it could be from a myoclonic idiopathic epilepsy.

## 181 (FP 21)

### 181.1 RALGAPA1 Deletion in Malinois Puppies With Cerebellar Ataxia

#### 181.1.1 E. Hünerfauth, Dr,^1^ M. Christen, PhD Cand,^2^ M. Leschnik, Dr,^5^ I. Zdora, PhD Cand,^3^ V. Jagannathan, PhD,^2^ C. Puff, Dr,^3^ W. Baumgärtner, Prof Dr,^3,4^ M. Kleiter, Prof,^5^ T. Leeb, Prof Dr,^2^ H. Volk, Prof Dr^1^

##### 181.1.1.1 ^1^Department for Small Animal Medicine and Surgery, University of Veterinary Medicine, Hannover, Germany; ^2^Institute of Genetics, Vetsuisse Faculty, University, Bern, Switzerland; ^3^Department of Pathology, University of Veterinary Medicine, Hannover, Germany; ^4^Center of Systems Neuroscience, Hannover Graduate School for Neurosciences, Infection Medicine, and Veterinary Sciences (HGNI), Hannover, Germany; ^5^Department for Companion Animals and Horses, University of Veterinary Medicine, Vienna, Austria

Three Malinois puppy siblings were presented with a history of chronic, slowly progressive cerebellar signs. The two male and one female, 5 weeks old puppies had an unremarkable general examination. The neurological examination revealed an ambulatory cerebellovestibular ataxia, predominantly symmetrical, with mild proprioceptive deficits. Paresis was not noted. The menace responses were absent. Occulovestibular response was normal to reduced. A mild intention tremor of the head was noted. One puppy also showed behavioral alterations, interacting abnormally with its environment. The neuroanatomical localization was therefore for the two puppies the cerebellum, whereas for the last puppy a multifocal localization including the cerebellum and the forebrain were suspected. Magnetic resonance imaging of the brain of the puppies revealed merely a slight reduction of the presentation of the Uvula and Pyramis area of the cerebellum in T2‐weighted sagittal sequences. Due to the progression of clinical signs, the puppies were euthanized. Histopathologically, in all three puppies, the Purkinje cells of the cerebellum revealed multifocal, slightly to moderate accumulations of intracytoplasmic, slightly basophilic material with a granular appearance. The Purkinje cells were stained positive with luxol fast blue. Genetic analysis revealed a homozygous deletion harboring exon 35 of the RALGAPA1 gene. It has been detected in a homozygous state in the puppies presented herein and two further ones. RALGAPA1 gene defects cause psychomotor disability and early‐onset epilepsy in humans. RALGAPA1 gene defects need to be considered for Malinois puppies presenting with cerebellar ataxia.

